# Thiolutin extends replicative lifespan by rewiring yeast transcription and metabolism

**DOI:** 10.1038/s41598-026-42387-1

**Published:** 2026-03-01

**Authors:** Mateusz Mołoń, Patrycja Kielar, Zofia Kobylińska, Agnieszka Mołoń, Jarosław Winiarski, Monika Kula-Maximenko, Sabina Galiniak

**Affiliations:** 1 Faculty of Biology, Natural Protection, and Sustainable Development, University of Rzeszow, al. Tadeusza Rejtana 16C, 35-959 Rzeszow, Poland; 2 Faculty of Medicine, University of Rzeszów, al. Tadeusza Rejtana 16C, 35-959 Rzeszow, Poland; 3 The Franciszek Górski Institute of Plant Physiology, Polish Academy of Sciences, 30-239 Kraków, Poland

**Keywords:** Aging, Longevity, Raman spectroscopy, Replicative lifespan, RNA-Seq, Thiolutin, Biochemistry, Cell biology, Molecular biology

## Abstract

Transcription is a major cellular energy sink tightly coupled to growth, metabolism, and aging. Thiolutin, a widely used RNA polymerase inhibitor in yeast, has unclear long-term effects on aging. We show that thiolutin oppositely affects replicative and chronological aging in *Saccharomyces cerevisiae* by remodeling transcription, metabolism, and proteostasis. Thiolutin extends replicative lifespan, increasing reproductive potential and prolonging the mitotic phase, despite slower growth and lower ATP. This longevity is linked to global transcriptional repression, including reduced ribosome biogenesis, translation, and mitochondrial oxidative phosphorylation, and dampening of TOR1-dependent growth programs. In parallel, thiolutin triggers a selective adaptive response with activation of RPN4-mediated proteasome remodeling and redox-responsive genes, without HOG1 induction. Conversely, thiolutin accelerates early chronological aging: post-mitotic survival drops alongside repression of reserve carbohydrate genes (GPH1, GSY2, TSL1), suggesting impaired adaptation when entering stationary phase. FT-Raman spectroscopy confirms coordinated depletion of RNA, proteins, lipids, and carbohydrates. Thus, thiolutin promotes a low-energy, stress-adaptive state that benefits budding yeast cells but compromises early survival of non-budding populations, underscoring transcription–energy coupling in aging trajectories.

**Supplementary Information:**

The online version contains supplementary material available at 10.1038/s41598-026-42387-1.

## Introduction

Thiolutin is a naturally occurring compound recognized for its role as a transcriptional inhibitor in yeast. It is part of the dithiolopyrrolone family, which is distinguished by redox-sensitive disulfide bonds. Thiolutin is extensively utilized in research to investigate mRNA stability and transcriptional regulation, primarily due to its ability to inhibit RNA polymerase II (Pol II) activity^1,2^.

In yeast, thiolutin has been demonstrated to obstruct transcription initiation, making it an essential tool for examining the dynamics of mRNA synthesis and degradation^2^. Its mechanism of action is intricate, involving interactions with various cellular pathways, including oxidative stress response and metal ion chelation^1^. Recent studies have indicated that thiolutin’s effects are not confined to transcription inhibition but also encompass mRNA degradation pathways, which can complicate its application in certain experimental contexts^2^. Overall, thiolutin remains a crucial compound for studying transcriptional processes in yeast, offering valuable insights into the complex mechanisms of gene expression regulation.

Transcription in yeast is an energy-consuming process that involves the synthesis of RNA from DNA templates^3^. This process requires significant amounts of ATP, which is used to fuel the activities of RNA polymerases. In yeast cells, transcription is a highly energy-demanding process, with distinct RNA polymerases contributing differently to the overall energy expenditure. RNA polymerase I is primarily responsible for the synthesis of ribosomal RNA (rRNA), in turn polymerase III transcribes tRNA and 5 S rRNA. The transcriptional activities of RNA polymerase I and RNA polymerase III are predominant in growing cells, together accounting for over 80% of total RNA synthesis^4^. RNA polymerase II, the main enzyme involved in mRNA synthesis, contributes about 20% to the total transcriptional activity.

Inhibition of RNA polymerase I disrupts ribosomal RNA synthesis, thereby impairing ribosome biogenesis and reducing protein production ‒ effects that are especially detrimental to rapidly proliferating cells, such as those found in tumors, making Pol I a promising target in cancer therapy. Suppression of RNA polymerase II activity halts the transcription of messenger RNAs and various regulatory non-coding RNAs, ultimately blocking the synthesis of proteins required for cell viability and division. Similarly, inhibition of RNA polymerase III interferes with the production of transfer RNAs and other small RNAs, which are critical for translation and other fundamental cellular functions^5^.

The energy consumption during transcription is influenced by several factors: RNA polymerase activity, chromatin remodeling, the binding and activity of transcription factors, post-transcriptional modifications, such as capping, splicing, and polyadenylation of mRNA^6–8^.

Aging is a multifaceted and intricate process impacting all living organisms, including the unicellular yeast *Saccharomyces cerevisiae*. Extensive research over the years has elucidated numerous factors influencing both the replicative and chronological lifespan of this widely utilized model organism in aging studies^9,10^. The relationship between aging and transcription inhibition by thiolutin remains unclear and not yet fully understood. An intriguing aspect of yeast aging involves thiolutin, a naturally occurring compound known for its diverse biological activities. The regulation of protein synthesis is a critical aspect of yeast aging, as translation is a major energy-consuming process. This highlights the importance of understanding the intricate connections between gene expression, metabolism, and the longevity.

The aim of this study was to demonstrate the impact of thiolutin, a known RNA polymerase inhibitor, on cellular aging and metabolic state. Our research showed that thiolutin treatment was correlated with delayed replicative aging and increased reproductive potential of cells. Additionally, we demonstrated that thiolutin activity leads to significant changes in the chemical composition of cells. Furthermore, RNA-Seq analyses revealed significant changes in the transcriptome.

## Materials and methods

### Strains and growth conditions

In this study, the reference strain BY4741 *MATa his3Δ1 leu2Δ0 met15Δ0 ura3Δ0* (Euroscarf) was utilized. The yeast cells were cultivated in a standard liquid YPD medium (comprising 1% Difco Yeast Extract, 1% Yeast Bacto-Peptone, and 2% glucose) on a rotary shaker set at 160 rpm. Alternatively, yeast were grown on a solid YPD medium with 2% agar for assessing reproductive potential, or on a solid YPD medium with 2% agar and Phloxine B for evaluating total lifespan. For the chronological lifespan (CLS) experiment, *S. cerevisiae* cells were cultured in synthetic defined complete (SDC) medium. SDC medium consisted of 0.67% yeast nitrogen base without amino acids (YNB), 2% glucose, and a complete supplement mixture providing the required amino acids and nucleotides for auxotrophic selection. The experiments were conducted at a temperature of 28 °C.

###  Growth rate determination

Growth kinetics of yeast cultures were assessed in liquid medium under controlled laboratory conditions. Cell suspensions were incubated at 28 °C for a duration of 12 h with continuous agitation using a Heidolph Incubator 1000 set to 1200 revolutions per minute. Optical density at 600 nm (OD₆₀₀) was recorded at 2-h intervals throughout the incubation period using an Anthos 2010 microplate reader (model 17550) to monitor cell proliferation.

### Calculation of the mean doubling time

The average doubling time was determined in accordance with the methodology described in a previously published study^11^. Doubling time calculations were integrated into the standard procedure for assessing replicative lifespan. A total of at least 90 individual cells were analyzed across at least three independent experimental replicates. Results are presented as mean values accompanied by standard deviations (SD). Statistical significance was evaluated using one-way analysis of variance (ANOVA), with a threshold of *p* < 0.001.

### Determination of replicative lifespan

Following overnight incubation, cells were arranged on a rich YPD medium plate with the aid of a micromanipulator. The budding lifespan was evaluated microscopically, in accordance with the methodology described in^12^. Cellular analysis was performed on a minimum of 90 individual cells across three biologically independent experiments. Microscopic observations were carried out using a Nikon Eclipse E200 optical microscope (Nikon, Amsterdam, Netherlands) equipped with a high-precision micromanipulator.

### Determination of the total lifespan

The total lifespan of an individual *S. cerevisiae* mother cell was defined as the cumulative duration of its life, measured in units of time. This parameter was calculated by summing the reproductive lifespan (the interval between the first and last budding events) and the post-reproductive lifespan (the period from the final budding to cell death). Lifespan assessment was conducted following the methodology described in^13^, with minor modifications based on the protocol outlined in^12^. Yeast cultures in the exponential growth phase were sampled in 10-µL aliquots and transferred onto YPD agar plates supplemented with Phloxine B (10 µg/mL). Phloxine B is a vital dye that accumulates in cells with impaired membrane function, allowing for the identification of non-viable cells. While healthy, metabolically active cells exclude the dye, damaged or dying cells retain it and become visibly stained, typically appearing red or pink under microscopic observation or on agar plates. In each experimental replicate, 45 individual cells were analyzed. During the experimental procedure, plates were maintained at 28 °C for 15 h and subsequently stored at 4 °C overnight. A minimum of 90 cells were evaluated across at least three independent experiments. Lifespan determination was performed via micromanipulation using a Nikon Eclipse E200 optical microscope equipped with a micromanipulator.

### Chronological lifespan (CLS) assays

Chronological lifespan (CLS) of *S. cerevisiae* cells cultured in synthetic defined complete (SDC) medium was assessed as previously described. Briefly, yeast cultures were grown in SDC supplemented with 2% (w/v) glucose and the appropriate auxotrophic requirements. Cell viability was monitored at defined time points ‒ days 2, 4, 7, 14, 21, and 28 ‒ during incubation. Viability was quantitatively assessed using propidium iodide staining. Reported values represent means derived from a minimum of three independent biological replicates.

### Measurement of ATP content

Intracellular ATP levels were quantified using the BacTiter-Glo™ Microbial Cell Viability Assay Kit (Promega), following the manufacturer’s instructions. Yeast cells harvested from cultures in the exponential growth phase were washed with sterile distilled water and resuspended to a final concentration of 10⁶ cells/mL in 100 mM phosphate buffer (pH 7.0) supplemented with 0.1% glucose and 1 mM sodium EDTA. For each measurement, 100 µL of the cell suspension was used. Luminescence, which is directly proportional to ATP concentration, was measured using a TECAN Infinite 200 microplate reader (Tecan Group Ltd., Switzerland). The experiments were conducted in ten independent biological replicates (*n* = 10).

### RNA sequencing and analysis of RNA-Seq data

Total RNA was extracted using the Invitrogen RNA extraction kit (AM1926, Invitrogen, Waltham, MA, USA) according to the manufacturer’s protocol. For each extraction, 2 × 10^6^ freshly harvested cells from overnight cultures were used. RNA quality and yield were assessed using a Tecan Infinite 200 microplate reader. Aliquots of approximately 5 µg were stored at − 80 °C at a concentration of 300 ng/µL. Library preparation was performed using the MGIEasy RNA Library Prep Set (MGI Tech), and sequencing was carried out using DNBSeq technology on the BGISEQ-500 platform at BGI’s Hong Kong facility. Data represent the average of two independent experiments. Differential expression was called with DESeq2 using Benjamini–Hochberg (BH) FDR < 0.05 and |log2 fold-change| ≥ 1. All RNA-Seq samples were collected after 3 h exposure to 3 µg/mL thiolutin. Unless stated otherwise, percentages refer to protein-coding mRNAs. Gene Ontology enrichment was performed with topGO (elim) using the BH FDR correction; the background was set to the union of expressed protein-coding genes across samples. Gene identifiers for significantly differentially expressed transcripts were retrieved using the BioMart tool, focusing on those most frequently represented among the significant comparisons.

### Reverse transcription, and RT–qPCR analyses

Reverse transcription, and RT–qPCR analyses were performed in accordance with a previously established and validated protocol, as described earlier, with no substantial modifications^14^. To eliminate contaminating genomic DNA and RNA–DNA hybrids, RNA samples were treated with RNase-free DNase I (50 U; A&A Biotechnology, Gdynia, Poland) for 30 min at 37 °C in a buffer containing 50 mM Tris-HCl (pH 8.0) and 5 mM MgCl₂ (TBD-100 thermoblock, Biosan, Riga, Latvia). DNase activity was terminated by heat inactivation at 75 °C, followed by rapid cooling to 4 °C. Reverse transcription was performed using a two-step approach. First-strand cDNA synthesis was carried out with the TranScriba Kit (A&A Biotechnology, Gdynia, Poland) in a MasterCycler^®^ thermal cycler (Eppendorf, Hamburg, Germany). Briefly, approximately 1 µg of total RNA was incubated with 1 µL of oligo(dT)₁₈ primer at 65 °C for 5 min in a final volume of 9.5 µL, followed by cooling to 4 °C. Subsequently, a reaction mixture containing 5× reaction buffer, RNase inhibitor (40 U/µL), dNTPs (10 mM), and recombinant MMLV reverse transcriptase (20 U/µL) was added, and the reaction volume was adjusted to 20 µL with nuclease-free water. Reverse transcription was conducted at 42 °C for 60 min and terminated by heating at 70 °C for 5 min. Synthesized cDNA was stored at − 20 °C until quantitative analysis. Quantitative real-time PCR (RT-qPCR) was performed using a LightCycler^®^ 96 system (Roche, Basel, Switzerland). Each reaction (15 µL total volume) contained 7.5 µL of 2× SYBR^®^ Green RT-PCR Mix (A&A Biotechnology; including Taq DNA polymerase, MgCl₂, and dNTPs), 0.56 µL of each gene-specific primer (10 µM; Genomed, Warsaw, Poland; see Table 1), and 0.75 µL of cDNA diluted 1:5 in nuclease-free water. Amplification conditions consisted of an initial denaturation at 95 °C for 180 s, followed by 40 cycles of denaturation (95 °C, 30 s), annealing (53 °C, 30 s), and extension (72 °C, 30 s), with fluorescence acquisition at the end of each cycle. Specificity of amplification was confirmed by melt-curve analysis (95 °C for 10 s, 65 °C for 60 s, followed by a gradual increase to 97 °C with five measurements per °C). Gene expression levels were normalized to the reference gene *ACT1*. Each experiment included at least three independent biological replicates and three technical replicates. Negative controls without template and positive controls containing yeast genomic DNA were included in each run. Absence of genomic DNA contamination was verified by conventional PCR using *ACT1* primers followed by agarose gel electrophoresis. All procedures were conducted in accordance with established best practices for RNA handling and quantitative gene expression analysis.

**Table 1:** Primers used for RT-qPCR gene expression analysis.

Gene name	Forward Primer 5′->3′	Reverse Primer 5′->3′
ACT1	GTAAGGAATTATACGGTAACATC	TAGATGGACCACTTTCGTCG
HOG1	CGGTACAGTTTTCGAGATCACA	AGGCAAATCAAGTTCTCGTGT
TOR1	TGGATAGAAATGTGCCGTTGG	CGTTCCTCCTTGTAATCACTGG
RPN4	CCAGGTCACAGTCAGTTTACG	CGAAGCTGCACTCGATTCAT
GPH1	TCCCAAGGCTTACAAGGAGA	ACGTGCTAATGTAGTTTCCACA
GSY2	TCCGTGCTAAAATCGAAGGC	TTCAATCAGCCACCTCCCAT
TSL1	TCGAGCTTGACACCTCTCTC	GCGGTTGAAAGCACTGGG
ATP19	GCTTATCATTTCATGGGGAAAG	CGCATCTTGCTTTTCCGAATG
COX26	CCTAAACAAACGCTCAATACT	CTGCCCCAATCAAGAGGTA
SOD1	AGTGTTAAAGGGTGATGCC	CACCGACATGTCTGACTTC
YAP1	TAGCGGAGTTTGAGGGTTCA	TCCTCTCCTTACGTTCCCTAA

### Raman spectroscopy

Raman spectroscopy was utilized to characterize the biochemical composition of *S. cerevisiae* cells. Spectroscopic measurements were performed on lyophilized samples using a Nicolet NXR 9650 FT-Raman Spectrometer equipped with an Nd: YAG laser (1064 nm) and an InGaAs detector. Spectra were acquired with a laser power of 0.5 W, a beam diameter of 50 μm, and a spectral resolution of 8 cm⁻¹. Data were collected over the spectral range of 250–1800 cm⁻¹, averaged across 64 scans. Spectral processing and analysis were conducted using Omnic (Thermo Scientific) and OriginLab software. To identify clustering patterns among samples, Principal Component Analysis (PCA) and Hierarchical Clustering Analysis (HCA) were applied to spectral regions associated with polysaccharides, lipids, and RNA. Yeast cells were harvested during the exponential growth phase following a 3 h treatment with 3 µg/mL thiolutin or without treatment (control), washed four times with sterile deionized water, lyophilized, and subsequently subjected to FT-Raman spectroscopy under standardized conditions. Six biological replicates were used.

### The area of FT-Raman spectra

The area of the FT-Raman spectra was analyzed in the range corresponding to bands typically dominated by contributions from RNA and proteins across six independent repetitions, and the results are presented as means. The significance of differences in mean values between groups was assessed using Tukey’s post-hoc test at a significance level of *p* ≤ 0.05. Statistical analysis was performed using OriginLab 2020 software.

### Cell cycle analysis using Muse™ cell analyzer

To assess the effect of thiolutin on cell cycle progression, the Muse™ Cell Cycle Kit (Cytek) was utilized according to the manufacturer’s instructions. Briefly, yeast cells were cultured to mid-logarithmic phase, harvested by centrifugation at 6000 rpm for 2 min, and resuspended in YPD medium. Cells were then treated with 3 µg/mL thiolutin for 3 h. Following treatment, cells were washed with phosphate-buffered saline (PBS), and the pellet was fixed in 70% ice-cold ethanol. Fixed cells (200 µL) were centrifuged again and washed with fresh PBS. Subsequently, 200 µL of Muse™ Cell Cycle reagent was added to the cell pellet, and samples were incubated in the dark for 30 min at room temperature. Cell cycle distribution was analyzed using the Muse™ Cell Analyzer.

### Assessment of superoxide anion production

Intracellular production of reactive oxygen species, specifically the superoxide anion, was quantified using dihydroethidine (DHET) as a fluorescent probe (final concentration 18.9 µM), following a modified protocol based on previously described methods. Yeast cells were harvested during the exponential growth phase, washed with sterile distilled water, and resuspended to a final density of 1 × 10⁸ cells/mL in 100 mM phosphate buffer (pH 7.0) supplemented with 0.1% glucose and 1 mM sodium EDTA. Oxidation-dependent fluorescence of DHET was monitored kinetically using a TECAN Infinite 200 microplate reader, with excitation and emission wavelengths set to 518 nm and 605 nm, respectively. All measurements were performed at 28 °C^15^.

### Statistical analysis

The results are presented as mean ± SD for all samples tested across three independent experiments. Differences between control BY4741 untreated and treated were evaluated using one-way ANOVA with Dunnett’s post hoc tests. Statistical significance was set at *p* < 0.05. Statistical analysis was executed using Statistica 10.0 (Statsoft, Tulsa, Oklahoma, USA) while both statistical and multidimensional analyses were performed with PAST 3.0 and Origin 2018 software (Raman spectroscopy).

## Results

### Thiolutin as a key player in yeast aging

**Fig. 1:**
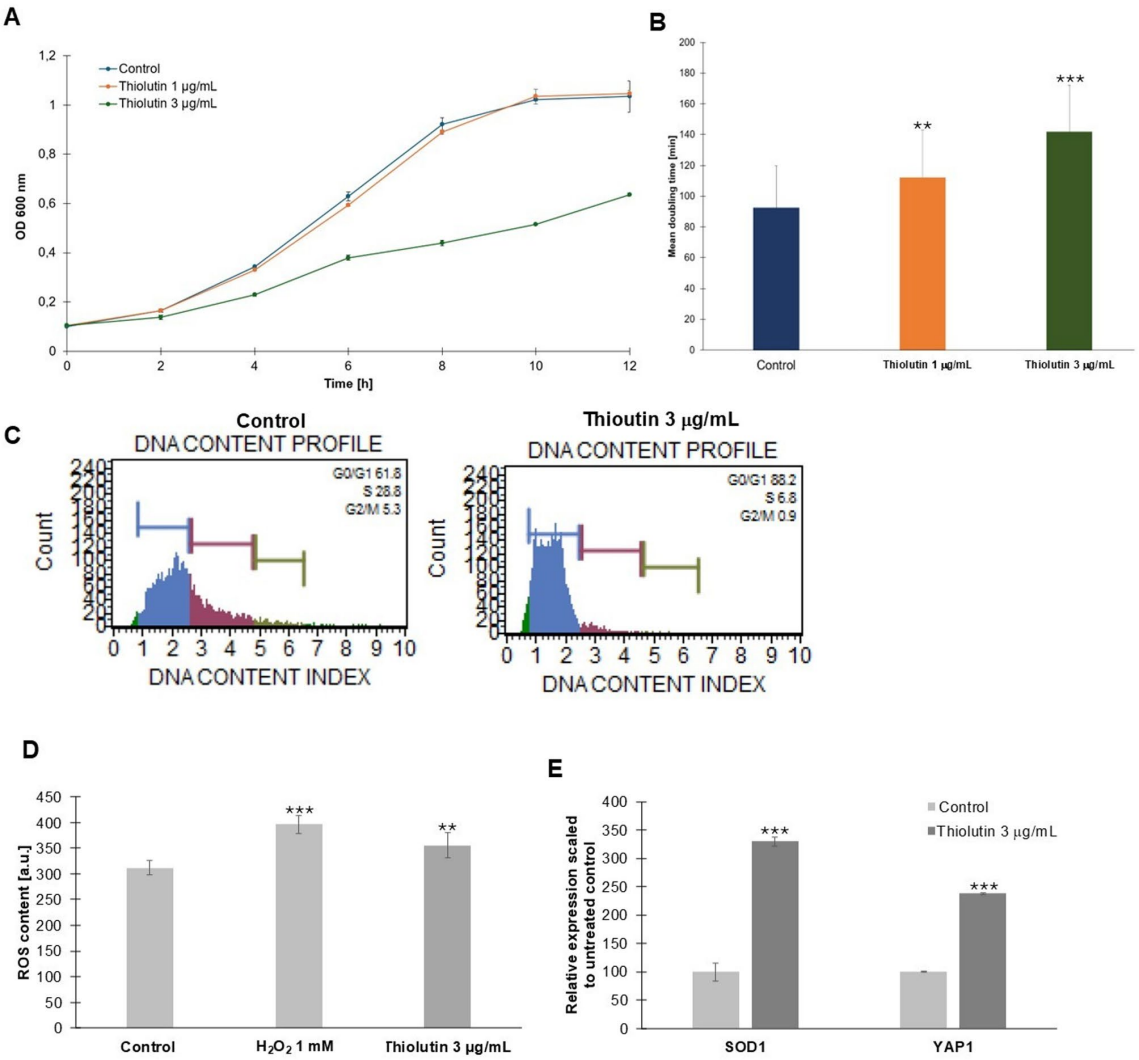
Thiolutin slows cell proliferation, induces G1 delay, and activates oxidative stress responses in yeast. **A** Growth kinetics of yeast cultures monitored as optical density at 600 nm (OD₆₀₀) over 12 h in untreated control cells and cells treated with thiolutin at 1 µg/mL or 3 µg/mL. Data represent mean ± SD from three independent biological replicates. **B** Mean doubling time of individual yeast mother cells under control conditions and following thiolutin treatment. Values represent mean ± SD. Statistical significance was assessed using one-way ANOVA with Dunnett’s post hoc test (**p < 0.01, ***p < 0.001 versus control).

Analysis of growth dynamics and cell-cycle progression revealed that thiolutin induces pronounced, dose-dependent alterations in *Saccharomyces cerevisiae* physiology. Growth kinetics (Fig. 1A) showed that thiolutin treatment slowed cell proliferation compared with untreated controls, with the strongest effect observed at 3 µg/mL. While control cells and those treated with 1 µg/mL thiolutin reached comparable optical densities at later time points, exposure to 3 µg/mL resulted in persistently reduced biomass accumulation throughout the experiment. Consistent with these observations, quantification of the mean doubling time (Fig. 1B) demonstrated a significant, dose-dependent prolongation of the cell cycle. Even low-dose thiolutin significantly increased doubling time, whereas treatment with 3 µg/mL produced a further and highly significant delay, indicating substantial restriction of proliferative capacity. Cell-cycle profiling based on DNA content (Fig. 1C) revealed that thiolutin-treated cells accumulated predominantly in the G0/G1 phase, accompanied by a marked reduction in the S-phase and G2/M fractions compared with control cells. These data indicate a delay at the G1/S transition, consistent with growth deceleration and reduced biosynthetic activity. Based on these phenotypic and cell-cycle alterations, we next performed biochemical and molecular analyses to assess whether thiolutin-induced growth limitation was accompanied by oxidative stress and activation of redox-responsive transcriptional programs. Measurement of intracellular reactive oxygen species (ROS) (Fig. 1D) showed a significant increase in thiolutin-treated cells relative to controls, although ROS levels remained lower than those observed under acute oxidative stress induced by H₂O₂. In parallel, transcriptional analysis revealed strong induction of the oxidative stress response genes *SOD1* and *YAP1* (Fig. 1E), indicating activation of antioxidant and redox-adaptive pathways. Our results demonstrate that thiolutin-induced slowing of growth and G1 delay are accompanied by moderate oxidative stress and a coordinated transcriptional response that supports redox homeostasis.

(C) Representative DNA content profiles obtained by flow cytometry showing cell-cycle distribution in control cells and cells treated with 3 µg/mL thiolutin. Percentages of cells in G0/G1, S, and G2/M phases are indicated. (D) Intracellular reactive oxygen species (ROS) levels measured in control cells, cells treated with 3 µg/mL thiolutin, and cells exposed to 1 mM H₂O₂ as a positive control for oxidative stress. Data are shown as mean ± SD (**p < 0.01, ***p < 0.001 versus control). (E) Relative expression of the oxidative stress-responsive genes *SOD1* and *YAP1* in cells treated with thiolutin (3 µg/mL), normalized to untreated control cells (set to 100%). Data represent mean ± SD from at least three independent biological replicates; ***p < 0.001 versus control.

**Fig. 2:**
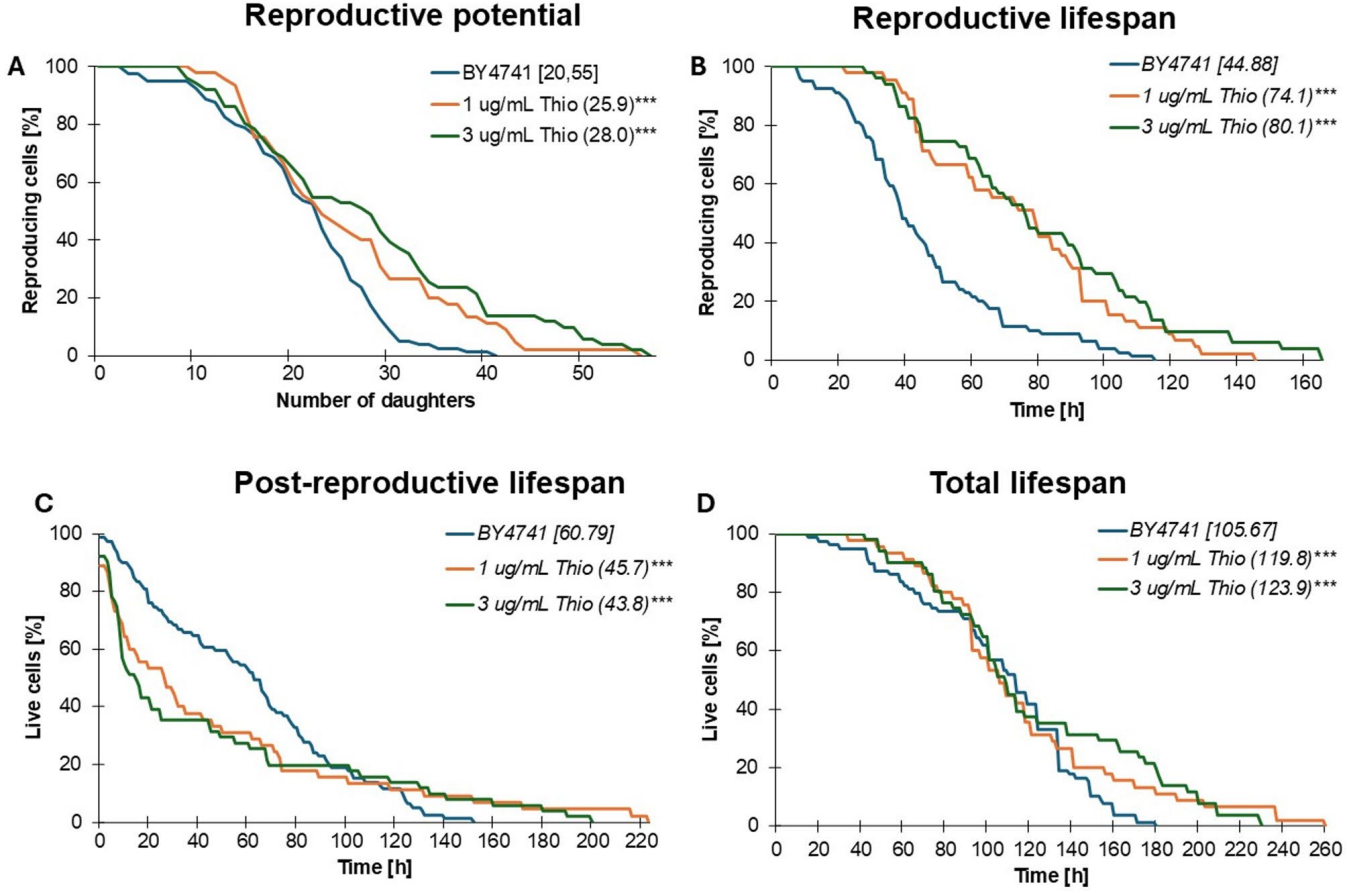
Analysis of lifespan parameters in haploid *S. cerevisiae* BY4741 (wild-type) cells, either untreated or exposed to thiolutin at concentrations of 1 µg/mL and 3 µg/mL. Panels represent: **A** reproductive potential, **B** reproductive lifespan, **C** post-reproductive lifespan, and **D** total lifespan. Statistical significance was evaluated using one-way ANOVA followed by Dunnett’s post hoc test (*p < 0.05, **p < 0.01, ***p < 0.001). Mean values, based on measurements from 90 cells across three independent experiments, are indicated in parentheses.

Our preliminary studies enabled the selection of concentrations for further analyses. We then investigated whether thiolutin treatment affects the aging of mitotically active and postmitotic cells. Replicative aging analyses, i.e., mitotically active cells, showed that cells treated with 1 and 3 µg/mL thiolutin exhibited increased reproductive potential and lifespan, which may be indicative of enhanced longevity. As shown in Fig. 2A, both concentrations of thiolutin significantly (*p* < 0.001) increased reproductive potential. It is important to highlight that the effect of thiolutin was crucial not only in increasing the average but also the maximum reproductive potential. The average number of daughter cells produced by a yeast mother cell increased by 25% and 28% when treated with 1 µg/mL and 3 µg/mL thiolutin, respectively. The consequence of the extended doubling time and the significant increase in reproductive potential is a statistically significant (*p* < 0.001) extension of the reproductive lifespan (Fig. 2B). We quantify reproductive lifespan from the initial cell budding to the onset of senescence. Thiolutin treatment was associated with reduced gene expression throughout the cell’s lifespan and a corresponding extension in doubling time. We then asked whether transcription inhibition could affect post-reproductive lifespan (Fig. 2C). Post-reproductive lifespan is the time from the last cell doubling until cell death. Interestingly, we found that cells treated with both 1 and 3 µg/mL thiolutin had a significantly shortened post-reproductive lifespan. This indicates that cells treated with thiolutin at both doses die significantly faster after the last doubling compared to untreated cells. Next, we posed the question of whether treating cells with a transcription inhibitor affects longevity. The total lifespan is the sum of the reproductive and post-reproductive lifespans. As shown in Fig. 2D, thiolutin-treated yeast cells exhibited extended lifespan, suggesting a potential beneficial effect on longevity. The cells showed a statistically significant extension of the average lifespan (*p* < 0.001) and also lived up to 50 h and 80 h longer for cells treated with 3 and 1 µg/mL thiolutin, respectively.

For the remaining experiments, we focused on a concentration of 3 µg/mL. First, we examined the aging rate of postmitotic cells. Postmitotic yeast cells, which have undergone their final mitosis and no longer budding, have specific requirements for proteins that differ from those of mitotic active cells. In postmitotic cells, key genes and proteins are involved in maintaining cellular functions, DNA repair, protection against oxidative stress, and regulation of metabolic processes. In our study, thiolutin has been shown to accelerate chronological aging in budding yeast. Studies have demonstrated that thiolutin treatment results in increased oxidative stress and damage to cellular components, which are key factors in the aging process^16^.

**Fig. 3:**
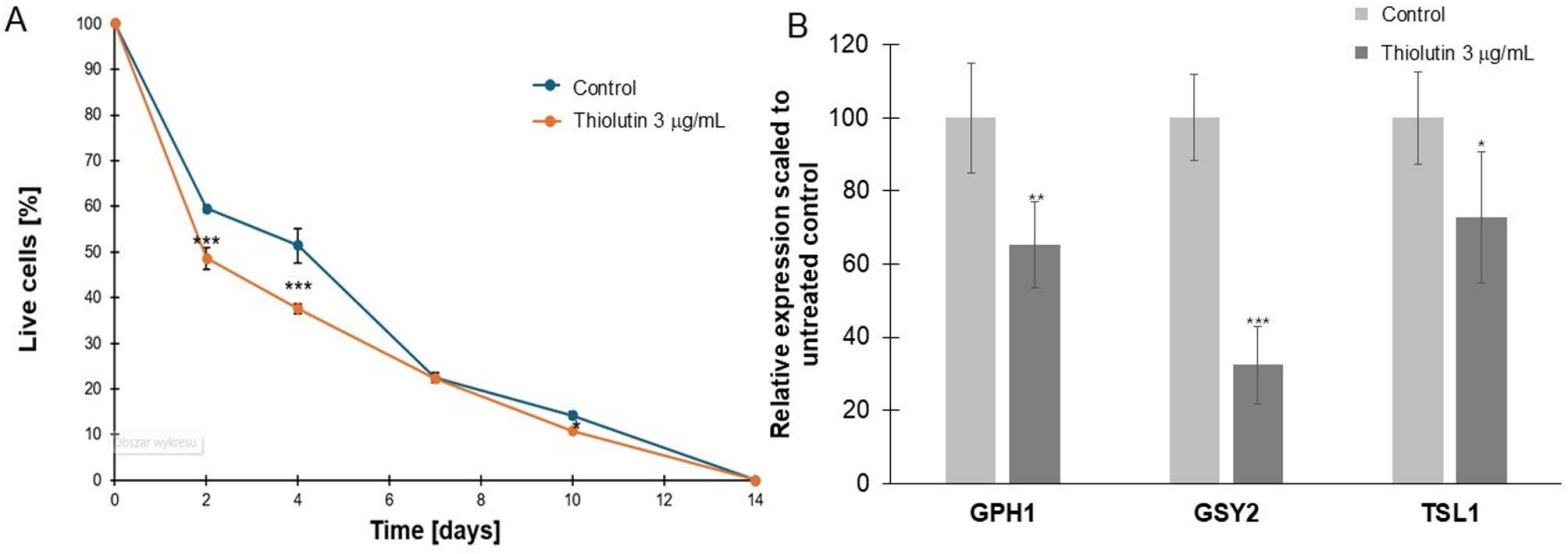
Thiolutin alters long-term cell survival and modulates expression of genes involved in carbohydrate storage. **A** Chronological survival of *Saccharomyces cerevisiae* cells cultured under control conditions or in the presence of thiolutin (3 µg/mL). The percentage of live cells was determined at the indicated time points and normalized to the initial population (day 0). Data are presented as mean ± SD. Asterisks indicate statistically significant differences between control and thiolutin-treated cells at the corresponding time points (**p < 0.01, ***p < 0.001). **B** Relative expression of genes associated with carbohydrate metabolism and storage (*GPH1*, *GSY2*, *TSL1*) in cells treated with 3 µg/mL thiolutin, normalized to untreated control cells. Expression values are shown as mean ± SD.

To further explore the metabolic basis of the early differences observed during chronological aging, we focused on genes associated with the biogenesis and protection of cellular storage compounds. *GPH1*, *GSY2*, and *TSL1* were selected for targeted expression analysis because they represent key and complementary nodes of storage carbohydrate metabolism that are known to be engaged at the onset of chronological aging and during early stationary phase adaptation. *GPH1* encodes glycogen phosphorylase, which mobilizes glycogen reserves and provides readily accessible energy during nutrient limitation^17^. *GSY2* encodes the major glycogen synthase active in stationary phase, supporting the accumulation of glycogen as an energy buffer under growth-restrictive conditions^18^. *TSL1* is a regulatory subunit of the trehalose-6-phosphate synthase complex and is required for efficient trehalose accumulation, a disaccharide that functions both as an energy reserve and as a stabilizing protectant of proteins and membranes during stress^19^. These storage carbohydrates play a dual role during early chronological aging: they provide metabolic flexibility under nutrient depletion and contribute to cellular stress tolerance. Accordingly, induction of *GPH1*,* GSY2*, and *TSL1* is commonly observed during the transition from exponential growth to stationary phase and has been linked to enhanced survival during the early stages of chronological aging. In thiolutin-treated cells, expression of *GPH1*, *GSY2*, and TSL1 was reduced relative to untreated controls (Fig. 3B), coinciding with decreased early chronological survival (Fig. 3A). This transcriptional pattern suggests that thiolutin may impair the establishment of a protective, storage-oriented metabolic state that normally supports cell viability at the onset of chronological aging. As illustrated in Fig. 3, a marked acceleration of aging was observed on both day 2 and day 4. Interestingly, from the 7th day of the experiment, the average survival values were similar, but cells treated with thiolutin exhibited a slightly faster tendency towards accelerated chronological aging.

### Thiolutin has a significant impact on the transcriptome and biochemical fingerprint

**Fig. 4:**
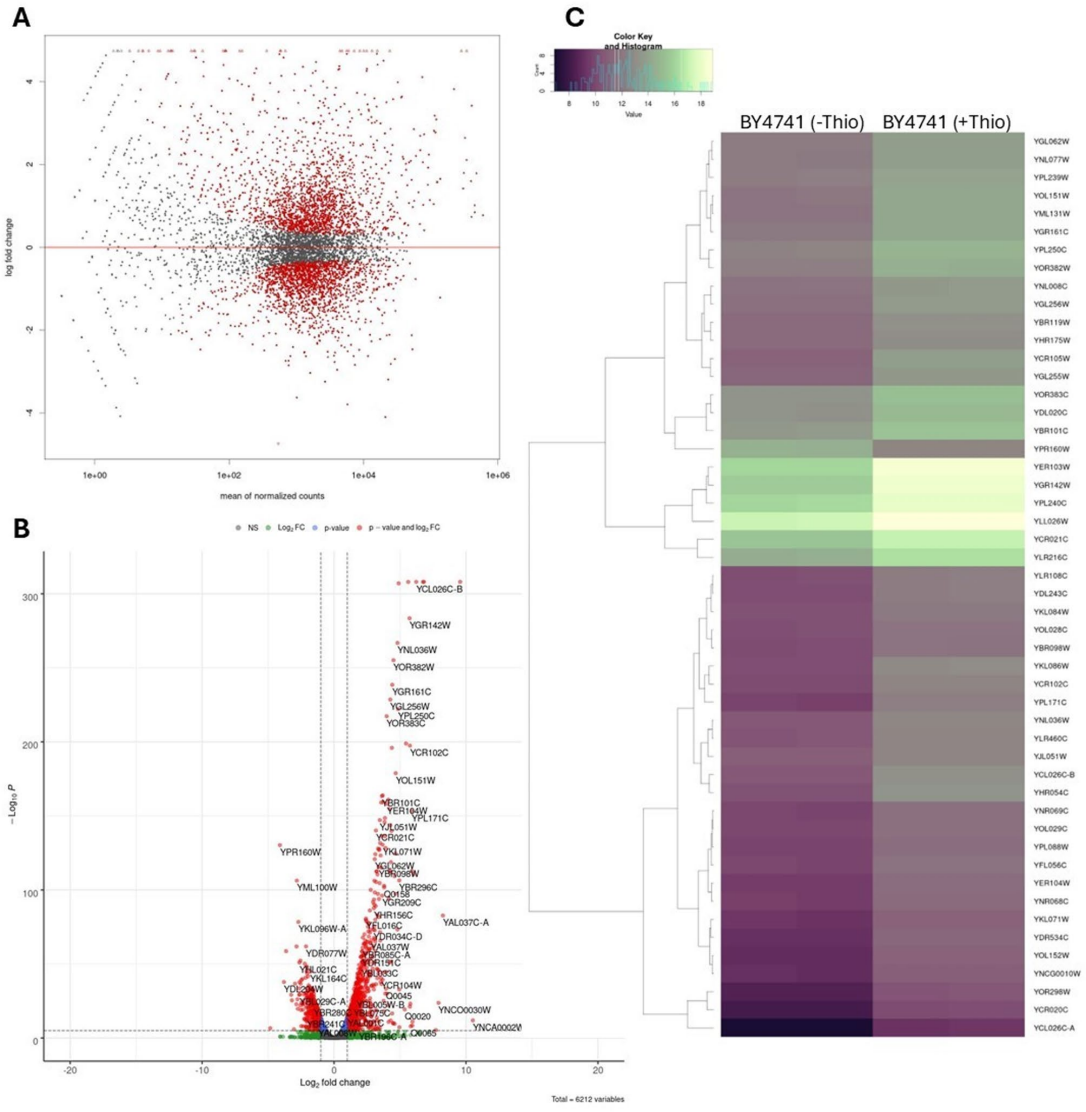
Identification of yeast cell transcripts treated and untreated with thiolutin. The relationship between logarithmic fold change and normalized averaged expression for a given gene is depicted, with each point on the graph representing the value for individual genes. Genes with an FDR < 0.05 are highlighted in red (**A**). Summary of the RNA-seq results. Volcano plot representation of differential expression analysis of genes in the untreated BY4741 versus treated with 3 µg/mL thiolutin for 3 h. Genes with FDR < 0.05 are marked with blue dots, genes with log2_Estimated_FoldChange> |1| are marked with green dots, while genes that meet both of the above conditions are marked with red dots. Non-statistically significant genes (not meeting the above-mentioned assumptions) are marked gray (**B**). Heatmap of differentially expressed genes in the untreated BY4741 (control) vs. treated with 3 µg/mL thiolutin for 3 h. Figure present expression value map in the analyzed samples for 50 genes with the lowest FDR_adjusted_p value coefficient.

To assess the cellular responses to thiolutin treatment, we conducted a comprehensive transcriptome analysis. Total RNA was extracted from both untreated wild-type cells and thiolutin-treated cells. The prepared libraries were then sequenced using next-generation sequencing techniques. Initially, we analyzed the relationship between logarithmic fold change and normalized averaged expression for each gene. As illustrated in Fig. 4A, thiolutin treatment resulted in extensive transcriptomic alterations, with both upregulation and downregulation of gene expression. These findings were corroborated by the numerical analyses presented in Fig. 4A, which demonstrated global changes by considering fluctuations in the expression of all genes as a frequency distribution. Of the 5 840 annotated protein-coding genes, 3 621 (62%) showed significant differential expression.

To visualize the extent and significance of gene expression changes, we generated a volcano plot (Fig. 4B), highlighting transcripts with the most pronounced differential expression following thiolutin treatment. Genes with significant changes are highlighted in different colors: blue for Log2_FC, green for p-value, and red for both p-value and Log2_FC. Non-significant genes are shown in gray. The plot also includes for specific genes that show significant changes.

As shown in Fig. 4C, the most significantly altered genes identified in the heatmap represent a diverse range of cellular localizations, biological processes, and molecular functions. Among the biological processes, notable changes were observed in genes involved in protein folding and stress response (*SSA4*,* HSP82*,* FES1*,* CPR6*,* BTN2*), oxidation-reduction processes (*ADH7*,* ADH4*,* GRE2*,* OYE3*,* AAD4*,* SRX1*), metal ion homeostasis and transport (*ZRT1*,* FRE7*,* FIT1*,* FIT2*,* FIT3*), transcriptional regulation and stress response (*RPN4*,* RTT105*), autophagy and vacuolar transport (*ATG41*,* BTN2*), and nitrogen compound metabolism (*HBN1*,* NCE103*).

In terms of molecular function, the most enriched categories included alcohol dehydrogenase activity (*ADH7*,* ADH4*,* AAD4*), chaperone binding and protein folding (*SSA4*,* HSP82*,* FES1*,* CPR6*), oxidoreductase activity (*GRE2*,* OYE3*,* SRX1*,* FRE7*), zinc ion transmembrane transporter activity (*ZRT1*), carbonic anhydrase activity (*NCE103*), and putative ubiquitin ligase activity (*ASI3*).

Differential transcriptome analysis revealed that thiolutin treatment led to statistically significant changes (*p* < 0.05) in the expression of 3,687 annotated genes. Among these, 3,621 corresponded to protein-coding mRNAs. In addition, the dataset included 11 transfer RNAs (tRNAs), 3 ribosomal RNAs (rRNAs), 5 non-coding RNAs (ncRNAs), 43 small nucleolar RNAs (snoRNAs), 2 small nuclear RNAs (snRNAs), 1 telomerase RNA, and 1 antisense RNA. These findings underscore the broad impact of thiolutin on both coding and non-coding elements of the yeast transcriptome (Data not shown). It is important to emphasize that RNA-Seq data represent steady-state RNA levels, which are determined by the dynamic balance between RNA synthesis (transcription) and RNA degradation. While our study focuses on the transcriptional effects of thiolutin, it is well established that thiolutin also interferes with mRNA degradation pathways^2^. Therefore, the observed transcriptomic changes in Fig. 4A, B may reflect not only transcriptional inhibition but also alterations in RNA turnover.

This distinction is particularly relevant because thiolutin has been shown to inhibit the exosome-dependent degradation of mRNA, potentially stabilizing certain transcripts while repressing others. As a result, the net expression levels captured by RNA-Seq may be influenced by both direct transcriptional repression and indirect effects on RNA stability. Although our data strongly support a global downregulation of gene expression consistent with transcriptional inhibition, we acknowledge that the contribution of thiolutin to the stabilization or destabilization of specific mRNA species cannot be excluded and may influence the interpretation of the results.

Differential gene expression analysis revealed a strong transcriptional response to thiolutin treatment (Table S1). Among the most significantly upregulated genes (FDR ≤ 0.05), we identified *ADH7* (log₂FC = + 6.81), *SRX1* (+ 6.74), *HBN1* (+ 6.23), and *SSA4* (+ 5.62), which are associated with oxidative stress response, redox homeostasis, and protein folding. Additionally, genes such as *BTN2* and *NCE103*—involved in vacuolar transport and carbon metabolism, respectively - were also strongly induced. Conversely, several genes were markedly downregulated, including *GPH1* (log₂FC = − 4.10), *TSL1*, *CWP2*, and *SED1*, which are linked to glycogen metabolism, cell wall integrity, and stress adaptation. These results indicate that thiolutin induces a broad and coordinated transcriptional reprogramming, affecting both metabolic and stress-related pathways.

Subsequently, we performed an in-depth analysis of the GO terms linked to biological processes, particularly those related to aging, cell death, transcriptional processes, and stress response (Table 2). In the category of biological processes, a total of 5764 genes were annotated. Among these, 3546 genes showed significant changes. Specifically, 1760 genes were upregulated, whereas 1786 genes were downregulated. As shown in Table 2, significant changes in gene expression were observed for terms involved in the regulation of cell aging, chronological cell aging, and the progressive alteration of chromatin associated with cell aging. These changes were also noted in terms related to the apoptotic process and cell death. Additionally, the regulation of the activity of all three RNA polymerases underwent key alterations. Interestingly, significant changes in gene expression were also identified for ribosome biogenesis, response to stress, and double-strand break repair. Significant changes were detected in several biological processes, including: generation of precursor metabolites and energy (GO:0006091), energy derivation by oxidation of organic compounds (GO:0015980), organonitrogen compound metabolic process (GO:1901564), phosphate-containing compound metabolic process (GO:0006796), phosphorus metabolic process (GO:0006793), oxidation-reduction process (GO:0055114), cellular process (GO:0009987), protein phosphorylation (GO:0006468), cellular metabolic process (GO:0044237), nucleoside triphosphate metabolic process (GO:0009141), glucose metabolic process (GO:0006006), and ATP metabolic process (GO:0046034) (Data not shown).

**Table 2:** Gene Ontology (GO) terms assigned to the differentially expressed genes – biological processes.

GO.ID	Term	Annotated	Significant	GO_upregulated	GO_downregulated
GO:0090342	Regulation of cell aging	11	11	7	4
GO:0001300	Chronological cell aging	32	26	8	18
GO:0001301	Progressive alteration of chromatin involved in cell aging	9	8	7	1
GO:0090344	Negative regulation of cell aging	5	5	5	0
GO:0007568	Aging	82	60	25	35
GO:1,900,062	Regulation of replicative cell aging	4	4	3	1
GO:0007569	Cell aging	80	58	24	34
GO:0001302	Replicative cell aging	49	31	14	17
GO:0006915	Apoptotic process	54	46	22	24
GO:0008219	Cell death	64	52	27	25
GO:0006356	Regulation of transcription by RNA polymerase I	40	30	18	12
GO:0006357	Regulation of transcription by RNA polymerase II	489	303	170	133
GO:0006359	Regulation of transcription by RNA polymerase III	18	14	6	8
GO:0043619	Regulation of transcription from RNA polymerase II promoter in response to oxidative stress	10	8	5	3
GO:0042254	Ribosome biogenesis	417	254	199	55
GO:0006950	Response to stress	886	585	298	287
GO:0006302	Double-strand break repair	145	78	39	39

**Fig. 5:**
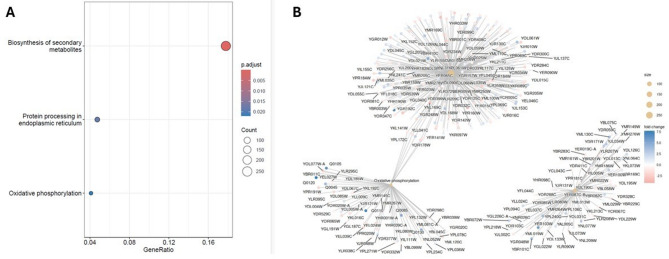
KEGG pathway enrichment analysis of differentially expressed genes (DEGs) identified by RNA-seq in *Saccharomyces cerevisiae* cells treated with thiolutin. **A** Dot plot summarizing the most significantly enriched KEGG pathways. Dot size corresponds to the number of DEGs mapped to each pathway, while dot color reflects the adjusted *p* value, with warmer colors indicating higher statistical significance.

To identify biological processes and signaling pathways affected by thiolutin at the transcriptomic level, differentially expressed genes obtained from RNA-seq analysis were subjected to KEGG pathway enrichment analysis. This approach revealed a highly coordinated and non-random distribution of DEGs across multiple core cellular pathways (Fig. 5). The most significantly enriched pathway was biosynthesis of secondary metabolites, characterized by the highest gene ratio and the largest number of associated DEGs. This enrichment reflects a broad transcriptional reorganization of metabolic networks, consistent with thiolutin-induced alterations in cellular redox balance, carbon flux, and anabolic–catabolic coordination. Such metabolic remodeling is frequently observed in conditions of growth inhibition and lifespan extension, suggesting that thiolutin promotes an adaptive metabolic state rather than a purely cytotoxic response. A second prominently enriched pathway was oxidative phosphorylation, indicating substantial transcriptional regulation of mitochondrial genes involved in electron transport and ATP production. The predominance of downregulated genes within this pathway is consistent with reduced respiratory activity and intracellular ATP levels observed in functional assays, supporting the notion that thiolutin attenuates mitochondrial energy output. This mitochondrial reprogramming aligns with established longevity paradigms, in which partial suppression of oxidative phosphorylation can trigger compensatory stress responses and promote replicative lifespan extension. Additionally, protein processing in the endoplasmic reticulum emerged as a significantly enriched pathway, implicating thiolutin in the modulation of proteostasis networks. Differential expression of genes involved in protein folding, quality control, and ER-associated degradation suggests increased proteotoxic stress or an adaptive reinforcement of protein quality control mechanisms. This finding is consistent with thiolutin’s known inhibitory effects on transcription and its capacity to perturb nascent protein synthesis, thereby placing additional demands on the ER folding machinery. Network-based visualization of gene–pathway associations (Fig. 5B) further revealed that individual DEGs frequently map to multiple pathways, underscoring the interconnected nature of thiolutin-induced responses. Notably, mitochondrial, metabolic, and proteostasis-related pathways formed partially overlapping subnetworks, highlighting a coordinated transcriptional program that integrates energy metabolism, stress adaptation, and protein homeostasis. Collectively, KEGG pathway analysis demonstrates that thiolutin elicits a systems-level transcriptional response encompassing metabolic rewiring, mitochondrial downscaling, and enhanced proteostasis control. These pathway-level changes provide a mechanistic framework linking thiolutin-induced transcriptional inhibition to altered cellular physiology and replicative lifespan extension in budding yeast.

(B) Gene–pathway network visualization illustrating the relationships between individual DEGs and enriched KEGG pathways. Nodes represent genes or pathways, with pathway nodes labeled accordingly. Gene node color indicates log₂ fold change in expression upon thiolutin treatment (red, upregulated; blue, downregulated), and node size reflects the number of gene–pathway connections. The network highlights prominent regulation of oxidative phosphorylation, protein processing in the endoplasmic reticulum, and biosynthesis of secondary metabolites, revealing a broad transcriptional response affecting mitochondrial energy metabolism, proteostasis, and metabolic adaptation.

**Fig. 6:**
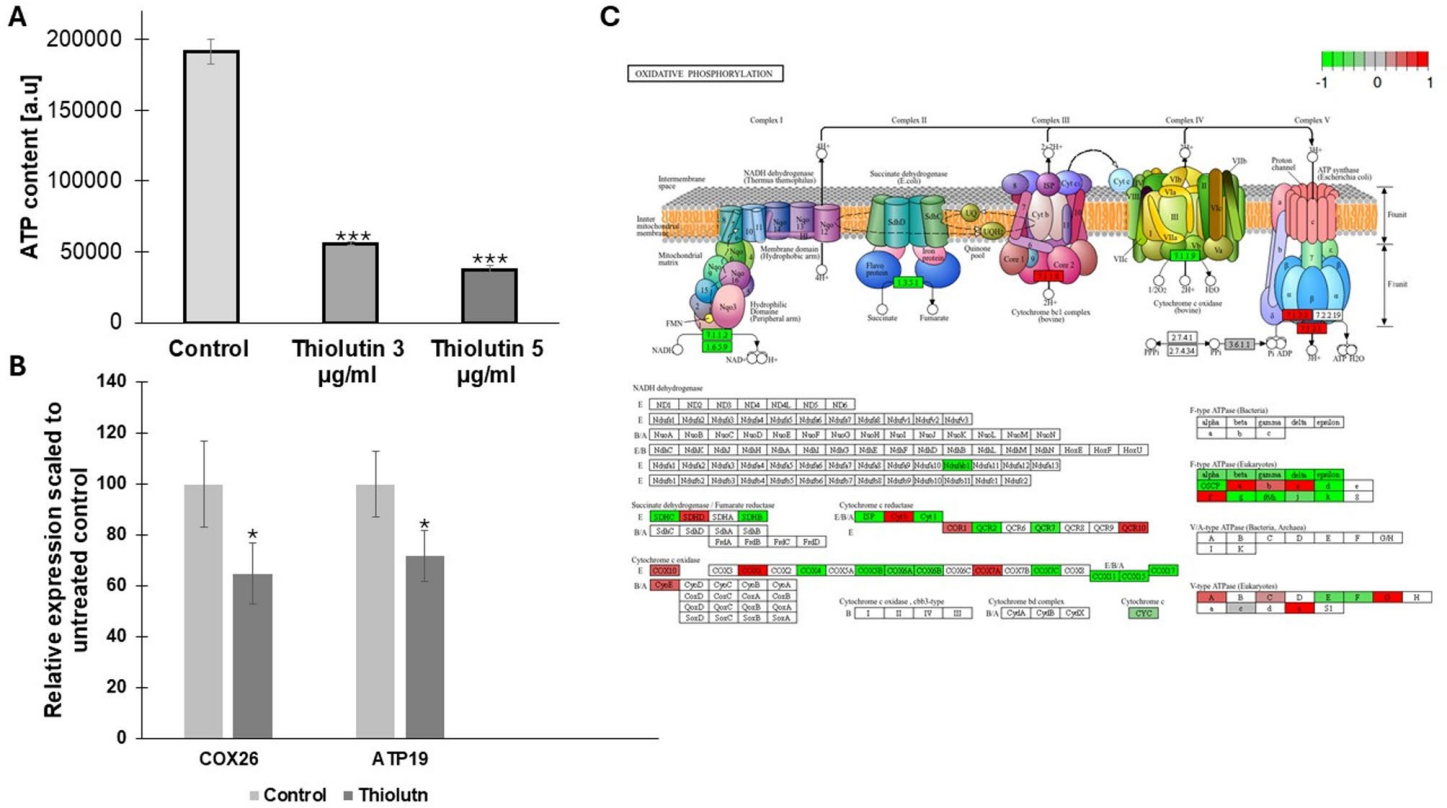
Thiolutin suppresses mitochondrial ATP production and remodels oxidative phosphorylation at the transcriptional level. **A** Quantification of intracellular ATP content in yeast cells treated with 3 and 5 µg/mL thiolutin compared with untreated control cells. Data are expressed as arbitrary units (a.u.) and presented as mean ± SD. Asterisks indicate statistically significant differences between control and thiolutin-treated cells at the corresponding time points (***p < 0.001). **B** Relative expression of mitochondrial oxidative phosphorylation–related genes *COX26* and *ATP19* following 3 µg/mL thiolutin treatment, normalized to untreated control cells. Asterisks indicate statistically significant differences between control and thiolutin-treated cells at the corresponding time points (*p < 0.05). **C** KEGG oxidative phosphorylation pathway map overlaid with RNA-seq expression data from thiolutin-treated cells^22–25^. Color coding reflects log₂ fold changes in gene expression (green, downregulation; red, upregulation). Thiolutin induces coordinated downregulation of multiple components of the electron transport chain and ATP synthase, consistent with impaired mitochondrial energy metabolism.

To determine whether thiolutin-induced transcriptional changes translate into functional alterations in cellular energy metabolism, intracellular ATP levels and expression of key oxidative phosphorylation genes were analyzed. Thiolutin treatment resulted in a marked and dose-dependent decrease in intracellular ATP content, with cells exposed to 3 µg/mL and 5 µg/mL thiolutin exhibiting a substantial reduction compared with untreated controls (Fig. 6A). These results indicate a strong impairment of cellular energy production upon thiolutin exposure. Consistent with these functional data, transcriptional analysis revealed reduced expression of genes encoding core mitochondrial components of the oxidative phosphorylation machinery. Specifically, *COX26*, a structural subunit of cytochrome c oxidase (complex IV)^20^, and *ATP19*, a component of the F₀F₁-ATP synthase (complex V)^21^, were both significantly downregulated following thiolutin treatment (Fig. 6B). These changes suggest attenuation of electron transport chain activity and ATP synthesis at the transcriptional level. Integration of RNA-seq data with the KEGG oxidative phosphorylation pathway further demonstrated that thiolutin elicits a coordinated remodeling of mitochondrial energy metabolism (Fig. 6C). Multiple genes encoding subunits of respiratory complexes and ATP synthase showed reduced expression, indicating a global suppression of oxidative phosphorylation rather than isolated effects on individual components. Importantly, this transcriptional signature aligns with the observed depletion of cellular ATP and supports the conclusion that thiolutin compromises mitochondrial energy output. Taken together, these findings demonstrate that thiolutin suppresses oxidative phosphorylation through combined transcriptional downregulation of mitochondrial respiratory genes and functional inhibition of ATP production. This mitochondrial energy limitation likely contributes to the reduced growth rate, altered cell cycle progression, and longevity-associated phenotypes observed in thiolutin-treated yeast cells.

**Fig. 7:**
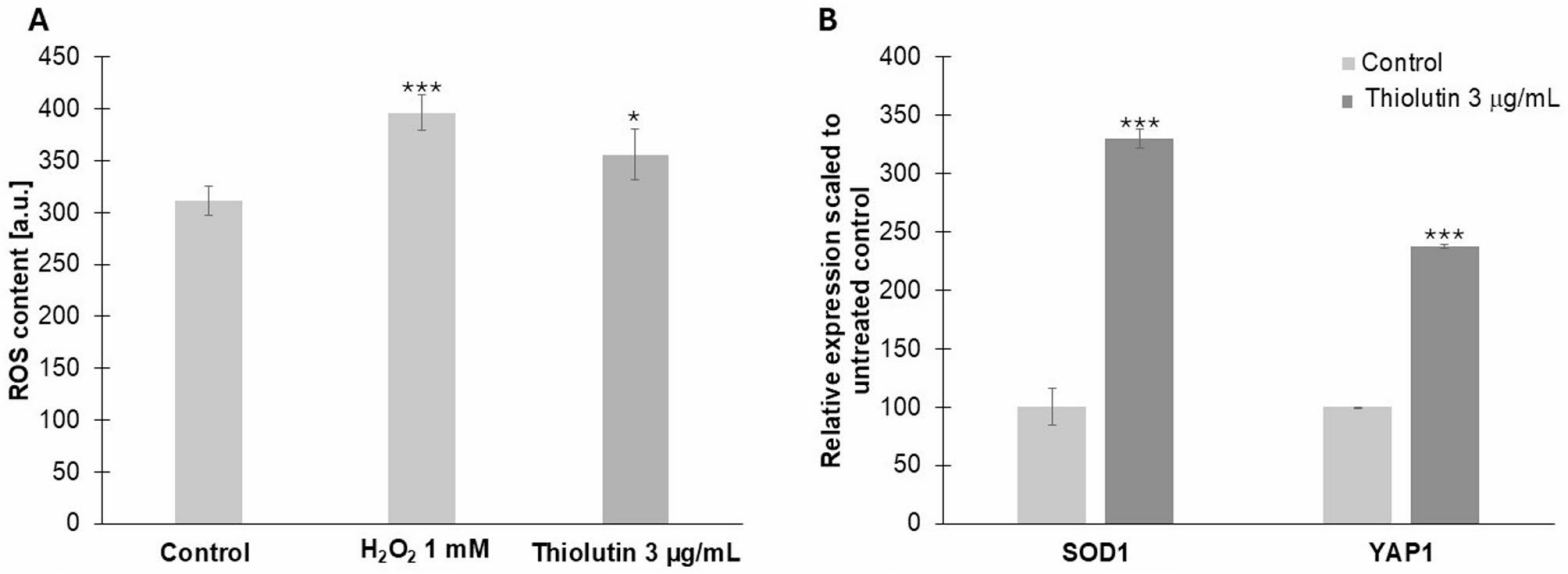
Thiolutin induces oxidative stress and activates redox-responsive gene expression. **A** Intracellular reactive oxygen species (ROS) levels measured in *Saccharomyces cerevisiae* under control conditions and following treatment with 3 µg/mL thiolutin. 1 mM hydrogen peroxide is shown as a reference oxidative stress condition to illustrate the dynamic range of the ROS assay. Data are presented as mean ± SD and expressed as arbitrary units (a.u.). **B** Relative expression of the oxidative stress–responsive genes *SOD1* and *YAP1* in cells treated with 3 µg/mL thiolutin, normalized to untreated control cells. Gene expression values are shown as mean ± SD. Asterisks indicate statistically significant differences between control and thiolutin-treated cells at the corresponding time points (*p < 0.05; ***p < 0.001).

To assess whether thiolutin affects cellular redox homeostasis, intracellular ROS levels were quantified. Treatment with 3 µg/mL thiolutin resulted in a clear increase in ROS relative to untreated control cells (Fig. 7A). 1 mM hydrogen peroxide was included as a reference oxidative stress condition to demonstrate assay responsiveness, without implying direct mechanistic equivalence between the two treatments. Consistent with increased oxidative burden, thiolutin treatment was accompanied by elevated expression of redox-responsive genes. Transcript levels of *YAP1*, a central regulator of oxidative stress responses, and *SOD1*, encoding a major antioxidant enzyme, were significantly increased relative to control cells (Fig. 7B). These data indicate that thiolutin elicits a measurable oxidative stress response and activates established redox-sensitive transcriptional markers. Importantly, this analysis does not assume that thiolutin acts through the same mechanisms as exogenous oxidants, but rather demonstrates that thiolutin exposure is sufficient to perturb redox balance and engage endogenous antioxidant response pathways.

**Fig. 8:**
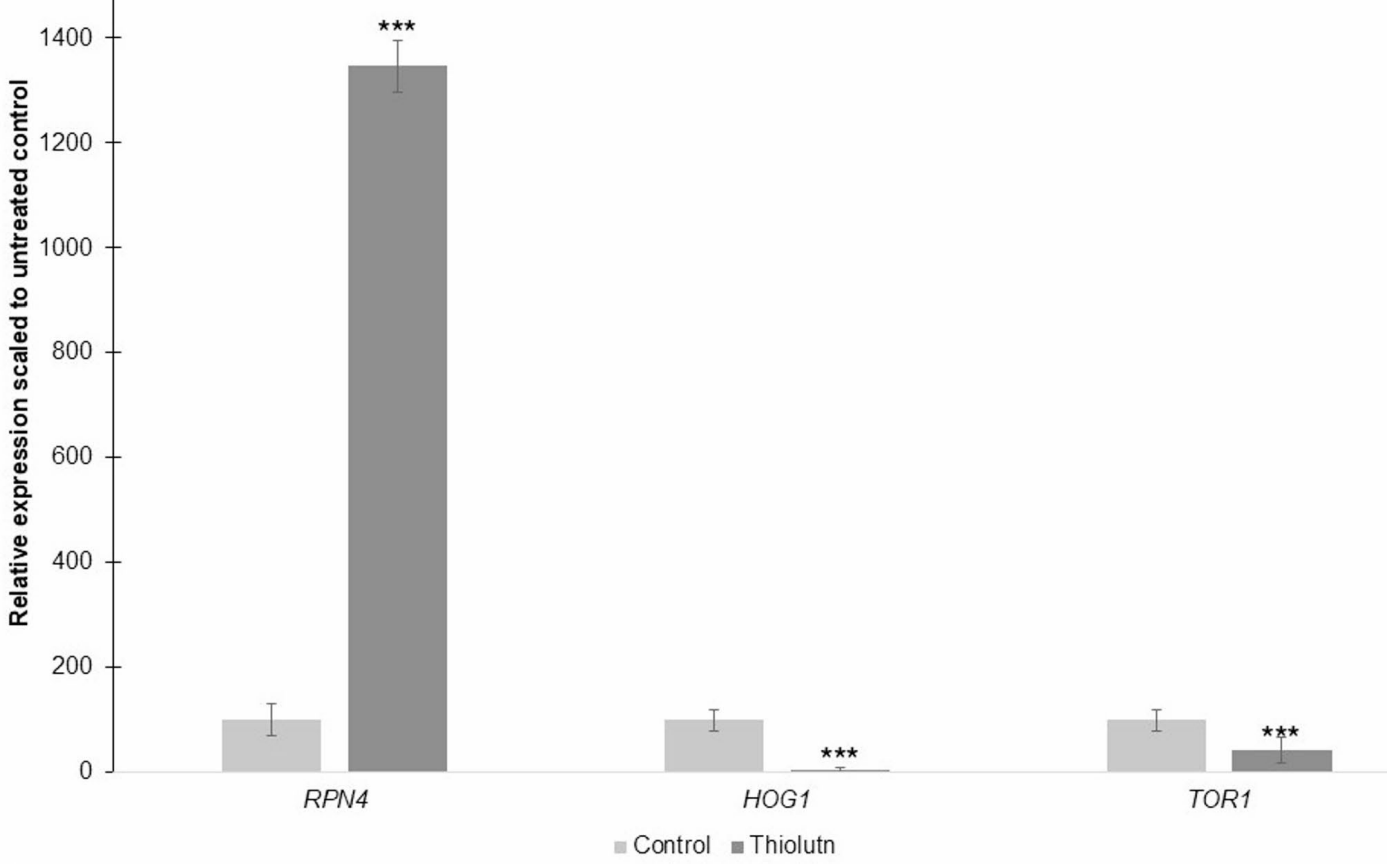
Thiolutin selectively modulates proteostasis and growth-related regulatory pathways. Relative expression of regulatory genes *RPN4*, *HOG1*, and *TOR1* in *S. cerevisiae* cells treated with 3 µg/mL thiolutin (), normalized to untreated control cells (set to 100%). Data are presented as mean ± SD.

To further characterize regulatory pathways engaged by thiolutin, we examined the expression of selected genes representing proteostasis control (*RPN4*)^26^, stress-activated MAPK signaling (*HOG1*)^27^, and nutrient- and growth-sensing regulation (*TOR1*)^28^ (Fig. 8). These genes were chosen to assess whether thiolutin elicits a generalized stress response or a more selective adaptive program. Thiolutin treatment resulted in a pronounced induction of *RPN4*, a transcription factor that coordinates expression of proteasome subunits and is activated under conditions of increased proteotoxic burden. This strong upregulation suggests that thiolutin imposes a substantial challenge to protein homeostasis, necessitating reinforcement of proteasome-mediated protein quality control. This observation is consistent with transcriptomic pathway enrichment analyses indicating activation of proteostasis-related processes. In contrast, expression of *HOG1*, a central MAP kinase involved in osmotic and environmental stress responses, was not induced and instead showed reduced transcript levels following thiolutin exposure. This finding indicates that thiolutin does not activate the canonical HOG-dependent stress response pathway, supporting the notion that the cellular response to thiolutin differs from classical environmental stress signaling. Expression of *TOR1*, a key regulator of growth and nutrient-responsive signaling, was also reduced in thiolutin-treated cells. Downregulation of *TOR1* is consistent with growth inhibition and metabolic reprogramming observed under thiolutin treatment and aligns with broader transcriptional and physiological changes associated with reduced anabolic activity. Together, these results demonstrate that thiolutin elicits a selective transcriptional response characterized by strong activation of proteostasis mechanisms and attenuation of growth-related regulatory pathways, rather than a generalized activation of canonical stress signaling. This regulatory profile supports a model in which thiolutin drives an adaptive cellular state shaped by impaired protein homeostasis and restricted growth capacity.

Significant changes in gene expression levels exceeding 60% must result in biochemical alterations within the cells. To investigate the biochemical fingerprint, we employed Raman spectroscopy. Raman spectroscopy is a powerful technique that provides detailed information about the chemical structure, polymorphism and molecular dynamics of a sample. This method allows us to detect and characterize the biochemical changes induced by the altered gene expression. FT-Raman spectroscopy enables the identification and determination of the chemical composition of the analyzed material. Both primary metabolites (mono- and oligosaccharides, fatty acids, amino acids, proteins) and secondary metabolites (alkaloids, flavonoids, terpenes, polyacetylenes) can be analyzed. Raman spectroscopy is a vibrational spectroscopic technique that enables the non-destructive analysis of molecular composition based on inelastic scattering of monochromatic light, typically from a laser source. When incident photons interact with molecular bonds, a small fraction undergoes energy shifts corresponding to specific vibrational modes, producing a spectrum that serves as a molecular fingerprint of the sample. This method is particularly well-suited for biological applications, as it allows for the detection and characterization of a wide range of biomolecules - including proteins, nucleic acids, lipids, and carbohydrates - without the need for labeling or extensive sample preparation. In the context of cellular studies, Raman spectroscopy provides valuable insights into biochemical alterations associated with metabolic changes, stress responses, or pharmacological interventions. In this study, the technique was employed to assess the impact of thiolutin on the biochemical profile of yeast cells, enabling the identification of molecular changes that reflect transcriptional and metabolic reprogramming. The 8 cm^− 1^ resolution and cellular complexity cause significant band overlap and individual peaks cannot unambiguously distinguish closely related structures. Therefore, FT‑Raman data cannot distinguish which specific proteins, lipids, or nucleic acid species are changing, and conclusions are limited to relative changes in band regions and overall biochemical composition between conditions.

**Fig. 9:**
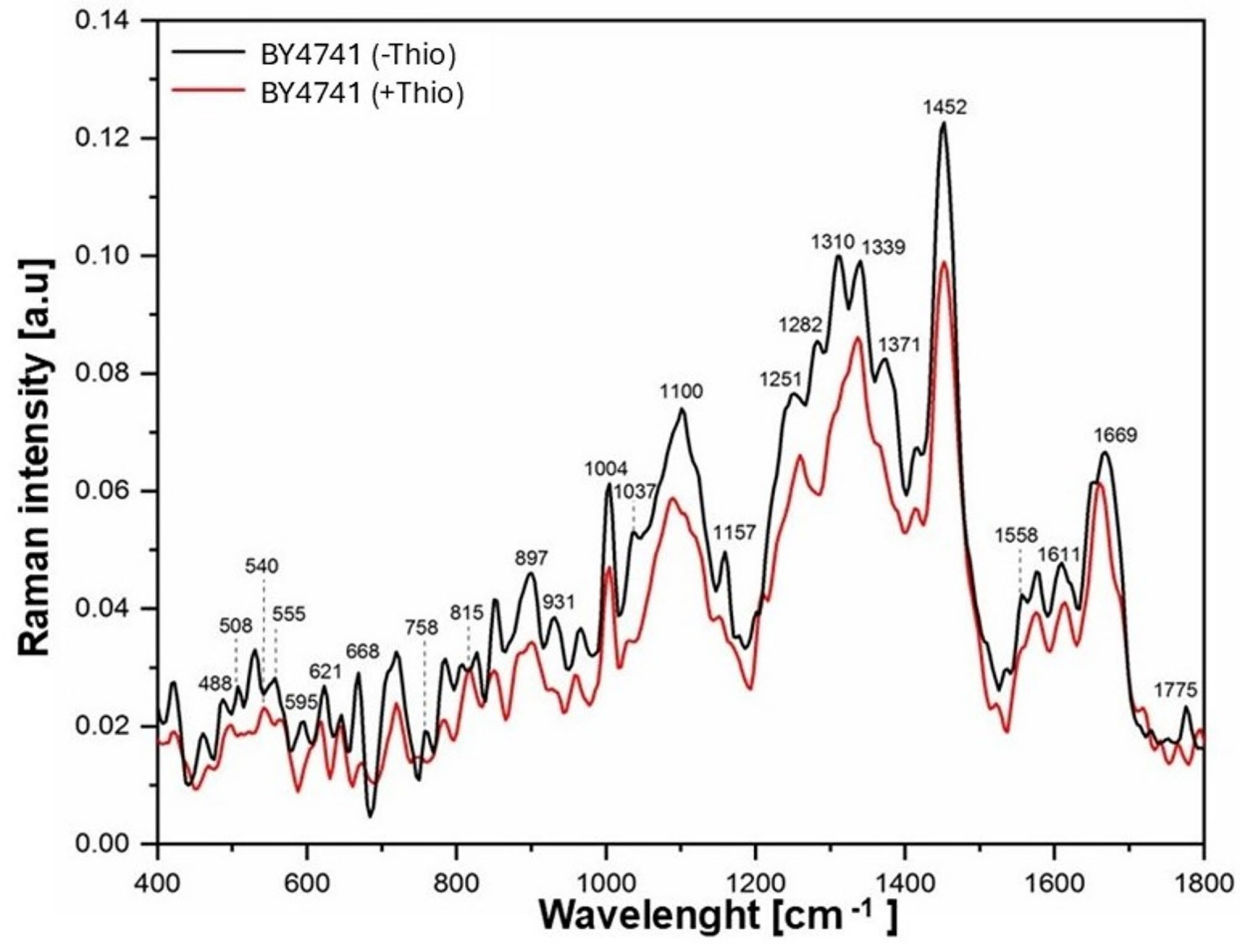
The Raman spectra of the yeast BY4741, untreated and treated with 3 µg/mL thiolutin. The spectra report global biochemical fingerprints and that band assignments are approximate, based on typical ranges for proteins, lipids, nucleic acids and carbohydrates in yeast and other cells. The experiments were conducted in six independent biological replicates.

FT-Raman spectra recorded in the range of 400 to 1800 cm⁻¹ for the BY4741 strain untreated with thiolutin and after the addition of thiolutin are presented in Fig. 9.

Changes in chemical composition are identified based on bands typically associated with particular functional groups or biomolecular classes (Table 3). The spectrum shows varying band intensities, indicating different compound contents or peak shifts, suggesting changes within the molecular bonds of the compound, thus indicating a reshaped cellular biochemical composition or modifications within the underlying molecular bonds. These regions are typically dominated by contributions from proteins/DNA/fatty acids in yeast, although each band may contain overlapping contributions from multiple components. The absence of a clearly discernible peak in a given region suggests a low content of the corresponding compound class, within the detection limits and considering possible band overlap.

**Table 3:** Summary of Raman spectrum peak positions where characteristic peaks for individual chemical compounds occur.

BY4741 (- Thio)	BY4741 (+ Thio)	Characteristic vibrations of functional groups
Peak positions [Raman shift cm⁻¹]		
1775	–	Fatty acids^29^
1669	1658	Fatty acids, proteins^29–32^
1611	1611	Mitochondria, proteins^31,33^
1574	1574	RNA/DNA^34,35^
1558	–	Amide II^35^
1452	1452	Proteins, fatty acids^31,32^
1415	1415	Fatty acids^29^
1371	–	RNA/DNA^34,35^
1339	1337	RNA^34,36,37^
1310	–	Fatty acids^35^
1282	–	Proteins^35^
1251	1258	Fatty acids^29^
1157	–	Proteins^36^
1100	1086	Polysaccharides, RNA/DNA^31,32,36^
1037	–	Proteins^35^
1004	1004	Proteins, RNA^29,32,33^
964	958	Polysaccharides^30,36^
931	–	DNA^36^
897	898	Polysaccharides^30,32^
853	853	Proteins^31–33^
825	–	RNA, proteins^35^
805	815	RNA^36^
782	782	RNA/DNA^34,35^
758	–	Proteins^31,35^
719	719	RNA^34,36,37^
668	672	RNA/DNA^34–36^
644	644	Proteins^36^
621	615	RNA/DNA^34–36^
595	–	Phospholipids^35^
555	565	RNA/DNA^34–36^
530	540	RNA, Polysaccharides^36,37^
508	–	Proteins^36^
488	498	Polysaccharides^35^
462	465	Polysaccharides^30,32^
422	422	

*Assignments in Table 3*are approximate and refer to predominant functional groups or biomolecular classes; individual bands may contain contributions from multiple species and should not be interpreted as unique structural markers.*

Spectral analysis revealed that in yeast with thiolutin (+ Thio), bands typically associated with proteins (508, 758, 825, 1037, 1282, 1371 cm^−1^), DNA (931 cm^−1^), and fatty acids (1310, 1775 cm⁻¹) were not clearly discernible compared to yeast without thiolutin (− Thio), suggesting a reduced contribution from these biomolecular classes within the detection limits and considering possible band overlap. Additionally, significant band shifts were observed in the spectrum of yeast with thiolutin compared to the spectrum of yeast without thiolutin. These bands fall in regions typically associated with polysaccharides (498 cm^−1^), RNA and DNA (540, 565, 615, 672, 815, 1086 cm^− 1^), and proteins and lipids (958, 1258, 1658 cm^− 1^). These spectral shifts and amplitude changes indicate that thiolutin reshapes cellular biochemical composition, consistent with transcriptome-level reprogramming.

**Fig. 10:**
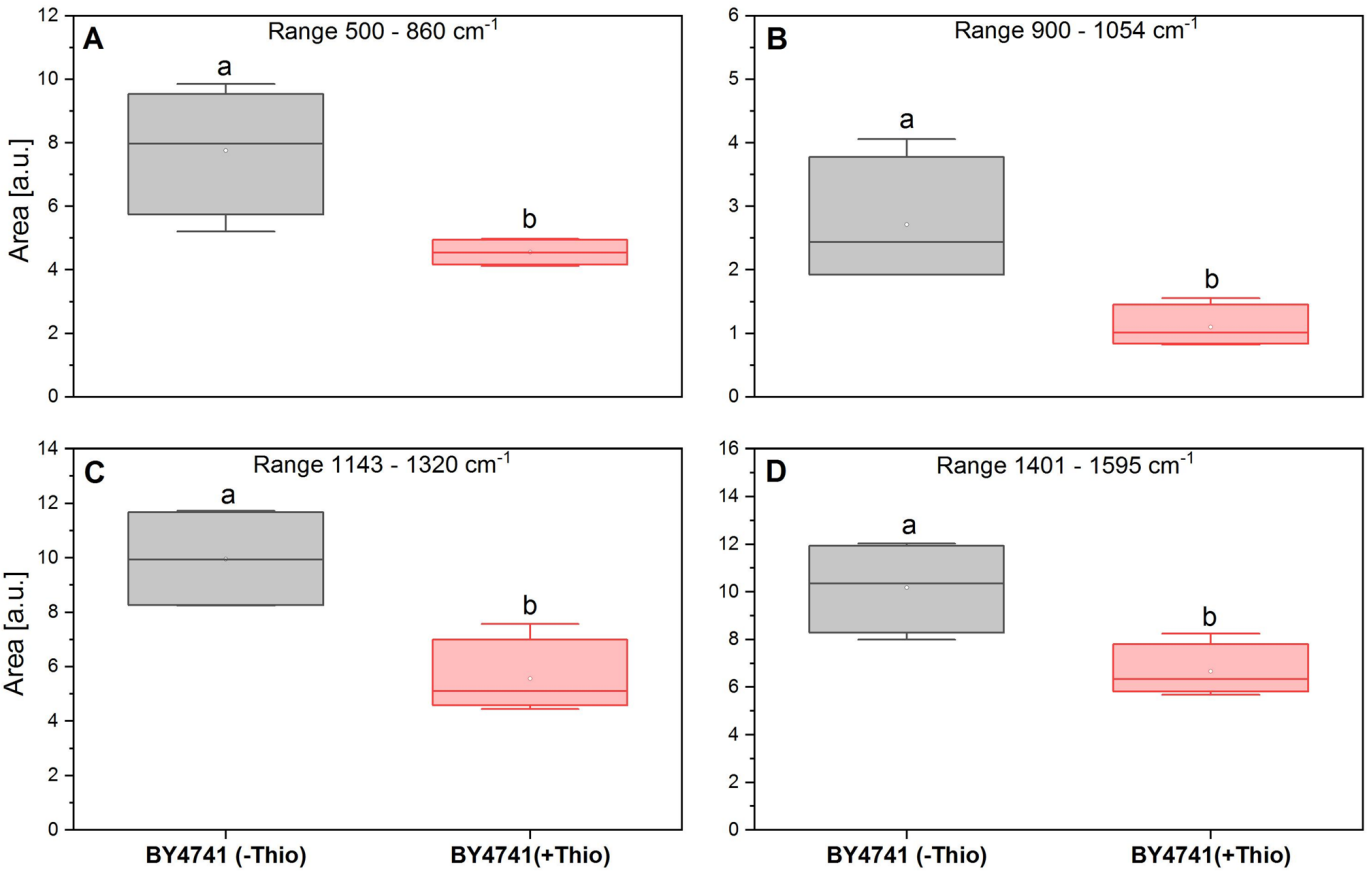
The comparison of the FT-Raman spectra area (BY4741 untreated - gray box, and BY4741 thiolutin treated - red box) only into the range where bands characteristic of RNA and proteins occur. The spectrum range: **A** – 500–860 cm^− 1^, **B** – 900–1054 cm^− 1^, **C** – 1143–1320 cm^− 1^, **D** – 1401–1595 cm^− 1^. The values represent means ± SD (*n* = 6). The statistical significance between objects was determined based on Tukey’s post-hoc test.

We also compared the area under the curve for the spectra of cells treated and untreated with thiolutin. As shown in Fig. 10, it is clearly demonstrated that thiolutin reshapes the cellular biochemical composition, consistent with the transcriptome-level reprogramming observed by RNA-Seq.

## Discussion

Thiolutin is a well-established transcriptional inhibitor commonly used in studies of mRNA stability. However, recent studies have raised important concerns regarding its broader application in probing other cellular processes, due to its pleiotropic effects and multifunctional nature^1,38^. Although thiolutin inhibits transcription, its relative impact on initiation versus elongation in vivo remains incompletely defined, and some studies have suggested that RNA polymerase II inhibition in vivo may occur indirectly^39,40^. Qiu et al. provided in vitro evidence that thiolutin can directly inhibit RNA polymerase II transcription initiation, and genetic analyses further indicated that thiolutin induces oxidative stress in vivo, likely through oxidation of thioredoxins, linking transcriptional inhibition to redox regulation^1^. Thiolutin can promote nuclear translocation of Yap1 and deplete both reduced and oxidized glutathione pools, generating a phenotype that differs from classical oxidants such as hydrogen peroxide or menadione^1,41^. Interestingly, earlier studies did not report an increase in mutation frequency, as assessed by canavanine resistance assays^1^. Our results reveal, for the first time, a functional link between thiolutin and the aging process in *S. cerevisiae*. Yeast aging is commonly assessed using two complementary paradigms: replicative aging, defined by the number of daughter cells produced by a single mother cell before senescence, and chronological aging, defined by the survival of non-budding cells in stationary phase^9,42–44^. Our study clearly demonstrates that thiolutin treatment enhances the reproductive potential of yeast cells at both tested concentrations. Notably, we observed a significant reduction in the rate of cell growth, measured both through growth kinetics and mean doubling time, which resulted in an extended overall reproductive period. Interestingly, despite this slower growth, post-reproductive aging was markedly accelerated in thiolutin-treated cells compared to untreated controls. However, the total lifespan of thiolutin-treated cells was longer than that of the control group, indicating that reduced transcriptional activity contributes to a beneficial cellular phenotype. A similar longevity phenotype was previously observed in our studies of the *sfp1Δ* mutant, as well as in mutants involved in the biogenesis of the 60 S ribosomal subunit^11,45,46^. In this study, we also demonstrated that thiolutin significantly alters the expression of over 60% of annotated genes. Notably, these transcriptomic changes were corroborated by biochemical evidence. Using the innovative FT-Raman spectroscopy technique, we observed a substantial reduction in the levels of all major cellular macromolecules compared to untreated controls. These findings not only confirm a general slowdown in cellular growth but, more importantly, highlight widespread transcriptional dysregulation likely resulting from RNA polymerase inhibition. Cellular exposure to environmental stress induces adaptive modifications in gene expression, with alterations in mRNA stability playing a pivotal role in this response^47^. While stress-activated signaling pathways are predominantly investigated for their influence on transcriptional and translational regulation, accumulating evidence indicates that several of these pathways contribute significantly to the modulation of mRNA decay in yeast and other organisms^48^. Recent findings indicate that thiolutin acts as an inhibitor of TORC1 signaling, a central regulator of cell growth, metabolism, and stress responses in yeast. In our study, this is consistent with the observed transcriptomic and phenotypic changes. Specifically, RNA-Seq data revealed significant downregulation of genes involved in ribosome biogenesis and energy metabolism - both of which are tightly regulated by TOR1. Moreover, the reduced intracellular ATP levels and slowed growth rate in thiolutin-treated cells further support TOR1 pathway suppression. These results suggest that thiolutin-induced TOR1 inhibition contributes to the observed extension of replicative lifespan and metabolic reprogramming, highlighting a multifactorial mechanism of action that integrates transcriptional repression with nutrient-sensing and stress-adaptive pathways^48,49^. Our findings suggest that the cellular effects of thiolutin cannot be solely attributed to transcriptional inhibition. Instead, the observed phenotypes—such as altered growth dynamics, changes in lifespan, and metabolic shifts—are likely the result of a multifactorial response. Transcriptomic and biochemical analyses revealed significant alterations in pathways associated with cellular stress and signaling. In particular, we observed gene expression changes consistent with the activation or modulation of the TOR, HOG, and oxidative stress response pathways. These pathways are known to play central roles in regulating cell growth, metabolism, and aging in yeast. Therefore, we propose that thiolutin exerts its effects through a complex network of interactions, and its impact on cellular physiology should be interpreted in the context of these broader regulatory mechanisms. Based on RNA-Seq data, we infer that the cellular response to thiolutin exposure is dynamic and multifactorial. The observed transcriptomic changes—affecting over 60% of annotated genes—suggest both direct transcriptional inhibition and compensatory activation of stress-responsive pathways, including those involved in ribosome biogenesis, oxidative stress, and transcriptional regulation. Importantly, the downregulation of genes involved in ribosome biogenesis and translation aligns with the observed extension of replicative lifespan. This phenotype is consistent with previous studies showing that reduced protein synthesis can promote longevity in yeast. The repression of ribosomal genes, coupled with decreased ATP levels, suggests that thiolutin induces a low-energy, stress-resistant state that favors survival over proliferation.

RT–qPCR analysis revealed downregulation of the reserve carbohydrate–related genes *GSY2*, *GPH1*, and *TSL1*. These transcriptional changes were fully consistent with Raman spectroscopy–based profiling, which showed a concurrent decrease in carbohydrate-related spectral features, as evidenced by both the Raman spectra and the corresponding band-area analysis.

From an applied perspective, thiolutin has emerged as a multifunctional compound with biomedical relevance beyond its classical use as a transcriptional inhibitor. Recent studies report anti-inflammatory effects through suppression of NLRP3 inflammasome activation and antitumor activity in preclinical cancer models^50–52^.

**Fig. 11:**
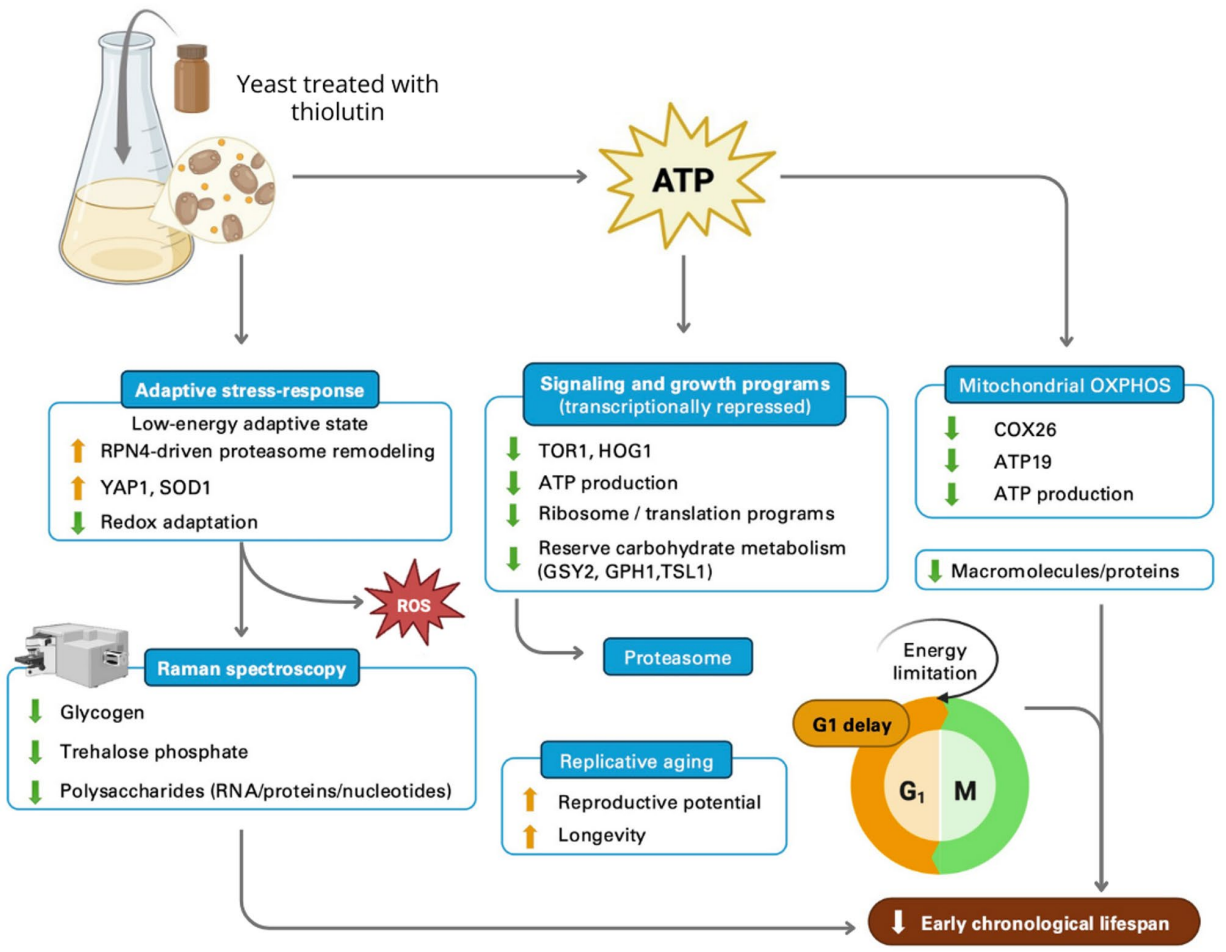
Integrative model summarizing the cellular consequences of thiolutin exposure in budding yeast *Saccharomyces cerevisiae*.

To integrate the phenotypic, transcriptomic, metabolic, and biochemical observations obtained in this study, we propose a conceptual model summarizing the cellular consequences of thiolutin exposure (Fig. 11). This model is intended to provide an integrative framework rather than a definitive mechanistic pathway. The observed phenotypes are consistent with a transcription–energy coupling mechanism, in which reduced ATP availability shifts cells from growth toward maintenance, extending replicative lifespan at the expense of early chronological survival.

## Conclusions

This study provides a comprehensive and integrative view of how thiolutin reshapes yeast physiology, aging, and metabolism by coupling transcriptional inhibition with energy limitation and adaptive stress responses. By combining lifespan analyses, RNA-Seq, targeted RT–qPCR, and FT-Raman spectroscopy, we demonstrate that thiolutin induces a coherent systems-level reprogramming rather than a single linear effect. Our data show that thiolutin markedly extends replicative lifespan by increasing reproductive potential and prolonging the mitotically active phase, despite slowing growth and reducing intracellular ATP levels. This phenotype is tightly associated with global repression of ribosome biogenesis, translation, and mitochondrial oxidative phosphorylation, together with downregulation of TOR1-dependent growth programs. These changes define a low-energy, low-anabolic cellular state that favors maintenance over proliferation—an established hallmark of longevity in yeast. At the same time, thiolutin elicits a selective adaptive stress response rather than a generalized stress program. Strong induction of RPN4 and redox-responsive genes such as YAP1 and SOD1 indicates reinforcement of proteostasis and antioxidant defenses, whereas the lack of HOG1 induction demonstrates that canonical stress-activated MAPK signaling is not engaged. Importantly, transcriptomic alterations were directly mirrored by biochemical changes detected by FT-Raman spectroscopy, which revealed a global reduction in macromolecular content (RNA, proteins, lipids, and carbohydrates) and confirmed that transcriptional reprogramming translates into a distinct biochemical fingerprint. In contrast to its beneficial effects on replicative aging, thiolutin accelerates early chronological aging. Reduced expression of reserve carbohydrate metabolism genes (GPH1, GSY2, TSL1), together with Raman-based evidence of diminished carbohydrate-related signals, suggests an impaired ability to establish a protective, storage-oriented metabolic state during the transition to stationary phase. This divergence highlights a fundamental trade-off: energy-limited transcription promotes longevity in mitotic active cells but compromises early survival of post-mitotic populations, likely due to persistent oxidative stress and reduced metabolic buffering capacity. Collectively, our findings support a unifying model in which thiolutin drives an energy-restricted, stress-adaptive state that extends replicative lifespan through coordinated repression of growth and biosynthetic pathways, reinforcement of proteostasis, and metabolic downscaling. These results underscore the importance of considering thiolutin as a pleiotropic modulator of cellular physiology rather than a simple transcriptional inhibitor. Beyond yeast aging, this work provides a conceptual framework for interpreting thiolutin’s emerging biomedical activities and highlights transcription–energy coupling as a conserved axis linking metabolism, stress adaptation, and longevity.

## Supplementary Information

Below is the link to the electronic supplementary material.

Supplementary Material 1

## Data Availability

The RNA-seq data supporting the findings of this study have been deposited in NCBI’s Gene Expression Omnibus and are accessible through GEO Series accession number GSE320120 (https://www.ncbi.nlm.nih.gov/geo/query/acc.cgi? acc=GSE320120) “.

## Declarations

### Accession codes

The RNA-seq data supporting the findings of this study have been deposited in NCBI’s Gene Expression Omnibus and are accessible through GEO Series accession number GSE320120 (https://www.ncbi.nlm.nih.gov/geo/query/acc.cgi?acc=GSE320120).
