# Supplementary material for: Thiolutin extends replicative lifespan by rewiring yeast transcription and metabolism
Source: Sci Rep. 2026 Mar 1;16:11498. doi: 10.1038/s41598-026-42387-1 (PMC13057240; doi:10.1038/s41598-026-42387-1)
Supplement: Supplementary file 1 — Supplementary Material 1 [file 41598_2026_42387_MOESM1_ESM.pdf]

Table S1. RNA-seq analysis of differential gene expression in *Saccharomyces cerevisiae* BY4741 cells treated with 3 μg/mL thiolutin compared with untreated control.

Gene	BaseMean	Estimated_Fo	log2_Estimate	Wald_stat	p_value_not_	FDR_adjusted
YCL026C-B	8810,58	75,04	6,23	43,9912955	0	0
YCR105W	13349,33	111,88	6,81	46,2629645	0	0
YER103W	342724,32	49,02	5,62	44,865751	0	0
YHR054C	10092,49	753,68	9,56	45,0586243	0	0
YKL086W	7253,7	107,25	6,74	39,0014298	0	0
YML131W	16070,32	29,69	4,89	37,5003498	9,091E-308	9,412E-305
YGR142W	286501,64	52,71	5,72	36,0252488	3,367E-284	2,988E-281
YNL036W	6003,72	28,04	4,81	34,9406506	1,796E-267	1,394E-264
YOR382W	19919,74	22,76	4,51	34,1682207	7,172E-256	4,95E-253
YGR161C	16203,01	21,29	4,41	33,0322924	2,794E-239	1,736E-236
YGL256W	11076,66	19,19	4,26	32,3332136	2,389E-229	1,349E-226
YPL250C	24612,67	28,88	4,85	31,8646893	8,24E-223	4,266E-220
YOR383C	32362,26	15,86	3,99	31,5143827	5,519E-218	2,637E-215
YGL255W	11167,07	43,8	5,45	30,1424038	1,349E-199	5,986E-197
YCR102C	5633,14	53,68	5,75	30,0343715	3,493E-198	1,447E-195
YDL243C	4368,49	20,79	4,38	29,9157315	1,229E-196	4,77E-194
YOL151W	15771,31	25,56	4,68	28,5719559	1,499E-179	5,478E-177
YNL008C	10551,28	12,91	3,69	27,3411302	1,377E-164	4,751E-162
YBR101C	36600,42	12,33	3,62	27,2979004	4,492E-164	1,469E-161
YLR216C	74886,22	17,29	4,11	27,0955079	1,112E-161	3,455E-159
YBR119W	8033,78	13,3	3,73	26,9603838	4,31E-160	1,275E-157
YDL020C	27567,36	11,94	3,58	26,9316684	9,354E-160	2,641E-157
YER104W	2506,11	16,07	4,01	26,8568089	7,023E-159	1,897E-156
YOL152W	1756,81	18,54	4,21	26,5570218	2,131E-155	5,515E-153
YPL171C	4807,42	59,08	5,88	26,4320821	5,864E-154	1,457E-151
YKL084W	3881,6	14,48	3,86	26,0161424	3,252E-149	7,771E-147
YJL051W	5203,73	11,07	3,47	25,8975338	7,1E-148	1,633E-145
YPL240C	221428,23	14,15	3,82	25,773392	1,763E-146	3,911E-144
YDR534C	1939,77	19,22	4,26	25,5879017	2,08E-144	4,456E-142
YCR021C	114723,97	9,05	3,18	25,2681119	7,163E-141	1,483E-138
YOR298W	1256,94	20,55	4,36	25,2398351	1,464E-140	2,935E-138
YNR069C	2707,81	12,06	3,59	24,931507	3,389E-137	6,579E-135
YFL056C	2871,7	14,5	3,86	24,9052231	6,531E-137	1,229E-134
YPL088W	2521,98	11,26	3,49	24,4843189	2,17E-132	3,965E-130
YKL071W	1812,2	12,8	3,68	24,3757433	3,093E-131	5,49E-129
YPR160W	21177,68	0,06	-4,1	-24,3508341	5,681E-131	9,802E-129
YNCG0010W	1650,59	14,93	3,9	24,1654697	5,135E-129	8,621E-127
YNL077W	11893,67	10,07	3,33	24,1251207	1,363E-128	2,228E-126
YOL029C	2623,08	10,74	3,42	24,1005817	2,465E-128	3,926E-126
YLL026W	407627,66	10,7	3,42	24,0309358	1,321E-127	2,052E-125
YLR460C	5663,69	25,51	4,67	23,8098059	2,643E-125	4,005E-123
YHR175W	6760,6	8,72	3,12	23,763936	7,886E-125	1,166E-122
YNR068C	2237,59	11,58	3,53	23,6817259	5,563E-124	8,037E-122

YGL062W	11825,96	8,47	3,08	23,4400243	1,671E-121	2,359E-119
YLR108C	4391,94	19,84	4,31	23,2356578	1,987E-119	2,742E-117
YBR098W	2995,9	10,32	3,37	22,9215863	2,831E-116	3,823E-114
YPL239W	14561,59	12,1	3,6	22,8695725	9,334E-116	1,234E-113
YCR020C	955,44	18,2	4,19	22,6376431	1,847E-113	2,39E-111
YCL026C-A	574,3	59,42	5,89	22,6360507	1,914E-113	2,427E-111
YOL028C	3112,01	9,42	3,24	22,6161261	3,008E-113	3,737E-111
YDL168W	9200,42	9,28	3,21	22,6104589	3,42E-113	4,165E-111
YJL033W	11280,67	19,89	4,31	22,5399749	1,684E-112	2,012E-110
YJR005C-A	588,04	66,55	6,06	22,4370706	1,712E-111	2,006E-109
YOL036W	12053,22	9,82	3,3	22,4059837	3,441E-111	3,959E-109
YCR025C	648,67	24	4,59	22,2865118	4,994E-110	5,641E-108
YBR296C	4483,86	30,81	4,95	21,9859419	3,926E-107	4,355E-105
YML100W	25355,15	0,14	-2,82	-21,9710736	5,447E-107	5,936E-105
YOR344C	15749,25	8,34	3,06	21,9676251	5,876E-107	6,294E-105
YDR256C	2328,12	9,07	3,18	21,8852708	3,589E-106	3,779E-104
YDL182W	11525,64	10,97	3,45	21,7942431	2,631E-105	2,724E-103
YOL154W	1348,17	13,84	3,79	21,6075059	1,527E-103	1,555E-101
Q0158	924,91	13,87	3,79	21,4897421	1,942E-102	1,946E-100
YDR171W	117788,3	6,86	2,78	21,3106942	9,035E-101	8,908E-99
YLL055W	3451,59	8,51	3,09	21,1146891	5,829E-99	5,658E-97
YNCA0003W	561,2	25,39	4,67	21,0150821	4,7742E-98	4,5626E-96
YHL047C	8538,21	10,01	3,32	21,0127614	5,0133E-98	4,7186E-96
YGR209C	37228,89	12,49	3,64	20,884076	7,4736E-97	6,9293E-95
YJL089W	600,65	17,64	4,14	20,6789802	5,3561E-95	4,893E-93
YKR071C	15681,71	10,55	3,4	20,6315968	1,4285E-94	1,2861E-92
YML027W	2133,25	8,13	3,02	20,5948541	3,0521E-94	2,7085E-92
YHL040C	4187,83	7,74	2,95	20,3969206	1,7808E-92	1,5581E-90
YPL241C	2554,04	8,26	3,05	20,2100406	7,9882E-91	6,8921E-89
YJL144W	13086,78	8,86	3,15	20,0931003	8,4798E-90	7,216E-88
YHR156C	1851,9	8,1	3,02	19,9309229	2,1948E-88	1,8424E-86
YLR077W	5131,83	6,55	2,71	19,7546946	7,3096E-87	6,0543E-85
YOL149W	6160,14	9,71	3,28	19,3978552	8,0459E-84	6,5765E-82
YAL037C-A	687,7	303,89	8,25	19,3500663	2,0358E-83	1,6424E-81
YLR214W	4582,23	10,52	3,4	19,2347397	1,8949E-82	1,5091E-80
YFL016C	14183,32	5,35	2,42	19,1024505	2,4091E-81	1,8943E-79
YPL052W	1291,88	8,72	3,12	19,0163202	1,2495E-80	9,7025E-79
YPR036W-A	20593,01	5,29	2,4	18,9913442	2,0111E-80	1,5424E-78
YLR064W	7059,77	5,51	2,46	18,8775437	1,7452E-79	1,3221E-77
YKL096W-A	29317,62	0,15	-2,7	-18,8238687	4,8138E-79	3,6028E-77
YML091C	15919,23	5,99	2,58	18,7354946	2,5427E-78	1,8804E-76
YML007W	12823,68	6,37	2,67	18,7322257	2,7037E-78	1,9759E-76
YOL113W	6317,47	8,22	3,04	18,4703773	3,5758E-76	2,5829E-74
YGR197C	2728,23	5,91	2,56	18,4409395	6,166E-76	4,4027E-74
YNL208W	20654,82	6,72	2,75	18,2041489	4,7843E-74	3,3773E-72
YLR111W	362,96	27,9	4,8	18,1992263	5,2342E-74	3,6533E-72
YDR034C-D	1763,53	7,98	3	18,189222	6,2826E-74	4,3364E-72
YLL056C	1148,65	8,45	3,08	18,1308013	1,8209E-73	1,243E-71
YPL005W	2280,12	6,04	2,6	18,0320903	1,0909E-72	7,3659E-71
YOL157C	1073,22	11,23	3,49	17,9697347	3,3632E-72	2,2465E-70

YHR179W	79707,61	6,63	2,73	17,9483516	4,9436E-72	3,267E-70
YDL184C	22644,47	4,95	2,31	17,7869572	8,9194E-71	5,8323E-69
YLR215C	1881,17	6,18	2,63	17,5822696	3,3682E-69	2,1795E-67
YBR082C	38341,74	4,55	2,19	17,50285	1,3628E-68	8,7273E-67
YDR253C	1406,48	8,14	3,02	17,4555191	3,1252E-68	1,981E-66
YLR109W	121092,57	8,53	3,09	17,3679365	1,4432E-67	9,0559E-66
YAL037W	1218,22	6,94	2,8	17,2515959	1,0885E-66	6,7618E-65
YGR027W-B	3414,34	5,12	2,36	17,2440739	1,2398E-66	7,6256E-65
YML130C	21881,86	11,34	3,5	17,2364834	1,4138E-66	8,6103E-65
YCR020W-B	709,75	8,68	3,12	17,1455273	6,788E-66	4,0939E-64
YLR136C	1915,05	5,69	2,51	17,1385423	7,6546E-66	4,5721E-64
YFR032C-A	3353,34	5,7	2,51	17,0943811	1,6342E-65	9,6683E-64
YPR167C	2069,56	6,8	2,77	17,0106613	6,8461E-65	4,0121E-63
YCL027W	2841,08	6,8	2,76	16,982804	1,101E-64	6,3918E-63
YNL136W	3776,73	4,89	2,29	16,9683645	1,408E-64	8,0985E-63
YHR146W	14865,25	5,08	2,34	16,9333097	2,556E-64	1,4567E-62
YML028W	93795,32	4,73	2,24	16,9114549	3,7045E-64	2,092E-62
YOL119C	2928,52	5,35	2,42	16,7973291	2,5528E-63	1,4287E-61
YDL133C-A	17983,03	7,95	2,99	16,7207075	9,2613E-63	5,1367E-61
YDR077W	52377,17	0,23	-2,12	-16,6955375	1,4124E-62	7,7645E-61
YER053C	7450,95	0,14	-2,84	-16,6873282	1,6206E-62	8,831E-61
YPR124W	5036,81	5,33	2,41	16,6243224	4,6459E-62	2,5096E-60
YBR085C-A	13129,05	4,48	2,16	16,5648965	1,2499E-61	6,6937E-60
YKR066C	25279,61	7,27	2,86	16,5329916	2,1234E-61	1,1274E-59
YGR287C	1251,58	6,34	2,66	16,4959594	3,9228E-61	2,0651E-59
YMR266W	9696,73	5,63	2,49	16,4854316	4,6695E-61	2,4376E-59
YPR174C	1363,65	6,36	2,67	16,3929112	2,149E-60	1,1125E-58
YPR152C	1865,63	6,24	2,64	16,3244144	6,6173E-60	3,3972E-58
YDR485C	4774,98	4,44	2,15	16,2511988	2,1904E-59	1,1153E-57
YHL028W	1018,64	0,08	-3,63	-16,2506394	2,2105E-59	1,1164E-57
YDL049C	548,81	8,82	3,14	16,2315062	3,0196E-59	1,5127E-57
YDL099W	6262,85	4,31	2,11	16,1962761	5,3575E-59	2,6624E-57
YGR072W	2664,55	5,22	2,39	16,0936432	2,827E-58	1,3938E-56
YER159C	8986,46	5,51	2,46	16,0537249	5,3834E-58	2,6332E-56
YML041C	1726,69	5,04	2,33	15,9274094	4,0895E-57	1,9847E-55
YDR162C	2520,14	4,63	2,21	15,852136	1,3588E-56	6,5434E-55
YDR353W	25713,11	4,76	2,25	15,809628	2,6704E-56	1,2745E-54
YLL060C	2275,35	7,35	2,88	15,8092207	2,6877E-56	1,2745E-54
YDR151C	4933,23	4,26	2,09	15,7917932	3,5436E-56	1,6676E-54
YPL053C	4820,86	4,6	2,2	15,776866	4,4893E-56	2,0968E-54
YDR258C	47801,25	4,16	2,06	15,7071396	1,3514E-55	6,2649E-54
YNL007C	42784,92	4,05	2,02	15,68078	2,0472E-55	9,4204E-54
YOR226C	5885,02	7,44	2,9	15,6578775	2,9353E-55	1,3407E-53
YJL056C	1458,58	5,5	2,46	15,4438004	8,3059E-54	3,7661E-52
YGR146C	6008,78	6,49	2,7	15,4236414	1,1352E-53	5,11E-52
YNL213C	1814,6	4,72	2,24	15,3408508	4,0781E-53	1,8226E-51
YDR069C	4584,98	4,49	2,17	15,3318821	4,6822E-53	2,0776E-51
YOL077W-A	1310,69	0,17	-2,54	-15,3095932	6,5975E-53	2,9067E-51
YPL202C	3051,55	4,82	2,27	15,2919573	8,6511E-53	3,7845E-51
YNCH0014W	253,72	19,98	4,32	15,2245054	2,4319E-52	1,0564E-50

YCR098C	648,3	8,7	3,12	15,2015051	3,4559E-52	1,4908E-50
YML116W	1891,03	4,52	2,18	15,190581	4,0828E-52	1,7491E-50
YPR065W	6553,75	4,37	2,13	15,1749176	5,1843E-52	2,2058E-50
YOR268C	239,29	18,54	4,21	15,1721853	5,4047E-52	2,2839E-50
YML071C	3188,24	4,29	2,1	15,1626585	6,2488E-52	2,6228E-50
YDR270W	2256,57	4,53	2,18	15,1564027	6,8732E-52	2,8655E-50
YHL021C	11689,68	0,16	-2,63	-15,1300412	1,0263E-51	4,2503E-50
YLR134W	1020,49	5,74	2,52	15,1242355	1,1209E-51	4,6115E-50
YJL153C	744,05	6,27	2,65	15,0962559	1,7139E-51	7,0046E-50
YDL183C	2306,71	7,88	2,98	15,0940316	1,7727E-51	7,1975E-50
YDR356W	4497,95	4,7	2,23	15,072697	2,4491E-51	9,879E-50
YBR008C	1469,47	4,78	2,26	15,0622283	2,8695E-51	1,15E-49
YLR260W	3013,68	4,94	2,3	15,0519394	3,3526E-51	1,335E-49
YLR258W	17697,44	0,21	-2,22	-15,0050353	6,8055E-51	2,6927E-49
YNL284C-B	1679,76	4,98	2,32	14,9914564	8,3502E-51	3,283E-49
YGR038C-B	10907,2	4,58	2,19	14,9170199	2,5543E-50	9,9794E-49
YNL241C	18756,65	3,74	1,9	14,881737	4,3311E-50	1,6815E-48
YJR010W	2500,17	5,03	2,33	14,8633802	5,6976E-50	2,1984E-48
YLR309C	7781,69	4,64	2,21	14,766901	2,3947E-49	9,1828E-48
YBL033C	4612,98	4,22	2,08	14,7303746	4,1142E-49	1,5679E-47
YPL014W	3425,73	0,24	-2,08	-14,7246294	4,4792E-49	1,6966E-47
YIL125W	4803,35	0,24	-2,06	-14,6106591	2,4018E-48	9,0425E-47
YEL072W	905,56	5,54	2,47	14,5928415	3,1193E-48	1,1673E-46
YOR303W	3670,63	3,99	2	14,5373357	7,0275E-48	2,6141E-46
YER044C-A	482,84	7,54	2,91	14,5068168	1,0969E-47	4,0561E-46
YPR007C	366,29	9,52	3,25	14,4745391	1,755E-47	6,4509E-46
YJL123C	10519,58	3,65	1,87	14,4460147	2,6562E-47	9,7059E-46
YIL108W	3688,04	3,98	1,99	14,4445357	2,7138E-47	9,8585E-46
YHR046C	2959,88	4,22	2,08	14,4140276	4,2235E-47	1,5254E-45
YJL088W	342,03	9,83	3,3	14,4108309	4,4236E-47	1,5884E-45
YGR096W	1010,72	5,03	2,33	14,4033112	4,9323E-47	1,7609E-45
YIR017C	2130,74	10,34	3,37	14,3131585	1,811E-46	6,4285E-45
YPL256C	2531,61	0,23	-2,1	-14,2146549	7,4317E-46	2,623E-44
YCR005C	7179	3,95	1,98	14,21071	7,8624E-46	2,7594E-44
YKL164C	10649,99	0,28	-1,81	-14,1756896	1,2956E-45	4,5216E-44
YNCK0020C	407,79	15,66	3,97	14,1356802	2,2891E-45	7,9441E-44
YPL051W	1495,2	5,89	2,56	14,1195562	2,878E-45	9,9322E-44
YBR150C	2486,35	4	2	14,116637	2,9997E-45	1,0295E-43
YIL117C	7120,31	5,76	2,52	14,0783281	5,1617E-45	1,7618E-43
YPR002W	628,64	6,98	2,8	14,0660892	6,1371E-45	2,0832E-43
YJL133C-A	3725,29	12,85	3,68	14,0407767	8,7746E-45	2,9624E-43
YMR173W	43138,52	3,66	1,87	14,0061582	1,4293E-44	4,7994E-43
YLR337C	3446,43	0,24	-2,03	-13,9697736	2,3838E-44	7,9615E-43
YJR139C	15600,93	3,43	1,78	13,9664397	2,4981E-44	8,2984E-43
YJL107C	535,08	10,18	3,35	13,9411404	3,562E-44	1,177E-42
YLR126C	1811,84	4,18	2,07	13,9291777	4,2118E-44	1,3843E-42
YGR254W	324372,14	6,25	2,64	13,8556841	1,1753E-43	3,8427E-42
YGR009C	5520,9	3,5	1,81	13,8440703	1,3816E-43	4,4933E-42
YOL043C	1841,43	3,99	2	13,8261875	1,7716E-43	5,732E-42
YOR027W	69679,52	6,47	2,69	13,821874	1,8811E-43	6,0545E-42

YDR365C	11859,63	4,51	2,17	13,7341942	6,3357E-43	2,0287E-41
YGR211W	20957,19	6,72	2,75	13,6814711	1,3101E-42	4,1737E-41
YFL026W	8834,28	0,25	-1,98	-13,6482741	2,0672E-42	6,5516E-41
YDR261W-B	1195,97	5,83	2,54	13,6305408	2,6362E-42	8,3127E-41
YER037W	6171,76	3,44	1,78	13,6034192	3,8214E-42	1,1989E-40
YDR232W	3476,37	4,4	2,14	13,51861	1,2145E-41	3,7912E-40
YMR152W	2164,93	0,22	-2,15	-13,4824937	1,9829E-41	6,1589E-40
YDR379W	4402,47	3,52	1,81	13,4595974	2,7039E-41	8,3564E-40
YGL158W	290,4	9,67	3,27	13,4280792	4,1401E-41	1,2732E-39
YCR104W	224,71	11,8	3,56	13,4248765	4,323E-41	1,3229E-39
YHR174W	477986,08	3,45	1,79	13,404465	5,6932E-41	1,7252E-39
YNL009W	1089,1	5,95	2,57	13,4045994	5,6829E-41	1,7252E-39
YJR046W	3519,3	4,56	2,19	13,3916916	6,7623E-41	2,0392E-39
YMR161W	2749,22	3,59	1,84	13,3836754	7,5328E-41	2,2606E-39
YOR267C	10713,75	3,65	1,87	13,3604648	1,0292E-40	3,0737E-39
YEL065W	4199,07	5,24	2,39	13,3354767	1,4393E-40	4,278E-39
YJL102W	895,88	0,19	-2,41	-13,3297608	1,554E-40	4,5967E-39
YOL032W	4466,7	3,95	1,98	13,3244198	1,6693E-40	4,9144E-39
YFL002W-A	233,1	12,91	3,69	13,246179	4,7477E-40	1,3912E-38
YGL035C	4012,6	4,25	2,09	13,2374568	5,3324E-40	1,5552E-38
YOR028C	1701,32	4,93	2,3	13,2146661	7,2206E-40	2,096E-38
YMR250W	8629,7	0,18	-2,48	-13,2112217	7,5588E-40	2,184E-38
YMR045C	3849,19	5,79	2,53	13,137	2,0208E-39	5,8117E-38
YDR354W	4032,37	4	2	13,1304384	2,2038E-39	6,3087E-38
YNL107W	1544,73	3,91	1,97	13,1085117	2,9432E-39	8,3867E-38
YJR009C	311592,82	3,47	1,8	13,1023451	3,1924E-39	9,0553E-38
YKL201C	4155,5	4,18	2,06	13,0750565	4,5722E-39	1,291E-37
YLR205C	692,66	5,64	2,5	13,0187569	9,5716E-39	2,6905E-37
YHR176W	1271,95	5,04	2,33	13,015164	1,0033E-38	2,8073E-37
YNCG0014C	338,74	11,58	3,53	12,9891158	1,4105E-38	3,9291E-37
YDL204W	5767,33	0,07	-3,8	-12,9822188	1,5434E-38	4,2803E-37
YPL247C	3635,77	0,16	-2,62	-12,9629431	1,9848E-38	5,4799E-37
YJR134C	4831,57	3,49	1,8	12,9480394	2,4103E-38	6,6252E-37
YNL006W	4911,5	3,29	1,72	12,9201602	3,4642E-38	9,48E-37
YDR153C	4351,43	3,26	1,71	12,9122743	3,838E-38	1,0457E-36
YAL005C	139276,75	3,14	1,65	12,8806517	5,7845E-38	1,5691E-36
YPL024W	578,19	5,39	2,43	12,8703775	6,6078E-38	1,7847E-36
YNL212W	5104,35	3,53	1,82	12,8467533	8,9696E-38	2,4121E-36
YDR132C	2087,85	3,63	1,86	12,8049035	1,5392E-37	4,1213E-36
YPL168W	2012,11	3,79	1,92	12,7918263	1,8214E-37	4,8561E-36
YOR173W	4058,72	0,13	-2,96	-12,7879714	1,9141E-37	5,0812E-36
YGR108W	1328,81	0,17	-2,53	-12,7871517	1,9343E-37	5,1132E-36
YDL180W	3164,85	3,33	1,74	12,7422807	3,4418E-37	9,0596E-36
YFR032C-B	224,65	12,14	3,6	12,7415127	3,4759E-37	9,1107E-36
YGL188C-A	278,56	9,12	3,19	12,7217198	4,4789E-37	1,169E-35
YNL134C	17028,7	3,14	1,65	12,7051967	5,533E-37	1,4381E-35
YLR267W	1814,79	7,89	2,98	12,6798037	7,6523E-37	1,9807E-35
YLR301W	6377,41	3,23	1,69	12,6608004	9,75E-37	2,5132E-35
YOR190W	937,53	5,27	2,4	12,5880983	2,4552E-36	6,3023E-35
YER156C	1695,64	0,26	-1,92	-12,5750705	2,8954E-36	7,4018E-35

YMR186W	112113,64	7,04	2,82	12,5309468	5,0555E-36	1,2871E-34
YBR244W	14760,97	8,11	3,02	12,5286983	5,2009E-36	1,3187E-34
YJL034W	56165,31	7,23	2,85	12,5262465	5,3642E-36	1,3546E-34
YPL020C	4193,07	3,79	1,92	12,4543987	1,3235E-35	3,3285E-34
YDR002W	11451,88	3,02	1,59	12,4406376	1,5724E-35	3,9387E-34
YJR029W	520,2	5,17	2,37	12,4174479	2,1016E-35	5,243E-34
YDR003W	1821,12	4,13	2,05	12,4164668	2,1275E-35	5,2865E-34
YNL300W	1582,51	0,27	-1,9	-12,3936739	2,8279E-35	6,9987E-34
YKR069W	1724,82	4,15	2,05	12,2916716	1,0041E-34	2,4751E-33
YGR095C	2008,89	3,46	1,79	12,2849792	1,0907E-34	2,6781E-33
YKR089C	5211,01	0,3	-1,73	-12,2842369	1,1008E-34	2,6922E-33
YGR035C	861,38	3,99	2	12,2737097	1,2537E-34	3,0542E-33
YCR012W	341675,4	4,31	2,11	12,2600589	1,4839E-34	3,6008E-33
YDR281C	448,44	5,79	2,53	12,2512587	1,6541E-34	3,9981E-33
YDL171C	6953,69	3,81	1,93	12,2309069	2,1255E-34	5,1177E-33
YJL016W	2335,17	0,24	-2,06	-12,2017073	3,0437E-34	7,3003E-33
YHR009C	3954,9	0,31	-1,68	-12,192626	3,4028E-34	8,13E-33
YIL045W	2953,62	0,16	-2,65	-12,191092	3,4674E-34	8,2528E-33
YLR346C	574,65	5,94	2,57	12,1779483	4,074E-34	9,6595E-33
YPL170W	2891,8	3,28	1,71	12,1620287	4,9514E-34	1,1695E-32
Q0045	155,35	15,36	3,94	12,1560138	5,3297E-34	1,2541E-32
YBR237W	2786,61	3,22	1,69	12,1378042	6,6589E-34	1,5609E-32
YHL015W	90705,61	2,85	1,51	12,1166344	8,6228E-34	2,0137E-32
YDR313C	1555,75	3,7	1,89	12,0971495	1,0934E-33	2,544E-32
YIL144W	2958,63	3,31	1,73	12,0945432	1,1287E-33	2,6162E-32
Q0055	143,24	30,76	4,94	12,0734683	1,4585E-33	3,3682E-32
YEL071W	13335,42	8,04	3,01	12,0640719	1,635E-33	3,7616E-32
YMR008C	4064,36	0,32	-1,66	-12,047283	2,0045E-33	4,5948E-32
YDR074W	20524,87	0,34	-1,58	-12,0193967	2,8102E-33	6,4179E-32
YLR177W	4676,8	0,32	-1,66	-11,9880802	4,103E-33	9,3363E-32
YPL146C	3030,52	4,98	2,31	11,9854619	4,2348E-33	9,6009E-32
YKL075C	1836,79	3,28	1,71	11,9808454	4,4773E-33	1,0114E-31
YPR173C	5175,15	3,1	1,63	11,9486922	6,5955E-33	1,4845E-31
YKL062W	2445,03	0,27	-1,88	-11,943121	7,0526E-33	1,5816E-31
YBR086C	5878,4	0,34	-1,57	-11,9070586	1,0875E-32	2,43E-31
YBR041W	1867,06	0,26	-1,96	-11,8874525	1,3754E-32	3,0623E-31
YIL165C	515,03	6,2	2,63	11,8805944	1,493E-32	3,3124E-31
YHL020C	2116,93	3,78	1,92	11,8782421	1,5356E-32	3,3948E-31
YGL157W	5397,22	3,04	1,6	11,8538218	2,056E-32	4,5289E-31
YJR116W	1195,62	3,48	1,8	11,8195118	3,0948E-32	6,7932E-31
YBR195C	1122,7	3,54	1,82	11,8086633	3,5211E-32	7,7019E-31
YMR128W	6369,94	5,79	2,53	11,7776214	5,0909E-32	1,1096E-30
YOR086C	4320,59	0,33	-1,58	-11,772981	5,379E-32	1,1683E-30
YDL065C	3779,56	3	1,59	11,7511086	6,9698E-32	1,5086E-30
YDR345C	25179,41	4,26	2,09	11,7184104	1,0258E-31	2,2126E-30
YNL124W	1653,53	3,23	1,69	11,6976478	1,3104E-31	2,8166E-30
YAL011W	1192,19	3,49	1,8	11,6924869	1,3925E-31	2,9828E-30
YPL042C	1634,84	4,04	2,01	11,6834805	1,5482E-31	3,305E-30
YGL229C	1105,92	0,27	-1,89	-11,658493	2,0769E-31	4,4183E-30
YMR105C	24347,19	0,23	-2,11	-11,6391957	2,6046E-31	5,5221E-30

YIL164C	934,46	3,99	2	11,6370876	2,6698E-31	5,641E-30
YPL106C	70014,81	5,09	2,35	11,6302909	2,8911E-31	6,088E-30
YNL207W	2902,12	4,01	2	11,6184109	3,3226E-31	6,9729E-30
YMR077C	1002,87	3,55	1,83	11,6144871	3,4787E-31	7,276E-30
YGR149W	1565,32	0,23	-2,11	-11,5725544	5,6767E-31	1,1833E-29
YPR158W	4988,02	4,58	2,2	11,5638863	6,2801E-31	1,3047E-29
YPL237W	13822,95	3,18	1,67	11,5484423	7,5171E-31	1,5565E-29
YOR261C	7381,31	3,04	1,61	11,5383622	8,4519E-31	1,7443E-29
Q0050	133,97	16,32	4,03	11,5276095	9,5766E-31	1,9699E-29
YNCK0009W	196,34	14,83	3,89	11,5134452	1,1288E-30	2,3142E-29
YMR136W	1873,76	0,22	-2,17	-11,5026137	1,2798E-30	2,6152E-29
YOR176W	3489,56	0,31	-1,71	-11,4931069	1,4288E-30	2,91E-29
YOL144W	1317,03	3,98	1,99	11,4847918	1,5731E-30	3,1936E-29
YAL012W	39276,46	4,4	2,14	11,4808565	1,6464E-30	3,3314E-29
YGL244W	6747,66	3,18	1,67	11,479732	1,668E-30	3,3641E-29
YKL035W	29848,6	0,34	-1,54	-11,4716631	1,8311E-30	3,6811E-29
YIR011C	3496,12	3,47	1,79	11,4675214	1,9208E-30	3,8491E-29
YEL024W	2050,21	0,2	-2,33	-11,4184888	3,3806E-30	6,7525E-29
YKR058W	3997,67	0,3	-1,74	-11,4032398	4,0284E-30	8,0207E-29
YDL058W	6224,83	3,9	1,96	11,401707	4,1E-30	8,1371E-29
YBR118W	125193,9	3	1,59	11,3973336	4,3112E-30	8,5291E-29
YPR184W	7156,73	0,16	-2,63	-11,3847049	4,9837E-30	9,8281E-29
YHR162W	15450,66	4,02	2,01	11,3808516	5,2089E-30	1,024E-28
YBL029C-A	1390,39	0,16	-2,63	-11,3722298	5,75E-30	1,1268E-28
YJL189W	11388,1	3,39	1,76	11,3637382	6,3374E-30	1,238E-28
YPL135W	3441,44	2,99	1,58	11,3599551	6,6179E-30	1,2887E-28
YNL200C	2676,45	0,11	-3,25	-11,3525935	7,1995E-30	1,3976E-28
YIL149C	7345,48	3,4	1,77	11,3401573	8,2995E-30	1,6061E-28
YDR391C	1693,25	0,3	-1,72	-11,3344428	8,8594E-30	1,7092E-28
YNL215W	3149,93	2,93	1,55	11,3308607	9,2293E-30	1,775E-28
YJL130C	12803,48	3,83	1,94	11,3020894	1,2813E-29	2,4567E-28
YBR214W	8363,22	0,3	-1,73	-11,297665	1,3475E-29	2,5757E-28
YJL082W	6646,72	3,42	1,78	11,2942661	1,4007E-29	2,669E-28
YMR039C	6360,15	2,79	1,48	11,2896421	1,4764E-29	2,8046E-28
YDL048C	4278,33	0,24	-2,05	-11,2760262	1,7236E-29	3,2643E-28
YHR029C	2626,85	3,25	1,7	11,2375063	2,6681E-29	5,0378E-28
YDR078C	675,49	5,11	2,35	11,2173125	3,3531E-29	6,3119E-28
YOR057W	2767,78	2,96	1,56	11,2150829	3,4386E-29	6,4534E-28
YDR152W	4033,58	3,14	1,65	11,2144118	3,4648E-29	6,483E-28
YKL025C	1898,39	0,31	-1,7	-11,2084637	3,7057E-29	6,9128E-28
YJL108C	504,43	4,23	2,08	11,1871684	4,7124E-29	8,7646E-28
YJR127C	3702,43	0,34	-1,54	-11,1443674	7,6284E-29	1,4146E-27
YOR116C	3278,67	4,72	2,24	11,1269013	9,2806E-29	1,7158E-27
YCR069W	2613,82	0,34	-1,56	-11,1256524	9,4115E-29	1,7348E-27
YKL082C	6084,38	4,9	2,29	11,1139734	1,0728E-28	1,9716E-27
YPL004C	19118,96	0,28	-1,84	-11,1094121	1,129E-28	2,0688E-27
YMR272C	3608,68	0,26	-1,96	-11,0974247	1,2911E-28	2,3589E-27
YOR306C	1860,4	3,15	1,66	11,0774269	1,6145E-28	2,9411E-27
YIL159W	1459,39	3,76	1,91	11,0650853	1,8529E-28	3,3655E-27
YPR101W	1540,94	3,11	1,64	11,0500381	2,1912E-28	3,9685E-27

YBR137W	3334,3	3,67	1,88	11,0331006	2,6458E-28	4,7779E-27
YPR103W	11134,23	2,65	1,4	11,0084364	3,4799E-28	6,2659E-27
YOR120W	2768,32	0,25	-2,03	-11,0004274	3,8033E-28	6,8283E-27
YNR044W	1740,31	0,27	-1,89	-10,9843777	4,5436E-28	8,1339E-27
YPL009C	9443,22	3,47	1,79	10,9582512	6,066E-28	1,0828E-26
YPL178W	3677,41	2,75	1,46	10,9555219	6,2517E-28	1,1128E-26
YBR149W	7797,76	0,29	-1,81	-10,9497591	6,6625E-28	1,1825E-26
YKL121W	1262,93	0,29	-1,8	-10,9442482	7,0803E-28	1,2531E-26
YMR251W	707,38	4,53	2,18	10,9327608	8,0365E-28	1,4183E-26
YMR227C	1945,33	2,97	1,57	10,9308153	8,2107E-28	1,4449E-26
YBL005W-B	3862,92	3,3	1,72	10,9295236	8,3285E-28	1,4615E-26
YPR191W	5965,85	0,23	-2,13	-10,9217319	9,0749E-28	1,588E-26
YNL263C	1820,29	0,31	-1,67	-10,9047384	1,0941E-27	1,9091E-26
YBR265W	1094,87	0,26	-1,93	-10,8972705	1,1877E-27	2,0666E-26
YHR007C-A	398,14	6,59	2,72	10,8951625	1,2155E-27	2,1091E-26
YOR125C	992,48	0,26	-1,97	-10,8848959	1,3605E-27	2,3542E-26
YLL050C	12531,23	2,86	1,51	10,8737213	1,538E-27	2,6539E-26
YOR032C	1200,33	3,19	1,67	10,873185	1,547E-27	2,6621E-26
YMR177W	2015,64	4,17	2,06	10,863241	1,7252E-27	2,9604E-26
YKR003W	1409,94	0,3	-1,72	-10,8541544	1,9056E-27	3,2611E-26
YNL147W	1645,27	3,11	1,64	10,8464089	2,0742E-27	3,5397E-26
YNL080C	2853,78	2,79	1,48	10,828052	2,5349E-27	4,3142E-26
YLR450W	2165,47	0,33	-1,6	-10,8085588	3,1356E-27	5,3219E-26
YNL065W	823,49	3,68	1,88	10,7949892	3,6351E-27	6,1528E-26
YKR011C	5792,33	2,75	1,46	10,7860804	4,0051E-27	6,7608E-26
YDR257C	2214,1	3,52	1,82	10,7800228	4,2778E-27	7,2015E-26
YBR007C	2253,54	3,02	1,59	10,7694768	4,7972E-27	8,0541E-26
YDR004W	2145,38	3,42	1,78	10,767692	4,8911E-27	8,1896E-26
YJL141C	6240,28	0,31	-1,68	-10,7556047	5,5767E-27	9,3125E-26
YGR044C	1961,51	0,29	-1,78	-10,7530305	5,7346E-27	9,5505E-26
YNL217W	2589,37	0,35	-1,5	-10,7442296	6,3086E-27	1,0478E-25
YGR248W	3729,86	0,15	-2,77	-10,7419253	6,4681E-27	1,0715E-25
YBR092C	2291,22	0,29	-1,81	-10,7273033	7,5775E-27	1,2519E-25
YNL231C	5807,08	3,31	1,73	10,7270099	7,6016E-27	1,2526E-25
YDR289C	2552,52	2,99	1,58	10,7129001	8,8544E-27	1,4551E-25
YOR257W	2428,45	2,9	1,54	10,7119983	8,9411E-27	1,4655E-25
YGR271C-A	798,96	3,87	1,95	10,67885	1,2785E-26	2,0899E-25
YJL218W	535,77	4,2	2,07	10,666461	1,4608E-26	2,3818E-25
YKR052C	2374,28	3,31	1,72	10,6461824	1,8166E-26	2,9542E-25
YKR053C	1211,16	3,48	1,8	10,6227958	2,3346E-26	3,7866E-25
YLR167W	65561,61	2,51	1,33	10,6200931	2,4032E-26	3,8877E-25
YIL121W	2944	0,34	-1,54	-10,5938813	3,1812E-26	5,1329E-25
YMR120C	23336,21	0,11	-3,2	-10,5809427	3,6526E-26	5,8783E-25
YDR210W-B	307,53	5,14	2,36	10,5768076	3,8174E-26	6,1276E-25
YGL096W	817,35	0,25	-1,98	-10,5764811	3,8308E-26	6,1332E-25
YOR153W	50512,51	4,25	2,09	10,57549	3,8715E-26	6,1824E-25
YDR516C	51913,37	3,09	1,63	10,5725281	3,9958E-26	6,3645E-25
YKL100C	3332,58	0,36	-1,47	-10,5689656	4,1505E-26	6,594E-25
YNCB0010W	2073,09	0,32	-1,66	-10,567722	4,2059E-26	6,665E-25
YOR385W	2050,63	0,2	-2,31	-10,5631868	4,4142E-26	6,9773E-25

YOL016C	7749,58	0,29	-1,81	-10,5423357	5,5113E-26	8,6894E-25
YGL160W	1747,45	0,32	-1,63	-10,5169969	7,2136E-26	1,1345E-24
YJL106W	964,16	3,19	1,67	10,5010942	8,5385E-26	1,3394E-24
YNCH0008W	546,67	13,52	3,76	10,4969349	8,9231E-26	1,3962E-24
YOL058W	4422,5	6,5	2,7	10,4894809	9,6557E-26	1,5071E-24
YGR210C	5729,65	2,7	1,44	10,4722443	1,1586E-25	1,8039E-24
YEL046C	14110,09	5,6	2,49	10,4708469	1,1759E-25	1,8261E-24
YOR020W-A	2761,58	3,13	1,65	10,4112116	2,204E-25	3,4143E-24
YJL042W	7343,66	2,74	1,45	10,4108334	2,2128E-25	3,4193E-24
YBR076W	523,71	4,85	2,28	10,4098999	2,2346E-25	3,4444E-24
YJL025W	647,56	4,01	2	10,405326	2,3445E-25	3,605E-24
YNL274C	1587,86	0,26	-1,95	-10,384019	2,9317E-25	4,4967E-24
YJL049W	1892,39	2,78	1,48	10,3765107	3,1716E-25	4,8526E-24
YMR119W	1564,35	0,3	-1,71	-10,3703152	3,3841E-25	5,1651E-24
YGL253W	76681,66	4,16	2,06	10,3451436	4,4026E-25	6,7031E-24
YJL116C	1451,56	3,99	2	10,325	5,4319E-25	8,2501E-24
YFR015C	17141,3	0,35	-1,51	-10,3245891	5,4552E-25	8,2653E-24
YDR068W	3797,68	2,67	1,42	10,3184944	5,8128E-25	8,7856E-24
YGL086W	3071,64	2,88	1,52	10,2958497	7,3567E-25	1,1092E-23
YPR153W	443,69	4,3	2,1	10,2847352	8,2569E-25	1,2419E-23
YJL164C	1563,48	0,33	-1,59	-10,2706787	9,553E-25	1,4334E-23
YCL025C	2476,47	0,3	-1,75	-10,2274787	1,4936E-24	2,2357E-23
YPR114W	2554,44	0,36	-1,46	-10,2087598	1,8117E-24	2,7053E-23
YNCO0030W	156,13	241,61	7,92	10,2064014	1,8562E-24	2,7652E-23
YFR053C	53493,22	0,18	-2,46	-10,2046961	1,8891E-24	2,8075E-23
YGL209W	1202,05	2,95	1,56	10,2000438	1,9818E-24	2,9382E-23
YOR007C	19164,15	2,65	1,41	10,1947091	2,0937E-24	3,0967E-23
YNL135C	18736,28	2,94	1,55	10,1722792	2,6367E-24	3,8905E-23
YOL014W	1111,65	0,25	-2,01	-10,1667249	2,7914E-24	4,109E-23
YER042W	3561,68	6,73	2,75	10,1538543	3,1853E-24	4,6778E-23
YNCJ0028C	510,92	0,19	-2,36	-10,1455074	3,4697E-24	5,0834E-23
YNL321W	1420	0,34	-1,54	-10,1417042	3,6075E-24	5,2729E-23
YER027C	2644,91	0,37	-1,44	-10,1397377	3,6809E-24	5,3675E-23
YEL011W	13163,68	0,21	-2,27	-10,1273953	4,1763E-24	6,0756E-23
YKR075C	2547,21	4,06	2,02	10,1233334	4,3533E-24	6,3184E-23
YPL252C	1571,88	2,9	1,54	10,0911861	6,0435E-24	8,7511E-23
YNL175C	3585,75	4,04	2,01	10,0739076	7,2057E-24	1,041E-22
YOR107W	702,67	3,66	1,87	10,0734359	7,2404E-24	1,0436E-22
YKR005C	403,17	3,96	1,98	10,0707764	7,4389E-24	1,0697E-22
YBR296C-A	90,24	55,11	5,78	10,0689492	7,5783E-24	1,0872E-22
YER008C	5261,96	2,73	1,45	10,0645243	7,927E-24	1,1346E-22
YDL069C	702,1	3,32	1,73	10,0430163	9,8612E-24	1,4082E-22
YLL039C	13882,96	2,46	1,3	10,0232678	1,2045E-23	1,7162E-22
YKL143W	3138,87	3,8	1,92	10,0073892	1,4144E-23	2,0105E-22
YBR066C	1702,13	2,89	1,53	10,0047762	1,4522E-23	2,0596E-22
YNL064C	21405,61	3,99	1,99	9,99215247	1,6496E-23	2,3342E-22
YGL121C	771,97	0,14	-2,85	-9,99004355	1,6851E-23	2,379E-22
YEL001C	3873,57	0,39	-1,34	-9,97045467	2,0529E-23	2,8917E-22
YBL046W	1638,12	2,78	1,47	9,95792214	2,3288E-23	3,2729E-22
YBR218C	9281,83	0,4	-1,31	-9,95719022	2,346E-23	3,2897E-22

YPL054W	1160,79	2,96	1,57	9,94504411	2,6506E-23	3,7084E-22
YHR175W-A	969,44	7,09	2,82	9,94332314	2,6968E-23	3,7646E-22
YDR261C-D	1275,52	3,19	1,67	9,93974646	2,7954E-23	3,8935E-22
YLR178C	4679,28	0,19	-2,37	-9,93190821	3,0241E-23	4,2026E-22
YGL232W	3202,31	2,56	1,36	9,92255092	3,3216E-23	4,6057E-22
YJR109C	4436,22	2,62	1,39	9,89782315	4,2543E-23	5,886E-22
YCL031C	3189,75	2,69	1,43	9,8943202	4,4059E-23	6,0821E-22
YML062C	2444,46	2,65	1,4	9,89017889	4,5921E-23	6,325E-22
YOL030W	5124,15	0,34	-1,55	-9,87939537	5,1141E-23	7,0284E-22
YDL173W	4975,62	2,58	1,37	9,87448346	5,3709E-23	7,3651E-22
YNL055C	12872,65	0,29	-1,79	-9,87151312	5,5324E-23	7,5698E-22
YHR108W	3915,88	0,34	-1,56	-9,86294468	6,0256E-23	8,2266E-22
Q0075	100,89	16,86	4,08	9,86214955	6,0735E-23	8,2738E-22
YMR075W	2000,89	2,82	1,5	9,85254379	6,6831E-23	9,0843E-22
YNR020C	646,06	3,42	1,77	9,84173164	7,4419E-23	1,0094E-21
YPR035W	28070,15	0,41	-1,28	-9,83975508	7,5895E-23	1,0272E-21
YFR017C	3777,12	0,17	-2,54	-9,81275466	9,9222E-23	1,3399E-21
YJL046W	992,94	0,32	-1,62	-9,79339707	1,2019E-22	1,6195E-21
YNL076W	3068,02	2,51	1,33	9,78772129	1,2713E-22	1,7094E-21
YGR194C	2969,62	0,29	-1,78	-9,78159602	1,3506E-22	1,8121E-21
YNL050C	1306,71	2,78	1,47	9,78041387	1,3665E-22	1,8295E-21
YBR280C	1398,21	0,34	-1,54	-9,77923735	1,3825E-22	1,8469E-21
YIL017C	1765,43	0,36	-1,48	-9,77589598	1,4289E-22	1,9048E-21
YFL013C	4562,92	2,44	1,29	9,77074596	1,5034E-22	1,9998E-21
YNL110C	1898,59	3,31	1,73	9,77041751	1,5083E-22	2,002E-21
YPL027W	165,52	7,87	2,98	9,76709061	1,5586E-22	2,0644E-21
YBR145W	6432,34	3,66	1,87	9,76598686	1,5757E-22	2,0826E-21
YBR295W	1261,54	3,99	2	9,75842438	1,6977E-22	2,2391E-21
YOR219C	2476,25	0,37	-1,45	-9,72212171	2,4267E-22	3,1938E-21
YCR107W	610,12	3,27	1,71	9,72119148	2,449E-22	3,2163E-21
YBL075C	1760,94	2,76	1,47	9,71552408	2,5891E-22	3,3932E-21
YOR386W	1604,07	0,33	-1,58	-9,6763416	3,8007E-22	4,9706E-21
YOR192C-B	1467,32	4,43	2,15	9,67473437	3,8609E-22	5,0387E-21
YMR011W	14004,1	5,64	2,5	9,662468	4,3525E-22	5,6683E-21
YHR104W	4838,64	0,38	-1,38	-9,65965464	4,4737E-22	5,8139E-21
YDR381W	10940,9	2,45	1,29	9,64657614	5,0824E-22	6,5912E-21
YML124C	6169,25	2,48	1,31	9,6434188	5,2413E-22	6,7831E-21
YHR025W	5719,13	0,41	-1,28	-9,6395163	5,4444E-22	7,0275E-21
YMR316W	1333,57	0,29	-1,8	-9,6393585	5,4528E-22	7,0275E-21
YLR012C	88,49	53,91	5,75	9,63086831	5,9227E-22	7,6173E-21
YMR062C	2988	2,99	1,58	9,6100426	7,2519E-22	9,3076E-21
YGR127W	908,96	0,29	-1,81	-9,60752369	7,4315E-22	9,5184E-21
YOR315W	995,24	0,28	-1,83	-9,60469677	7,6382E-22	9,7631E-21
YPL151C	1587,16	2,68	1,42	9,59723668	8,2116E-22	1,0474E-20
YMR191W	2459,51	2,93	1,55	9,59691241	8,2375E-22	1,0486E-20
YJL074C	2031,37	4,07	2,03	9,58455317	9,2859E-22	1,1796E-20
YIL063C	2631,77	2,6	1,38	9,58231776	9,4891E-22	1,203E-20
YOR043W	4643,24	2,4	1,26	9,56810289	1,0889E-21	1,3776E-20
YDL059C	711,88	3,08	1,62	9,55732925	1,2083E-21	1,5257E-20
YDR088C	1731,62	2,58	1,37	9,5555341	1,2295E-21	1,5492E-20

YOL132W	188,85	6,62	2,73	9,55453828	1,2414E-21	1,561E-20
YDR178W	2171,16	0,23	-2,12	-9,5462339	1,345E-21	1,6879E-20
YOR065W	3577,36	0,25	-2,02	-9,54254999	1,3936E-21	1,7454E-20
YKL029C	5432,44	0,37	-1,45	-9,54175966	1,4043E-21	1,7552E-20
YOL086C	397903,78	3,03	1,6	9,53637399	1,4791E-21	1,8451E-20
YLR297W	1306,89	2,84	1,51	9,52963725	1,5783E-21	1,9649E-20
YCR035C	2367,63	2,63	1,39	9,51787421	1,7676E-21	2,1917E-20
YML120C	4850,97	0,26	-1,94	-9,51790297	1,7671E-21	2,1917E-20
YLR168C	1592,38	2,74	1,45	9,50972136	1,9117E-21	2,3657E-20
YHR088W	2388,48	3,26	1,7	9,50680219	1,9661E-21	2,4282E-20
YNL238W	4334,41	2,86	1,52	9,50304974	2,0383E-21	2,5123E-20
YLR275W	914,69	3,16	1,66	9,49361719	2,2315E-21	2,745E-20
YER028C	734,24	3,1	1,63	9,48844299	2,3451E-21	2,879E-20
YOR239W	10063,78	2,58	1,37	9,48071395	2,5255E-21	3,0943E-20
YNL079C	17584,9	2,32	1,22	9,45480213	3,2363E-21	3,9575E-20
YHR207C	2192,25	2,49	1,32	9,44890005	3,4241E-21	4,1789E-20
YGR255C	2866,78	0,38	-1,41	-9,43078294	4,0704E-21	4,9579E-20
YPL026C	1758,05	3,3	1,72	9,4279228	4,1829E-21	5,085E-20
YLR257W	14750,65	0,43	-1,23	-9,42141579	4,4505E-21	5,3997E-20
YNR056C	942,8	2,91	1,54	9,41304726	4,8196E-21	5,8361E-20
YBR095C	1716,03	2,57	1,36	9,3937474	5,7903E-21	6,9979E-20
YKL085W	23636,31	3,48	1,8	9,39136217	5,9229E-21	7,1443E-20
YDR061W	3159,02	2,55	1,35	9,37385331	6,9932E-21	8,4189E-20
YLR345W	3513,34	0,37	-1,44	-9,37278924	7,0641E-21	8,4878E-20
YIL033C	6593,07	0,41	-1,29	-9,36108181	7,8924E-21	9,4648E-20
YPR018W	1529,87	2,68	1,42	9,36031822	7,9497E-21	9,5151E-20
YJL192C	3053,26	0,38	-1,38	-9,33839149	9,7809E-21	1,1684E-19
YDR033W	12484,98	0,43	-1,21	-9,33723497	9,8883E-21	1,179E-19
YIL015W	2697,75	0,38	-1,41	-9,33181926	1,0407E-20	1,2385E-19
YHR049W	3349,22	2,46	1,3	9,29977167	1,4075E-20	1,6717E-19
YLL029W	11646,6	2,54	1,35	9,27483691	1,7789E-20	2,1089E-19
YJL161W	696,21	0,15	-2,75	-9,25884271	2,0666E-20	2,4453E-19
YDR050C	124943,8	2,96	1,57	9,25845961	2,074E-20	2,4494E-19
YNL277W	1901,54	2,89	1,53	9,23408263	2,6051E-20	3,0707E-19
YLR223C	1950,89	2,97	1,57	9,22860167	2,7419E-20	3,2259E-19
YOR066W	934,4	0,33	-1,58	-9,22310092	2,8863E-20	3,3894E-19
YOR113W	1527,18	2,64	1,4	9,21639409	3,0726E-20	3,6013E-19
YDR146C	1577,11	0,38	-1,39	-9,19709835	3,6775E-20	4,3021E-19
YER160C	450,39	3,37	1,75	9,18791906	4,0051E-20	4,6767E-19
YBR026C	3119,26	0,38	-1,38	-9,18393217	4,1563E-20	4,8441E-19
YMR061W	4808,67	2,42	1,28	9,17633384	4,4602E-20	5,1885E-19
YDR096W	1897,56	0,4	-1,34	-9,17587905	4,479E-20	5,2007E-19
YOR081C	1858,52	0,4	-1,33	-9,17210082	4,6389E-20	5,3763E-19
YDR011W	9834,09	4,15	2,05	9,16010258	5,1848E-20	5,9978E-19
Q0020	81,52	40,99	5,36	9,15826717	5,2737E-20	6,0893E-19
YPL204W	8700,29	2,31	1,21	9,15345244	5,5142E-20	6,3552E-19
YOR181W	1989,86	0,39	-1,34	-9,12293389	7,3118E-20	8,4112E-19
YDL130W-A	3240,88	0,26	-1,94	-9,11964632	7,537E-20	8,6543E-19
YEL007W	3243,8	0,34	-1,54	-9,11467923	7,8904E-20	9,0433E-19
YML074C	20056,38	2,92	1,54	9,11052086	8,1987E-20	9,3795E-19

YMR160W	1304,83	0,37	-1,43	-9,10871805	8,3361E-20	9,5191E-19
YER089C	3997,39	0,43	-1,22	-9,10844291	8,3573E-20	9,5258E-19
YIL136W	6559,34	0,2	-2,35	-9,1077403	8,4116E-20	9,5701E-19
YBR169C	18545,04	2,45	1,29	9,1059317	8,5529E-20	9,7131E-19
YLR206W	5999,02	0,43	-1,21	-9,09554119	9,4117E-20	1,0669E-18
YGL178W	3170,82	3,25	1,7	9,094441	9,5074E-20	1,0758E-18
YKL160W	4762,22	2,3	1,2	9,091188	9,7963E-20	1,1064E-18
YPR020W	1345,12	0,36	-1,48	-9,09074707	9,8361E-20	1,1089E-18
YNR034W-A	5830,39	0,18	-2,51	-9,07499808	1,1368E-19	1,2793E-18
YHR039C-A	4704,42	2,3	1,2	9,05790229	1,3298E-19	1,4938E-18
YGR144W	190,3	6,92	2,79	9,0543678	1,3736E-19	1,5402E-18
YJR014W	5587,38	2,48	1,31	9,04941918	1,4373E-19	1,6087E-18
YGR049W	767,05	0,31	-1,67	-9,04512079	1,495E-19	1,6703E-18
YOL005C	1243,71	0,38	-1,41	-9,03914214	1,5791E-19	1,7611E-18
YIL034C	4587,51	0,41	-1,3	-9,03481106	1,6429E-19	1,8289E-18
YEL003W	1229,17	2,75	1,46	9,02824404	1,7445E-19	1,9386E-18
YPR094W	324,45	3,77	1,91	9,02224628	1,8427E-19	2,0441E-18
YHR082C	10910,81	2,87	1,52	9,01781829	1,9187E-19	2,1246E-18
YAL028W	999,75	0,31	-1,7	-9,01054217	2,0504E-19	2,2664E-18
YPL110C	2195,87	2,85	1,51	9,00700993	2,1175E-19	2,3364E-18
YKR103W	649,25	3,06	1,61	8,99868754	2,2843E-19	2,516E-18
YNL308C	5086,21	3,09	1,63	8,99134582	2,4422E-19	2,6851E-18
YGL037C	45083,15	2,83	1,5	8,98762986	2,5262E-19	2,7726E-18
YJR094W-A	4717,51	2,62	1,39	8,98242504	2,6486E-19	2,9018E-18
YFR032C	105,31	9,39	3,23	8,9797536	2,7137E-19	2,9679E-18
YLR106C	12380,68	3,18	1,67	8,97207116	2,9099E-19	3,1769E-18
YIR015W	959,46	2,78	1,47	8,96543282	3,0906E-19	3,3683E-18
YIL143C	3521,83	2,34	1,22	8,96446123	3,118E-19	3,3921E-18
YLR025W	4832,97	2,29	1,2	8,96211033	3,1852E-19	3,4592E-18
YLR241W	2223,25	0,35	-1,53	-8,95639402	3,3547E-19	3,6369E-18
YPR151C	645,52	8,62	3,11	8,95122165	3,5157E-19	3,8048E-18
YHR188C	3493,62	0,42	-1,25	-8,94372589	3,7627E-19	4,065E-18
YGR221C	701,07	0,33	-1,58	-8,93693675	4,0011E-19	4,315E-18
YLL051C	2632,71	2,35	1,23	8,93611374	4,031E-19	4,3398E-18
YNL145W	6643,01	0,29	-1,78	-8,92725767	4,367E-19	4,6934E-18
YCL050C	14745,5	2,77	1,47	8,92693591	4,3797E-19	4,6989E-18
YGL114W	2007,96	2,45	1,29	8,92474023	4,4675E-19	4,7848E-18
YHR006W	3092,81	2,32	1,21	8,91131169	5,0432E-19	5,3922E-18
YGR227W	827,63	0,33	-1,6	-8,90972251	5,116E-19	5,4606E-18
YIL124W	2148,6	0,26	-1,94	-8,90681716	5,2519E-19	5,596E-18
YHR039C	3231,66	0,4	-1,32	-8,9021288	5,4786E-19	5,8275E-18
YIL019W	1134,3	3,34	1,74	8,90070176	5,5495E-19	5,8929E-18
YHR048W	727,93	2,89	1,53	8,88802292	6,2206E-19	6,5942E-18
YLR299W	1702,3	0,35	-1,53	-8,88756154	6,2465E-19	6,6104E-18
YGR131W	272,32	4,15	2,05	8,87329283	7,1014E-19	7,5024E-18
YGR161W-B	78,49	13,74	3,78	8,85962104	8,0287E-19	8,4676E-18
YGL049C	3739,56	0,36	-1,49	-8,85744018	8,1873E-19	8,6202E-18
YCR030C	3117,07	0,4	-1,34	-8,85067581	8,6991E-19	9,1436E-18
YJL050W	5371,85	4,02	2,01	8,84650585	9,0302E-19	9,4756E-18
YOR329C	2586	0,41	-1,28	-8,8405716	9,523E-19	9,9759E-18

YER111C	858,45	0,34	-1,57	-8,83491394	1,0018E-18	1,0476E-17
YKR068C	2320,63	2,34	1,23	8,83416351	1,0085E-18	1,0529E-17
YOR150W	1306,31	2,97	1,57	8,8086054	1,2671E-18	1,3207E-17
YIL111W	3029,03	0,27	-1,87	-8,79417312	1,4411E-18	1,4995E-17
YHL027W	2320,69	0,41	-1,27	-8,78083496	1,6227E-18	1,6856E-17
YCR091W	1987,23	0,28	-1,84	-8,76942551	1,7958E-18	1,8624E-17
YJR048W	2235,2	3,75	1,91	8,75501838	2,0407E-18	2,1128E-17
YHR044C	422,64	3,47	1,8	8,75474525	2,0457E-18	2,1144E-17
YGR222W	800,54	0,34	-1,55	-8,7285775	2,579E-18	2,6568E-17
YLR203C	3008,58	0,35	-1,51	-8,72860947	2,5782E-18	2,6568E-17
YLR042C	517,49	3,2	1,68	8,72795165	2,5933E-18	2,6668E-17
YOL010W	796,96	2,75	1,46	8,72777687	2,5973E-18	2,6668E-17
YLR164W	1071,25	6,78	2,76	8,72178224	2,7386E-18	2,8073E-17
YDR179C	351,13	3,6	1,85	8,71614433	2,8784E-18	2,9457E-17
YGR268C	1104,94	0,38	-1,38	-8,7148441	2,9116E-18	2,9748E-17
YPR133C	3808,56	2,26	1,18	8,70683	3,1249E-18	3,1875E-17
YJL035C	485,79	3,53	1,82	8,69636401	3,4269E-18	3,4898E-17
YMR098C	1232,52	0,4	-1,34	-8,69603512	3,4368E-18	3,4942E-17
YNR030W	1488,93	0,38	-1,4	-8,69294218	3,5317E-18	3,5848E-17
YFR021W	1095,94	0,38	-1,38	-8,68829348	3,6792E-18	3,7285E-17
YDL216C	820,4	0,35	-1,5	-8,67315296	4,2032E-18	4,2525E-17
YCR097W	1985,89	3,25	1,7	8,66529004	4,5036E-18	4,5491E-17
YJR033C	1800,89	0,42	-1,26	-8,66329451	4,5832E-18	4,6219E-17
YCR061W	619,22	0,34	-1,57	-8,63061491	6,1023E-18	6,1438E-17
YPR068C	803,27	2,66	1,41	8,62243805	6,5543E-18	6,5883E-17
YEL017W	1233,95	0,37	-1,43	-8,60793906	7,4386E-18	7,465E-17
YDL082W	12966,78	0,4	-1,31	-8,60186906	7,8428E-18	7,858E-17
YNR074C	1661,94	2,37	1,25	8,59599718	8,2545E-18	8,2571E-17
YJL005W	3619,32	0,45	-1,16	-8,57539904	9,8744E-18	9,8617E-17
YFR041C	2542,55	2,3	1,2	8,5560796	1,1677E-17	1,1643E-16
YKL172W	4118,09	2,65	1,41	8,55337226	1,1954E-17	1,1901E-16
YPR009W	1181,28	2,58	1,37	8,55149064	1,2151E-17	1,2077E-16
YMR165C	1821,3	0,39	-1,36	-8,54631611	1,2708E-17	1,2611E-16
YBR207W	2918,57	2,43	1,28	8,53083048	1,453E-17	1,4373E-16
YDR215C	132,5	6,03	2,59	8,5309348	1,4517E-17	1,4373E-16
YOR056C	2130,2	2,55	1,35	8,53055331	1,4565E-17	1,4384E-16
YJL217W	2697,33	2,55	1,35	8,50981939	1,742E-17	1,7177E-16
YGR138C	1498,15	0,24	-2,03	-8,5075112	1,7771E-17	1,7495E-16
YGR213C	523,28	2,96	1,56	8,50670849	1,7894E-17	1,7588E-16
YDR122W	3171,39	0,41	-1,29	-8,48426201	2,1709E-17	2,1305E-16
YHL044W	327,36	0,23	-2,14	-8,47480732	2,3547E-17	2,3071E-16
YBR072W	60573,48	4,61	2,21	8,47423517	2,3663E-17	2,3149E-16
YDR001C	9456,6	0,47	-1,09	-8,4739317	2,3725E-17	2,3173E-16
YGR077C	1218,73	0,36	-1,46	-8,4686281	2,483E-17	2,4214E-16
YNL101W	2808,65	2,32	1,21	8,46663236	2,5259E-17	2,4594E-16
Q0130	82,55	21,19	4,41	8,4621683	2,6245E-17	2,5514E-16
YLR390W-A	8569,97	0,46	-1,12	-8,45990011	2,6761E-17	2,5975E-16
YJL029C	1798,23	0,43	-1,23	-8,45346646	2,8278E-17	2,7404E-16
YPR073C	2674,16	2,3	1,2	8,45256082	2,8498E-17	2,7575E-16
YOR247W	7959,03	0,47	-1,1	-8,44815174	2,9595E-17	2,8592E-16

YLR089C	3357,01	0,45	-1,15	-8,44340028	3,0824E-17	2,9732E-16
YCL005W-A	1716,99	2,31	1,21	8,44081927	3,1512E-17	3,0343E-16
YDR307W	1306,21	0,34	-1,55	-8,44066207	3,1555E-17	3,0343E-16
YOL025W	606,12	0,34	-1,57	-8,43000313	3,4566E-17	3,3187E-16
YDL206W	966,45	0,37	-1,42	-8,42672201	3,5548E-17	3,4078E-16
YLL001W	1917,38	0,42	-1,25	-8,42038277	3,7526E-17	3,5888E-16
YPR127W	6864,53	4,13	2,05	8,42030127	3,7552E-17	3,5888E-16
YDR533C	3269,37	2,57	1,36	8,41763994	3,8414E-17	3,6656E-16
YDL153C	5909,23	3,73	1,9	8,40607191	4,2397E-17	4,0394E-16
YPL145C	5769,7	0,41	-1,27	-8,39551141	4,6387E-17	4,4128E-16
YPL156C	1641,23	2,32	1,22	8,39359386	4,715E-17	4,4786E-16
YLR356W	1135,73	0,27	-1,88	-8,38702083	4,9862E-17	4,7289E-16
YDR523C	136,51	5,67	2,5	8,38499153	5,0729E-17	4,8038E-16
YEL039C	3806,41	0,22	-2,16	-8,38345355	5,1397E-17	4,8596E-16
YCR010C	262,36	4,34	2,12	8,38265452	5,1747E-17	4,8853E-16
YER065C	1434,99	2,75	1,46	8,36673455	5,9237E-17	5,5839E-16
YIR007W	1197,2	0,4	-1,33	-8,36475035	6,0242E-17	5,6701E-16
YKR023W	1595,45	2,45	1,3	8,36440428	6,0419E-17	5,6781E-16
YDR539W	3213,53	2,37	1,24	8,36111133	6,213E-17	5,8301E-16
YCR020C-A	704,6	2,74	1,45	8,35546128	6,5178E-17	6,1068E-16
YNL014W	969,05	3,23	1,69	8,35166643	6,7307E-17	6,2968E-16
YBR013C	379,89	0,29	-1,77	-8,33237236	7,9238E-17	7,4019E-16
YOR067C	954,33	0,36	-1,48	-8,32862635	8,1786E-17	7,6284E-16
YJL158C	13580,3	0,47	-1,1	-8,32534044	8,4087E-17	7,8313E-16
YJL002C	5539,49	0,46	-1,12	-8,31672572	9,0427E-17	8,4091E-16
YOL103W-B	628,62	2,71	1,44	8,31607153	9,0927E-17	8,443E-16
YGL098W	1599,96	2,52	1,33	8,31287719	9,3409E-17	8,6606E-16
YIR018C-A	221,24	4,15	2,05	8,30786864	9,7436E-17	9,0205E-16
YHR157W	225,88	6,93	2,79	8,30697499	9,8173E-17	9,0751E-16
YJR056C	1002,7	2,45	1,29	8,2999211	1,0418E-16	9,6162E-16
YLR110C	138087,58	0,43	-1,22	-8,29906571	1,0493E-16	9,6713E-16
YNR034W	1765,9	2,3	1,2	8,29311628	1,1032E-16	1,0153E-15
YKL091C	2349,35	0,26	-1,93	-8,29182786	1,1152E-16	1,0248E-15
YCL059C	3701,23	2,74	1,45	8,28786721	1,153E-16	1,0579E-15
YHR214C-B	2724,57	2,38	1,25	8,27644545	1,2691E-16	1,1627E-15
YML034W	4473,49	2,13	1,09	8,27250147	1,3118E-16	1,2001E-15
YDL018C	574,82	0,34	-1,57	-8,27107258	1,3276E-16	1,2128E-15
YNR073C	286,15	3,54	1,83	8,25487125	1,5207E-16	1,3871E-15
YPR156C	2283,45	0,39	-1,37	-8,2532697	1,5412E-16	1,4038E-15
YDR183W	765,79	2,58	1,37	8,25011195	1,5825E-16	1,4393E-15
YPL123C	1954,44	0,4	-1,31	-8,24730888	1,62E-16	1,4713E-15
YER099C	1383,94	0,41	-1,3	-8,2432575	1,6758E-16	1,5198E-15
YBL100W-B	805,25	2,75	1,46	8,23748588	1,7587E-16	1,5925E-15
YBR196C	77283,84	2,03	1,02	8,23035722	1,8666E-16	1,6853E-15
YDR388W	6976,03	0,44	-1,17	-8,23042863	1,8654E-16	1,6853E-15
YGR169C-A	1832,91	2,3	1,2	8,22845895	1,8964E-16	1,7098E-15
YDR342C	2997,86	0,25	-2,02	-8,22785688	1,9059E-16	1,7159E-15
YGL215W	4166,86	2,38	1,25	8,22663487	1,9255E-16	1,731E-15
YIL113W	768,36	0,32	-1,62	-8,22631931	1,9305E-16	1,733E-15
YBR238C	1172,43	3,68	1,88	8,2222572	1,9971E-16	1,7902E-15

YLR250W	3934,15	2,15	1,11	8,21947317	2,044E-16	1,8296E-15
YDR173C	901,72	2,77	1,47	8,2176605	2,0751E-16	1,8548E-15
YIL138C	3589,79	2,14	1,1	8,2152265	2,1176E-16	1,8901E-15
YLR063W	439,35	3,05	1,61	8,21144937	2,1853E-16	1,9477E-15
YGR260W	3208,04	0,41	-1,28	-8,21020128	2,2082E-16	1,9652E-15
YMR302C	2635,22	0,44	-1,19	-8,19752193	2,4539E-16	2,1808E-15
YMR027W	8767,39	2,07	1,05	8,1884727	2,6456E-16	2,3478E-15
YKL073W	5213,18	3,34	1,74	8,18116788	2,8111E-16	2,4911E-15
YKR013W	3688,5	0,46	-1,12	-8,17985137	2,8419E-16	2,5113E-15
YML057W	3245,52	0,46	-1,12	-8,17991016	2,8406E-16	2,5113E-15
YIL123W	3638,34	0,39	-1,37	-8,17171649	3,0403E-16	2,6827E-15
YGL030W	16752,65	2,13	1,09	8,1640758	3,2391E-16	2,854E-15
YNL227C	1438,02	2,53	1,34	8,15769415	3,4148E-16	3,0046E-15
YOL109W	15845,37	2,08	1,06	8,1551922	3,4862E-16	3,0632E-15
YER143W	3304,6	2,13	1,09	8,15090197	3,6122E-16	3,1693E-15
YJR001W	2231,01	0,41	-1,27	-8,14466478	3,8033E-16	3,3323E-15
YBL029W	687,84	0,37	-1,43	-8,14204103	3,8867E-16	3,4006E-15
YNL031C	15862,48	0,49	-1,03	-8,13291453	4,1909E-16	3,6616E-15
YOR063W	97498,63	2,21	1,14	8,12736655	4,3872E-16	3,8277E-15
YGL006W	4475,06	0,37	-1,44	-8,12266914	4,5604E-16	3,9733E-15
YOR054C	5003,92	2,15	1,11	8,12117081	4,6171E-16	4,017E-15
YLR049C	1093,93	0,39	-1,38	-8,11307401	4,9355E-16	4,288E-15
YJR022W	302,31	3,39	1,76	8,10509059	5,2706E-16	4,5728E-15
YOL158C	6315,36	2,47	1,31	8,09142607	5,897E-16	5,1091E-15
YPL084W	3018,27	0,4	-1,33	-8,0866556	6,1325E-16	5,3058E-15
YHR072W	1764,38	0,4	-1,33	-8,08042779	6,454E-16	5,5761E-15
YER086W	4617,67	0,45	-1,16	-8,07976771	6,489E-16	5,5986E-15
YDR026C	2083,18	2,29	1,19	8,07220186	6,9042E-16	5,9485E-15
YPR024W	5800,03	2,14	1,1	8,06893685	7,0913E-16	6,1013E-15
YMR215W	2401,45	0,46	-1,13	-8,06171339	7,5232E-16	6,464E-15
YLL027W	2580,33	2,34	1,23	8,06128738	7,5495E-16	6,4776E-15
YER075C	900,58	2,7	1,43	8,06083026	7,5778E-16	6,4929E-15
YGL032C	1827,6	0,28	-1,82	-8,06005231	7,6262E-16	6,5253E-15
YNR008W	1761,34	0,36	-1,46	-8,04965722	8,3026E-16	7,0943E-15
YGL045W	2145,02	2,56	1,35	8,03700801	9,2059E-16	7,8553E-15
YLL019C	2270,02	2,19	1,13	8,03540863	9,3268E-16	7,9476E-15
YMR240C	1097,4	2,42	1,28	8,03095924	9,6713E-16	8,2299E-15
YER185W	152,28	4,95	2,31	8,0205317	1,0529E-15	8,9474E-15
YGL005C	2441,47	2,42	1,28	8,01298174	1,1196E-15	9,5013E-15
YPL091W	9672,43	2,04	1,03	8,00200639	1,2241E-15	1,0374E-14
YMR269W	665,73	2,68	1,42	7,99392878	1,3071E-15	1,1062E-14
YDR350C	1817,72	2,26	1,17	7,9869332	1,3834E-15	1,1692E-14
YLR074C	1857,09	2,2	1,14	7,9718337	1,5634E-15	1,3195E-14
YCL036W	355,5	3,24	1,7	7,96455586	1,6582E-15	1,3976E-14
YER169W	1408,91	0,41	-1,3	-7,96146277	1,7002E-15	1,4311E-14
YLR259C	26036,2	2,07	1,05	7,96109885	1,7052E-15	1,4334E-14
YJL121C	2484,15	0,45	-1,15	-7,96012118	1,7187E-15	1,4428E-14
YAL001C	3776,61	2,08	1,06	7,95327842	1,8164E-15	1,5227E-14
YBR283C	5642,48	0,47	-1,08	-7,94124601	2,0016E-15	1,6757E-14
YBR139W	4496,67	0,38	-1,39	-7,9404858	2,0139E-15	1,6838E-14

YML118W	501,93	0,33	-1,6	-7,92949829	2,2003E-15	1,8372E-14
YER001W	1460,45	0,35	-1,5	-7,91388106	2,4949E-15	2,0803E-14
YLR353W	913,72	0,4	-1,32	-7,90471479	2,6855E-15	2,2362E-14
YNCP0005C	110,94	7	2,81	7,90119397	2,7624E-15	2,2972E-14
YCL010C	1334,97	2,31	1,21	7,89735925	2,8487E-15	2,3658E-14
YBL047C	7389,99	0,45	-1,16	-7,89385432	2,9299E-15	2,43E-14
YHL010C	754,58	0,38	-1,39	-7,89344915	2,9395E-15	2,4347E-14
YDR490C	1180	0,39	-1,34	-7,89061196	3,0071E-15	2,4874E-14
YGR112W	902,01	0,38	-1,4	-7,88547398	3,1334E-15	2,5884E-14
YDL125C	4471,84	2,46	1,3	7,88463139	3,1547E-15	2,6025E-14
YJR045C	56618,15	2,18	1,12	7,88277093	3,202E-15	2,638E-14
YBR003W	1705,33	0,41	-1,3	-7,87870485	3,3079E-15	2,7181E-14
YOR010C	492,01	2,79	1,48	7,87881597	3,305E-15	2,7181E-14
YGL142C	837,07	0,38	-1,38	-7,87732351	3,3447E-15	2,7447E-14
YLR192C	8583,47	2,12	1,08	7,87116376	3,5136E-15	2,8795E-14
YNCB0003W	107,86	7,16	2,84	7,86647081	3,6479E-15	2,9856E-14
YIL107C	5151,64	2,41	1,27	7,85822248	3,8962E-15	3,1847E-14
YDL022W	11059,25	0,49	-1,03	-7,84856583	4,2082E-15	3,4351E-14
YER162C	2057,47	2,18	1,13	7,83958919	4,5202E-15	3,685E-14
YAL038W	450122,55	2,02	1,01	7,83778975	4,5855E-15	3,7333E-14
YEL061C	1689,77	2,42	1,27	7,82742574	4,9796E-15	4,0489E-14
YKR104W	216,69	4,03	2,01	7,82161066	5,2152E-15	4,2349E-14
YNL115C	2115,59	0,37	-1,45	-7,81714608	5,4034E-15	4,382E-14
YNR045W	1208,56	0,35	-1,51	-7,81028419	5,7059E-15	4,6213E-14
YGL115W	2486,13	0,47	-1,1	-7,79710768	6,3342E-15	5,1235E-14
YDR195W	1640,28	2,63	1,39	7,78819399	6,7974E-15	5,4909E-14
YMR279C	563,71	0,34	-1,54	-7,78481601	6,9815E-15	5,6323E-14
YNL062C	1455,46	2,59	1,37	7,78216719	7,1293E-15	5,7441E-14
YOR192C-C	194,43	4,46	2,16	7,77518541	7,5337E-15	6,0621E-14
YOR052C	3672,02	0,46	-1,14	-7,77168115	7,7451E-15	6,2242E-14
YMR300C	3917,27	0,37	-1,45	-7,75999867	8,493E-15	6,8164E-14
YFR031C	2784,97	2,42	1,27	7,75138046	9,0899E-15	7,286E-14
YKR090W	1252,65	0,41	-1,3	-7,74506284	9,5535E-15	7,6477E-14
YPR148C	7364,63	2,13	1,09	7,74190135	9,7941E-15	7,8302E-14
YHL001W	6533,09	2,32	1,21	7,73313467	1,0493E-14	8,3782E-14
YIL026C	3068,24	2,45	1,29	7,73126166	1,0649E-14	8,4915E-14
YGR174C	1257,52	0,31	-1,7	-7,72808903	1,0917E-14	8,6946E-14
YNL108C	1535,56	2,5	1,32	7,72694798	1,1016E-14	8,7617E-14
YBR241C	1071,34	0,24	-2,06	-7,71661051	1,1946E-14	9,4899E-14
YLL018C	27304,13	2	1	7,71410667	1,2183E-14	9,6657E-14
YBR054W	3816,22	0,43	-1,21	-7,70431278	1,3155E-14	1,0423E-13
YLR075W	54166,87	1,96	0,97	7,69866538	1,3749E-14	1,088E-13
YER031C	2406,66	0,47	-1,09	-7,69806985	1,3814E-14	1,0917E-13
YMR185W	807,7	2,47	1,31	7,69450044	1,4205E-14	1,1212E-13
YDR377W	2598,39	2,59	1,37	7,69424764	1,4233E-14	1,122E-13
YBL069W	1319,95	0,43	-1,23	-7,69369724	1,4294E-14	1,1254E-13
YER119C	1524,37	0,36	-1,49	-7,6849516	1,5305E-14	1,2035E-13
YPR091C	2752,15	0,46	-1,13	-7,68397456	1,5423E-14	1,2112E-13
YBL058W	4418,67	0,48	-1,05	-7,68020748	1,5883E-14	1,2458E-13
YGL149W	82,52	9,42	3,24	7,67929245	1,5997E-14	1,2531E-13

YBR080C	3810,89	0,42	-1,24	-7,6734202	1,6747E-14	1,3102E-13
YHR060W	1238,39	2,35	1,23	7,66719783	1,7579E-14	1,3736E-13
YKL028W	2818,95	2,09	1,06	7,66462196	1,7936E-14	1,3997E-13
YNR055C	1985,04	0,46	-1,13	-7,65929176	1,8696E-14	1,4572E-13
YNL260C	970,66	2,3	1,2	7,65730995	1,8987E-14	1,478E-13
YGL058W	2142,95	2,37	1,24	7,6569178	1,9045E-14	1,4807E-13
YKL006C-A	856,35	2,51	1,33	7,65286735	1,9655E-14	1,5262E-13
YCL021W-A	755,81	2,98	1,58	7,6378059	2,2095E-14	1,7136E-13
YKL204W	5033,51	2,01	1	7,63248789	2,3027E-14	1,7836E-13
YBL005W	3103,6	2,19	1,13	7,61758932	2,5846E-14	1,9965E-13
YBR067C	8986,07	0,48	-1,07	-7,61773155	2,5817E-14	1,9965E-13
YBR183W	1691,79	0,33	-1,59	-7,61745828	2,5872E-14	1,9965E-13
YGR280C	2338,16	2,2	1,14	7,6154038	2,6287E-14	2,026E-13
YKR042W	30374,17	2,53	1,34	7,60556939	2,8365E-14	2,1835E-13
YNL310C	714,98	0,39	-1,35	-7,5979732	3,008E-14	2,3126E-13
YBR047W	682,61	3,2	1,68	7,59428331	3,095E-14	2,3765E-13
YPL011C	1588,64	2,17	1,12	7,58836394	3,2397E-14	2,4846E-13
YDR135C	6888,07	2,22	1,15	7,58482221	3,3294E-14	2,5502E-13
YMR261C	5612,64	0,47	-1,08	-7,58446458	3,3386E-14	2,5541E-13
YML058W-A	264,91	4,89	2,29	7,58310832	3,3737E-14	2,5778E-13
YFR024C-A	7066,64	1,97	0,98	7,58276965	3,3826E-14	2,5814E-13
YPL186C	1811,52	0,26	-1,95	-7,58109991	3,4264E-14	2,6116E-13
YLR036C	1162,26	2,28	1,19	7,57517796	3,5864E-14	2,7302E-13
YHR018C	5368,73	2	1	7,57388957	3,6221E-14	2,7541E-13
YKL015W	2036,88	2,44	1,28	7,57081622	3,7089E-14	2,8166E-13
YJL098W	1472,19	2,96	1,56	7,56970981	3,7406E-14	2,8372E-13
YNCK0015C	170,86	4,22	2,08	7,56753366	3,8038E-14	2,8816E-13
YER177W	47458,11	2,1	1,07	7,56506517	3,8767E-14	2,9333E-13
YOR209C	5560,24	0,44	-1,19	-7,56385137	3,9131E-14	2,9572E-13
YOR006C	2187,86	2,25	1,17	7,56363074	3,9197E-14	2,9586E-13
YPR025C	2143,24	2,25	1,17	7,5594949	4,0464E-14	3,0505E-13
YDR097C	2283,58	3,38	1,76	7,55827806	4,0844E-14	3,0754E-13
YBR001C	2278,17	0,37	-1,45	-7,55796832	4,0941E-14	3,079E-13
YMR291W	3322,2	2,03	1,02	7,55699431	4,1249E-14	3,0984E-13
YGL252C	5080,3	2,11	1,08	7,55101016	4,319E-14	3,2403E-13
YLL041C	3511,22	0,35	-1,53	-7,54841677	4,4058E-14	3,3014E-13
YJL031C	2332,71	2,07	1,05	7,54363096	4,5706E-14	3,4208E-13
YDR055W	5206,82	0,5	-1,01	-7,54058183	4,6788E-14	3,4976E-13
YKL016C	3880,14	0,34	-1,55	-7,53975889	4,7084E-14	3,5155E-13
YMR255W	2425,02	2,22	1,15	7,53735724	4,7959E-14	3,5765E-13
YMR133W	496,11	0,24	-2,08	-7,53595766	4,8476E-14	3,6107E-13
YGL122C	5098,5	2,02	1,01	7,52832555	5,1395E-14	3,8224E-13
YLR183C	837,57	0,39	-1,35	-7,52820934	5,1441E-14	3,8224E-13
YNL309W	517,91	0,36	-1,48	-7,5273867	5,1766E-14	3,8419E-13
YLR112W	404,24	2,91	1,54	7,52452956	5,291E-14	3,9222E-13
YGR070W	2260,56	0,47	-1,08	-7,52383892	5,3191E-14	3,9383E-13
YBL050W	4839,8	2,01	1,01	7,50923572	5,9474E-14	4,3982E-13
YPL225W	5998,55	1,99	0,99	7,50237308	6,2673E-14	4,6293E-13
YPL143W	17188,96	0,52	-0,96	-7,50183758	6,2929E-14	4,6427E-13
YDL195W	8652,51	0,41	-1,3	-7,50151551	6,3084E-14	4,6486E-13

YNCM0031W	928,54	5	2,32	7,49470288	6,6449E-14	4,8908E-13
YOR215C	2174,29	0,38	-1,38	-7,4909657	6,8369E-14	5,0261E-13
YBR037C	1011,42	0,32	-1,62	-7,48662369	7,0668E-14	5,189E-13
YDR216W	3690	2,2	1,14	7,48547071	7,1291E-14	5,2286E-13
YPL006W	1758,98	0,45	-1,14	-7,48506113	7,1514E-14	5,2387E-13
YGL234W	16280,74	0,36	-1,48	-7,46718908	8,1926E-14	5,9944E-13
YBR126C	21605,19	0,41	-1,28	-7,46473277	8,3469E-14	6,1001E-13
YDL079C	2214,05	0,21	-2,25	-7,45849333	8,7517E-14	6,3824E-13
YDR227W	1840,62	2,16	1,11	7,45846335	8,7537E-14	6,3824E-13
YLR240W	1144,77	0,43	-1,21	-7,44936991	9,3787E-14	6,8301E-13
YAL056W	3508,7	2,23	1,16	7,44738934	9,5205E-14	6,9252E-13
YER054C	2451,99	0,34	-1,54	-7,44448169	9,7326E-14	7,0712E-13
YER063W	2149,48	0,46	-1,14	-7,44165047	9,9435E-14	7,216E-13
YPL268W	2390,72	2,16	1,11	7,43992924	1,0074E-13	7,3021E-13
YKL079W	2504,77	2,05	1,03	7,43754353	1,0257E-13	7,4265E-13
YDR506C	596,09	0,37	-1,43	-7,43535434	1,0429E-13	7,5418E-13
YBL078C	2490,02	2,56	1,36	7,43420939	1,052E-13	7,5985E-13
YPL127C	2407,15	0,43	-1,22	-7,42815158	1,1013E-13	7,9454E-13
YCL051W	1523,19	0,46	-1,11	-7,42776165	1,1045E-13	7,9596E-13
YMR014W	1513,14	2,46	1,3	7,42322944	1,143E-13	8,2274E-13
YKL096W	3246,16	0,47	-1,1	-7,4227088	1,1475E-13	8,2502E-13
YGR023W	1830,18	2,21	1,14	7,4215385	1,1577E-13	8,3139E-13
YPL015C	2298,48	0,49	-1,04	-7,42044094	1,1673E-13	8,3734E-13
YPR194C	698,93	0,28	-1,86	-7,41943135	1,1762E-13	8,4277E-13
YPR158C-D	3600,19	1,99	0,99	7,41888414	1,1811E-13	8,4528E-13
YOR337W	654,02	2,49	1,31	7,4184405	1,1851E-13	8,4714E-13
YDR092W	2752,16	2,02	1,01	7,4174156	1,1943E-13	8,5274E-13
YNR051C	7631,28	2,52	1,33	7,41519845	1,2144E-13	8,6613E-13
YIR031C	1058,04	2,88	1,53	7,40875331	1,2749E-13	9,0824E-13
YLL024C	91938,67	3,8	1,93	7,40276474	1,3338E-13	9,4908E-13
YDR131C	1482,98	2,12	1,08	7,39635538	1,3997E-13	9,9487E-13
YDL188C	1702,6	0,45	-1,14	-7,39406213	1,4241E-13	1,011E-12
YER134C	1498,59	0,44	-1,19	-7,39357449	1,4293E-13	1,0136E-12
YKR095W	8442,48	2,4	1,26	7,38844007	1,4856E-13	1,0523E-12
YEL060C	9016,84	0,51	-0,96	-7,38616457	1,5112E-13	1,0692E-12
YER059W	1877,04	0,47	-1,09	-7,38227579	1,5561E-13	1,0997E-12
YMR313C	1398,71	0,43	-1,21	-7,38149539	1,5652E-13	1,1049E-12
YDR285W	721,42	2,38	1,25	7,37693798	1,6197E-13	1,1421E-12
YOL031C	2336,64	2,51	1,33	7,37385244	1,6577E-13	1,1675E-12
YDR464W	2820,01	2,19	1,13	7,36855296	1,7249E-13	1,2135E-12
YOL040C	27869,03	0,5	-1	-7,3660754	1,7572E-13	1,2348E-12
YLR225C	3510,09	2,46	1,3	7,35591111	1,8963E-13	1,331E-12
YOR149C	859,42	0,43	-1,21	-7,3493905	1,9911E-13	1,396E-12
YML016C	2770,23	2,02	1,01	7,34384784	2,0754E-13	1,4535E-12
YOR078W	937,53	2,38	1,25	7,34258654	2,095E-13	1,4656E-12
YMR086W	3016,32	0,49	-1,03	-7,33749921	2,1762E-13	1,5207E-12
YJL145W	3125,92	2,26	1,17	7,31456956	2,5821E-13	1,8022E-12
YOR051C	5244,73	2,23	1,16	7,31013078	2,6688E-13	1,8607E-12
YNL128W	291,67	3,42	1,77	7,30710777	2,7295E-13	1,9009E-12
YGL180W	1350,75	2,43	1,28	7,30362428	2,8012E-13	1,9486E-12

YKR099W	829,06	2,35	1,23	7,30259305	2,8227E-13	1,9614E-12
YOR087W	1329,18	0,46	-1,12	-7,30053125	2,8663E-13	1,9895E-12
YDL019C	3614,39	0,5	-0,99	-7,29929479	2,8928E-13	2,0056E-12
YOR231W	2807,29	2,05	1,03	7,29805766	2,9195E-13	2,0219E-12
YDL115C	2434,31	2,09	1,06	7,29738456	2,9342E-13	2,0297E-12
YOR018W	3130,51	2,24	1,16	7,29570827	2,9709E-13	2,0529E-12
YLR394W	544,43	0,37	-1,43	-7,29453367	2,997E-13	2,0686E-12
YDR021W	468,39	2,59	1,37	7,29405683	3,0076E-13	2,0736E-12
YMR081C	4133,85	0,21	-2,26	-7,29302822	3,0306E-13	2,0872E-12
YOR119C	1317,73	2,16	1,11	7,29065536	3,0845E-13	2,1219E-12
YOL003C	844,47	0,43	-1,21	-7,28596562	3,1937E-13	2,1946E-12
YML117W	7183,32	0,51	-0,97	-7,28107624	3,3117E-13	2,2732E-12
YLL028W	2856,46	3,05	1,61	7,27557943	3,4494E-13	2,3651E-12
YDR163W	1226,86	2,13	1,09	7,27220261	3,5367E-13	2,4223E-12
YPR143W	1197,93	2,23	1,15	7,26998811	3,5952E-13	2,4596E-12
YDR251W	4373,42	0,49	-1,04	-7,26789951	3,6512E-13	2,4952E-12
YJL190C	16038,94	0,5	-1	-7,26209816	3,8113E-13	2,6017E-12
YBR229C	1886,48	0,42	-1,24	-7,26155755	3,8266E-13	2,6093E-12
YLL005C	559,08	0,37	-1,42	-7,25649281	3,9726E-13	2,7059E-12
YLR433C	1385,38	0,44	-1,18	-7,25521905	4,0101E-13	2,7285E-12
YBR015C	3237,44	0,48	-1,06	-7,24601713	4,2921E-13	2,9151E-12
YPR175W	1265,51	2,47	1,3	7,24596403	4,2937E-13	2,9151E-12
YNL315C	1942,94	0,42	-1,24	-7,24088019	4,4578E-13	3,0231E-12
YOR356W	1547,44	0,44	-1,18	-7,23603002	4,6201E-13	3,1298E-12
YBR297W	1161,85	2,88	1,53	7,2342135	4,6823E-13	3,1685E-12
YKL138C-A	662,66	2,52	1,33	7,23217227	4,7533E-13	3,213E-12
YBR060C	1875,94	2,06	1,04	7,2273782	4,9241E-13	3,3248E-12
YOL007C	898,25	0,37	-1,43	-7,22259608	5,1004E-13	3,4402E-12
YAR009C	1201,89	2,3	1,2	7,21510248	5,3893E-13	3,6311E-12
YER002W	3505,57	2,56	1,36	7,20936303	5,6214E-13	3,7833E-12
YDR044W	2861,02	0,47	-1,09	-7,2069368	5,7225E-13	3,8472E-12
YPL203W	3064,95	0,44	-1,18	-7,20436245	5,8316E-13	3,9163E-12
YNCC0011W	124,72	5,15	2,37	7,20399747	5,8472E-13	3,9226E-12
YER180C-A	223,38	3,32	1,73	7,20286526	5,896E-13	3,951E-12
YBR120C	1193,7	2,61	1,39	7,19352438	6,314E-13	4,2266E-12
YOR014W	4595,86	0,47	-1,09	-7,19244101	6,3643E-13	4,2557E-12
YCL052C	2483,93	0,48	-1,07	-7,18993876	6,482E-13	4,3297E-12
YJL172W	2241,06	0,49	-1,03	-7,18440936	6,7498E-13	4,5038E-12
YOR269W	591,61	2,52	1,33	7,17488781	7,2367E-13	4,8234E-12
YAR015W	12485,84	0,23	-2,11	-7,166281	7,7063E-13	5,1309E-12
YJR080C	981,76	0,37	-1,42	-7,16555471	7,7472E-13	5,1526E-12
YOL018C	1734,94	2,06	1,04	7,16406779	7,8318E-13	5,2033E-12
YGR205W	1373,02	0,38	-1,39	-7,16251135	7,9212E-13	5,2571E-12
YGR026W	4480,11	0,52	-0,95	-7,15795284	8,1891E-13	5,4291E-12
YBL030C	7519,97	0,42	-1,25	-7,15551834	8,3357E-13	5,5204E-12
YFR016C	7591,41	1,91	0,94	7,14567079	8,9557E-13	5,9247E-12
YOR343W-B	838,29	2,33	1,22	7,14255666	9,1611E-13	6,0541E-12
YKL152C	303454,63	2,67	1,42	7,13995719	9,336E-13	6,158E-12
YOL140W	614,55	2,39	1,26	7,13992604	9,3381E-13	6,158E-12
YBL016W	1692,92	0,41	-1,28	-7,1253343	1,0383E-12	6,8324E-12

YLR327C	36928,79	3,34	1,74	7,12534311	1,0382E-12	6,8324E-12
YGL162W	517,05	2,49	1,31	7,12447381	1,0448E-12	6,868E-12
YBR239C	778,94	2,4	1,26	7,11904949	1,0867E-12	7,1328E-12
YNL075W	1621,17	2,53	1,34	7,11896931	1,0874E-12	7,1328E-12
YOR123C	6259,8	1,99	1	7,11557304	1,1145E-12	7,303E-12
YGR180C	25711,04	1,9	0,92	7,11475905	1,1211E-12	7,3384E-12
YIR013C	67,38	9,69	3,28	7,10278818	1,2226E-12	7,9948E-12
YOL064C	2814,3	2,22	1,15	7,10196254	1,23E-12	8,0343E-12
YNCH0012W	131,74	5,2	2,38	7,09605355	1,2837E-12	8,3764E-12
YDL248W	458,69	0,37	-1,44	-7,09428458	1,3002E-12	8,4753E-12
YNCA0002W	86,97	1468,02	10,52	7,09216852	1,3203E-12	8,597E-12
YLR066W	1460,61	2,49	1,32	7,07451062	1,4998E-12	9,7556E-12
YGL036W	5206,99	1,92	0,94	7,07340167	1,5118E-12	9,8236E-12
YHR181W	834,56	0,45	-1,17	-7,07244702	1,5223E-12	9,8811E-12
YDL039C	4212,32	19,61	4,29	7,07227101	1,5242E-12	9,8833E-12
YJL019W	720,27	0,4	-1,33	-7,07187456	1,5285E-12	9,9013E-12
YPL049C	3739,78	0,52	-0,95	-7,0697703	1,5519E-12	1,0042E-11
YIL047C	2069,01	2,39	1,26	7,06447826	1,6122E-12	1,0421E-11
YJR130C	1867,63	2,06	1,05	7,06007685	1,6641E-12	1,0746E-11
YER020W	3306,2	0,48	-1,05	-7,05522616	1,7232E-12	1,1116E-11
YHR216W	9137,99	2,68	1,42	7,04352326	1,8744E-12	1,2079E-11
YJR049C	3129,9	2	1	7,0383269	1,9456E-12	1,2525E-11
YNL092W	426,98	0,35	-1,53	-7,03754648	1,9565E-12	1,2569E-11
YOR109W	2527,49	0,44	-1,2	-7,03761689	1,9556E-12	1,2569E-11
YMR278W	1490,15	0,46	-1,13	-7,03517721	1,9901E-12	1,2771E-11
YNCL0046W	783,42	2,28	1,19	7,03304045	2,0208E-12	1,2955E-11
YPL230W	2802,03	0,24	-2,06	-7,02786494	2,0972E-12	1,3431E-11
YMR237W	4003,9	0,5	-1,01	-7,01863717	2,2404E-12	1,4333E-11
YCL057W	4261,23	0,46	-1,13	-7,01780516	2,2538E-12	1,4404E-11
YGR038W	1286,15	0,47	-1,09	-7,01730865	2,2618E-12	1,444E-11
YGR118W	16522,81	2,13	1,09	7,01266001	2,3383E-12	1,4913E-11
YHL002W	3970,62	2,03	1,02	7,01072293	2,3709E-12	1,5106E-11
YFL034C-B	1968,5	2	1	7,00314094	2,5029E-12	1,593E-11
YGR207C	3361,15	2,19	1,13	7,00211564	2,5213E-12	1,6031E-11
YLR054C	228,74	0,28	-1,83	-6,99711843	2,6128E-12	1,6596E-11
YMR318C	6834,18	1,9	0,93	6,99611082	2,6317E-12	1,6699E-11
YNR014W	1534,49	0,38	-1,38	-6,99102821	2,7288E-12	1,7297E-11
YLL061W	1186,59	2,46	1,3	6,9863273	2,8218E-12	1,7868E-11
YER006W	5453,69	2,56	1,36	6,98602958	2,8277E-12	1,7888E-11
YNL327W	7831,1	0,49	-1,03	-6,98558346	2,8367E-12	1,7927E-11
YNCO0028W	181,09	5,28	2,4	6,981588	2,9186E-12	1,8425E-11
YLR023C	1959,22	2	1	6,97233326	3,1173E-12	1,9659E-11
YGL208W	1108,41	0,38	-1,4	-6,97049649	3,1582E-12	1,9898E-11
YKR067W	4595,92	2,17	1,12	6,96879786	3,1966E-12	2,0119E-11
YGR125W	1391,6	0,48	-1,05	-6,96814594	3,2115E-12	2,019E-11
YKL007W	4359,4	0,52	-0,93	-6,96801975	3,2143E-12	2,019E-11
YMR187C	726,53	2,52	1,33	6,96194603	3,356E-12	2,1058E-11
YGR186W	6646,3	1,91	0,94	6,96016883	3,3987E-12	2,1304E-11
YLR410W-B	1166,11	2,79	1,48	6,95626737	3,4941E-12	2,188E-11
YML035C	2614,16	0,51	-0,96	-6,95337445	3,5665E-12	2,2311E-11

YFR045W	683,48	0,38	-1,4	-6,95141115	3,6165E-12	2,2601E-11
YER138W-A	629,29	2,67	1,42	6,94925849	3,6721E-12	2,2926E-11
YCL024W	653,89	0,38	-1,39	-6,94625702	3,7511E-12	2,3395E-11
YGL059W	1384,1	0,45	-1,15	-6,92875769	4,2455E-12	2,6426E-11
YHR138C	1589,53	0,48	-1,06	-6,92880658	4,2441E-12	2,6426E-11
YBR293W	841,95	2,25	1,17	6,92638577	4,3173E-12	2,6846E-11
YGR244C	4952,99	0,36	-1,47	-6,92544007	4,3462E-12	2,6999E-11
YPR084W	808,91	0,44	-1,17	-6,92461454	4,3716E-12	2,713E-11
YKR065C	2918,85	1,96	0,97	6,92075139	4,4925E-12	2,7852E-11
YOR377W	1138,48	0,46	-1,11	-6,91902504	4,5476E-12	2,8165E-11
YDL148C	3442,84	2,68	1,42	6,91743612	4,5989E-12	2,8455E-11
YLR052W	1094,67	0,46	-1,12	-6,91694714	4,6148E-12	2,8525E-11
YNL098C	5922,95	0,52	-0,95	-6,91468025	4,6892E-12	2,8956E-11
YML038C	1831,53	2,02	1,01	6,90499872	5,0204E-12	3,097E-11
YBR083W	2624,05	0,4	-1,33	-6,90006651	5,1978E-12	3,2033E-11
YNL104C	5979,01	0,53	-0,92	-6,89035342	5,5654E-12	3,4264E-11
YFL045C	7207,24	0,52	-0,95	-6,88512862	5,7735E-12	3,551E-11
YGL083W	1255,79	0,48	-1,06	-6,88081889	5,9509E-12	3,6565E-11
YIL036W	3834,33	0,49	-1,04	-6,88036579	5,9699E-12	3,6645E-11
YFL015C	92,44	7,84	2,97	6,8782847	6,0578E-12	3,7148E-11
YKL110C	1746,35	1,98	0,98	6,87613863	6,1497E-12	3,7674E-11
YML088W	1855,34	2,54	1,35	6,87531334	6,1854E-12	3,7856E-11
YBR090C	128,65	5,06	2,34	6,87411219	6,2377E-12	3,8138E-11
YOL038W	3798,72	0,52	-0,95	-6,87197403	6,3319E-12	3,8677E-11
YDR098C	4715,04	2,36	1,24	6,8705935	6,3935E-12	3,9014E-11
YDL177C	364,7	2,6	1,38	6,85816655	6,975E-12	4,2521E-11
YPL080C	83,25	8,08	3,01	6,85499683	7,1314E-12	4,3432E-11
YER069W	1666,25	2,01	1,01	6,85227897	7,2683E-12	4,4222E-11
YOR287C	509,17	2,93	1,55	6,84753098	7,5135E-12	4,5669E-11
YBR111C	3804,96	0,43	-1,22	-6,84554749	7,6184E-12	4,6261E-11
YHR208W	9849,73	0,41	-1,3	-6,84515561	7,6393E-12	4,6343E-11
YKL032C	3165,31	0,51	-0,98	-6,83969504	7,9362E-12	4,8097E-11
YHR199C	3123,33	2,33	1,22	6,83733992	8,0677E-12	4,8847E-11
YER176W	3503,63	2,14	1,1	6,8338371	8,2673E-12	5,0006E-11
YJR161C	449,72	0,38	-1,38	-6,83209552	8,3683E-12	5,0568E-11
YNCM0006W	40,36	61,81	5,95	6,81934685	9,1455E-12	5,5211E-11
YPL224C	3122,82	2,02	1,01	6,81898392	9,1687E-12	5,5297E-11
YLR163C	2351,36	2	1	6,81852853	9,1978E-12	5,5419E-11
YNL220W	12831,73	2,1	1,07	6,81498927	9,4271E-12	5,6745E-11
YJL055W	5509,38	1,93	0,95	6,81302942	9,5565E-12	5,7468E-11
YDL021W	6998,96	2,84	1,51	6,80763867	9,9214E-12	5,9605E-11
YGL029W	535,82	2,44	1,29	6,80668569	9,9873E-12	5,9943E-11
YBR191W	20086,25	0,52	-0,95	-6,80522668	1,0089E-11	6,0495E-11
YDR054C	4717,53	1,94	0,96	6,80450486	1,014E-11	6,0741E-11
YGR146C-A	115,43	5,89	2,56	6,80213203	1,0308E-11	6,169E-11
YJL103C	639,45	0,36	-1,48	-6,80084663	1,0401E-11	6,2183E-11
YGL197W	2987,3	0,48	-1,07	-6,80011302	1,0454E-11	6,2441E-11
YBL004W	2810,63	2,73	1,45	6,79496166	1,0834E-11	6,4651E-11
YNL322C	5051,78	0,51	-0,98	-6,79224006	1,1041E-11	6,582E-11
YER029C	1398,98	2,02	1,02	6,78628834	1,1506E-11	6,8526E-11

YBR052C	3893,75	0,39	-1,34	-6,78549166	1,1569E-11	6,8839E-11
YNCM0001W	75,64	6,34	2,66	6,78446137	1,1652E-11	6,9266E-11
YPL258C	546,77	2,44	1,29	6,77951291	1,2058E-11	7,1611E-11
YLR102C	1171,89	2,05	1,04	6,77854402	1,2139E-11	7,2024E-11
YLR393W	370,77	0,36	-1,49	-6,77803756	1,2182E-11	7,2208E-11
YLR152C	1178,44	0,43	-1,23	-6,77670162	1,2295E-11	7,2809E-11
YIL005W	2529,63	0,51	-0,98	-6,77294292	1,2619E-11	7,4656E-11
YMR206W	1225,18	0,24	-2,06	-6,77237262	1,2669E-11	7,4879E-11
YDR309C	3462,44	1,89	0,92	6,76861383	1,3002E-11	7,6777E-11
YJL199C	270,77	0,29	-1,77	-6,76845094	1,3017E-11	7,6791E-11
YBR179C	1249,86	0,48	-1,07	-6,76744642	1,3108E-11	7,7252E-11
YLR412C-A	41,73	17,53	4,13	6,75847281	1,3945E-11	8,2113E-11
YEL059W	54,49	10,66	3,41	6,74978007	1,4807E-11	8,7103E-11
YKL088W	3022,49	2,03	1,02	6,74835656	1,4953E-11	8,7878E-11
YJL115W	2113,5	1,93	0,95	6,74599031	1,5199E-11	8,9238E-11
YBL091C	11196,06	1,82	0,86	6,74272463	1,5544E-11	9,1182E-11
YIL131C	882,61	0,47	-1,1	-6,73955887	1,5887E-11	9,3103E-11
YFL041W	1898,49	2,1	1,07	6,72370641	1,7716E-11	1,0372E-10
YER141W	3901,09	0,45	-1,16	-6,72113302	1,8032E-11	1,0537E-10
YGR249W	1102,76	0,42	-1,25	-6,72122093	1,8021E-11	1,0537E-10
YFR007W	675,33	0,4	-1,32	-6,7198687	1,8189E-11	1,0619E-10
YEL012W	3704,3	2,38	1,25	6,71448596	1,8873E-11	1,1008E-10
YMR028W	1398,36	2	1	6,71190616	1,921E-11	1,1194E-10
YKL175W	2951,32	1,98	0,99	6,70550726	2,0071E-11	1,1685E-10
YGR019W	2521,97	0,47	-1,08	-6,70320975	2,0389E-11	1,1859E-10
YMR145C	6189,81	0,49	-1,02	-6,69890142	2,0999E-11	1,2203E-10
YMR154C	544,22	0,41	-1,27	-6,69690575	2,1288E-11	1,2359E-10
YDR436W	1329,13	0,46	-1,12	-6,69552286	2,149E-11	1,2465E-10
YGL124C	1725,66	2,03	1,02	6,69506035	2,1558E-11	1,2493E-10
YGL242C	1428,14	0,41	-1,3	-6,68617672	2,2908E-11	1,3262E-10
YFR006W	3753,98	0,54	-0,9	-6,68507091	2,3081E-11	1,335E-10
YDL122W	3928,99	2,02	1,02	6,68466619	2,3145E-11	1,3363E-10
YNR028W	1175,25	0,48	-1,05	-6,68460754	2,3154E-11	1,3363E-10
YOR084W	661,2	0,34	-1,54	-6,6845243	2,3168E-11	1,3363E-10
YGL070C	1078,48	0,48	-1,06	-6,67575894	2,4596E-11	1,4173E-10
YIL053W	28904,62	1,81	0,86	6,67093428	2,5418E-11	1,4634E-10
YPR040W	2033,42	2,22	1,15	6,66387998	2,6669E-11	1,534E-10
YGR237C	3005,13	0,53	-0,91	-6,66215604	2,6984E-11	1,5506E-10
YDL238C	491,22	0,4	-1,3	-6,65902154	2,7566E-11	1,5826E-10
YGL110C	2457,74	1,96	0,97	6,65372629	2,8576E-11	1,6391E-10
YPL217C	3682,26	2,33	1,22	6,65251633	2,8812E-11	1,6511E-10
YPL075W	6853,23	1,92	0,94	6,64603557	3,0109E-11	1,7239E-10
YLR117C	1845	1,96	0,97	6,64311079	3,0713E-11	1,7568E-10
YOL156W	280	2,72	1,44	6,6384192	3,1707E-11	1,812E-10
YPL103C	820,45	0,47	-1,1	-6,63565926	3,2306E-11	1,8445E-10
YGL057C	431,27	2,44	1,28	6,6353392	3,2376E-11	1,8468E-10
YNL087W	4731,22	2,35	1,23	6,6293202	3,3724E-11	1,9219E-10
YPR057W	1059,75	2,14	1,1	6,62717116	3,4218E-11	1,9483E-10
YAL034C	1737,77	0,4	-1,34	-6,6255973	3,4585E-11	1,9656E-10
YEL013W	2324,22	0,47	-1,09	-6,62563443	3,4576E-11	1,9656E-10

YLR359W	18955,24	0,32	-1,64	-6,62292434	3,5216E-11	1,9997E-10
YCL009C	8800,99	0,55	-0,87	-6,61226478	3,7848E-11	2,1472E-10
YBR245C	3984,29	2,35	1,23	6,60872736	3,8764E-11	2,1971E-10
YIL118W	1805,51	0,51	-0,98	-6,60805985	3,8939E-11	2,205E-10
YOR381W	899,33	2,12	1,08	6,60621024	3,9428E-11	2,2307E-10
YHR087W	5867,56	0,37	-1,43	-6,60300851	4,029E-11	2,2773E-10
YMR113W	1182,31	2,02	1,02	6,6024365	4,0445E-11	2,2841E-10
YLR211C	676,41	2,19	1,13	6,60008185	4,1093E-11	2,3185E-10
YHR075C	709,71	0,38	-1,4	-6,59811457	4,1642E-11	2,3474E-10
YHR128W	2345,9	0,43	-1,21	-6,5961793	4,2189E-11	2,376E-10
YDR247W	2970,05	2,13	1,09	6,59534578	4,2427E-11	2,3873E-10
YGL187C	2157,47	0,36	-1,49	-6,59507531	4,2504E-11	2,3895E-10
YBL032W	3410,95	0,51	-0,98	-6,59322393	4,3038E-11	2,4173E-10
YPR137C-B	172,14	3,42	1,77	6,58639134	4,5065E-11	2,5288E-10
YPR112C	2246,59	2,78	1,48	6,57879504	4,7428E-11	2,659E-10
YCL047C	555,62	0,39	-1,36	-6,5739364	4,9002E-11	2,7448E-10
YER088C	7825,63	0,56	-0,85	-6,5701465	5,0266E-11	2,8131E-10
YLR403W	1370,33	0,49	-1,03	-6,55900205	5,4169E-11	3,0288E-10
YGR080W	2017,29	0,51	-0,97	-6,55661096	5,5044E-11	3,075E-10
YML126C	4919,67	0,45	-1,14	-6,54313294	6,0243E-11	3,3624E-10
YKL174C	1021,86	0,43	-1,22	-6,54004708	6,1499E-11	3,4294E-10
YJL062W	1065,05	0,46	-1,12	-6,53595591	6,3205E-11	3,5213E-10
YER013W	1429,73	2,22	1,15	6,5251292	6,7943E-11	3,7819E-10
YIL071C	827,51	0,46	-1,12	-6,52410277	6,841E-11	3,8045E-10
YER168C	1326,07	2,17	1,12	6,52143518	6,9638E-11	3,8659E-10
YGR008C	8154,61	0,39	-1,37	-6,52153057	6,9594E-11	3,8659E-10
YGR065C	681,74	2,45	1,29	6,5208671	6,9902E-11	3,8771E-10
YLR201C	1909,82	0,41	-1,27	-6,51648846	7,1972E-11	3,9883E-10
YDR040C	361,71	2,48	1,31	6,51569102	7,2356E-11	4,006E-10
YKL074C	1402,35	2,1	1,07	6,51306337	7,3633E-11	4,0731E-10
YHR068W	9042,23	2,05	1,03	6,51141544	7,4446E-11	4,1144E-10
YDR363W-A	3007,53	1,93	0,95	6,51045374	7,4924E-11	4,1371E-10
YDL037C	1970,39	22,24	4,48	6,50122506	7,9669E-11	4,3952E-10
YBR109C	7027,83	2,11	1,07	6,50038371	8,0115E-11	4,4159E-10
YHR021C	10119,28	0,54	-0,9	-6,49793587	8,143E-11	4,4822E-10
YNL149C	1567,07	2,03	1,02	6,49787595	8,1462E-11	4,4822E-10
YNL130C-A	68,63	7,16	2,84	6,49599945	8,2484E-11	4,5344E-10
YKR070W	2100,18	1,91	0,94	6,49067556	8,5452E-11	4,6935E-10
YER053C-A	388,82	0,29	-1,8	-6,48263385	9,0135E-11	4,9463E-10
YMR130W	908,96	0,48	-1,05	-6,48164126	9,073E-11	4,9745E-10
YBR016W	1681,89	0,49	-1,02	-6,47615365	9,409E-11	5,1542E-10
YER052C	5316,09	3,12	1,64	6,47367475	9,5648E-11	5,2349E-10
YJL163C	1060,31	0,34	-1,55	-6,47075346	9,7515E-11	5,3324E-10
YLR145W	547,29	2,22	1,15	6,46684501	1,0007E-10	5,4673E-10
YML119W	783,84	0,45	-1,15	-6,46607533	1,0058E-10	5,4904E-10
YBL072C	15807,24	1,94	0,95	6,46477695	1,0145E-10	5,5329E-10
YML128C	10750,25	0,3	-1,74	-6,46057637	1,043E-10	5,6837E-10
YOR338W	202,83	3,6	1,85	6,45318561	1,0952E-10	5,9628E-10
YGR214W	23723,8	0,53	-0,92	-6,44480483	1,1575E-10	6,2963E-10
YOR327C	2080,9	1,9	0,93	6,4444255	1,1604E-10	6,3065E-10

YDL031W	2899,16	2,3	1,2	6,44140986	1,1837E-10	6,4275E-10
YDR273W	578,14	2,37	1,24	6,4411799	1,1855E-10	6,4316E-10
YJL070C	1143,72	0,45	-1,15	-6,43740669	1,2153E-10	6,5861E-10
YNL129W	1128,54	2,08	1,05	6,43731308	1,2161E-10	6,5861E-10
YBL034C	1213,04	2,19	1,13	6,43643875	1,2231E-10	6,6183E-10
YPL089C	3785,08	1,96	0,97	6,43216246	1,258E-10	6,8014E-10
YPR172W	1882,1	0,51	-0,96	-6,42814637	1,2917E-10	6,9774E-10
YAL035W	15170,39	2,29	1,19	6,42661168	1,3048E-10	7,042E-10
YNL288W	1271,1	0,51	-0,98	-6,42113742	1,3526E-10	7,2937E-10
YNL264C	1481,22	0,52	-0,95	-6,41743216	1,3859E-10	7,4669E-10
YDL110C	2681,95	0,34	-1,56	-6,41378351	1,4195E-10	7,6413E-10
YFL022C	6424,55	0,47	-1,09	-6,41331639	1,4239E-10	7,6581E-10
YDR297W	2642,83	0,5	-0,99	-6,41204934	1,4358E-10	7,7154E-10
YOR011W-A	41,7	22,39	4,48	6,4068335	1,4857E-10	7,977E-10
YDL076C	1320,18	0,51	-0,98	-6,40582482	1,4956E-10	8,023E-10
YAL059W	707,39	2,27	1,19	6,40277915	1,5257E-10	8,1776E-10
YMR311C	6358,77	2,04	1,03	6,40224914	1,531E-10	8,199E-10
YDR043C	1588,28	2,23	1,15	6,3989561	1,5644E-10	8,3706E-10
YNL173C	6965,93	2,14	1,1	6,39492304	1,6063E-10	8,5871E-10
YKL067W	5681,62	0,5	-1,01	-6,39278554	1,6289E-10	8,7006E-10
YIL087C	844,37	0,3	-1,73	-6,39179381	1,6395E-10	8,7497E-10
YMR296C	3340,41	0,44	-1,2	-6,39097723	1,6483E-10	8,789E-10
YDR487C	5675,26	2,01	1,01	6,38289604	1,7377E-10	9,2578E-10
YAL022C	1077,54	0,47	-1,1	-6,38245935	1,7427E-10	9,2763E-10
YDR486C	2466,25	1,87	0,9	6,37464075	1,8339E-10	9,7537E-10
YCR016W	1914,97	2,38	1,25	6,37359153	1,8465E-10	9,8123E-10
YLR293C	23118,29	2,01	1,01	6,37265827	1,8578E-10	9,8638E-10
YOL026C	681,07	0,36	-1,49	-6,36708389	1,9266E-10	1,022E-09
YLR035C	771,88	0,48	-1,07	-6,36685955	1,9294E-10	1,0226E-09
YDL194W	1116,3	0,46	-1,11	-6,35558602	2,0763E-10	1,0996E-09
YGR145W	2830,13	2,87	1,52	6,35468066	2,0886E-10	1,1051E-09
YNL069C	24052,04	0,54	-0,88	-6,34184099	2,2704E-10	1,2003E-09
YDR403W	251,38	3,12	1,64	6,33743418	2,3362E-10	1,2341E-09
YBR126W-A	2470,96	0,44	-1,2	-6,33306907	2,4033E-10	1,2684E-09
Q0070	32,21	24,02	4,59	6,33240969	2,4136E-10	1,2728E-09
YCR106W	796,14	2,26	1,17	6,32959659	2,458E-10	1,2951E-09
YOR002W	1868,78	0,52	-0,93	-6,32441658	2,5419E-10	1,3382E-09
YDR383C	581,43	2,21	1,15	6,31913053	2,6304E-10	1,3836E-09
YOR259C	6694,15	1,82	0,86	6,31857646	2,6398E-10	1,3874E-09
YHR147C	1131,5	0,44	-1,18	-6,31723666	2,6628E-10	1,3983E-09
YLR170C	1133,54	1,96	0,97	6,31617505	2,6812E-10	1,4067E-09
YLR149C	2673,11	0,39	-1,36	-6,31180019	2,7581E-10	1,4458E-09
YPR140W	1115,46	0,42	-1,24	-6,30882715	2,8116E-10	1,4726E-09
YAR027W	1712,54	0,52	-0,96	-6,3051236	2,8796E-10	1,507E-09
YJR039W	1054,38	0,49	-1,02	-6,2980792	3,0136E-10	1,5758E-09
YDR451C	523,17	0,41	-1,28	-6,29567623	3,0606E-10	1,5977E-09
YHR215W	2701,79	2,8	1,48	6,29577239	3,0587E-10	1,5977E-09
YDL004W	4263,06	0,54	-0,89	-6,29521893	3,0697E-10	1,6011E-09
YKL040C	2369,73	1,83	0,87	6,28954109	3,1841E-10	1,6593E-09
YDR415C	814,59	0,47	-1,08	-6,28506028	3,2773E-10	1,7065E-09

YJR107W	697,8	0,44	-1,18	-6,28323433	3,316E-10	1,7252E-09
YHL004W	1794,53	0,49	-1,02	-6,28092205	3,3657E-10	1,7496E-09
YGL050W	758,27	0,47	-1,1	-6,27951118	3,3964E-10	1,7641E-09
YER083C	1716,12	0,53	-0,92	-6,27911545	3,405E-10	1,7671E-09
YDR423C	2746,85	1,89	0,92	6,27450708	3,5074E-10	1,8187E-09
YOR302W	152,04	3,98	1,99	6,2693825	3,6248E-10	1,878E-09
YMR207C	1525,9	1,96	0,97	6,26843551	3,6469E-10	1,8879E-09
YNL067W	5917,62	1,8	0,85	6,26780103	3,6618E-10	1,894E-09
YFR043C	775,49	2,2	1,14	6,26709009	3,6786E-10	1,9011E-09
YMR083W	14217,29	0,5	-1	-6,26446077	3,7412E-10	1,9319E-09
YAR019C	970,56	0,5	-1	-6,26407262	3,7505E-10	1,9351E-09
YER050C	2279,89	1,84	0,88	6,25016611	4,1002E-10	2,1137E-09
YLR132C	555,37	0,43	-1,2	-6,2446935	4,2463E-10	2,1872E-09
YLL002W	544,83	0,45	-1,14	-6,24294674	4,294E-10	2,21E-09
YBL040C	3131,81	1,92	0,94	6,24091084	4,3503E-10	2,2325E-09
YKL041W	1969,84	1,9	0,93	6,24084536	4,3521E-10	2,2325E-09
YKL215C	2766,19	1,9	0,93	6,24096022	4,3489E-10	2,2325E-09
YOR178C	2597,77	0,5	-1,01	-6,2408742	4,3513E-10	2,2325E-09
YNL172W	1333,31	2,21	1,14	6,23799347	4,4322E-10	2,2717E-09
YKL161C	692,88	2,17	1,12	6,23711049	4,4573E-10	2,2827E-09
YBR114W	1231,79	0,51	-0,98	-6,23546119	4,5045E-10	2,3049E-09
YKL044W	204,92	3,47	1,8	6,23521924	4,5115E-10	2,3066E-09
YNCL0003W	753,04	2,45	1,29	6,23459439	4,5295E-10	2,3139E-09
YBR257W	548,66	2,18	1,13	6,22259826	4,8899E-10	2,4944E-09
YIL076W	7263,92	1,75	0,81	6,22256691	4,8909E-10	2,4944E-09
YNL103W	2677,31	1,86	0,89	6,22045548	4,9571E-10	2,5261E-09
YDR185C	294,68	0,34	-1,55	-6,21961475	4,9838E-10	2,5376E-09
YGL196W	1980,13	0,53	-0,91	-6,21830558	5,0255E-10	2,5568E-09
YNL176C	3311,17	1,85	0,89	6,21769828	5,045E-10	2,5646E-09
YOR241W	1497,34	0,48	-1,06	-6,20998217	5,2991E-10	2,6916E-09
YHR136C	1091,16	2,09	1,06	6,20309417	5,5364E-10	2,8098E-09
YKL078W	503,86	3,11	1,64	6,19591854	5,7946E-10	2,9385E-09
YAL031C	1237,54	0,51	-0,97	-6,19521932	5,8204E-10	2,9491E-09
YOL107W	411	0,4	-1,32	-6,18546168	6,1921E-10	3,1349E-09
YBR078W	15463,77	0,5	-1,01	-6,18394832	6,2518E-10	3,1625E-09
YOR108W	2220,65	0,52	-0,96	-6,18225161	6,3194E-10	3,1941E-09
YGR085C	7760,38	1,77	0,82	6,18207414	6,3265E-10	3,1951E-09
YNL093W	396,03	0,37	-1,43	-6,18010759	6,4058E-10	3,2326E-09
YDR158W	11804,24	1,74	0,8	6,17865245	6,4651E-10	3,2598E-09
YJL091C	969,07	0,5	-1	-6,17726934	6,522E-10	3,2858E-09
YBR161W	584,57	0,42	-1,26	-6,17462507	6,6321E-10	3,3386E-09
YPR132W	13612,12	1,91	0,94	6,17369578	6,6712E-10	3,3556E-09
YIR002C	2467,16	1,83	0,87	6,16484596	7,0552E-10	3,5459E-09
YDL190C	3288,89	0,49	-1,04	-6,16341289	7,1194E-10	3,5752E-09
YOR187W	7092,08	0,48	-1,06	-6,16274633	7,1494E-10	3,5874E-09
YDL234C	4440,03	0,54	-0,88	-6,16166123	7,1986E-10	3,6092E-09
YBR044C	1164,46	0,49	-1,02	-6,15912064	7,315E-10	3,6646E-09
YAR042W	3324,87	0,56	-0,84	-6,15852462	7,3426E-10	3,6754E-09
YML021C	878,05	0,46	-1,12	-6,15792306	7,3705E-10	3,6864E-09
YIR014W	556,69	2,2	1,14	6,15776734	7,3778E-10	3,6871E-09

YIL055C	1413,81	1,99	0,99	6,15411288	7,5499E-10	3,7701E-09
YOR371C	1461,19	2,01	1	6,15247517	7,6283E-10	3,8062E-09
YOR191W	4596,02	1,79	0,84	6,15020693	7,7382E-10	3,8579E-09
YDR463W	2601,57	0,53	-0,91	-6,14801937	7,8456E-10	3,9084E-09
YGR122W	886,65	0,49	-1,02	-6,13823899	8,3441E-10	4,1533E-09
YDR098C-B	110,01	3,84	1,94	6,13417459	8,5602E-10	4,2541E-09
YJL101C	6843,88	2,1	1,07	6,1342676	8,5552E-10	4,2541E-09
YGL084C	1770,68	0,54	-0,89	-6,13351618	8,5958E-10	4,2661E-09
YHL017W	1209,07	0,5	-1	-6,1334721	8,5982E-10	4,2661E-09
YPL246C	1950,18	0,5	-1,01	-6,13319371	8,6132E-10	4,2702E-09
YHR194W	884,2	0,42	-1,24	-6,13252528	8,6495E-10	4,2813E-09
YNR001C	19396,79	1,81	0,85	6,13256193	8,6475E-10	4,2813E-09
YKL043W	3731,84	1,88	0,91	6,12512743	9,0611E-10	4,4815E-09
YNL026W	1530,73	0,49	-1,02	-6,12348146	9,1553E-10	4,5245E-09
YJL126W	630,94	2,24	1,16	6,12218147	9,2303E-10	4,5579E-09
YHR065C	2506,06	2,13	1,09	6,1212174	9,2863E-10	4,5819E-09
YKR093W	2610,01	0,44	-1,19	-6,11383576	9,7264E-10	4,7953E-09
YLL042C	226,05	3,14	1,65	6,11277878	9,7911E-10	4,8233E-09
YFR028C	1385,55	0,52	-0,96	-6,112139	9,8304E-10	4,8389E-09
YPR078C	110,57	4,72	2,24	6,10577327	1,023E-09	5,0318E-09
YNCO0025W	347,53	4,59	2,2	6,10550581	1,0248E-09	5,0362E-09
YOR004W	896,68	1,99	0,99	6,10271472	1,0428E-09	5,1209E-09
YLR332W	1400,37	1,98	0,99	6,10238458	1,045E-09	5,1275E-09
YIL065C	829,78	0,45	-1,15	-6,10127135	1,0523E-09	5,1592E-09
YIL093C	1023,03	0,39	-1,35	-6,09641043	1,0848E-09	5,3143E-09
YNL004W	5259,75	1,76	0,81	6,09386861	1,1021E-09	5,3952E-09
YOL013C	1403,54	0,53	-0,91	-6,09009363	1,1284E-09	5,5196E-09
YBR096W	1935,49	0,55	-0,87	-6,08843854	1,1402E-09	5,5726E-09
YOR291W	1577,91	0,51	-0,98	-6,08774328	1,1451E-09	5,5924E-09
YLR213C	284,55	2,54	1,34	6,07787013	1,2179E-09	5,9431E-09
YNL045W	3456,64	0,54	-0,88	-6,07404787	1,2473E-09	6,0768E-09
YPL210C	4877,73	1,88	0,91	6,07415429	1,2464E-09	6,0768E-09
YKL180W	21431,73	0,59	-0,77	-6,071924	1,2639E-09	6,1529E-09
YPR122W	1949,26	0,54	-0,88	-6,07113898	1,2701E-09	6,1783E-09
YNCM0026C	57,28	6,89	2,79	6,06582746	1,3128E-09	6,381E-09
YBR087W	982,55	0,51	-0,98	-6,06500114	1,3195E-09	6,4088E-09
YJL026W	12605,31	1,74	0,8	6,06415311	1,3265E-09	6,4377E-09
YDL051W	3842,48	2,3	1,2	6,06036372	1,3581E-09	6,5861E-09
YHR017W	1552,7	0,41	-1,27	-6,05982475	1,3627E-09	6,603E-09
YGL141W	1550,84	0,48	-1,07	-6,05939795	1,3663E-09	6,6154E-09
YCR095C	1185,49	1,88	0,91	6,05877815	1,3716E-09	6,6358E-09
YGR212W	937,01	2,02	1,01	6,05823698	1,3762E-09	6,653E-09
YHR103W	2066,76	0,55	-0,86	-6,05538618	1,4008E-09	6,7666E-09
YPR013C	1745,48	0,54	-0,89	-6,05321317	1,4198E-09	6,8532E-09
YKR014C	5103,72	1,73	0,79	6,04944579	1,4534E-09	7,01E-09
YPL274W	2155,51	3,57	1,83	6,04802668	1,4663E-09	7,0665E-09
YDL141W	1204,16	0,43	-1,22	-6,0443282	1,5003E-09	7,2249E-09
YGL143C	725,32	0,44	-1,19	-6,03995845	1,5415E-09	7,4175E-09
YOR174W	1100,72	1,97	0,98	6,03806645	1,5597E-09	7,4992E-09
YFR050C	4073,53	0,52	-0,94	-6,03724271	1,5677E-09	7,5317E-09

YLR273C	323,02	0,37	-1,44	-6,03631304	1,5768E-09	7,5694E-09
YOR286W	1568,97	0,53	-0,92	-6,03474746	1,5921E-09	7,6372E-09
YJR111C	877,83	2,04	1,03	6,03368318	1,6026E-09	7,6818E-09
YER045C	1232	1,89	0,92	6,0321659	1,6178E-09	7,7483E-09
YGL127C	785,14	2,03	1,02	6,03188516	1,6206E-09	7,7558E-09
YJR122W	1663,49	2,17	1,12	6,02286622	1,7136E-09	8,1944E-09
YER131W	14314,2	0,49	-1,04	-6,02054708	1,7383E-09	8,3063E-09
YKL127W	2857,05	0,51	-0,96	-6,00655577	1,8951E-09	9,0485E-09
YGL041C-B	51,47	7,43	2,89	6,00285372	1,9388E-09	9,2502E-09
YDL215C	3429,11	2,02	1,01	6,00067398	1,965E-09	9,3681E-09
YER033C	1050,99	0,48	-1,04	-5,998333	1,9935E-09	9,4919E-09
YGR134W	1217,39	2,17	1,12	5,99829296	1,994E-09	9,4919E-09
YOR118W	1479,38	0,53	-0,92	-5,99729973	2,0063E-09	9,5428E-09
YJR103W	6392,08	1,89	0,92	5,99449073	2,0412E-09	9,6943E-09
YKL167C	847,55	2,16	1,11	5,99457073	2,0402E-09	9,6943E-09
YOR136W	10674,34	0,58	-0,78	-5,99267908	2,0641E-09	9,7955E-09
YLR128W	496,57	0,45	-1,14	-5,99146203	2,0796E-09	9,8616E-09
YPR196W	453,93	2,18	1,12	5,99099133	2,0857E-09	9,8826E-09
YLR395C	2176,2	0,42	-1,24	-5,98923897	2,1083E-09	9,9821E-09
YJL181W	293,38	0,38	-1,38	-5,98861343	2,1164E-09	1,0013E-08
YIL112W	4674,26	1,88	0,91	5,97347723	2,3225E-09	1,098E-08
YLR227W-B	5059,01	1,82	0,86	5,95647534	2,5774E-09	1,2175E-08
YKL211C	3721,76	1,86	0,9	5,9559273	2,586E-09	1,2207E-08
YLR436C	2397,82	0,53	-0,91	-5,94746724	2,7232E-09	1,2845E-08
YER055C	5459,33	0,56	-0,85	-5,94452521	2,7726E-09	1,3068E-08
YGR253C	5997,09	1,73	0,79	5,94351591	2,7897E-09	1,3139E-08
YBL061C	3274,35	2	1	5,93991755	2,8517E-09	1,342E-08
YOR292C	657,48	0,47	-1,08	-5,93586528	2,923E-09	1,3745E-08
YLR239C	901,69	0,46	-1,13	-5,93324061	2,9701E-09	1,3956E-08
YBR002C	1861,71	1,82	0,87	5,93272587	2,9795E-09	1,399E-08
YGR233C	2675,4	1,81	0,85	5,92922475	3,0437E-09	1,428E-08
YJR104C	24643,13	1,87	0,9	5,92555377	3,1125E-09	1,4592E-08
YGL164C	2030,37	1,81	0,86	5,92388682	3,1442E-09	1,473E-08
YNL054W-B	1920,51	1,81	0,86	5,92350715	3,1515E-09	1,4753E-08
YIL027C	1027,6	2,15	1,1	5,92001874	3,219E-09	1,5058E-08
YIL162W	10135,85	3,29	1,72	5,91650536	3,2885E-09	1,5371E-08
YIL155C	2786,7	0,32	-1,64	-5,90635969	3,4975E-09	1,6336E-08
YMR181C	1857,55	0,5	-1,01	-5,90602507	3,5046E-09	1,6357E-08
YPR056W	694,75	0,49	-1,02	-5,90559076	3,5139E-09	1,6387E-08
YNCL0006W	30	60,82	5,93	5,90156764	3,6006E-09	1,678E-08
YJR062C	1434,89	0,52	-0,93	-5,89840922	3,6702E-09	1,7091E-08
YNL237W	1605,79	2,24	1,16	5,89810572	3,677E-09	1,711E-08
YHR122W	621,2	2,05	1,03	5,89770362	3,686E-09	1,7139E-08
YLR194C	3274,19	0,47	-1,1	-5,89585342	3,7275E-09	1,7319E-08
YNCJ0007C	375,39	2,64	1,4	5,89465111	3,7547E-09	1,7432E-08
YGL038C	2578,07	1,76	0,82	5,89394417	3,7708E-09	1,7494E-08
YJL205C	482,36	0,36	-1,46	-5,89271194	3,7991E-09	1,7612E-08
YDR128W	3097,32	1,77	0,82	5,89076118	3,8442E-09	1,7808E-08
YDR071C	7538,73	1,75	0,81	5,88930994	3,8781E-09	1,7951E-08
YER126C	1540,06	2,11	1,07	5,88508952	3,9784E-09	1,8402E-08

YGL228W	4599,36	1,78	0,83	5,88433138	3,9967E-09	1,8473E-08
YIR018W	622,13	2,05	1,03	5,87750588	4,1649E-09	1,9236E-08
YDR466W	1942,69	0,55	-0,86	-5,87331321	4,2717E-09	1,9715E-08
YGR238C	1031,79	0,5	-0,99	-5,87103451	4,3308E-09	1,9973E-08
YPL132W	854,74	0,45	-1,14	-5,86982137	4,3626E-09	2,0104E-08
YBL091C-A	1589,2	2,02	1,02	5,86852209	4,397E-09	2,0248E-08
YDR234W	1598,98	0,47	-1,1	-5,86773131	4,418E-09	2,0329E-08
YMR158W	728,62	0,44	-1,18	-5,86694884	4,4389E-09	2,041E-08
YMR049C	3254,56	2,43	1,28	5,86551988	4,4773E-09	2,0572E-08
YDL028C	2464,17	1,76	0,82	5,86455151	4,5035E-09	2,0677E-08
YGR159C	5512,62	2,85	1,51	5,86423569	4,5121E-09	2,0701E-08
YPL193W	1196	1,93	0,95	5,85371092	4,8072E-09	2,2039E-08
YJL047C-A	74,65	5,79	2,53	5,85123391	4,8794E-09	2,2353E-08
YBR222C	3385	0,58	-0,79	-5,84988511	4,9191E-09	2,2519E-08
YBR108W	2468,97	0,56	-0,83	-5,8496608	4,9258E-09	2,2532E-08
YKL056C	25389,33	1,67	0,74	5,84561341	5,047E-09	2,307E-08
YMR006C	885,83	0,42	-1,24	-5,84449768	5,081E-09	2,3208E-08
YOR175C	2638,26	0,57	-0,81	-5,8379234	5,2855E-09	2,4125E-08
YER024W	932,49	2,08	1,05	5,83725161	5,3069E-09	2,4204E-08
YCL044C	1799,9	1,8	0,85	5,82616041	5,6717E-09	2,5849E-08
YPL104W	1000,56	0,52	-0,94	-5,8233204	5,769E-09	2,6273E-08
YBR269C	1180,33	0,44	-1,2	-5,82094648	5,8515E-09	2,663E-08
YEL044W	1381,01	1,95	0,96	5,81257095	6,1521E-09	2,7977E-08
YDR042C	148,39	3,34	1,74	5,80968766	6,259E-09	2,8442E-08
YHR071W	3617,93	1,74	0,8	5,80796611	6,3236E-09	2,8715E-08
YHR202W	694,56	0,5	-1	-5,80565497	6,4115E-09	2,9093E-08
YNL144C	3263,3	0,51	-0,96	-5,80506289	6,4342E-09	2,9175E-08
YFL047W	1443,35	0,53	-0,92	-5,79850506	6,6909E-09	3,0316E-08
YOR148C	771,83	2,16	1,11	5,79669986	6,7633E-09	3,0622E-08
YNL305C	2383,7	0,44	-1,18	-5,796089	6,7879E-09	3,0711E-08
YJR137C	2304,03	3,05	1,61	5,79563593	6,8063E-09	3,0772E-08
YDR299W	1344,58	2,27	1,19	5,79510146	6,828E-09	3,0848E-08
YMR280C	889,58	0,29	-1,78	-5,78615466	7,2016E-09	3,2512E-08
YHR161C	6139,36	1,77	0,82	5,78594598	7,2105E-09	3,2529E-08
YDR495C	1912,37	1,82	0,86	5,78420323	7,2857E-09	3,2844E-08
YDR365W-B	513,75	2,2	1,14	5,78273001	7,3498E-09	3,3109E-08
YKL108W	704,71	1,96	0,97	5,78205146	7,3795E-09	3,3219E-08
YCR067C	2291,21	0,52	-0,95	-5,77977734	7,48E-09	3,3646E-08
YAL008W	1493,02	0,44	-1,18	-5,77707429	7,6011E-09	3,4166E-08
YER107C	1747,55	1,82	0,87	5,77495447	7,6974E-09	3,4574E-08
YDL239C	482,21	0,45	-1,14	-5,77170234	7,8475E-09	3,5223E-08
YMR026C	1184,31	1,83	0,87	5,7699333	7,9303E-09	3,5569E-08
YDR003W-A	385,94	2,27	1,18	5,76964469	7,9439E-09	3,5604E-08
YBR081C	2889,84	1,86	0,9	5,76519392	8,1564E-09	3,653E-08
YNL223W	729,36	0,45	-1,14	-5,76312964	8,2568E-09	3,6953E-08
YGR257C	1907,43	1,87	0,91	5,75313262	8,7605E-09	3,9179E-08
YMR251W-A	31762,61	2,3	1,2	5,74801314	9,0298E-09	4,0355E-08
YJR106W	744,42	0,5	-1,01	-5,73727142	9,6214E-09	4,2968E-08
YFR037C	5263,94	1,71	0,77	5,73332637	9,848E-09	4,3948E-08
YLR231C	1836,74	0,38	-1,38	-5,73137865	9,9618E-09	4,4424E-08

YER087C-B	2852,18	0,58	-0,79	-5,73048268	1,0015E-08	4,4627E-08
YPL150W	1850,58	1,79	0,84	5,72860974	1,0126E-08	4,509E-08
YBR285W	333,04	0,18	-2,49	-5,72283286	1,0476E-08	4,6618E-08
YER095W	762,5	0,5	-0,99	-5,72086375	1,0598E-08	4,7128E-08
YAL017W	3690,31	0,58	-0,79	-5,7200806	1,0647E-08	4,7311E-08
YHR116W	702,32	1,96	0,97	5,71865217	1,0737E-08	4,7677E-08
YGR103W	11478,5	2,69	1,43	5,718342	1,0757E-08	4,773E-08
YKR056W	1178,78	1,95	0,97	5,71459585	1,0997E-08	4,8758E-08
YMR270C	1300,76	1,85	0,89	5,71244221	1,1137E-08	4,9344E-08
YNL038W	1424,68	1,79	0,84	5,70802814	1,1429E-08	5,0605E-08
YDL012C	1478,9	0,55	-0,86	-5,70132126	1,1888E-08	5,26E-08
YJR129C	479,37	2,15	1,1	5,70088995	1,1918E-08	5,2695E-08
YGR143W	2520,55	0,52	-0,94	-5,69956477	1,2011E-08	5,3069E-08
YKL052C	1458,11	1,81	0,85	5,6984558	1,209E-08	5,3377E-08
YOL060C	2843,55	0,58	-0,78	-5,69677157	1,221E-08	5,3869E-08
YBL089W	602,53	0,49	-1,03	-5,69046351	1,2669E-08	5,5857E-08
Q0065	27,46	56,03	5,81	5,68831656	1,283E-08	5,6524E-08
YHR140W	223,93	0,2	-2,34	-5,68667824	1,2953E-08	5,7028E-08
YDR505C	2067,08	0,51	-0,97	-5,68455468	1,3115E-08	5,77E-08
YOR352W	1283,84	0,47	-1,09	-5,68310046	1,3227E-08	5,8152E-08
YEL025C	1582,17	0,55	-0,86	-5,68212335	1,3303E-08	5,8444E-08
YDR361C	1638,02	2	1	5,68142956	1,3357E-08	5,864E-08
YFL058W	38,16	9,26	3,21	5,68119758	1,3375E-08	5,8678E-08
YGR225W	156,95	0,23	-2,15	-5,66804429	1,4444E-08	6,332E-08
YNL022C	1178,2	1,88	0,91	5,66475284	1,4724E-08	6,4502E-08
YMR204C	714,87	2,04	1,03	5,65996505	1,514E-08	6,6281E-08
YBR216C	1349,44	0,53	-0,92	-5,65977311	1,5157E-08	6,6308E-08
YDL164C	947,73	0,46	-1,13	-5,65555338	1,5534E-08	6,791E-08
YJL132W	532,21	0,46	-1,11	-5,65062451	1,5987E-08	6,9837E-08
YNL153C	2339,69	1,84	0,88	5,64244318	1,6765E-08	7,3188E-08
YLR079W	2421,14	0,52	-0,95	-5,64083673	1,6923E-08	7,3822E-08
YOR373W	744,85	0,51	-0,96	-5,64033151	1,6972E-08	7,3987E-08
YPR021C	1616,94	0,56	-0,83	-5,63994408	1,7011E-08	7,4102E-08
YPL096C-A	235,6	0,31	-1,69	-5,63948282	1,7056E-08	7,4249E-08
YBR159W	3578,93	0,58	-0,8	-5,63731938	1,7272E-08	7,5135E-08
YJL143W	3630,62	2,04	1,03	5,63713533	1,729E-08	7,5162E-08
YOL115W	1271,47	1,83	0,87	5,6366876	1,7335E-08	7,5305E-08
YFR030W	1877,8	2,37	1,24	5,63473766	1,7533E-08	7,6109E-08
YDR025W	14177,13	0,55	-0,85	-5,63232412	1,778E-08	7,7128E-08
YOR058C	1109,42	1,9	0,92	5,62888983	1,8137E-08	7,8625E-08
YGR005C	3063,02	1,73	0,79	5,62835018	1,8194E-08	7,8816E-08
YJR011C	493,17	2,2	1,14	5,62684088	1,8354E-08	7,9453E-08
YBR012W-B	222,58	2,66	1,41	5,6185042	1,9262E-08	8,3237E-08
YBR017C	3201,29	0,55	-0,86	-5,61844571	1,9268E-08	8,3237E-08
YCR066W	450,85	0,47	-1,1	-5,61865827	1,9245E-08	8,3237E-08
YOR387C	140,68	0,27	-1,9	-5,6183068	1,9284E-08	8,3246E-08
YLR120C	3018,8	0,57	-0,81	-5,61567558	1,958E-08	8,4461E-08
YLR212C	778,6	0,49	-1,02	-5,61556204	1,9592E-08	8,4461E-08
YPR023C	3505,64	1,72	0,78	5,61519099	1,9635E-08	8,4584E-08
YHL031C	1230,21	0,54	-0,88	-5,61483076	1,9675E-08	8,4701E-08

YDR167W	1348,07	1,85	0,89	5,60609122	2,0695E-08	8,8974E-08
YKR088C	1502,7	1,78	0,83	5,60607557	2,0697E-08	8,8974E-08
YHR040W	695,06	2,13	1,09	5,60464613	2,0868E-08	8,9649E-08
YHR047C	3685,98	0,51	-0,96	-5,60327459	2,1034E-08	9,0237E-08
YLR050C	521,09	0,43	-1,2	-5,6033808	2,1021E-08	9,0237E-08
YGR192C	598455,74	1,71	0,78	5,60202063	2,1187E-08	9,083E-08
YOR142W-B	210,73	2,55	1,35	5,59813139	2,1667E-08	9,2826E-08
YBR260C	1251,62	0,54	-0,88	-5,59577877	2,1963E-08	9,4029E-08
YBR187W	1534,79	0,56	-0,83	-5,5932567	2,2285E-08	9,534E-08
YNCE0001C	47,6	6,78	2,76	5,59101806	2,2574E-08	9,6511E-08
YOL071W	1005,41	0,5	-0,99	-5,5872284	2,3072E-08	9,8573E-08
YGR109C	92,91	0,23	-2,1	-5,58395523	2,3511E-08	1,0038E-07
YLR329W	154,07	2,86	1,51	5,58378458	2,3534E-08	1,0041E-07
YER133W	11903,17	1,66	0,73	5,58301168	2,3639E-08	1,0079E-07
YIL066C	534,65	2,11	1,08	5,58221564	2,3747E-08	1,0118E-07
YOR313C	122,53	0,27	-1,91	-5,57655509	2,4533E-08	1,0445E-07
YDR479C	1791,01	0,48	-1,07	-5,5748729	2,4771E-08	1,054E-07
YBR256C	4103,72	1,82	0,87	5,56680996	2,5945E-08	1,1031E-07
YDR368W	5717,49	1,76	0,81	5,56278987	2,655E-08	1,1281E-07
YPL031C	2195,11	0,52	-0,94	-5,56124569	2,6786E-08	1,1373E-07
YEL070W	430,63	3,65	1,87	5,55601198	2,7601E-08	1,1711E-07
YGR083C	5440,3	1,93	0,95	5,55588727	2,7621E-08	1,1712E-07
YMR024W	1449,7	0,55	-0,86	-5,55539135	2,7699E-08	1,1737E-07
YGR037C	4411,04	0,58	-0,78	-5,5544989	2,7841E-08	1,1789E-07
YNL011C	806,63	0,5	-0,99	-5,55265313	2,8137E-08	1,1906E-07
YPR043W	10752,26	1,66	0,73	5,55054963	2,8477E-08	1,2042E-07
YHR180W	497,44	2,42	1,27	5,5415398	2,9982E-08	1,267E-07
YGL223C	2692,3	1,71	0,77	5,53772343	3,0643E-08	1,294E-07
YPR113W	3244,44	0,5	-1	-5,53660763	3,0839E-08	1,3014E-07
YAR018C	713,05	0,52	-0,94	-5,53298258	3,1483E-08	1,3277E-07
YBR005W	1171,89	0,52	-0,93	-5,53175218	3,1705E-08	1,3362E-07
YGL183C	90,66	3,77	1,91	5,52892687	3,222E-08	1,3569E-07
YAR014C	1919,06	0,58	-0,79	-5,52491104	3,2965E-08	1,3874E-07
YDR443C	1056,53	0,52	-0,95	-5,5238861	3,3158E-08	1,3946E-07
YPL036W	341,15	2,28	1,19	5,52113949	3,3681E-08	1,4156E-07
YLR207W	1739,42	0,56	-0,84	-5,51777932	3,4331E-08	1,4419E-07
YAR071W	1195,47	2,66	1,41	5,51591826	3,4696E-08	1,4563E-07
YKL055C	151,68	3,31	1,73	5,5153969	3,4799E-08	1,4596E-07
YGR193C	3126,1	0,59	-0,75	-5,5142974	3,5018E-08	1,4678E-07
YOR204W	4694,67	2,29	1,2	5,51024277	3,5834E-08	1,501E-07
YKL140W	2741,87	0,59	-0,76	-5,50878007	3,6133E-08	1,5125E-07
YKR006C	1639,68	0,54	-0,88	-5,50687554	3,6526E-08	1,5279E-07
YJR027W	1716,37	1,94	0,95	5,50555235	3,6801E-08	1,5384E-07
YOR090C	2494	0,59	-0,76	-5,50483126	3,6952E-08	1,5437E-07
YIL011W	463,34	0,39	-1,38	-5,50368951	3,7192E-08	1,5527E-07
YER060W-A	547,49	0,43	-1,22	-5,4992258	3,8146E-08	1,5914E-07
YJR107C-A	415,83	0,47	-1,1	-5,49655276	3,8729E-08	1,6147E-07
YDR126W	517,27	2	1	5,49633535	3,8776E-08	1,6156E-07
YJL125C	1043,74	1,87	0,91	5,49598747	3,8853E-08	1,6166E-07
YNL111C	469,41	0,45	-1,15	-5,49602047	3,8846E-08	1,6166E-07

YML059C	1415,99	0,54	-0,89	-5,49404248	3,9284E-08	1,6334E-07
YNR072W	273,17	2,3	1,2	5,49356834	3,9389E-08	1,6367E-07
YHR081W	1579,36	1,87	0,9	5,49295352	3,9527E-08	1,6413E-07
YDL046W	4490,55	0,59	-0,76	-5,49279669	3,9562E-08	1,6417E-07
YBR038W	1242,28	0,56	-0,85	-5,49215932	3,9705E-08	1,6465E-07
YKL189W	853,35	0,48	-1,05	-5,49092283	3,9984E-08	1,657E-07
YHL008C	2319,53	1,71	0,77	5,48754292	4,0756E-08	1,6879E-07
YKL038W	1808,08	0,55	-0,86	-5,48688627	4,0908E-08	1,693E-07
YLR326W	869,09	1,96	0,97	5,48282222	4,1859E-08	1,7312E-07
YDL159W	1205,27	1,79	0,84	5,48264145	4,1902E-08	1,7318E-07
YBR143C	8351,75	0,55	-0,87	-5,48133669	4,2212E-08	1,7435E-07
YFR010W	7194,81	1,67	0,74	5,48034538	4,245E-08	1,7521E-07
YHR086W	1042,24	0,54	-0,88	-5,47065265	4,4838E-08	1,8495E-07
YCR022C	48,47	6,15	2,62	5,47053219	4,4869E-08	1,8495E-07
YML076C	1086,07	0,52	-0,94	-5,46900305	4,5257E-08	1,8643E-07
YER182W	1173,79	0,44	-1,17	-5,46828269	4,5442E-08	1,8707E-07
YOR233W	1347,39	1,81	0,86	5,46668581	4,5853E-08	1,8863E-07
YOL133W	1267,81	2,02	1,01	5,46351174	4,6681E-08	1,9191E-07
YER139C	834,74	1,86	0,89	5,45927814	4,7807E-08	1,9642E-07
YNL281W	3644,43	1,67	0,74	5,45883335	4,7927E-08	1,9678E-07
YOR158W	1655,59	0,51	-0,96	-5,45626172	4,8626E-08	1,9952E-07
YIL010W	1700,62	0,57	-0,8	-5,45180245	4,9862E-08	2,0445E-07
YFL040W	368,61	2,22	1,15	5,45132405	4,9996E-08	2,0487E-07
YER098W	1267,24	0,51	-0,96	-5,44237549	5,2575E-08	2,1529E-07
YLR116W	1035,7	0,55	-0,86	-5,43785739	5,3925E-08	2,2067E-07
YOL019W	846,58	0,51	-0,96	-5,4376421	5,399E-08	2,208E-07
YGR241C	2385,51	0,58	-0,78	-5,43594126	5,4508E-08	2,2277E-07
YDR091C	2181,67	0,47	-1,09	-5,4354967	5,4644E-08	2,2317E-07
YLR307C-A	31,05	30,93	4,95	5,43525418	5,4718E-08	2,2333E-07
YOR228C	663,04	0,39	-1,37	-5,43424321	5,5029E-08	2,2445E-07
YHL035C	4309,12	1,64	0,72	5,43292397	5,5438E-08	2,2582E-07
YML093W	2628,61	2,02	1,01	5,43293572	5,5434E-08	2,2582E-07
YLR369W	1705,21	0,57	-0,8	-5,43178291	5,5794E-08	2,2712E-07
YDR298C	4404,86	0,52	-0,94	-5,42886398	5,6714E-08	2,3072E-07
YKL168C	1370,76	0,53	-0,93	-5,4286965	5,6767E-08	2,3078E-07
YMR108W	13176,13	2,2	1,14	5,42548989	5,7796E-08	2,3481E-07
YOL118C	226,06	2,74	1,46	5,41915612	5,9881E-08	2,4312E-07
YCR033W	4797,79	1,69	0,76	5,4183149	6,0163E-08	2,4411E-07
YLL058W	1340,63	1,88	0,91	5,41726122	6,0519E-08	2,4539E-07
YPL215W	1352,09	0,55	-0,87	-5,41398186	6,1638E-08	2,4977E-07
YPR079W	1179,97	0,53	-0,92	-5,41290398	6,2011E-08	2,5112E-07
YOR079C	676,19	2,51	1,33	5,41170546	6,2427E-08	2,5264E-07
YGL259W	143,92	0,23	-2,09	-5,4098814	6,3067E-08	2,5506E-07
YMR114C	2001,86	2,07	1,05	5,40835582	6,3606E-08	2,5707E-07
YLL007C	508,63	0,45	-1,16	-5,40522865	6,4726E-08	2,6143E-07
YNL316C	512,77	0,49	-1,03	-5,40318961	6,5466E-08	2,6425E-07
YIL046W	1594,56	1,74	0,8	5,39903911	6,6999E-08	2,7026E-07
YLR352W	824	0,54	-0,89	-5,39514636	6,8468E-08	2,76E-07
YKL068W-A	761,19	2,36	1,24	5,39269345	6,9409E-08	2,7962E-07
YER148W	4533,99	1,76	0,82	5,39199472	6,968E-08	2,8053E-07

YIL062C	3819	0,55	-0,87	-5,39148822	6,9877E-08	2,8114E-07
YOR342C	1276,86	0,56	-0,83	-5,38908804	7,0816E-08	2,8473E-07
YKL149C	799,83	1,85	0,89	5,38364985	7,299E-08	2,9328E-07
YGL213C	1252,25	0,55	-0,87	-5,37596226	7,6175E-08	3,0588E-07
YER112W	2720,09	1,69	0,75	5,37341396	7,726E-08	3,1004E-07
YOR276W	2947,6	0,56	-0,84	-5,37075812	7,8406E-08	3,1444E-07
YGL116W	1506,65	1,73	0,79	5,36985062	7,8802E-08	3,1582E-07
YJL079C	8471,16	0,34	-1,55	-5,36888949	7,9223E-08	3,173E-07
YDR266C	2011,85	0,57	-0,82	-5,36763487	7,9776E-08	3,1931E-07
YMR096W	851,93	3,02	1,59	5,36600513	8,05E-08	3,22E-07
YDL230W	845,72	0,51	-0,98	-5,36227484	8,218E-08	3,2851E-07
YCL012C	569,93	2,05	1,03	5,36041475	8,3031E-08	3,317E-07
YIR008C	786,76	0,51	-0,96	-5,35897134	8,3697E-08	3,3414E-07
YKR057W	7084,77	1,68	0,75	5,35811836	8,4093E-08	3,3551E-07
YPR026W	1579,11	0,51	-0,98	-5,35689848	8,4663E-08	3,3756E-07
YLR296W	47,96	6,11	2,61	5,35495496	8,5578E-08	3,4099E-07
YJL154C	3501,92	0,54	-0,89	-5,3532423	8,6392E-08	3,4402E-07
YIL050W	1356,81	0,55	-0,87	-5,3526121	8,6694E-08	3,45E-07
YFL034C-A	2061,13	1,8	0,85	5,35090513	8,7515E-08	3,4804E-07
YLL006W	600,18	0,5	-1,01	-5,34353149	9,1153E-08	3,6227E-07
YNR035C	5666,7	1,64	0,71	5,34341801	9,121E-08	3,6227E-07
YDR300C	2362,82	1,75	0,81	5,33301379	9,6596E-08	3,8342E-07
YBR290W	2484,87	1,68	0,75	5,32798396	9,9309E-08	3,9394E-07
YLR404W	385,82	0,44	-1,17	-5,32505598	1,0092E-07	4,0008E-07
YPL166W	420,71	0,48	-1,05	-5,32443954	1,0126E-07	4,0118E-07
YJL063C	1731,22	0,52	-0,94	-5,32420419	1,014E-07	4,0145E-07
YLR390W	563,99	0,37	-1,43	-5,32328985	1,0191E-07	4,0321E-07
YIL022W	3650,48	0,61	-0,71	-5,31671089	1,0566E-07	4,178E-07
YJR120W	72,81	4,02	2,01	5,30983208	1,0973E-07	4,336E-07
YLL043W	4422,86	1,74	0,8	5,30670569	1,1162E-07	4,4082E-07
YNL257C	1381,08	0,54	-0,89	-5,30555434	1,1233E-07	4,4333E-07
YGR199W	1267,24	0,57	-0,82	-5,30181908	1,1465E-07	4,5192E-07
YLR019W	1951,88	0,59	-0,76	-5,30183129	1,1465E-07	4,5192E-07
YLR448W	9587,49	0,53	-0,9	-5,29595047	1,184E-07	4,6639E-07
YGR101W	713,67	0,47	-1,09	-5,29523403	1,1886E-07	4,6792E-07
YKL219W	604,29	0,5	-1	-5,29364635	1,199E-07	4,7171E-07
YMR001C	1378,75	0,57	-0,82	-5,29301638	1,2032E-07	4,7304E-07
YNL020C	1086,9	0,54	-0,9	-5,29285572	1,2042E-07	4,7315E-07
YNL063W	885,77	1,98	0,99	5,29214769	1,2089E-07	4,7469E-07
YJL212C	1468,49	0,39	-1,35	-5,29055427	1,2195E-07	4,7854E-07
YEL037C	3550,14	0,57	-0,81	-5,28728742	1,2414E-07	4,8655E-07
YNL010W	4022,44	0,6	-0,74	-5,28731935	1,2412E-07	4,8655E-07
YBL054W	660,11	2,12	1,09	5,28573096	1,252E-07	4,9039E-07
YMR292W	654,28	1,9	0,92	5,28498965	1,2571E-07	4,9207E-07
YOR157C	2903,34	0,58	-0,79	-5,27963825	1,2944E-07	5,0635E-07
YNL123W	3089,02	2,05	1,03	5,2759597	1,3206E-07	5,1628E-07
YBR006W	1539	0,58	-0,78	-5,27436732	1,3321E-07	5,2046E-07
YBL002W	8381,15	0,63	-0,68	-5,27373487	1,3367E-07	5,2193E-07
YHR085W	405,12	2,18	1,13	5,27177737	1,3511E-07	5,272E-07
YOR301W	754,4	0,49	-1,03	-5,26991884	1,3648E-07	5,3223E-07

YGR032W	3916,88	1,65	0,72	5,26884358	1,3729E-07	5,3502E-07
YAR028W	955,24	0,53	-0,91	-5,26443726	1,4062E-07	5,4767E-07
YGL047W	629,54	0,51	-0,97	-5,26369747	1,4119E-07	5,4953E-07
YIL119C	876,57	0,54	-0,89	-5,26311138	1,4164E-07	5,5094E-07
YDR528W	475,58	2,84	1,51	5,2612876	1,4305E-07	5,5609E-07
YLR452C	1886,23	0,5	-0,99	-5,25937853	1,4454E-07	5,6154E-07
YHR092C	13349,03	0,61	-0,72	-5,25874014	1,4505E-07	5,6314E-07
YPL033C	40,67	7,73	2,95	5,25798262	1,4564E-07	5,6511E-07
YGL055W	2099,41	0,59	-0,77	-5,25597593	1,4724E-07	5,7095E-07
YGL163C	1512,53	1,74	0,8	5,25420019	1,4867E-07	5,7613E-07
YHR132C	3739,88	1,63	0,7	5,25164039	1,5075E-07	5,8383E-07
YMR116C	35110,78	1,62	0,69	5,24636457	1,5513E-07	6,0042E-07
YNR060W	460,43	1,97	0,98	5,24206638	1,5879E-07	6,1419E-07
YBR058C	3207,22	1,67	0,74	5,24158982	1,592E-07	6,154E-07
YJR061W	677,09	0,54	-0,9	-5,23749897	1,6277E-07	6,288E-07
YNCE0019W	352,78	0,47	-1,1	-5,2363849	1,6375E-07	6,3221E-07
YMR012W	8102,74	0,52	-0,94	-5,23421802	1,6568E-07	6,3928E-07
YLR119W	775,27	1,83	0,87	5,23102225	1,6858E-07	6,5002E-07
YER032W	854,12	0,55	-0,85	-5,23072149	1,6885E-07	6,5068E-07
YBR148W	211,65	2,38	1,25	5,23023013	1,693E-07	6,516E-07
YBR287W	3149,31	0,58	-0,78	-5,23029782	1,6924E-07	6,516E-07
YMR221C	1280,64	0,58	-0,79	-5,22915726	1,7028E-07	6,5499E-07
YNL240C	1264,2	1,89	0,91	5,22682199	1,7245E-07	6,629E-07
YEL017C-A	2878,17	1,65	0,73	5,22569505	1,735E-07	6,6631E-07
YPR138C	1117,54	1,9	0,92	5,22564432	1,7355E-07	6,6631E-07
YHR090C	794,48	1,94	0,96	5,22114009	1,7782E-07	6,823E-07
YEL029C	447,72	0,5	-1,01	-5,2190374	1,7986E-07	6,8924E-07
YNL039W	3030,64	1,64	0,71	5,2190913	1,798E-07	6,8924E-07
YER087W	1024,35	0,56	-0,84	-5,21602849	1,828E-07	7,0009E-07
YKL141W	3504,19	0,42	-1,25	-5,20966201	1,8918E-07	7,241E-07
YFR014C	2237,92	0,5	-1,01	-5,20726712	1,9164E-07	7,326E-07
YHL033C	10987,2	0,56	-0,83	-5,20728368	1,9163E-07	7,326E-07
YMR304W	2276,15	0,57	-0,81	-5,20534941	1,9363E-07	7,3975E-07
YAL049C	1705,38	0,57	-0,81	-5,20301396	1,9608E-07	7,4866E-07
YDR276C	7198,85	0,55	-0,87	-5,20255359	1,9657E-07	7,5005E-07
YKL209C	3866,4	0,59	-0,75	-5,20116039	1,9805E-07	7,5523E-07
YKR016W	3259,41	0,61	-0,72	-5,19837417	2,0104E-07	7,6617E-07
YDR148C	5275,23	0,53	-0,93	-5,19418916	2,0561E-07	7,8312E-07
YLR308W	29,21	11,08	3,47	5,19377037	2,0608E-07	7,8441E-07
YER122C	5034,46	0,6	-0,73	-5,18954921	2,108E-07	8,0191E-07
YJL134W	1332,21	1,77	0,82	5,18705616	2,1364E-07	8,1221E-07
YAL003W	32033,7	1,78	0,83	5,18482677	2,1622E-07	8,2149E-07
YHR094C	3943,54	9,93	3,31	5,18137361	2,2026E-07	8,3633E-07
YJL109C	2631,49	2,22	1,15	5,17823376	2,24E-07	8,5001E-07
YHL050C	608,54	1,91	0,94	5,17254333	2,3093E-07	8,7578E-07
YJL073W	1483,77	1,85	0,89	5,17147693	2,3225E-07	8,8026E-07
YJR017C	2336,82	0,56	-0,84	-5,17133281	2,3243E-07	8,804E-07
YJL168C	1556,43	1,92	0,94	5,16974816	2,3441E-07	8,8736E-07
YJL200C	1572,66	0,57	-0,8	-5,16855465	2,3591E-07	8,925E-07
YKL116C	915,32	0,54	-0,89	-5,16745092	2,3731E-07	8,9724E-07

YMR050C	336,42	2,09	1,06	5,16729347	2,3751E-07	8,9745E-07
YDR119W-A	541,15	0,03	-4,84	-5,16552359	2,3977E-07	9,0543E-07
YAL021C	1418,17	0,48	-1,04	-5,15944955	2,4768E-07	9,3473E-07
YLR044C	405303,24	1,67	0,74	5,15878442	2,4856E-07	9,3749E-07
YOL006C	3007,98	1,69	0,76	5,15306285	2,5627E-07	9,6597E-07
YHR035W	200,25	0,38	-1,4	-5,15231718	2,5729E-07	9,6924E-07
YPL066W	1978,44	0,57	-0,8	-5,15197562	2,5776E-07	9,7041E-07
YKL185W	2586,18	0,57	-0,8	-5,15154328	2,5835E-07	9,7207E-07
YPL109C	788,09	0,47	-1,08	-5,15125286	2,5875E-07	9,7298E-07
YDR161W	2091,51	1,96	0,97	5,13941089	2,756E-07	1,0357E-06
YDR295C	1912,02	1,7	0,76	5,13685746	2,7937E-07	1,0492E-06
YPL222W	2227,96	2,38	1,25	5,13662719	2,7971E-07	1,0499E-06
YDR322W	1223,01	0,56	-0,83	-5,13245743	2,8598E-07	1,0728E-06
YMR058W	9166,59	1,84	0,88	5,12995288	2,8981E-07	1,0865E-06
YHR037W	2410,25	0,54	-0,88	-5,12884256	2,9153E-07	1,0923E-06
YFR042W	585,65	0,51	-0,98	-5,12250678	3,015E-07	1,1289E-06
YJR096W	2615,28	0,44	-1,18	-5,11772174	3,0925E-07	1,1573E-06
YOL136C	2755,65	2,28	1,19	5,11681006	3,1075E-07	1,1622E-06
YFR055W	1328,63	1,9	0,93	5,11545826	3,1298E-07	1,1698E-06
YOR070C	1488,43	0,56	-0,85	-5,11348616	3,1627E-07	1,1814E-06
YLR285W	1959,74	0,49	-1,03	-5,11166455	3,1933E-07	1,1921E-06
YDR430C	1925,24	0,59	-0,75	-5,11079847	3,208E-07	1,1969E-06
YDL015C	3868,38	0,61	-0,72	-5,107657	3,2618E-07	1,2162E-06
YOL108C	1014,54	1,99	0,99	5,10335802	3,3368E-07	1,2434E-06
YDR080W	2283,52	1,69	0,76	5,10170011	3,3662E-07	1,2536E-06
YKL192C	4091,04	0,49	-1,02	-5,10102416	3,3782E-07	1,2574E-06
YDR483W	7551,19	0,63	-0,66	-5,09884711	3,4173E-07	1,2704E-06
YOL066C	1396,69	1,7	0,77	5,09886555	3,417E-07	1,2704E-06
YPR129W	2738,78	0,62	-0,7	-5,09669397	3,4564E-07	1,2841E-06
YPL093W	2161,36	2,2	1,14	5,09600595	3,4689E-07	1,288E-06
YGR074W	443,14	0,5	-0,99	-5,09448877	3,4968E-07	1,2976E-06
YLR382C	1337,01	0,58	-0,79	-5,09272326	3,5296E-07	1,309E-06
YPL063W	4797,38	0,61	-0,72	-5,09171268	3,5484E-07	1,3152E-06
YGL248W	2348,76	0,58	-0,79	-5,09016856	3,5775E-07	1,3252E-06
YLR048W	22922,38	0,54	-0,89	-5,08788047	3,6209E-07	1,3405E-06
YGL212W	406,87	0,5	-1,01	-5,08745565	3,629E-07	1,3427E-06
YGR283C	548,74	1,92	0,94	5,08295816	3,716E-07	1,374E-06
YGL091C	1319,79	1,69	0,76	5,08221731	3,7305E-07	1,3786E-06
YIL157C	1629,37	0,53	-0,93	-5,07841609	3,8059E-07	1,4056E-06
YPR179C	1995,41	1,67	0,74	5,07713094	3,8318E-07	1,4143E-06
YGL080W	1377,4	0,59	-0,76	-5,07582786	3,8581E-07	1,4232E-06
YMR102C	3610,26	2,06	1,04	5,07468252	3,8814E-07	1,4309E-06
YMR052W	399,11	2,02	1,02	5,07432309	3,8888E-07	1,4328E-06
YML019W	1254,85	0,58	-0,79	-5,07409423	3,8935E-07	1,4337E-06
YOR375C	17242,17	1,57	0,65	5,0693133	3,9925E-07	1,4693E-06
YBL019W	666,48	0,53	-0,93	-5,06714225	4,0383E-07	1,4853E-06
YER019C-A	1952,66	0,55	-0,85	-5,0669233	4,043E-07	1,4861E-06
YCR083W	738,66	0,52	-0,94	-5,06232817	4,1417E-07	1,5215E-06
YMR034C	402	2	1	5,06134014	4,1632E-07	1,5285E-06
YLR139C	721,65	0,55	-0,87	-5,05781577	4,2409E-07	1,5561E-06

YPL253C	692,77	1,85	0,89	5,05735233	4,2512E-07	1,5589E-06
YJL159W	59745,36	0,59	-0,77	-5,05706054	4,2577E-07	1,5604E-06
YJL084C	1356,4	0,58	-0,78	-5,04745541	4,4773E-07	1,6399E-06
YGL126W	1282,66	0,58	-0,77	-5,04460969	4,5445E-07	1,6635E-06
YLR295C	3095,34	0,5	-1	-5,04056284	4,6416E-07	1,6981E-06
YHR102W	1606,5	0,58	-0,79	-5,03608454	4,7515E-07	1,7373E-06
YDR141C	2416,25	0,53	-0,91	-5,0348925	4,7812E-07	1,7471E-06
YDL085W	395,73	0,24	-2,07	-5,03340499	4,8184E-07	1,7597E-06
YPL098C	1211,39	0,53	-0,91	-5,03230673	4,8461E-07	1,7688E-06
YDR204W	2143,47	0,53	-0,91	-5,03027055	4,8979E-07	1,7866E-06
YLR344W	9739,25	0,58	-0,78	-5,02813587	4,9527E-07	1,8055E-06
YHR052W	2100,75	2,02	1,01	5,02671623	4,9895E-07	1,8179E-06
YBR230W-A	252,55	2,87	1,52	5,02624203	5,0018E-07	1,8213E-06
YJL196C	1739,71	0,52	-0,94	-5,02301949	5,0865E-07	1,8511E-06
YDL089W	1193,12	0,59	-0,77	-5,02240899	5,1027E-07	1,8559E-06
YIR039C	820,8	0,41	-1,29	-5,02116911	5,1358E-07	1,8668E-06
YJR115W	872,45	0,36	-1,48	-5,01477441	5,3096E-07	1,9288E-06
YJL023C	640,28	1,97	0,98	5,00925316	5,4642E-07	1,9838E-06
YBR088C	1163,07	0,54	-0,89	-5,00870485	5,4798E-07	1,9883E-06
YBR084C-A	28622,04	1,68	0,75	5,00596729	5,5582E-07	2,0156E-06
YOL047C	645,97	0,41	-1,3	-5,00535359	5,576E-07	2,0209E-06
YGL198W	1396,16	0,48	-1,06	-5,00478419	5,5925E-07	2,0257E-06
YKL049C	889,41	1,75	0,8	5,003069	5,6425E-07	2,0426E-06
YHR031C	847,03	1,74	0,8	5,00144406	5,6902E-07	2,0587E-06
YPL165C	693,24	0,52	-0,95	-5,00101089	5,703E-07	2,0621E-06
YKL060C	452179,57	1,8	0,84	5,00044206	5,7199E-07	2,066E-06
YKL061W	687,3	1,97	0,98	5,00042381	5,7204E-07	2,066E-06
YOR307C	1392,65	0,59	-0,76	-4,99740243	5,8108E-07	2,0974E-06
YOR197W	4505,22	0,58	-0,78	-4,99089389	6,0101E-07	2,1681E-06
YIL088C	3717,12	0,54	-0,88	-4,99051268	6,0219E-07	2,1698E-06
YMR178W	2122,36	0,58	-0,78	-4,99061436	6,0188E-07	2,1698E-06
YJL036W	3110,78	1,6	0,68	4,98864358	6,0805E-07	2,1897E-06
YMR071C	2061	1,82	0,86	4,98363344	6,2401E-07	2,2459E-06
YIL099W	521,48	2,3	1,2	4,98257216	6,2745E-07	2,2569E-06
YHR016C	2867,96	0,54	-0,88	-4,98182664	6,2987E-07	2,2643E-06
YLL062C	743,59	2,77	1,47	4,97597851	6,4919E-07	2,3324E-06
YFL031W	8881,68	1,58	0,66	4,96816514	6,7589E-07	2,4249E-06
YKL099C	932,62	1,77	0,82	4,96810279	6,7611E-07	2,4249E-06
YNL225C	986,98	1,81	0,86	4,96825247	6,7559E-07	2,4249E-06
YOR297C	874,7	1,75	0,81	4,96550785	6,8522E-07	2,4562E-06
YHR019C	14986,07	0,59	-0,76	-4,96391515	6,9086E-07	2,475E-06
YDL241W	475,51	0,45	-1,14	-4,96216254	6,9713E-07	2,496E-06
YHR013C	1752,83	0,6	-0,73	-4,96200667	6,9769E-07	2,4966E-06
YDR087C	1268,75	1,96	0,97	4,96119856	7,006E-07	2,5041E-06
YER019W	1218,94	0,57	-0,81	-4,96120827	7,0056E-07	2,5041E-06
YAL002W	2094,17	0,6	-0,74	-4,95807715	7,1194E-07	2,5432E-06
YKR048C	9637,62	1,58	0,66	4,95629305	7,1851E-07	2,5652E-06
YCR015C	555,03	1,85	0,89	4,9539072	7,2738E-07	2,5948E-06
YFR040W	6564,06	1,59	0,67	4,95383776	7,2764E-07	2,5948E-06
YDR374C	30,7	9,04	3,18	4,95017581	7,4146E-07	2,6426E-06

YKL162C	412,81	0,5	-1	-4,94917435	7,4529E-07	2,6547E-06
YGL225W	1835,54	0,54	-0,9	-4,94267202	7,7059E-07	2,7432E-06
YOR075W	1286,12	1,87	0,9	4,93821717	7,884E-07	2,805E-06
YNL059C	3838,8	1,65	0,72	4,93585256	7,9801E-07	2,8376E-06
YOL059W	8276,77	1,61	0,69	4,93487736	8,0201E-07	2,8502E-06
YER144C	1624,19	0,56	-0,84	-4,93412715	8,051E-07	2,8595E-06
YPL159C	1407,31	1,7	0,77	4,93125537	8,1703E-07	2,9002E-06
YER079W	3075,99	1,94	0,96	4,92973108	8,2343E-07	2,9213E-06
YGL202W	10402,25	0,64	-0,64	-4,92900778	8,2648E-07	2,9304E-06
YPL201C	175,15	2,8	1,49	4,92865798	8,2796E-07	2,934E-06
YNL317W	1056,84	0,58	-0,79	-4,92589685	8,3974E-07	2,974E-06
YKR087C	1747,15	1,72	0,78	4,9239905	8,4797E-07	3,0015E-06
YIL126W	4773,84	1,68	0,75	4,91413338	8,9176E-07	3,1547E-06
YDR502C	4861,54	2,58	1,37	4,90961585	9,1255E-07	3,2264E-06
YOR348C	170,08	3,09	1,63	4,90831573	9,1862E-07	3,246E-06
YLR146W-A	177,53	0,27	-1,89	-4,90731171	9,2333E-07	3,2608E-06
YBR094W	1582,52	0,59	-0,75	-4,90304622	9,4362E-07	3,3305E-06
YPL116W	1385,45	0,6	-0,73	-4,90118124	9,5262E-07	3,3604E-06
YDR014W	554,72	0,5	-0,99	-4,89970154	9,5982E-07	3,382E-06
YLL045C	31851,36	0,6	-0,74	-4,89974071	9,5963E-07	3,382E-06
YJL140W	4898,43	1,62	0,7	4,89935246	9,6153E-07	3,3861E-06
YKL139W	1149,06	1,68	0,75	4,89885515	9,6397E-07	3,3927E-06
YBR056W	4204,64	0,6	-0,74	-4,89772074	9,6955E-07	3,4104E-06
YPR133W-A	1925,32	0,55	-0,87	-4,89514236	9,8235E-07	3,4535E-06
YNL121C	7842,17	0,63	-0,66	-4,89344637	9,9085E-07	3,4814E-06
YJR143C	3032,99	0,54	-0,89	-4,8906232	1,0052E-06	3,5298E-06
YNL209W	25995,8	0,54	-0,89	-4,88997446	1,0085E-06	3,5394E-06
YNR024W	797,73	1,75	0,8	4,88864997	1,0153E-06	3,5613E-06
YPL023C	2548,49	0,61	-0,72	-4,88802981	1,0185E-06	3,5705E-06
YNR016C	9676,54	1,99	0,99	4,88733516	1,0221E-06	3,5811E-06
YJL177W	10848,95	0,62	-0,69	-4,88060659	1,0576E-06	3,7034E-06
YDR404C	1632,13	0,61	-0,71	-4,88039076	1,0588E-06	3,7054E-06
YDL226C	2326,01	0,61	-0,71	-4,87585212	1,0834E-06	3,7895E-06
YJR057W	677,33	0,54	-0,89	-4,874758	1,0894E-06	3,8084E-06
YPR046W	253,92	2,17	1,12	4,87167296	1,1066E-06	3,8662E-06
YFR034C	1338,32	1,67	0,74	4,86745391	1,1305E-06	3,9474E-06
YFR004W	6602,96	1,58	0,66	4,86224403	1,1606E-06	4,0504E-06
YLL031C	1932,15	0,53	-0,92	-4,8520545	1,2219E-06	4,2619E-06
YMR110C	4713,02	0,53	-0,91	-4,85134817	1,2263E-06	4,2747E-06
YPR170W-B	3931,57	1,81	0,86	4,8511221	1,2276E-06	4,2771E-06
YDL066W	2955,74	1,58	0,66	4,84989354	1,2353E-06	4,3013E-06
YDR366C	34,3	8,3	3,05	4,84908056	1,2404E-06	4,3166E-06
YPL130W	27,65	10,5	3,39	4,84855467	1,2436E-06	4,3256E-06
YNR002C	545,82	0,41	-1,29	-4,84691093	1,254E-06	4,3591E-06
YML069W	5348,74	1,69	0,75	4,84445928	1,2696E-06	4,4108E-06
YEL047C	3486,48	0,63	-0,66	-4,84103973	1,2916E-06	4,4824E-06
YGR161C-D	476,68	1,87	0,9	4,84109508	1,2913E-06	4,4824E-06
YMR273C	2009,69	0,61	-0,71	-4,84064278	1,2942E-06	4,4864E-06
YNL194C	1609,25	0,12	-3,04	-4,84068992	1,2939E-06	4,4864E-06
YER007C-A	2565,49	1,61	0,69	4,8386089	1,3075E-06	4,53E-06

YDL198C	1575,06	0,6	-0,75	-4,8354199	1,3286E-06	4,6006E-06
YHR160C	454,2	2,14	1,1	4,83509137	1,3308E-06	4,6057E-06
YLL023C	2326,64	0,51	-0,97	-4,82793838	1,3795E-06	4,7715E-06
YLL052C	113,53	3,49	1,8	4,82633341	1,3907E-06	4,8075E-06
YMR038C	4947,3	1,58	0,66	4,82512204	1,3992E-06	4,8341E-06
YFR049W	1013,26	0,48	-1,07	-4,82485786	1,401E-06	4,8378E-06
YJL185C	372,98	0,38	-1,41	-4,82437676	1,4044E-06	4,8468E-06
YGL009C	23813,84	2,15	1,1	4,82034468	1,4331E-06	4,9431E-06
YCR092C	859,58	0,58	-0,78	-4,81767504	1,4524E-06	5,0054E-06
YML101C	460,11	0,5	-1	-4,81762117	1,4528E-06	5,0054E-06
YFL051C	150,09	0,28	-1,84	-4,81472205	1,474E-06	5,0758E-06
YGR013W	2363,25	1,66	0,73	4,81350962	1,483E-06	5,1039E-06
YPR145W	11466,75	0,51	-0,97	-4,81220925	1,4927E-06	5,1344E-06
YIL078W	27387,72	1,67	0,74	4,80741119	1,529E-06	5,2562E-06
YLR263W	575,02	1,8	0,85	4,80330691	1,5607E-06	5,3622E-06
YBR152W	426,6	2,02	1,02	4,80251653	1,5668E-06	5,3775E-06
YDR045C	690,1	1,78	0,83	4,8026116	1,5661E-06	5,3775E-06
YML072C	8513,47	0,65	-0,61	-4,79543377	1,6232E-06	5,5679E-06
YCR068W	719,38	0,57	-0,82	-4,79238553	1,6481E-06	5,6501E-06
YBR246W	1584,91	0,59	-0,77	-4,79149936	1,6554E-06	5,672E-06
YNL001W	1447,61	1,71	0,77	4,78918783	1,6746E-06	5,7345E-06
YER173W	825,99	1,72	0,79	4,78824867	1,6824E-06	5,7583E-06
YDL014W	8799,3	0,53	-0,92	-4,77655632	1,7832E-06	6,0999E-06
YNCP0003W	37,31	8,66	3,11	4,7707709	1,8352E-06	6,2743E-06
YLL053C	89,8	3,32	1,73	4,76859144	1,8552E-06	6,3391E-06
YNL258C	2107,19	1,6	0,68	4,76678502	1,8719E-06	6,3926E-06
YHR110W	1533,69	0,59	-0,77	-4,76536117	1,8852E-06	6,4344E-06
YLL025W	185,94	2,91	1,54	4,76474848	1,8909E-06	6,4504E-06
YOR130C	626,6	1,77	0,82	4,76338945	1,9037E-06	6,4905E-06
YML087C	533,15	2,2	1,14	4,76178431	1,9189E-06	6,5387E-06
YGR021W	937,1	0,52	-0,95	-4,76065206	1,9297E-06	6,5719E-06
YLL040C	5703,23	0,64	-0,65	-4,75863946	1,949E-06	6,6342E-06
YAR007C	1560,99	0,58	-0,79	-4,75738252	1,9612E-06	6,6719E-06
YLR387C	2867,65	1,63	0,7	4,75550945	1,9795E-06	6,7267E-06
YMR219W	4984,3	1,55	0,63	4,75553981	1,9792E-06	6,7267E-06
YLR461W	56,42	4,54	2,18	4,75536493	1,9809E-06	6,7279E-06
YJL210W	911,67	0,52	-0,95	-4,75485537	1,9859E-06	6,7412E-06
YOR294W	993,38	1,88	0,91	4,75395874	1,9947E-06	6,7674E-06
YDL227C	820,08	0,55	-0,86	-4,75068586	2,0273E-06	6,8742E-06
YER158C	1250,86	0,48	-1,07	-4,74405009	2,0949E-06	7,0995E-06
YNL181W	2037,14	1,63	0,7	4,74160004	2,1204E-06	7,182E-06
YCL035C	5844,69	0,57	-0,81	-4,74125832	2,1239E-06	7,1902E-06
YER078C	1231,65	0,59	-0,75	-4,74079687	2,1288E-06	7,2026E-06
YPL140C	987,96	0,58	-0,79	-4,73908275	2,1469E-06	7,2599E-06
YGL022W	5402,17	0,62	-0,69	-4,73864528	2,1515E-06	7,2716E-06
YNL032W	1188,05	0,6	-0,73	-4,73456513	2,1953E-06	7,4154E-06
YDL179W	683,05	0,53	-0,92	-4,73355234	2,2062E-06	7,4485E-06
YFR003C	1626,25	0,61	-0,7	-4,73195032	2,2237E-06	7,5034E-06
YBR196C-A	86,19	3,63	1,86	4,73006604	2,2445E-06	7,5693E-06
YCR075C	364,93	0,47	-1,09	-4,72388755	2,3138E-06	7,7988E-06

YOL073C	1227,86	0,58	-0,8	-4,7166868	2,3972E-06	8,0755E-06
YPL081W	3148,79	1,57	0,65	4,71162968	2,4574E-06	8,274E-06
YCL045C	4622,36	0,59	-0,75	-4,71011294	2,4758E-06	8,3268E-06
YLR354C	16362,71	0,59	-0,77	-4,71020114	2,4747E-06	8,3268E-06
YGR277C	662,92	0,56	-0,84	-4,70985359	2,4789E-06	8,3329E-06
YMR258C	1336,29	0,57	-0,8	-4,70952456	2,483E-06	8,3419E-06
YCR084C	4327,33	0,61	-0,71	-4,70872207	2,4927E-06	8,3702E-06
YNCB0019C	385,09	3,06	1,61	4,70310711	2,5623E-06	8,5992E-06
YBR192W	813,29	0,56	-0,83	-4,70294754	2,5643E-06	8,6013E-06
YDR508C	3038,35	0,64	-0,65	-4,69937774	2,6096E-06	8,7483E-06
YGL015C	38,69	6,78	2,76	4,69765083	2,6317E-06	8,8178E-06
YGR087C	142,87	3,17	1,66	4,69236904	2,7006E-06	9,0437E-06
YPR052C	351,62	0,41	-1,3	-4,69003884	2,7315E-06	9,1424E-06
YOR016C	884,59	0,57	-0,81	-4,68406175	2,8125E-06	9,4082E-06
YBL086C	2241,18	0,58	-0,78	-4,67375746	2,9574E-06	9,8877E-06
YER070W	2731,88	0,56	-0,84	-4,66995924	3,0126E-06	1,0067E-05
YKR076W	2344,46	2,27	1,18	4,66940167	3,0208E-06	1,0089E-05
YDL074C	3012,86	1,63	0,7	4,66609182	3,0698E-06	1,0247E-05
YBR157C	840,98	0,51	-0,96	-4,65617658	3,2214E-06	1,0747E-05
YOL141W	160,66	2,49	1,32	4,65504017	3,2392E-06	1,0801E-05
YNL289W	366,88	0,48	-1,05	-4,65398284	3,2558E-06	1,085E-05
YBR031W	41608,4	1,5	0,59	4,64935276	3,3298E-06	1,1091E-05
YDR099W	14368,74	0,66	-0,6	-4,64903411	3,3349E-06	1,1102E-05
YER067W	30239,01	0,55	-0,87	-4,64749235	3,3599E-06	1,1179E-05
YOR083W	381,97	0,52	-0,95	-4,64614737	3,3819E-06	1,1246E-05
YPL153C	656,66	2,27	1,19	4,64597257	3,3848E-06	1,125E-05
YHR050W	1504,18	0,61	-0,72	-4,6405369	3,4751E-06	1,1544E-05
YJL097W	1489,27	0,57	-0,82	-4,63948665	3,4928E-06	1,1596E-05
YJR154W	259,5	2,08	1,05	4,63907152	3,4998E-06	1,1614E-05
YOR220W	4064,05	0,63	-0,67	-4,63498036	3,5697E-06	1,1839E-05
YKL101W	3309,72	0,57	-0,82	-4,63130358	3,6337E-06	1,2045E-05
YOR320C	1652,13	0,63	-0,67	-4,62976382	3,6608E-06	1,2129E-05
YKL002W	3212,81	1,61	0,68	4,62877002	3,6784E-06	1,218E-05
YBR177C	5267,99	0,65	-0,61	-4,62719117	3,7066E-06	1,2267E-05
YJL171C	2683,62	1,74	0,8	4,62585161	3,7306E-06	1,234E-05
YOR384W	1409,29	1,61	0,69	4,62540475	3,7387E-06	1,236E-05
YDR311W	1167,72	0,61	-0,72	-4,62528447	3,7408E-06	1,2361E-05
YER178W	12394,11	0,65	-0,63	-4,62347579	3,7736E-06	1,2462E-05
YFR011C	1191,49	0,6	-0,74	-4,62334506	3,776E-06	1,2464E-05
YOL104C	594,9	0,57	-0,82	-4,62321578	3,7784E-06	1,2465E-05
YDR473C	1484,24	1,6	0,68	4,62281882	3,7856E-06	1,2482E-05
YJL193W	298,22	0,5	-1,01	-4,62252538	3,791E-06	1,2493E-05
YGR175C	3628,75	0,61	-0,7	-4,62217762	3,7973E-06	1,2507E-05
YPR095C	1228,17	0,55	-0,86	-4,61711974	3,891E-06	1,2809E-05
YFR029W	760,37	0,58	-0,78	-4,61682585	3,8965E-06	1,2821E-05
YKR029C	1868,96	1,62	0,7	4,61366783	3,9562E-06	1,301E-05
YDR063W	2106,63	0,62	-0,7	-4,61024915	4,0219E-06	1,3219E-05
YJR072C	2793,9	1,59	0,67	4,60835703	4,0586E-06	1,3333E-05
YDR064W	17516,19	0,64	-0,64	-4,60707249	4,0838E-06	1,3408E-05
YPR003C	794,21	0,56	-0,85	-4,60270382	4,1704E-06	1,3685E-05

YER080W	5222,1	0,66	-0,61	-4,60107813	4,2031E-06	1,3785E-05
YOR036W	2190,44	0,62	-0,69	-4,60044813	4,2158E-06	1,382E-05
YJR005W	2345,49	0,63	-0,66	-4,59904296	4,2444E-06	1,3906E-05
YJL165C	3854,58	0,62	-0,68	-4,59849104	4,2556E-06	1,3936E-05
YMR166C	664,16	0,53	-0,91	-4,59660293	4,2943E-06	1,4055E-05
Q0120	12,4	208,91	7,71	4,59622146	4,3022E-06	1,4073E-05
YOR201C	1338,25	1,67	0,74	4,59586342	4,3096E-06	1,409E-05
YJL137C	548,52	0,44	-1,17	-4,59453178	4,3372E-06	1,4173E-05
YOL004W	4725,83	0,63	-0,67	-4,59431179	4,3418E-06	1,418E-05
YPR166C	784,93	1,82	0,86	4,58851759	4,464E-06	1,4572E-05
YNL232W	1340,23	1,61	0,68	4,58556822	4,5275E-06	1,4772E-05
YOR154W	836,17	1,72	0,78	4,58464408	4,5476E-06	1,4829E-05
YDR402C	146,05	2,4	1,26	4,58383495	4,5652E-06	1,4879E-05
YGL245W	26282,96	1,59	0,67	4,58318253	4,5795E-06	1,4918E-05
YNL131W	4432,14	0,64	-0,64	-4,58266001	4,591E-06	1,4947E-05
YKL064W	3553,72	0,64	-0,65	-4,58165062	4,6132E-06	1,5012E-05
YOR353C	1586,37	1,6	0,68	4,57933589	4,6645E-06	1,5171E-05
YOR390W	96,48	0,33	-1,62	-4,57744801	4,7068E-06	1,53E-05
YDR400W	1583,98	1,68	0,75	4,57648013	4,7286E-06	1,5363E-05
YPL206C	2476,12	1,65	0,73	4,57534266	4,7544E-06	1,5439E-05
YJR095W	75,16	3,2	1,68	4,57378298	4,79E-06	1,5546E-05
YGL117W	1151,49	1,65	0,72	4,57336023	4,7996E-06	1,5569E-05
YBL064C	5167,75	2,03	1,02	4,57177809	4,836E-06	1,5679E-05
YCL054W	1886,29	2,01	1,01	4,56935189	4,8923E-06	1,5854E-05
YOR289W	1189,53	0,48	-1,07	-4,56886989	4,9036E-06	1,5882E-05
YMR140W	2121,59	0,63	-0,67	-4,56676982	4,953E-06	1,6033E-05
YMR295C	3906,32	0,64	-0,64	-4,55660258	5,1988E-06	1,682E-05
YGR113W	1492,6	1,65	0,73	4,55434448	5,2549E-06	1,6993E-05
YLR080W	948,57	0,58	-0,79	-4,55242993	5,303E-06	1,714E-05
YGL174W	645,29	1,78	0,83	4,55058634	5,3497E-06	1,7281E-05
YGL113W	1011,09	1,67	0,74	4,54992239	5,3666E-06	1,7322E-05
YMR199W	1391,48	0,58	-0,8	-4,54987608	5,3678E-06	1,7322E-05
YNL234W	868,86	1,7	0,76	4,54967019	5,373E-06	1,733E-05
YPL219W	524,85	0,56	-0,83	-4,54923231	5,3842E-06	1,7357E-05
YBR249C	14580,67	1,54	0,62	4,54755489	5,4273E-06	1,7487E-05
YOL098C	2886,03	0,52	-0,93	-4,54667057	5,4501E-06	1,7551E-05
YLR001C	1570,38	0,62	-0,68	-4,5389509	5,6535E-06	1,8195E-05
YLR144C	1079,95	0,6	-0,75	-4,53885713	5,656E-06	1,8195E-05
YOR389W	487,82	1,79	0,84	4,53752816	5,6917E-06	1,8301E-05
YMR037C	2522,6	1,7	0,77	4,53443901	5,7757E-06	1,8561E-05
YBR212W	3542,19	0,65	-0,62	-4,53409879	5,785E-06	1,8575E-05
YDR156W	588,63	0,55	-0,86	-4,53406309	5,786E-06	1,8575E-05
YJR044C	2820,87	1,57	0,65	4,53090009	5,8733E-06	1,8845E-05
YDL178W	2242,17	0,58	-0,8	-4,52693366	5,9846E-06	1,9193E-05
YAR002C-A	3471,81	0,64	-0,65	-4,52662205	5,9934E-06	1,9211E-05
YJR108W	105,75	0,27	-1,89	-4,52635046	6,0011E-06	1,9226E-05
YGL155W	1405,96	0,59	-0,76	-4,52514215	6,0355E-06	1,9326E-05
YLR173W	769,74	0,57	-0,81	-4,52480919	6,045E-06	1,9346E-05
YLR276C	3590,24	1,92	0,94	4,52098558	6,1552E-06	1,9689E-05
YBR255C-A	725,91	1,93	0,95	4,51957221	6,1965E-06	1,9811E-05

YGL085W	626,1	1,82	0,86	4,51789008	6,2459E-06	1,9959E-05
YBR223C	744,91	0,58	-0,79	-4,51503331	6,3307E-06	2,0219E-05
YNL162W	7933,2	1,63	0,71	4,51315698	6,387E-06	2,0388E-05
YDR425W	2510,43	1,55	0,63	4,51223789	6,4147E-06	2,0466E-05
YGR279C	14297,62	1,5	0,59	4,50901647	6,5129E-06	2,0769E-05
YMR265C	1645,91	1,62	0,7	4,5057177	6,6149E-06	2,1084E-05
YNL242W	1343,6	0,62	-0,68	-4,50498387	6,6378E-06	2,1146E-05
YDR117C	1593,98	0,59	-0,75	-4,50288628	6,7037E-06	2,1345E-05
YJL093C	1226,64	0,62	-0,69	-4,49623358	6,9168E-06	2,2012E-05
YBR133C	1903,8	0,64	-0,65	-4,49475686	6,9649E-06	2,2154E-05
YLR182W	1876,45	0,61	-0,71	-4,48910316	7,1524E-06	2,2738E-05
YKL112W	1409,68	0,63	-0,67	-4,4834539	7,3444E-06	2,3337E-05
YIL014W	1355,98	0,62	-0,7	-4,48294647	7,3619E-06	2,3381E-05
YKL218C	1144,61	0,61	-0,72	-4,48178196	7,4022E-06	2,3497E-05
YCR038C	471,78	0,55	-0,87	-4,47822071	7,5268E-06	2,388E-05
YOR162C	1717,64	1,57	0,65	4,47636241	7,5926E-06	2,4076E-05
YJL186W	1577,02	0,63	-0,67	-4,47464003	7,654E-06	2,4259E-05
YDR330W	2006,51	0,64	-0,64	-4,47403953	7,6756E-06	2,4314E-05
YJR125C	3513,12	0,65	-0,61	-4,47243458	7,7334E-06	2,4485E-05
YGL169W	828,47	1,71	0,77	4,47015109	7,8164E-06	2,4735E-05
YKL213C	3794,95	0,66	-0,61	-4,46702748	7,9314E-06	2,5086E-05
YCL049C	1437,15	0,58	-0,79	-4,46135696	8,1442E-06	2,5747E-05
YDL147W	11152,23	1,6	0,67	4,46052039	8,1761E-06	2,5831E-05
YOR273C	3623,2	0,58	-0,8	-4,46043565	8,1793E-06	2,5831E-05
YML055W	1128,44	0,61	-0,72	-4,45818927	8,2655E-06	2,6077E-05
YNCI0012	274,02	0,47	-1,1	-4,45824429	8,2634E-06	2,6077E-05
YHR190W	6083,95	0,65	-0,62	-4,45639236	8,335E-06	2,6283E-05
YDR018C	274,16	0,34	-1,54	-4,45545034	8,3717E-06	2,6385E-05
YBR196C-B	173,02	2,49	1,31	4,45487204	8,3943E-06	2,6443E-05
YNCD0010C	68,71	3,91	1,97	4,45380086	8,4363E-06	2,6562E-05
YMR123W	1113,24	0,6	-0,73	-4,45328436	8,4567E-06	2,6612E-05
YGR185C	7414,02	0,61	-0,72	-4,44857513	8,6442E-06	2,7189E-05
YLR283W	651,68	0,57	-0,82	-4,44532499	8,7759E-06	2,7589E-05
YOL100W	1905,46	0,63	-0,67	-4,44459415	8,8058E-06	2,7669E-05
YBR024W	1012,34	0,58	-0,8	-4,44332796	8,8578E-06	2,7818E-05
YHR171W	830,56	0,58	-0,77	-4,44316768	8,8644E-06	2,7825E-05
YKR037C	682,35	0,53	-0,91	-4,4427932	8,8799E-06	2,7859E-05
YMR022W	1777,88	0,62	-0,68	-4,44251515	8,8913E-06	2,7881E-05
YGR216C	729,79	0,57	-0,81	-4,43484636	9,2138E-06	2,8878E-05
YPR181C	6513,12	0,61	-0,71	-4,43465971	9,2218E-06	2,8888E-05
YOR264W	1549,7	0,61	-0,72	-4,43235159	9,3211E-06	2,9185E-05
YOR362C	5012,14	0,67	-0,58	-4,43220184	9,3276E-06	2,919E-05
YEL042W	3103,87	0,64	-0,65	-4,42171708	9,792E-06	3,0628E-05
YMR231W	1058,01	0,55	-0,87	-4,41404564	1,0146E-05	3,1719E-05
YJL052W	111092,16	2,06	1,04	4,412131	1,0236E-05	3,1984E-05
YNL253W	846,23	1,64	0,71	4,41091602	1,0293E-05	3,2132E-05
YOR380W	441,74	0,54	-0,88	-4,41091985	1,0293E-05	3,2132E-05
YDR411C	1605,67	0,57	-0,81	-4,41006684	1,0334E-05	3,2242E-05
YOR144C	446,35	0,54	-0,88	-4,40898016	1,0386E-05	3,2388E-05
YMR230W	10870,08	0,67	-0,58	-4,40436716	1,0609E-05	3,3068E-05

YPR107C	1061,9	1,72	0,78	4,39866506	1,0892E-05	3,3932E-05
YMR087W	551,34	0,54	-0,9	-4,39745487	1,0953E-05	3,4105E-05
YKL150W	8470,1	0,51	-0,97	-4,39577332	1,1038E-05	3,4352E-05
YGR267C	3108,15	0,65	-0,61	-4,3955896	1,1047E-05	3,4364E-05
YLR262C	295,41	1,92	0,94	4,39545735	1,1054E-05	3,4368E-05
YLR300W	6653,82	0,6	-0,74	-4,39528792	1,1063E-05	3,4378E-05
YGR202C	3123,35	1,54	0,62	4,39406008	1,1125E-05	3,4555E-05
YPL038W	579,58	1,74	0,8	4,39282764	1,1189E-05	3,4734E-05
YDR233C	9556,63	0,67	-0,59	-4,38969952	1,1351E-05	3,522E-05
YNL127W	1529,43	0,64	-0,65	-4,38373089	1,1666E-05	3,6182E-05
YPR081C	1928,4	1,54	0,62	4,37876094	1,1936E-05	3,6998E-05
YOR074C	1185,37	0,63	-0,67	-4,37680656	1,2043E-05	3,7313E-05
YDR449C	674,93	1,87	0,91	4,37271453	1,2271E-05	3,7981E-05
YMR060C	809,74	1,72	0,78	4,37271999	1,2271E-05	3,7981E-05
YEL054C	15349,86	0,61	-0,72	-4,37178999	1,2323E-05	3,8123E-05
YOR334W	575,12	1,72	0,78	4,37106923	1,2364E-05	3,823E-05
YIL037C	234,25	0,48	-1,07	-4,36964645	1,2445E-05	3,8461E-05
YPR168W	394,73	2,02	1,01	4,36755149	1,2565E-05	3,8813E-05
YEL016C	907,54	0,59	-0,75	-4,36450301	1,2741E-05	3,9338E-05
YJR059W	6601,52	0,67	-0,57	-4,36225012	1,2873E-05	3,9726E-05
YPL043W	2520,13	2	1	4,35295465	1,3431E-05	4,1428E-05
YGL224C	1033,48	1,59	0,67	4,35137751	1,3528E-05	4,1707E-05
YCR028C	658,79	1,7	0,76	4,34846305	1,3709E-05	4,2244E-05
YBR201W	849,76	0,59	-0,77	-4,34803187	1,3736E-05	4,2306E-05
YIR022W	1566,19	0,61	-0,71	-4,34570623	1,3883E-05	4,2735E-05
YIL139C	671,66	1,69	0,76	4,34519176	1,3915E-05	4,2815E-05
YDR458C	2453,47	1,54	0,62	4,34472317	1,3945E-05	4,2885E-05
YHR214W	142,63	2,34	1,23	4,34250964	1,4086E-05	4,3298E-05
YJR002W	3176,47	1,75	0,81	4,3389704	1,4315E-05	4,3979E-05
YDL103C	2220,1	0,65	-0,63	-4,3359438	1,4514E-05	4,4567E-05
YGL226W	406,92	0,55	-0,87	-4,33460555	1,4602E-05	4,4772E-05
YOR045W	1423,12	1,76	0,81	4,33470902	1,4595E-05	4,4772E-05
YPL067C	376,81	0,53	-0,91	-4,33473773	1,4593E-05	4,4772E-05
YNL133C	507,8	1,81	0,86	4,33123888	1,4827E-05	4,544E-05
YOL126C	2607,79	0,63	-0,68	-4,33108979	1,4837E-05	4,5448E-05
YDR380W	1958,04	0,38	-1,39	-4,32896709	1,4981E-05	4,5866E-05
YJL142C	127,65	3,04	1,6	4,32624986	1,5167E-05	4,6412E-05
YML053C	1420,81	1,65	0,72	4,32463813	1,5278E-05	4,673E-05
YER152C	2350,53	0,58	-0,79	-4,32255966	1,5423E-05	4,7149E-05
YPR027C	400,98	0,44	-1,2	-4,32168348	1,5484E-05	4,7314E-05
YOR328W	825,76	1,66	0,73	4,31907476	1,5668E-05	4,7853E-05
YNL085W	5589,47	0,58	-0,8	-4,31896182	1,5676E-05	4,7854E-05
YDL170W	1052,13	1,67	0,74	4,31582167	1,5901E-05	4,8515E-05
YNL283C	1198,87	0,63	-0,67	-4,31230572	1,6156E-05	4,9269E-05
YGR117C	1220,46	0,62	-0,69	-4,31099091	1,6252E-05	4,9539E-05
YDR224C	8621,7	0,68	-0,56	-4,30950895	1,6362E-05	4,9848E-05
YJR123W	39441,49	0,62	-0,69	-4,30922531	1,6383E-05	4,9887E-05
YDR315C	349,71	0,48	-1,04	-4,30829994	1,6451E-05	5,0072E-05
YIL024C	332,56	2,01	1,01	4,3068718	1,6558E-05	5,0371E-05
YLR027C	8778,12	0,68	-0,57	-4,30376204	1,6792E-05	5,1059E-05

YOL052C	1943,17	1,53	0,61	4,30270906	1,6872E-05	5,1277E-05
YJR094C	420,82	0,56	-0,84	-4,29838926	1,7204E-05	5,2257E-05
YKR002W	2018,14	0,65	-0,61	-4,29829804	1,7211E-05	5,2257E-05
YNL239W	5965,72	1,56	0,64	4,2962053	1,7375E-05	5,2727E-05
YDL008W	479,49	0,55	-0,85	-4,29254665	1,7664E-05	5,3577E-05
YLR372W	3467,28	0,55	-0,87	-4,29200661	1,7707E-05	5,3681E-05
YDL136W	6556,82	1,68	0,75	4,29188138	1,7717E-05	5,3685E-05
YGL258W-A	69,27	0,28	-1,83	-4,28881456	1,7963E-05	5,4406E-05
YCR014C	847,36	1,63	0,7	4,28796788	1,8032E-05	5,4587E-05
YOR357C	1092,81	1,65	0,72	4,28481446	1,8289E-05	5,534E-05
YGL246C	1192,39	1,57	0,65	4,28460517	1,8306E-05	5,5365E-05
YNL025C	861,52	0,6	-0,74	-4,27797994	1,886E-05	5,701E-05
YDR169C	2288,11	0,64	-0,65	-4,27663058	1,8974E-05	5,7329E-05
YKR044W	921,91	1,61	0,69	4,27366936	1,9228E-05	5,8068E-05
YFL039C	68806,84	1,48	0,56	4,27102069	1,9458E-05	5,8733E-05
YJR043C	613,34	0,52	-0,95	-4,26865063	1,9666E-05	5,9332E-05
YEL027W	12637,4	0,68	-0,55	-4,26654445	1,9852E-05	5,9866E-05
YOL076W	851,52	0,51	-0,96	-4,26542135	1,9953E-05	6,0138E-05
YKR091W	1514,17	1,57	0,65	4,26419733	2,0062E-05	6,044E-05
YLR209C	1851,09	1,59	0,67	4,26406433	2,0074E-05	6,0446E-05
YML036W	455,76	0,57	-0,82	-4,26275081	2,0193E-05	6,0773E-05
YHR097C	5744,91	0,62	-0,68	-4,26127289	2,0327E-05	6,1147E-05
YPL115C	1611,37	0,55	-0,86	-4,25940831	2,0497E-05	6,163E-05
YHR111W	1275,91	1,56	0,65	4,25213539	2,1174E-05	6,3635E-05
YOR347C	2082,59	0,65	-0,61	-4,24980177	2,1396E-05	6,4271E-05
YJL129C	2673,73	1,54	0,62	4,24766265	2,1601E-05	6,4856E-05
YGR007W	1607,13	0,65	-0,62	-4,24754034	2,1613E-05	6,486E-05
YDR121W	1025,42	1,63	0,71	4,24693815	2,1671E-05	6,5003E-05
YDR294C	3761,3	0,66	-0,59	-4,24553515	2,1807E-05	6,5348E-05
YPL184C	1846,03	0,64	-0,63	-4,24557378	2,1803E-05	6,5348E-05
YHR205W	1451,7	0,58	-0,78	-4,2447842	2,188E-05	6,5536E-05
YOR332W	12801,83	0,67	-0,57	-4,2440297	2,1954E-05	6,5725E-05
YIL145C	2009,54	0,65	-0,61	-4,22986066	2,3384E-05	6,9971E-05
YKL196C	3162,58	0,67	-0,57	-4,22897215	2,3476E-05	7,0214E-05
YHR197W	1347,08	2,08	1,06	4,22687685	2,3696E-05	7,0836E-05
YNR043W	1763,55	0,59	-0,76	-4,22595012	2,3793E-05	7,1094E-05
YDR407C	1987,26	0,61	-0,71	-4,22578649	2,3811E-05	7,1112E-05
YLR121C	1516,58	0,6	-0,73	-4,22404925	2,3995E-05	7,1628E-05
YDR372C	3459,65	0,65	-0,63	-4,22241303	2,417E-05	7,2115E-05
YDL142C	932,95	0,61	-0,7	-4,21955539	2,4478E-05	7,3001E-05
YBR115C	3345,05	1,66	0,73	4,21876569	2,4564E-05	7,3222E-05
YNL256W	1673,89	1,69	0,76	4,21827011	2,4618E-05	7,3347E-05
YMR298W	2275,12	0,64	-0,65	-4,2171073	2,4746E-05	7,3691E-05
YFR051C	5064,81	0,64	-0,65	-4,2169017	2,4768E-05	7,3723E-05
YLR360W	941,81	0,63	-0,68	-4,21605452	2,4861E-05	7,3965E-05
YNL068C	1545	0,63	-0,67	-4,21543981	2,4929E-05	7,4131E-05
YDR192C	571,73	0,58	-0,78	-4,21478583	2,5002E-05	7,4311E-05
YDR469W	680,53	1,65	0,73	4,21006579	2,553E-05	7,5808E-05
YJL001W	6305,56	1,46	0,55	4,21014363	2,5521E-05	7,5808E-05
YML052W	1684,65	0,58	-0,79	-4,20922717	2,5625E-05	7,6053E-05

YDL077C	1398,35	0,58	-0,78	-4,20800325	2,5764E-05	7,6393E-05
YGL043W	2837,23	1,52	0,6	4,20810244	2,5752E-05	7,6393E-05
YML060W	406,19	0,49	-1,02	-4,20676579	2,5905E-05	7,6776E-05
YEL048C	141,06	2,26	1,17	4,20225239	2,6427E-05	7,8286E-05
YGL100W	2128,22	0,66	-0,6	-4,20112438	2,6559E-05	7,8621E-05
YGR071C	819,16	0,58	-0,77	-4,2010718	2,6565E-05	7,8621E-05
YGR082W	1857,35	0,6	-0,73	-4,1995791	2,6741E-05	7,9103E-05
YAR033W	427,28	0,53	-0,91	-4,19579074	2,7192E-05	8,0399E-05
YLR254C	775,63	1,69	0,76	4,19488273	2,7301E-05	8,0683E-05
YBR194W	445,82	0,55	-0,87	-4,18860498	2,8067E-05	8,2908E-05
YNL284C	2456	1,66	0,73	4,1875034	2,8204E-05	8,3271E-05
YER068W	3026,54	1,55	0,63	4,18473203	2,855E-05	8,4254E-05
YOR064C	829,09	1,88	0,91	4,18341099	2,8717E-05	8,4705E-05
YER100W	3503,99	1,49	0,58	4,18286615	2,8786E-05	8,4868E-05
YGR224W	595,09	0,58	-0,8	-4,18194444	2,8903E-05	8,5172E-05
YDR274C	97,3	3,05	1,61	4,18101095	2,9022E-05	8,5482E-05
YML063W	30709,93	0,6	-0,73	-4,17831014	2,9368E-05	8,6463E-05
YCL001W	1841,92	0,64	-0,64	-4,17799956	2,9408E-05	8,654E-05
YHR189W	418,84	0,49	-1,03	-4,17426137	2,9895E-05	8,7896E-05
YLR039C	1523,56	0,6	-0,75	-4,17424432	2,9898E-05	8,7896E-05
YMR168C	775,62	0,61	-0,72	-4,17234006	3,0149E-05	8,8592E-05
YIL070C	1812,3	0,62	-0,69	-4,16651763	3,0929E-05	9,0841E-05
YGR054W	3334,19	0,58	-0,79	-4,16582148	3,1023E-05	9,1076E-05
YLR096W	2177,98	1,53	0,61	4,16514257	3,1116E-05	9,1304E-05
Q0140	14,44	28,88	4,85	4,16492442	3,1146E-05	9,1349E-05
YPL260W	4840,39	1,48	0,56	4,16436812	3,1222E-05	9,1528E-05
YJL038C	82,54	0,33	-1,61	-4,16272073	3,1448E-05	9,2148E-05
YJR032W	875,75	0,58	-0,78	-4,16100174	3,1685E-05	9,2801E-05
YLR233C	420,34	0,56	-0,83	-4,1582763	3,2066E-05	9,387E-05
YOR202W	1326,47	0,53	-0,9	-4,15579512	3,2416E-05	9,485E-05
YDR384C	1351,5	1,86	0,9	4,1526458	3,2865E-05	9,612E-05
YKR097W	267,36	1,94	0,96	4,14788574	3,3556E-05	9,8094E-05
YPR041W	4927,9	0,66	-0,6	-4,14716293	3,3662E-05	9,8358E-05
YNCM0013W	52,64	0,25	-2	-4,14676356	3,3721E-05	9,8483E-05
YDL036C	1203,05	1,54	0,63	4,14484579	3,4004E-05	9,9264E-05
YMR235C	4128,39	0,64	-0,64	-4,14354017	3,4199E-05	9,9784E-05
YMR031C	6064	0,66	-0,6	-4,14140413	3,4519E-05	0,00010067
YPL040C	1025,89	0,58	-0,79	-4,14021301	3,4698E-05	0,00010115
YGR165W	1511,63	0,63	-0,67	-4,13396419	3,5656E-05	0,00010389
YLR379W-A	18,89	10,67	3,42	4,13288934	3,5823E-05	0,00010433
YOL129W	2713,27	0,62	-0,68	-4,13255	3,5876E-05	0,00010443
YBL013W	171,49	0,46	-1,12	-4,1307965	3,6151E-05	0,00010514
YGR136W	5932,92	1,48	0,57	4,13080112	3,615E-05	0,00010514
YOL065C	1170,22	1,58	0,66	4,12983718	3,6302E-05	0,00010553
YDL144C	1222,02	0,63	-0,66	-4,12885274	3,6458E-05	0,00010593
YBL059W	245,18	0,47	-1,09	-4,12735875	3,6695E-05	0,00010657
YBR142W	2135,21	1,76	0,81	4,12520832	3,704E-05	0,00010752
YLR422W	1467,56	0,65	-0,62	-4,124681	3,7125E-05	0,00010772
YOL091W	103,1	2,55	1,35	4,119941	3,7897E-05	0,0001099
YNL112W	5297,12	2,01	1	4,11871354	3,8099E-05	0,00011044

YPR066W	666,8	1,81	0,85	4,1145497	3,8794E-05	0,0001124
YIL083C	1125,58	0,64	-0,65	-4,1140001	3,8886E-05	0,00011262
YDR468C	1636,29	0,66	-0,6	-4,10862074	3,9803E-05	0,00011522
YML003W	309,16	0,54	-0,89	-4,10820843	3,9874E-05	0,00011537
YNR017W	3332	1,51	0,59	4,10757304	3,9984E-05	0,00011563
YER171W	1279,93	0,63	-0,67	-4,10595449	4,0265E-05	0,00011639
YER064C	2046,88	0,64	-0,63	-4,10386463	4,0631E-05	0,00011739
YIR005W	441,34	2,13	1,09	4,10320871	4,0746E-05	0,00011767
YNL261W	1320,43	1,57	0,65	4,10289031	4,0802E-05	0,00011778
YPL177C	1993,32	0,65	-0,62	-4,0986038	4,1565E-05	0,00011993
YOR260W	3542,09	0,58	-0,79	-4,09678781	4,1892E-05	0,00012081
YOR129C	484,73	0,58	-0,78	-4,09484985	4,2244E-05	0,00012177
YDR209C	398,46	2,12	1,08	4,09296239	4,259E-05	0,00012271
YEL062W	641,62	0,6	-0,73	-4,09225116	4,2721E-05	0,00012301
YNR071C	206,74	2,27	1,18	4,09218103	4,2733E-05	0,00012301
YJR093C	968,11	0,64	-0,65	-4,08854717	4,3408E-05	0,0001249
YKR008W	2703,92	1,53	0,62	4,08673356	4,3749E-05	0,00012582
YDL199C	643,96	0,55	-0,87	-4,086199	4,385E-05	0,00012605
YEL020C	859,26	0,6	-0,73	-4,08447417	4,4177E-05	0,00012693
YLL011W	604,55	1,99	1	4,07915529	4,52E-05	0,00012981
Q0110	8,74	147,79	7,21	4,07814894	4,5396E-05	0,00013031
YNL058C	1711,15	0,65	-0,62	-4,0774864	4,5525E-05	0,00013062
YBR059C	4386,1	0,66	-0,6	-4,07668781	4,5682E-05	0,00013101
YDR387C	1151,7	0,62	-0,69	-4,07598525	4,582E-05	0,00013135
YDR082W	716,89	1,64	0,71	4,07562525	4,5891E-05	0,00013149
YOR341W	4420,41	1,88	0,91	4,07302187	4,6407E-05	0,00013291
YML001W	2022,01	0,66	-0,6	-4,0705986	4,6892E-05	0,00013424
YCR099C	247,98	1,91	0,93	4,0695926	4,7095E-05	0,00013476
YER174C	592,45	0,6	-0,74	-4,06936831	4,7141E-05	0,00013482
YCR094W	1351,97	0,61	-0,7	-4,06688171	4,7646E-05	0,00013621
YDR545W	37,48	0,18	-2,47	-4,06612362	4,7802E-05	0,00013659
YER061C	1330,21	0,65	-0,61	-4,06569151	4,789E-05	0,00013678
YDR501W	500,54	1,71	0,77	4,06334604	4,8374E-05	0,0001381
YLR059C	1290,68	0,65	-0,63	-4,06222672	4,8607E-05	0,0001387
YNL293W	1295,58	1,52	0,6	4,05979937	4,9115E-05	0,00014008
YLR264C-A	2212,5	0,51	-0,98	-4,05920105	4,9241E-05	0,00014038
YOR171C	2584,14	1,48	0,57	4,05836706	4,9417E-05	0,00014082
YDR070C	2939,76	0,17	-2,52	-4,05718811	4,9667E-05	0,00014146
YNL320W	482,78	0,56	-0,83	-4,05464163	5,0211E-05	0,00014295
YCL037C	371,43	0,53	-0,93	-4,05263456	5,0644E-05	0,00014411
YHR109W	394,02	0,56	-0,84	-4,0516455	5,0859E-05	0,00014466
YDR182W-A	73,73	2,88	1,53	4,0508105	5,1041E-05	0,00014511
YPL271W	2963,03	0,5	-0,99	-4,04978564	5,1265E-05	0,00014568
YHR183W	13169,13	0,67	-0,58	-4,0491897	5,1395E-05	0,00014598
YOR011W	667	1,77	0,83	4,04835498	5,1579E-05	0,00014644
YGR020C	5254,18	0,68	-0,56	-4,043912	5,2567E-05	0,00014918
YIL009C-A	470,94	0,54	-0,88	-4,04214948	5,2963E-05	0,00015023
YPR062W	4893,29	0,69	-0,54	-4,04156527	5,3096E-05	0,00015054
YIL091C	987,72	1,61	0,69	4,04136281	5,3141E-05	0,0001506
YER161C	2035,77	1,49	0,57	4,04116614	5,3186E-05	0,00015066

YJL083W	655,96	0,61	-0,71	-4,04036727	5,3368E-05	0,0001511
YPR019W	1669,05	0,63	-0,66	-4,03922113	5,3629E-05	0,00015177
YGR001C	2191,6	1,5	0,58	4,03714938	5,4105E-05	0,00015305
Q0060	13,78	42,11	5,4	4,03297891	5,5074E-05	0,00015572
YHR067W	634,05	0,61	-0,72	-4,02982065	5,5819E-05	0,00015776
YML064C	647,79	0,57	-0,81	-4,02803424	5,6245E-05	0,00015889
YDR541C	784,55	0,62	-0,7	-4,02702149	5,6488E-05	0,0001595
YMR229C	3510,3	1,93	0,94	4,02015094	5,8161E-05	0,00016415
YGL194C-A	391,69	0,54	-0,9	-4,0194328	5,8338E-05	0,00016458
YJR016C	5970,23	0,59	-0,77	-4,01880741	5,8493E-05	0,00016494
YJR013W	1050,2	0,62	-0,69	-4,01773983	5,8759E-05	0,00016561
YEL050C	1867,77	0,61	-0,71	-4,01677803	5,8999E-05	0,00016621
YKL087C	2206,52	1,63	0,7	4,0162487	5,9132E-05	0,00016651
YBR033W	464,79	0,49	-1,04	-4,01544801	5,9333E-05	0,000167
YGL040C	2754,46	0,67	-0,58	-4,01524664	5,9384E-05	0,00016707
YIL082W-A	3078,47	1,46	0,54	4,01456492	5,9556E-05	0,00016748
YLL010C	1561,39	0,65	-0,63	-4,01261168	6,0051E-05	0,00016879
YIL031W	1702,65	1,55	0,64	4,0105537	6,0577E-05	0,0001702
YKR009C	851,19	0,55	-0,87	-4,00464116	6,2112E-05	0,00017443
YNL003C	999,82	0,64	-0,64	-4,0037542	6,2345E-05	0,00017501
YGR161W-C	663,85	1,73	0,79	4,00280455	6,2596E-05	0,00017563
YLR013W	16,18	15,8	3,98	4,00197075	6,2817E-05	0,00017617
YAL054C	685,41	1,86	0,9	3,9997681	6,3405E-05	0,00017774
YPL074W	1730,43	1,51	0,59	3,99948505	6,348E-05	0,00017787
YPR169W	3331,2	1,67	0,74	3,99840168	6,3772E-05	0,00017861
YBR061C	780,37	0,63	-0,68	-3,99708876	6,4126E-05	0,00017952
YLR286C	22859,34	0,69	-0,54	-3,99660269	6,4258E-05	0,00017981
YPL242C	2649,28	1,52	0,61	3,99303126	6,5234E-05	0,00018246
YOL105C	766,98	1,6	0,68	3,99189068	6,5549E-05	0,00018325
YDL186W	93,53	0,32	-1,62	-3,99046992	6,5943E-05	0,00018419
YLR088W	1346,76	0,65	-0,63	-3,99051483	6,593E-05	0,00018419
YKL023W	1675,68	1,56	0,64	3,98975014	6,6143E-05	0,00018467
YNL159C	1045,02	0,61	-0,71	-3,98955637	6,6197E-05	0,00018473
YMR042W	370,59	1,79	0,84	3,9850363	6,747E-05	0,0001882
YNCP0013C	50,36	0,23	-2,13	-3,98248272	6,8199E-05	0,00019015
YKL109W	8901,48	0,7	-0,52	-3,98013958	6,8875E-05	0,00019195
YLR417W	925,07	0,62	-0,69	-3,97918859	6,9151E-05	0,00019263
YNL265C	1916,96	1,56	0,64	3,97721906	6,9726E-05	0,00019414
YIL137C	3509,78	0,66	-0,6	-3,97687434	6,9827E-05	0,00019432
YNCI0006W	24,32	7,44	2,9	3,97679072	6,9852E-05	0,00019432
YGL173C	8382,59	0,64	-0,64	-3,97518668	7,0324E-05	0,00019555
YLR084C	806,63	1,65	0,72	3,97360708	7,0792E-05	0,00019676
YCR101C	321,75	1,76	0,82	3,97254698	7,1108E-05	0,00019755
YNL138W-A	255,74	1,87	0,91	3,97075152	7,1646E-05	0,00019896
YDL237W	2539,24	0,64	-0,64	-3,96521353	7,333E-05	0,00020354
YDR164C	1735,38	0,67	-0,58	-3,96510178	7,3365E-05	0,00020355
YMR205C	19595,45	1,73	0,79	3,96368206	7,3803E-05	0,00020467
YHR043C	2520,21	1,54	0,62	3,96315082	7,3967E-05	0,00020503
YGR256W	768,49	0,15	-2,74	-3,96079086	7,4702E-05	0,00020698
YCR028C-A	4468,77	0,7	-0,52	-3,95706913	7,5875E-05	0,00021014

YGL025C	990,56	0,63	-0,67	-3,95693285	7,5918E-05	0,00021016
YDR379C-A	373,69	0,5	-1,01	-3,95527857	7,6446E-05	0,00021153
YGL039W	1807,46	1,72	0,78	3,95417438	7,6799E-05	0,00021241
YPR022C	1461,84	0,66	-0,6	-3,95300547	7,7176E-05	0,00021336
YLR418C	1495,19	0,67	-0,59	-3,95180836	7,7563E-05	0,00021433
YKL126W	4048,67	0,69	-0,53	-3,9502346	7,8075E-05	0,00021565
YNL243W	4858,34	0,65	-0,62	-3,94996592	7,8162E-05	0,0002158
YNL061W	3976,38	1,68	0,75	3,94871394	7,8572E-05	0,00021683
YPL259C	2660,04	1,48	0,56	3,94518306	7,9739E-05	0,00021996
YIL127C	855,23	1,77	0,82	3,94505894	7,978E-05	0,00021997
YDL244W	75,41	2,75	1,46	3,94286887	8,0513E-05	0,00022189
YLR193C	912,25	0,55	-0,86	-3,93708431	8,2478E-05	0,00022721
YOR161C-C	38,64	4,72	2,24	3,93406449	8,3521E-05	0,00022998
YIR033W	3011,67	1,64	0,71	3,93391595	8,3573E-05	0,00023002
YKR031C	2758,17	1,5	0,59	3,93352571	8,3709E-05	0,00023029
YMR208W	2345,96	0,68	-0,57	-3,93244868	8,4085E-05	0,00023122
YPL249C	3306,62	1,49	0,57	3,93009341	8,4913E-05	0,0002334
YLR376C	245,46	0,52	-0,93	-3,92474423	8,6822E-05	0,00023854
YDR118W	650,26	0,62	-0,7	-3,92441316	8,6941E-05	0,00023876
YHL049C	25,56	6,55	2,71	3,91507893	9,0375E-05	0,00024808
YMR277W	2296,84	0,66	-0,6	-3,91468734	9,0521E-05	0,00024837
YKL042W	1156,61	1,55	0,63	3,91199382	9,1537E-05	0,00025105
YIR004W	2651,09	1,45	0,54	3,91123186	9,1827E-05	0,00025173
YNL088W	4474,91	1,58	0,66	3,90832616	9,2938E-05	0,00025467
YDR540C	424,09	1,68	0,75	3,90663546	9,359E-05	0,00025634
YJR047C	756,02	1,58	0,66	3,90583386	9,3901E-05	0,00025708
YDL092W	1601,95	0,63	-0,66	-3,90495529	9,4243E-05	0,0002579
YDL205C	2072,19	0,65	-0,62	-3,90371384	9,4728E-05	0,00025911
YHR011W	831,65	0,62	-0,68	-3,9024665	9,5217E-05	0,00026034
YER046W	299,22	1,84	0,88	3,90207252	9,5373E-05	0,00026065
YIL052C	12759,56	1,48	0,56	3,90195608	9,5418E-05	0,00026066
YDR160W	1112,71	1,52	0,6	3,90144412	9,5621E-05	0,0002611
YLR148W	2273,17	1,48	0,57	3,90095107	9,5816E-05	0,00026151
YCR042C	2790,84	1,5	0,58	3,89874215	9,6694E-05	0,00026379
YDR312W	949,85	1,68	0,75	3,89838034	9,6838E-05	0,00026407
YBL084C	1187,34	1,52	0,6	3,89594316	9,7817E-05	0,00026663
YKL070W	576,52	1,67	0,74	3,88983602	0,00010031	0,00027331
YFR022W	1092,88	0,64	-0,65	-3,88750946	0,00010128	0,00027582
YGL002W	1399,62	1,57	0,65	3,88548691	0,00010212	0,000278
YFL008W	2407,03	1,6	0,67	3,88260763	0,00010334	0,00028119
YKR060W	806,59	1,57	0,65	3,87859581	0,00010506	0,00028574
YLL034C	1057,28	1,98	0,99	3,87729175	0,00010563	0,00028715
YKR007W	1093,69	1,51	0,6	3,87710751	0,00010571	0,00028725
YNR046W	850,83	0,64	-0,65	-3,87631808	0,00010605	0,00028805
YFL049W	1673,65	1,48	0,57	3,87314478	0,00010744	0,0002917
YDL229W	35881,96	0,58	-0,79	-3,86846533	0,00010952	0,00029723
YNCJ0023C	13,5	82,87	6,37	3,86715219	0,00011011	0,0002987
YKR017C	799,01	0,63	-0,68	-3,86671624	0,00011031	0,00029911
YCL073C	12,45	18,24	4,19	3,86557818	0,00011083	0,00030037
YHR144C	484,55	0,54	-0,88	-3,86429602	0,00011141	0,00030182

YJL131C	1135,83	0,64	-0,65	-3,86302498	0,00011199	0,00030327
YLR180W	9231,45	0,7	-0,52	-3,86254696	0,00011221	0,00030373
YNL169C	2240,94	0,67	-0,57	-3,86240336	0,00011228	0,00030377
YDL117W	1136	0,63	-0,66	-3,85983999	0,00011346	0,00030658
YDR119W	1743,99	0,62	-0,69	-3,8598735	0,00011345	0,00030658
YPL139C	1292,66	0,66	-0,59	-3,86000875	0,00011338	0,00030658
YGR067C	415,25	2,08	1,06	3,85918552	0,00011377	0,00030727
YGR068C	701,65	0,62	-0,69	-3,85884618	0,00011392	0,00030756
YDR343C	4576,42	0,44	-1,17	-3,85741359	0,00011459	0,00030912
YMR193W	1941,77	1,51	0,6	3,8573955	0,0001146	0,00030912
YFL052W	368,31	0,44	-1,18	-3,85629301	0,00011512	0,00031038
YJL003W	462,43	1,66	0,73	3,85183819	0,00011723	0,00031581
YLR432W	7747,76	0,63	-0,68	-3,8519011	0,0001172	0,00031581
YNL023C	1039,94	1,55	0,63	3,85062595	0,00011782	0,00031724
YGL031C	16454,86	0,67	-0,57	-3,8503714	0,00011794	0,00031743
YNCO0022C	122,34	2,28	1,19	3,85014214	0,00011805	0,00031759
YLR091W	368,34	0,57	-0,8	-3,85001833	0,00011811	0,00031762
YAL032C	877,25	1,55	0,63	3,84961338	0,0001183	0,000318
YGL082W	3016,96	0,69	-0,55	-3,8487064	0,00011874	0,00031905
YMR307W	16856,98	0,67	-0,57	-3,84781464	0,00011918	0,00032007
YKR106W	274,98	0,52	-0,95	-3,84585953	0,00012013	0,00032249
YLR365W	20,73	7	2,81	3,84562684	0,00012024	0,00032266
YDL106C	1982,13	1,47	0,56	3,8451563	0,00012048	0,00032314
YNR013C	2948,57	0,64	-0,65	-3,84486941	0,00012062	0,00032338
YBL106C	951,63	1,62	0,69	3,84327791	0,0001214	0,00032534
YKR095W-A	496,15	1,62	0,7	3,84040716	0,00012283	0,00032903
YOR280C	1332,9	1,56	0,64	3,83924462	0,00012341	0,00033045
YBR203W	1235,55	1,49	0,57	3,83548839	0,00012532	0,0003354
YPL046C	308,46	0,51	-0,96	-3,83427098	0,00012594	0,00033692
YPR047W	1127,11	0,63	-0,66	-3,83382559	0,00012617	0,00033738
YDL100C	7771,3	1,53	0,61	3,83134877	0,00012744	0,00034065
YLR118C	1576,17	0,62	-0,69	-3,82911436	0,00012861	0,00034361
YPL176C	1894,09	0,67	-0,57	-3,82893768	0,0001287	0,00034371
YDR023W	22722,33	1,55	0,64	3,82629647	0,00013009	0,00034727
YOL145C	4327,66	1,54	0,62	3,82317553	0,00013174	0,00035154
YLR122C	202,64	2,22	1,15	3,82024636	0,00013332	0,00035559
YOR367W	743,37	0,6	-0,75	-3,81887989	0,00013406	0,00035741
YAL040C	1909,14	0,6	-0,73	-3,81779982	0,00013465	0,00035883
YBR022W	574	0,59	-0,77	-3,81703541	0,00013506	0,00035979
YBR025C	16480,04	1,52	0,61	3,80863283	0,00013974	0,00037207
YBR014C	1761,16	1,52	0,6	3,80754684	0,00014035	0,00037355
YML095C	252,21	0,53	-0,93	-3,80662626	0,00014088	0,00037478
YOR365C	312,44	0,57	-0,81	-3,80459959	0,00014203	0,0003777
YBR071W	1225,11	0,66	-0,59	-3,8043609	0,00014217	0,00037791
YMR306W	599,7	1,6	0,68	3,8037609	0,00014252	0,00037866
YIL156W	1591,48	1,49	0,58	3,80147048	0,00014384	0,00038202
YER120W	5045,22	0,7	-0,51	-3,80113895	0,00014403	0,00038236
YJL061W	1627,02	0,63	-0,66	-3,8001894	0,00014459	0,00038367
YGR126W	224,4	0,49	-1,03	-3,7980676	0,00014583	0,0003868
YPL064C	1282,87	1,5	0,59	3,79657793	0,00014671	0,00038897

YJR036C	1363,41	1,57	0,65	3,79555049	0,00014732	0,00039041
YOR134W	496,85	0,6	-0,73	-3,79495321	0,00014767	0,00039119
YDR103W	1574,41	0,68	-0,56	-3,7945014	0,00014794	0,00039173
YML030W	1324,88	0,63	-0,66	-3,7853623	0,00015348	0,00040624
YPL218W	6206,01	1,41	0,49	3,78438462	0,00015409	0,00040767
YBR091C	322,32	1,81	0,86	3,78216863	0,00015547	0,00041113
YFL018C	8949,69	0,68	-0,57	-3,78206618	0,00015553	0,00041113
YOR227W	4138,67	0,69	-0,53	-3,78028779	0,00015665	0,00041391
YDL209C	300,7	1,74	0,8	3,77933266	0,00015725	0,00041532
YOL096C	904,46	0,58	-0,78	-3,77791823	0,00015814	0,00041751
YOR346W	1638,3	1,56	0,64	3,77288477	0,00016137	0,00042584
YMR232W	185,04	0,48	-1,06	-3,77232272	0,00016173	0,00042662
YBR205W	2900,33	0,7	-0,52	-3,7704792	0,00016293	0,0004296
YGL066W	2288,82	1,44	0,52	3,77022626	0,0001631	0,00042986
YNL012W	547,75	1,61	0,69	3,76865406	0,00016413	0,00043239
YGL171W	1245,59	1,89	0,92	3,76807504	0,00016451	0,00043321
YPL126W	3151,17	1,79	0,84	3,76763456	0,0001648	0,00043379
YDR336W	607,97	1,58	0,66	3,76395386	0,00016725	0,00044004
YBL028C	701,66	1,79	0,84	3,76335775	0,00016765	0,00044091
YLR316C	786,33	1,57	0,65	3,76317125	0,00016777	0,00044105
YGR053C	671,61	1,86	0,9	3,76089518	0,00016931	0,00044489
YMR224C	672,65	0,64	-0,65	-3,76037783	0,00016966	0,00044563
YBR261C	1264	0,66	-0,59	-3,75938358	0,00017033	0,00044721
YKR105C	249,61	1,83	0,87	3,75866171	0,00017082	0,00044832
YDR100W	1322,3	0,58	-0,79	-3,75764315	0,00017152	0,00044995
YPL223C	251,76	2,62	1,39	3,75505781	0,0001733	0,00045443
YAL010C	844,89	0,64	-0,65	-3,75346452	0,00017441	0,00045686
YBR028C	808,71	0,61	-0,72	-3,7535209	0,00017437	0,00045686
YER071C	660,68	1,7	0,77	3,75340609	0,00017445	0,00045686
YKR049C	1127,43	0,49	-1,02	-3,7518577	0,00017553	0,0004595
YGL094C	1538,36	0,55	-0,86	-3,75011479	0,00017675	0,00046251
YBR247C	1329,36	1,61	0,69	3,74883107	0,00017766	0,00046469
YGL172W	1996,67	0,66	-0,6	-3,74822581	0,00017809	0,00046561
YGR033C	1867,25	0,69	-0,54	-3,74788631	0,00017833	0,00046605
YPL270W	1847,81	0,69	-0,54	-3,74560314	0,00017996	0,00047011
YJL020C	8163,34	0,71	-0,5	-3,74223085	0,00018239	0,00047626
YPL236C	764,89	0,62	-0,68	-3,74206098	0,00018252	0,00047639
YML115C	2198,36	0,67	-0,58	-3,74075725	0,00018347	0,00047866
YPR106W	805,64	0,63	-0,66	-3,7383442	0,00018524	0,00048308
YKR102W	226,86	0,52	-0,93	-3,73721667	0,00018607	0,00048504
YLR038C	1392,03	0,59	-0,75	-3,73485219	0,00018783	0,00048942
YNR070W	767,94	0,62	-0,69	-3,73327074	0,00018901	0,0004923
YOR208W	2094,48	1,7	0,77	3,73260664	0,00018951	0,00049339
YGL053W	1095,28	0,64	-0,65	-3,73184457	0,00019008	0,00049468
YMR200W	3038,51	0,7	-0,52	-3,73147951	0,00019036	0,00049519
YMR043W	3056,47	0,7	-0,52	-3,72915186	0,00019213	0,00049957
YEL073C	145,73	0,44	-1,18	-3,72844678	0,00019266	0,00050076
YNL307C	2771,97	0,68	-0,55	-3,72728289	0,00019356	0,00050287
YPR096C	44,97	0,24	-2,06	-3,72679365	0,00019393	0,00050364
YGL161C	3202,87	0,64	-0,64	-3,72537281	0,00019503	0,00050627

YDR394W	8470,78	1,44	0,52	3,72449303	0,00019571	0,00050783
YDR229W	2875,62	1,45	0,54	3,72252365	0,00019724	0,00051159
YMR276W	5826,02	0,71	-0,49	-3,72184906	0,00019777	0,00051275
YDL045C	1592,3	0,64	-0,64	-3,71996366	0,00019925	0,00051637
YLR040C	391,34	0,58	-0,79	-3,7187483	0,00020021	0,00051865
YPL172C	858,47	1,52	0,6	3,71807885	0,00020074	0,00051981
YIL116W	5962,3	1,67	0,74	3,7172391	0,00020141	0,00052132
YMR252C	864,59	1,64	0,71	3,71520973	0,00020304	0,0005253
YLL046C	50,36	3,53	1,82	3,71260424	0,00020514	0,00053052
YNCG0034W	11,48	16,79	4,07	3,71190686	0,0002057	0,00053177
YHR195W	1624,03	0,62	-0,69	-3,70990793	0,00020733	0,00053576
YDL054C	994,76	0,63	-0,66	-3,70835667	0,00020861	0,00053883
YJL048C	2854,39	1,72	0,78	3,70716219	0,00020959	0,00054115
YDR494W	2092,04	0,64	-0,65	-3,70346698	0,00021267	0,00054887
YEL035C	31,14	5,01	2,32	3,70143671	0,00021438	0,00055305
YOR216C	2205,29	1,47	0,55	3,70093731	0,0002148	0,00055391
YIR006C	6714,01	0,7	-0,52	-3,6975365	0,0002177	0,00056115
YPL078C	5026,61	1,51	0,59	3,69693179	0,00021822	0,00056225
YDR005C	2624,94	1,42	0,51	3,69621215	0,00021884	0,00056361
YBR274W	714,6	0,64	-0,65	-3,69314834	0,00022149	0,00057021
YNR047W	1149,42	1,49	0,58	3,69300882	0,00022162	0,00057029
YGL079W	628,39	0,59	-0,77	-3,68955551	0,00022465	0,00057785
YDR382W	34681,75	1,63	0,7	3,68941831	0,00022477	0,00057792
YDR448W	721,66	0,63	-0,66	-3,68486716	0,00022882	0,0005878
YJR040W	1269,51	0,64	-0,65	-3,68485917	0,00022883	0,0005878
YML043C	234,77	1,92	0,94	3,6847868	0,00022889	0,0005878
YNL254C	425,94	1,62	0,7	3,68272584	0,00023075	0,00059233
YPL189C-A	618,38	0,53	-0,92	-3,68219581	0,00023123	0,00059332
YHL013C	721,07	1,58	0,66	3,67913372	0,00023403	0,00060024
YKR061W	1167,9	1,49	0,57	3,67707064	0,00023593	0,00060486
YNL019C	116,22	0,42	-1,24	-3,67557977	0,00023731	0,00060816
YNCO0024C	100,43	2,73	1,45	3,67521011	0,00023765	0,00060879
YDR217C	1247,28	1,61	0,69	3,67480828	0,00023803	0,00060949
YLR362W	1587,11	1,46	0,55	3,67297956	0,00023974	0,00061362
YDR107C	515,78	0,59	-0,77	-3,67178534	0,00024086	0,00061624
YOL069W	728,34	0,65	-0,62	-3,6702633	0,0002423	0,00061967
YNR032W	872,3	0,62	-0,69	-3,66998236	0,00024257	0,00062009
YBL071W-A	130,96	0,46	-1,11	-3,668193	0,00024427	0,00062408
YOR245C	1446,99	0,66	-0,59	-3,66813184	0,00024433	0,00062408
YDR265W	1166,26	1,49	0,57	3,66744573	0,00024499	0,0006255
YDR272W	2607,12	0,62	-0,7	-3,66479931	0,00024753	0,00063175
YBR072C-A	23,05	5,82	2,54	3,6628488	0,00024943	0,00063632
YJL174W	3372,92	0,68	-0,57	-3,65901459	0,00025319	0,00064565
YOL116W	1811,71	1,49	0,57	3,65866446	0,00025353	0,00064626
YGL093W	1485,14	0,68	-0,56	-3,65773152	0,00025446	0,00064835
YOR132W	2891,92	0,7	-0,5	-3,6570608	0,00025512	0,00064979
YIL134W	354,17	0,57	-0,8	-3,65630049	0,00025588	0,00065145
YLR172C	2703,07	0,69	-0,54	-3,65159382	0,00026062	0,00066311
YNL272C	1610,79	0,67	-0,58	-3,65153731	0,00026068	0,00066311
YKL048C	551,47	1,68	0,75	3,65121131	0,00026101	0,00066368

YMR233W	475,46	0,61	-0,7	-3,64738779	0,00026492	0,00067336
YCR073C	1138,41	1,56	0,64	3,64677434	0,00026555	0,00067469
YML018C	892,72	1,94	0,95	3,6448861	0,00026751	0,00067938
YML086C	3844,91	0,71	-0,49	-3,64458633	0,00026782	0,0006799
YPL018W	527,64	1,61	0,69	3,64363247	0,00026882	0,00068215
YDR363W	950,72	0,66	-0,61	-3,64332281	0,00026914	0,00068269
YIR028W	152,08	0,46	-1,13	-3,64306237	0,00026941	0,0006831
YLR009W	1744,45	1,47	0,56	3,64223851	0,00027028	0,00068501
YPL012W	2585,05	1,85	0,89	3,64204086	0,00027049	0,00068526
YML110C	5028,41	0,66	-0,6	-3,64118864	0,00027138	0,00068725
YNL259C	534,71	2,02	1,02	3,64071658	0,00027188	0,00068823
YDR206W	2519,06	1,51	0,6	3,64050264	0,00027211	0,00068852
YFR005C	1001,54	0,67	-0,57	-3,63915204	0,00027354	0,0006918
YNL185C	474,71	0,56	-0,84	-3,63906873	0,00027363	0,0006918
YLR348C	1390,88	1,46	0,55	3,6379153	0,00027485	0,00069463
YDR186C	3142,73	1,45	0,54	3,63730548	0,00027551	0,00069599
YIR016W	1565,11	1,73	0,79	3,63710391	0,00027572	0,00069625
YNL206C	1695,44	1,52	0,6	3,63503391	0,00027794	0,00070158
YBR242W	1013,59	1,51	0,6	3,63340973	0,0002797	0,00070548
YLR008C	1096,09	0,65	-0,61	-3,63339561	0,00027972	0,00070548
YCR043C	816,68	0,62	-0,68	-3,6321208	0,0002811	0,0007084
YLR368W	1006,72	0,66	-0,59	-3,63222015	0,00028099	0,0007084
YHR143W	5854,34	1,54	0,62	3,63175499	0,0002815	0,00070883
YOR376W	23,8	6,06	2,6	3,63180016	0,00028145	0,00070883
YPR064W	75,29	2,81	1,49	3,62952799	0,00028394	0,00071468
YLR388W	4927,35	1,52	0,6	3,62865905	0,0002849	0,0007168
YOR035C	2066,81	0,66	-0,6	-3,626587	0,00028719	0,00072228
YDR259C	411,21	1,67	0,74	3,62596651	0,00028788	0,00072373
YER010C	1170,11	0,68	-0,56	-3,62384884	0,00029025	0,00072939
YLR190W	1552,15	0,69	-0,54	-3,62076316	0,00029374	0,00073784
YFL033C	2501,11	1,49	0,57	3,61964009	0,00029501	0,00074075
YJR051W	1842,76	0,69	-0,54	-3,61858679	0,00029622	0,00074347
YGR078C	1268,5	0,69	-0,54	-3,61731402	0,00029768	0,00074684
YNL287W	7767,86	0,66	-0,61	-3,61606646	0,00029911	0,00075014
YCR003W	1006,4	1,65	0,72	3,6141127	0,00030138	0,00075551
YJR015W	2834,96	0,71	-0,49	-3,61386503	0,00030167	0,00075593
YNL201C	1425,98	0,68	-0,57	-3,61106119	0,00030495	0,00076353
YPR088C	5392,11	1,46	0,55	3,61112867	0,00030487	0,00076353
YOR326W	6766,33	1,61	0,69	3,61046075	0,00030565	0,000765
YDR399W	3855,97	0,71	-0,49	-3,60965634	0,0003066	0,00076699
YOR167C	7244,11	1,49	0,57	3,60957792	0,0003067	0,00076699
YMR201C	725,04	1,52	0,6	3,60539663	0,00031168	0,00077913
YGL034C	56,83	2,84	1,5	3,60239566	0,0003153	0,00078786
YDR397C	1208,33	1,47	0,55	3,59504295	0,00032434	0,00081013
YML073C	16531,08	0,73	-0,46	-3,59414937	0,00032545	0,00081259
YGR050C	186,36	2,01	1,01	3,59306852	0,00032681	0,00081564
YDR351W	1721,5	1,49	0,57	3,59131306	0,00032902	0,00082082
YDR036C	2216,91	1,44	0,53	3,59108189	0,00032931	0,00082122
YBL071C	71,03	0,33	-1,6	-3,59064312	0,00032986	0,00082227
YDL013W	1126,25	0,68	-0,55	-3,59010909	0,00033054	0,00082363

YJL094C	1649,54	0,67	-0,58	-3,58474174	0,00033741	0,00084042
YDR264C	4078,92	1,39	0,48	3,58347343	0,00033906	0,00084417
YIL122W	635,2	0,62	-0,69	-3,58290796	0,00033979	0,00084566
YCR047C	733,78	1,52	0,6	3,58222921	0,00034067	0,00084753
YLR333C	5135,77	1,52	0,61	3,58094974	0,00034235	0,00085135
YDL073W	1790,67	1,7	0,76	3,57780171	0,0003465	0,00086132
YFL021W	2035,83	1,45	0,54	3,57268449	0,00035334	0,00087798
YGL154C	404,83	0,58	-0,77	-3,57118004	0,00035538	0,00088269
YDR398W	1704,22	1,67	0,74	3,57020717	0,0003567	0,00088562
YCR019W	895,11	1,51	0,59	3,5694131	0,00035778	0,00088795
YDR435C	1082,94	0,68	-0,56	-3,56570046	0,00036289	0,00090026
YLR370C	3819,49	0,72	-0,48	-3,5647896	0,00036415	0,00090303
YCR004C	5646,29	0,62	-0,69	-3,564248	0,0003649	0,00090453
YBL088C	2352,5	1,48	0,56	3,56107259	0,00036934	0,00091518
YLR262C-A	1741,73	1,47	0,56	3,55926995	0,00037189	0,00092112
YOR097C	687,72	0,6	-0,73	-3,55688555	0,00037528	0,00092914
YGL104C	1056	0,59	-0,77	-3,54858565	0,00038731	0,00095854
YDL218W	117,13	2,13	1,09	3,54558333	0,00039175	0,00096914
YJR141W	455,97	1,58	0,66	3,53747423	0,00040397	0,000999
YHR001W	2098,03	0,69	-0,53	-3,53665455	0,00040523	0,0010017
YLR014C	339,42	1,73	0,79	3,53632501	0,00040574	0,00100256
YCL043C	23442,49	0,72	-0,47	-3,53377401	0,00040967	0,00101188
YGL191W	2581,6	0,59	-0,76	-3,53261199	0,00041148	0,00101593
YPR199C	1082,83	0,64	-0,65	-3,53112459	0,0004138	0,00102126
YPL061W	11905,1	1,79	0,84	3,52919849	0,00041682	0,00102831
YNR033W	2030,96	0,7	-0,51	-3,52785461	0,00041894	0,00103314
YNL052W	2771,49	0,62	-0,69	-3,52511099	0,00042331	0,00104348
YMR194W	4757,21	1,56	0,64	3,5248374	0,00042374	0,00104415
YDR532C	1270,16	1,45	0,53	3,52462554	0,00042408	0,00104457
YDR496C	2241,43	1,64	0,71	3,51972188	0,000432	0,00106365
YJL219W	76,81	2,4	1,27	3,51813875	0,00043459	0,00106959
YOR184W	9746,08	0,58	-0,79	-3,51623322	0,00043772	0,00107687
YLR386W	1578,77	0,7	-0,52	-3,51533118	0,00043921	0,00108011
YBR235W	1312,01	0,69	-0,55	-3,51169787	0,00044525	0,00109455
YDR084C	859,8	0,66	-0,59	-3,5107532	0,00044684	0,00109801
YLR137W	454,32	0,62	-0,68	-3,50502706	0,00045656	0,00112145
YPR011C	1175,61	1,54	0,62	3,50378449	0,0004587	0,00112625
YDR032C	7441,9	1,72	0,78	3,50343397	0,0004593	0,00112729
YER018C	443,09	0,54	-0,89	-3,50240101	0,00046109	0,00113122
YOR165W	2585,31	0,68	-0,55	-3,50174959	0,00046221	0,00113355
YGR031W	317,53	0,55	-0,86	-3,50064212	0,00046414	0,00113782
YNR006W	3213,01	1,43	0,52	3,4998131	0,00046558	0,00114091
YKL027W	1401,07	1,45	0,53	3,49933324	0,00046642	0,00114207
YNL306W	2026,14	0,71	-0,5	-3,49935243	0,00046639	0,00114207
YOR038C	818,64	0,65	-0,61	-3,49909961	0,00046683	0,00114262
YFL041W-A	85,02	2,42	1,27	3,49437828	0,00047517	0,00116256
YDR511W	989,31	1,69	0,76	3,49408232	0,00047569	0,00116339
YGL101W	1040,24	0,67	-0,58	-3,49326848	0,00047715	0,00116648
YOR194C	3300,81	1,38	0,47	3,49005447	0,00048292	0,00118014
YNL046W	811,63	0,61	-0,72	-3,4885201	0,0004857	0,00118647

YPR147C	1345,01	0,68	-0,55	-3,4881148	0,00048644	0,0011878
YBR258C	315,04	0,6	-0,74	-3,4876698	0,00048725	0,00118931
YHR214C-E	19,57	6,13	2,62	3,48752511	0,00048751	0,00118949
YDL063C	1006,43	1,63	0,71	3,48348697	0,00049493	0,0012071
YLR087C	1274,74	0,68	-0,55	-3,48318753	0,00049548	0,00120798
YMR030W	801,4	1,5	0,58	3,47979251	0,0005018	0,00122291
YGL159W	719,45	0,65	-0,63	-3,47952231	0,00050231	0,00122366
YML029W	1998,67	1,41	0,5	3,47919572	0,00050292	0,00122468
YDL044C	536,37	0,64	-0,63	-3,47905772	0,00050318	0,00122483
YDL220C	548,59	0,61	-0,72	-3,47878134	0,0005037	0,00122561
YER082C	2551,17	1,62	0,7	3,47825699	0,00050469	0,00122741
YMR167W	931,78	0,67	-0,58	-3,47817906	0,00050483	0,00122741
YJL041W	4775,96	1,44	0,53	3,47761198	0,0005059	0,00122952
YOR284W	358,67	0,6	-0,73	-3,47587234	0,00050919	0,00123704
YOR085W	1312,6	0,62	-0,69	-3,47572008	0,00050948	0,00123726
YDL055C	32439,75	0,74	-0,44	-3,47198235	0,00051663	0,00125413
YLR363C	643,06	1,56	0,64	3,47138339	0,00051778	0,00125644
YKR004C	571,26	1,55	0,63	3,47114718	0,00051824	0,00125656
YPL245W	624,73	0,64	-0,65	-3,47118679	0,00051816	0,00125656
YPR182W	754,69	1,74	0,8	3,46935695	0,00052171	0,00126447
YHR186C	1232,35	0,66	-0,59	-3,46353583	0,00053313	0,00129164
YMR147W	331,23	0,6	-0,74	-3,46103374	0,00053811	0,0013032
YHR079C-A	11,88	17,54	4,13	3,45949499	0,00054119	0,00131016
YML077W	871,92	1,57	0,65	3,45830328	0,00054359	0,00131546
YBR180W	91,38	0,42	-1,26	-3,45783555	0,00054453	0,00131707
YDR249C	333,84	1,66	0,73	3,45776363	0,00054468	0,00131707
YDR056C	1996,66	0,71	-0,49	-3,45719427	0,00054583	0,00131934
YOR073W	533,2	0,64	-0,64	-3,45173823	0,00055699	0,00134578
YPL069C	472,07	0,62	-0,68	-3,45145363	0,00055758	0,00134668
YER074W	11134,55	1,52	0,61	3,44809439	0,00056456	0,00136269
YOL041C	1878,69	1,65	0,72	3,44805219	0,00056464	0,00136269
YNCM0005C	64,43	2,57	1,36	3,44699039	0,00056687	0,00136753
YOR250C	1164,87	1,52	0,6	3,44540631	0,0005702	0,00137504
YNCC0003C	82,5	2,63	1,4	3,44446855	0,00057218	0,00137928
YFR001W	1671,22	1,57	0,65	3,44195454	0,00057753	0,00139162
YEL043W	3278,95	1,44	0,53	3,44047989	0,00058068	0,00139868
YER076C	454,72	0,62	-0,68	-3,44033468	0,000581	0,00139889
YDL067C	2841,06	1,56	0,64	3,43954058	0,0005827	0,00140246
YLR188W	1586,7	0,7	-0,51	-3,43792892	0,00058618	0,00141029
YLR247C	2635,24	1,5	0,59	3,43686907	0,00058848	0,00141527
YNL336W	574,45	0,61	-0,71	-3,4343419	0,00059399	0,00142798
YML079W	1570,57	0,69	-0,54	-3,42993092	0,00060373	0,00145083
YMR066W	1052,61	1,44	0,53	3,42897867	0,00060586	0,00145537
YAL023C	7180,04	0,69	-0,53	-3,41980774	0,00062665	0,00150474
YCL038C	664,21	0,64	-0,65	-3,4187223	0,00062916	0,00151018
YPL059W	1784,94	1,46	0,54	3,41754739	0,00063188	0,00151612
YNL056W	1325,04	1,44	0,53	3,41692569	0,00063333	0,001519
YMR162C	1514,4	0,7	-0,52	-3,41608145	0,00063529	0,00152313
Q0085	9,41	28,31	4,82	3,41373564	0,00064079	0,00153571
YJL155C	2120,22	0,67	-0,59	-3,41327457	0,00064187	0,00153772

YKL132C	484,44	0,64	-0,65	-3,41313295	0,00064221	0,00153793
YDL003W	1216,25	0,68	-0,56	-3,41295894	0,00064262	0,00153814
YKL018C-A	518,64	0,6	-0,73	-3,41288444	0,00064279	0,00153814
YLR270W	3771,31	0,71	-0,49	-3,41245546	0,0006438	0,00153997
YKL159C	726,41	0,65	-0,62	-3,41177997	0,0006454	0,0015432
YGR130C	10774,71	1,42	0,5	3,41158436	0,00064587	0,00154371
YLR069C	2240,53	0,7	-0,51	-3,41023876	0,00064906	0,00155075
YGR002C	1415,82	1,41	0,5	3,40722717	0,00065626	0,00156737
YFR009W	7418,49	1,45	0,53	3,40324473	0,00066591	0,00158978
YOR207C	2650,64	1,65	0,72	3,40177533	0,0006695	0,00159774
YLR457C	553,56	1,55	0,63	3,40059663	0,00067239	0,00160403
YBR267W	296,27	1,65	0,73	3,39960129	0,00067484	0,00160926
YDR429C	5928,85	1,42	0,51	3,39940239	0,00067533	0,00160981
YER009W	5406,77	0,73	-0,45	-3,39867567	0,00067713	0,00161347
YPL272C	437,67	1,67	0,74	3,39835149	0,00067793	0,00161477
YBR189W	23919,78	0,72	-0,48	-3,3979975	0,00067881	0,00161624
YPR048W	769,34	1,58	0,66	3,39671109	0,00068201	0,00162323
YOR025W	707,34	0,67	-0,57	-3,39461102	0,00068726	0,00163511
YNR018W	1856,96	0,7	-0,51	-3,39386406	0,00068914	0,00163895
YIL067C	1034,3	1,62	0,7	3,3924505	0,0006927	0,0016468
YKR096W	2767,29	1,38	0,46	3,39152046	0,00069506	0,00165176
YBR128C	396,45	0,54	-0,89	-3,38992598	0,00069911	0,00166077
YDR020C	371,83	0,62	-0,69	-3,38759768	0,00070508	0,00167365
YGL016W	829,82	0,59	-0,77	-3,38760921	0,00070505	0,00167365
YML108W	341,56	1,63	0,71	3,38747467	0,00070539	0,00167376
YKL014C	1834,07	1,75	0,81	3,38725015	0,00070597	0,00167449
YJR110W	1009,76	1,47	0,56	3,38712083	0,0007063	0,00167464
YPL095C	1421,73	0,51	-0,96	-3,38584762	0,00070959	0,00168179
YGL200C	5092,02	0,73	-0,45	-3,3850371	0,00071169	0,00168555
YLR313C	393,35	1,65	0,72	3,38502423	0,00071172	0,00168555
YGR098C	926,87	0,66	-0,59	-3,3839659	0,00071447	0,00169077
YJL053W	3564,21	1,38	0,47	3,38399274	0,0007144	0,00169077
YMR194C-B	252,09	2,27	1,18	3,38188718	0,0007199	0,00170297
YGR058W	624,68	0,66	-0,61	-3,37830252	0,00072935	0,00172467
YKL026C	671,17	0,55	-0,87	-3,37763316	0,00073113	0,00172774
YNR015W	1223,2	1,5	0,58	3,37760364	0,0007312	0,00172774
YLR068W	573,96	1,58	0,66	3,37695504	0,00073293	0,00173116
YER014W	1090,89	0,67	-0,57	-3,37659675	0,00073389	0,00173276
YLR154C	440,88	0,62	-0,69	-3,3759618	0,00073558	0,00173611
YER066W	266,78	0,5	-1,01	-3,37520877	0,0007376	0,0017402
YBR034C	1440,17	1,6	0,68	3,37306575	0,00074336	0,00175314
YKL077W	3013,69	0,69	-0,55	-3,37274405	0,00074423	0,00175452
YDR367W	790,1	1,5	0,59	3,37229004	0,00074546	0,00175675
YGR086C	16878,1	0,74	-0,43	-3,37194605	0,00074639	0,00175828
YGR040W	849,97	1,46	0,54	3,37064761	0,00074992	0,00176592
YHR069C	1019,37	1,48	0,56	3,37015254	0,00075127	0,00176842
YDL189W	2304,76	1,42	0,51	3,36628518	0,00076188	0,00179272
YBL039C	2489,84	1,69	0,76	3,36571838	0,00076345	0,00179573
YGL021W	1296,49	0,69	-0,54	-3,3641553	0,00076778	0,00180525
YLR321C	1425,78	0,69	-0,54	-3,36166407	0,00077474	0,00182092

YBR185C	698,84	0,61	-0,71	-3,35754101	0,00078639	0,0018476
YNL277W-A	21,63	4,85	2,28	3,35673607	0,00078868	0,00185159
YOR151C	7001,49	1,5	0,59	3,35678906	0,00078853	0,00185159
YCR096C	22,23	4,76	2,25	3,35429233	0,00079568	0,00186731
YPL032C	4178,77	1,53	0,61	3,3524432	0,00080102	0,00187912
YGL003C	1491,94	1,42	0,5	3,35068508	0,00080612	0,00189038
YDL080C	1287,03	0,7	-0,51	-3,3492205	0,00081039	0,00189968
YKR077W	657,62	0,65	-0,63	-3,34837405	0,00081287	0,00190478
YIL133C	12801,54	0,72	-0,48	-3,34771473	0,00081481	0,00190859
YNR026C	1111,76	1,47	0,56	3,34383221	0,0008263	0,00193477
YHR036W	454,34	0,62	-0,7	-3,34252064	0,00083021	0,00194321
YNCM0030W	315,68	0,6	-0,75	-3,34121327	0,00083413	0,00195165
YLL008W	2575,39	1,61	0,69	3,34086625	0,00083517	0,00195335
YFL011W	53,98	2,69	1,43	3,34028291	0,00083693	0,00195672
YDL185W	16152,86	1,4	0,49	3,33989544	0,0008381	0,00195872
YBL059C-A	694,73	0,63	-0,67	-3,33575658	0,00085068	0,00198736
YOR020C	8007,77	1,48	0,57	3,33433011	0,00085505	0,00199683
YGR102C	837,93	0,65	-0,62	-3,33297469	0,00085923	0,00200583
YNL139C	1807,69	0,67	-0,58	-3,33162382	0,00086341	0,00201414
YOR376W-A	238,02	0,54	-0,88	-3,33161535	0,00086344	0,00201414
YGR153W	218,12	1,72	0,79	3,33054053	0,00086678	0,00202117
YDL119C	579,3	0,66	-0,6	-3,32744027	0,00087648	0,00204303
YPR108W	4319,79	0,72	-0,47	-3,32418117	0,00088679	0,00206628
YJR088C	2986,73	0,73	-0,45	-3,32347938	0,00088902	0,00207071
YDR170W-A	593,88	1,51	0,59	3,3221875	0,00089315	0,00207955
YNL141W	1922,4	1,72	0,78	3,32154233	0,00089521	0,00208358
YBR141C	330,1	1,62	0,7	3,3202724	0,0008993	0,0020923
YML097C	1020,67	0,69	-0,54	-3,31638843	0,00091189	0,0021208
YPL200W	30,62	3,63	1,86	3,31545214	0,00091495	0,00212712
YPL192C	173,59	1,97	0,98	3,31380609	0,00092035	0,00213888
YDR409W	1811,65	1,39	0,47	3,31257256	0,00092442	0,00214753
YMR284W	1573,2	0,72	-0,48	-3,30997459	0,00093304	0,00216639
YNL097C	1418,06	1,45	0,54	3,30991761	0,00093323	0,00216639
YPR082C	391,31	0,59	-0,76	-3,30919363	0,00093565	0,00217119
YLR221C	1572,54	1,55	0,63	3,30904346	0,00093615	0,00217154
YDL155W	826,42	0,68	-0,56	-3,30696106	0,00094314	0,00218693
YGL054C	4574,97	1,4	0,49	3,30383736	0,00095371	0,00221062
YDL124W	21314,28	0,73	-0,46	-3,30360528	0,0009545	0,00221162
YMR008C-A	172,58	0,49	-1,02	-3,30155567	0,0009615	0,00222701
YFL053W	372,54	0,51	-0,97	-3,29924112	0,00096947	0,00224462
YER106W	33,9	3,89	1,96	3,29841145	0,00097234	0,00225043
YDL224C	1986,88	0,72	-0,48	-3,29723403	0,00097642	0,00225904
YAL036C	1964,44	1,5	0,58	3,29376557	0,00098855	0,00228625
YOR296W	797,5	0,68	-0,55	-3,29160796	0,00099616	0,002303
YKL034W	1910,41	0,72	-0,47	-3,28917677	0,00100481	0,00232213
YPL249C-A	23039,69	1,38	0,46	3,28481519	0,00102049	0,00235749
YBL100W-C	27,71	0,22	-2,21	-3,28457863	0,00102135	0,00235772
YJL167W	8870,1	0,74	-0,43	-3,28466712	0,00102103	0,00235772
YIR042C	189,67	0,52	-0,95	-3,28434861	0,00102218	0,00235877
YOR142W	4526,29	0,71	-0,5	-3,2832143	0,00102631	0,0023674

YBR123C	993,22	0,7	-0,52	-3,28302911	0,00102698	0,00236808
YGL131C	1025,52	0,68	-0,56	-3,28131169	0,00103325	0,00238166
YDR034C	1656,59	0,72	-0,48	-3,28106865	0,00103415	0,00238282
YLR373C	1466,69	0,68	-0,55	-3,28096541	0,00103452	0,00238282
YBR225W	1311,53	0,71	-0,49	-3,27755406	0,00104711	0,00241091
YLR285C-A	56,07	0,36	-1,48	-3,27456149	0,00105826	0,00243569
YDR219C	1306,39	0,7	-0,5	-3,27340906	0,00106259	0,00244473
YGR156W	1499,66	0,72	-0,48	-3,27250285	0,001066	0,00245168
YIL170W	200,15	1,76	0,81	3,27196295	0,00106804	0,00245545
YLR143W	825,91	0,62	-0,68	-3,27180924	0,00106862	0,00245588
YPL175W	907,04	1,47	0,56	3,27124507	0,00107075	0,00245988
YPR069C	5598,77	1,47	0,55	3,27014054	0,00107494	0,00246859
YPL108W	213,22	1,92	0,94	3,2699574	0,00107564	0,00246927
YKL176C	1713,34	1,42	0,51	3,26762872	0,00108453	0,00248876
YJR100C	1136,39	0,7	-0,51	-3,26544275	0,00109293	0,00250712
YBL082C	977,83	0,7	-0,52	-3,26510974	0,00109422	0,00250915
YGL090W	821,28	1,49	0,57	3,26482493	0,00109532	0,00251051
YNL094W	1807,84	0,71	-0,49	-3,26474638	0,00109562	0,00251051
YDR441C	644,54	1,48	0,56	3,26171222	0,00110742	0,00253637
YEL052W	2178,85	0,7	-0,52	-3,26163324	0,00110772	0,00253637
YKR078W	902,63	0,69	-0,53	-3,26108213	0,00110988	0,00254037
YOR262W	843,2	1,49	0,58	3,25989982	0,00111452	0,00255004
YLR238W	1345,63	1,44	0,52	3,25761347	0,00112353	0,00256973
YHL048C-A	5,18	87,96	6,46	3,25657928	0,00112763	0,00257816
Q0105	5,22	87,43	6,45	3,25393106	0,0011382	0,00260136
YDR471W	8931,56	0,71	-0,5	-3,25349579	0,00113994	0,00260439
YOR298C-A	20936,88	1,4	0,48	3,25325503	0,00114091	0,00260564
YDR325W	741,74	0,65	-0,62	-3,25081359	0,00115075	0,00262715
YBR085W	379,08	1,68	0,75	3,24934101	0,00115673	0,00263982
YHR106W	1121,79	0,65	-0,62	-3,2485383	0,00116	0,00264631
YGL181W	3927,65	1,42	0,5	3,24738547	0,00116471	0,00265608
YHR073W	3287,29	0,73	-0,46	-3,2465681	0,00116805	0,00266274
YLR208W	4606,41	0,74	-0,43	-3,24522355	0,00117358	0,00267436
YPL163C	5357,71	0,74	-0,42	-3,24510419	0,00117408	0,0026745
YIL073C	550,7	0,66	-0,61	-3,24478053	0,00117541	0,00267656
YGR100W	2428,15	0,72	-0,47	-3,24436565	0,00117713	0,00267948
YGR234W	23631,59	1,59	0,67	3,24314792	0,00118217	0,00268997
YOL117W	1341,28	1,4	0,48	3,24250126	0,00118485	0,0026951
YPR074C	13736,39	0,64	-0,65	-3,24217096	0,00118623	0,00269724
YPL211W	992,69	1,5	0,58	3,23786234	0,00120429	0,0027373
YLR095C	3065,86	1,37	0,45	3,23503975	0,00121626	0,00276349
YHR098C	4653,67	0,69	-0,54	-3,23446134	0,00121872	0,00276727
YOR131C	1680,51	1,47	0,56	3,23444146	0,00121881	0,00276727
YKL120W	1552,74	0,6	-0,74	-3,23223106	0,00122828	0,00278673
YOL042W	1011,27	1,43	0,51	3,23224456	0,00122822	0,00278673
YDR478W	278,1	1,66	0,73	3,23163825	0,00123083	0,00279149
YEL020W-A	455,1	0,55	-0,86	-3,23139529	0,00123187	0,00279285
YGL250W	551,12	0,63	-0,66	-3,2311701	0,00123285	0,00279403
YPL058C	2032,97	1,39	0,48	3,22979217	0,0012388	0,00280651
YKL210W	12615,65	1,42	0,5	3,22909663	0,00124182	0,00281232

YLR298C	1173,82	1,43	0,52	3,22867806	0,00124364	0,00281541
YIL151C	1383,35	0,7	-0,51	-3,22634965	0,0012538	0,00283738
YNL244C	3853,35	0,72	-0,47	-3,22526855	0,00125855	0,00284708
YBR230C	2403,8	0,52	-0,95	-3,22451668	0,00126186	0,00285353
YLR253W	2072,18	1,39	0,48	3,22376341	0,00126518	0,00286
YDR051C	2470,43	1,39	0,47	3,21998368	0,00128198	0,00289693
YDL219W	964,14	1,46	0,55	3,21600965	0,00129986	0,00293574
YLR265C	1004,08	0,68	-0,56	-3,21595796	0,0013001	0,00293574
YGR148C	19560,5	1,35	0,43	3,21582987	0,00130068	0,00293598
YDL167C	1492,79	1,6	0,68	3,21493447	0,00130474	0,00294408
YNL074C	2683,65	0,73	-0,46	-3,21403308	0,00130885	0,00295227
YPR034W	1796,89	0,72	-0,48	-3,21339173	0,00131177	0,0029578
YAR050W	289,26	0,61	-0,72	-3,21213906	0,00131751	0,00296857
YHR168W	561,23	1,49	0,57	3,21215743	0,00131742	0,00296857
YHR003C	1614,53	1,38	0,47	3,21017901	0,00132652	0,0029878
YPL269W	869,27	1,45	0,53	3,20775075	0,00133777	0,00301205
YMR121C	1888,7	1,39	0,48	3,20705024	0,00134104	0,0030183
YOR321W	1137,63	0,69	-0,53	-3,20596851	0,00134609	0,00302857
YDR538W	587,55	0,67	-0,57	-3,20404233	0,00135513	0,00304781
YML020W	1138,64	0,71	-0,5	-3,20365821	0,00135693	0,00305077
YLR245C	252,4	0,6	-0,74	-3,20332701	0,0013585	0,00305318
YOR230W	18307,76	0,67	-0,57	-3,19977268	0,00137536	0,00308996
YIL060W	191,32	0,52	-0,96	-3,19875993	0,0013802	0,00309971
YDR459C	563,81	0,65	-0,61	-3,19681846	0,00138952	0,0031184
YPL101W	1270,68	0,7	-0,51	-3,19682063	0,00138951	0,0031184
YKL179C	2409,93	0,7	-0,51	-3,19362633	0,00140498	0,00315194
YPR080W	40371,48	1,48	0,56	3,19255943	0,00141018	0,00316247
YDL095W	6085,79	0,71	-0,5	-3,19098171	0,0014179	0,00317864
YBR074W	1426,31	0,68	-0,56	-3,18970087	0,0014242	0,00319161
YGL205W	206,33	0,55	-0,87	-3,1885469	0,0014299	0,00320322
YGR099W	689,17	0,68	-0,56	-3,18755148	0,00143483	0,00321311
YER022W	2109,33	1,38	0,47	3,18162945	0,00146449	0,00327719
YKR039W	676,3	1,57	0,65	3,18162716	0,0014645	0,00327719
YHR177W	332,11	0,6	-0,74	-3,18027525	0,00147135	0,00329134
YPR045C	1941,9	0,71	-0,5	-3,18013762	0,00147205	0,00329171
YMR157C	665,63	0,67	-0,57	-3,17952559	0,00147516	0,00329749
YER005W	1211,64	0,71	-0,49	-3,17901695	0,00147775	0,00330209
YPR083W	365,97	0,6	-0,73	-3,17831438	0,00148134	0,00330891
YOL033W	692,23	0,65	-0,62	-3,17663561	0,00148994	0,00332693
YLR455W	967,42	1,44	0,52	3,17629772	0,00149168	0,00332961
YGL119W	809,35	0,69	-0,54	-3,17344476	0,00150642	0,0033613
YIL142W	6103,98	1,41	0,5	3,17152232	0,00151642	0,00338241
YBR130C	2740,01	1,41	0,49	3,17139898	0,00151707	0,00338263
YJL204C	1096,98	0,69	-0,54	-3,17053053	0,00152161	0,00339154
YBR270C	443,34	0,57	-0,8	-3,17014633	0,00152362	0,00339479
YOR060C	513,19	0,66	-0,6	-3,17004395	0,00152416	0,00339479
YMR139W	2609,67	0,72	-0,47	-3,1682095	0,00153381	0,00341506
YJR131W	784,06	0,7	-0,52	-3,16671542	0,00154171	0,00343143
YDR197W	1026,32	0,62	-0,7	-3,16645247	0,00154311	0,0034333
YNL066W	2635,41	0,68	-0,57	-3,1661397	0,00154477	0,00343576

YHR063C	2720,91	0,73	-0,45	-3,16571496	0,00154702	0,00343955
YLR437C	996,41	1,53	0,62	3,16517989	0,00154987	0,00344465
YBR299W	166,34	1,88	0,91	3,16429388	0,0015546	0,00345392
YDL213C	1910,56	1,42	0,51	3,16163527	0,00156886	0,00348436
YPL169C	4027,1	1,46	0,54	3,16058812	0,00157451	0,00349566
YPR159W	4616,99	1,41	0,5	3,15940685	0,00158091	0,00350861
YMR282C	1126,88	1,4	0,48	3,15804894	0,00158829	0,00352247
YOR110W	1043,23	0,7	-0,51	-3,15809448	0,00158804	0,00352247
YMR262W	963,55	0,63	-0,66	-3,15685676	0,0015948	0,00353564
YPR115W	4303,35	0,74	-0,43	-3,15630957	0,00159779	0,00354102
YFL044C	931,95	0,69	-0,53	-3,15618779	0,00159846	0,00354124
YPR098C	1202,21	0,61	-0,71	-3,15439957	0,00160829	0,00356174
YJR098C	410,55	0,65	-0,62	-3,15417169	0,00160954	0,00356325
YNL095C	730,99	0,69	-0,54	-3,15385683	0,00161128	0,00356583
YCL069W	9,83	11,15	3,48	3,15361126	0,00161264	0,003567
YNL155W	2354,37	1,42	0,5	3,15355298	0,00161296	0,003567
YPL128C	510,86	0,59	-0,75	-3,14934608	0,00163636	0,00361747
YLR440C	1842,13	1,38	0,46	3,14595852	0,00165543	0,00365833
YER154W	2157,51	0,72	-0,47	-3,14469588	0,0016626	0,00367285
YDR513W	4724,86	1,66	0,73	3,14339402	0,00167001	0,00368721
YMR294W	567,36	1,48	0,57	3,14334535	0,00167029	0,00368721
YMR023C	601,9	1,5	0,58	3,14142188	0,0016813	0,0037102
YLR083C	1884,62	0,69	-0,54	-3,14023848	0,0016881	0,0037239
YBR136W	1075,33	1,47	0,56	3,13955078	0,00169207	0,00373133
YJR019C	708,74	0,63	-0,66	-3,1390044	0,00169523	0,00373696
YBR201C-A	304,86	0,6	-0,75	-3,13873053	0,00169681	0,00373913
YJR006W	1783,84	0,71	-0,49	-3,13854168	0,00169791	0,00374021
YBR279W	4481,69	1,34	0,42	3,13742816	0,00170437	0,00375312
YJR121W	24949,38	0,73	-0,46	-3,1363924	0,0017104	0,00376507
YBL060W	815,11	1,43	0,52	3,13536828	0,00171638	0,0037769
YNL280C	2022,38	0,73	-0,45	-3,13317398	0,00172927	0,0038039
YMR156C	377,92	0,65	-0,63	-3,1319148	0,0017367	0,0038189
YKL033W-A	2911,98	0,69	-0,54	-3,13118377	0,00174103	0,00382707
YJL128C	2525,16	0,74	-0,44	-3,12788771	0,00176067	0,00386887
YGL136C	926,9	0,69	-0,54	-3,12758021	0,00176252	0,00387155
YER094C	3677,82	0,74	-0,44	-3,1273626	0,00176382	0,00387305
YDL087C	482,91	1,51	0,6	3,12708148	0,00176551	0,00387539
YKR041W	95,59	2,14	1,1	3,12421878	0,00178278	0,00391191
YOL049W	4792,73	0,75	-0,42	-3,12239456	0,00179386	0,00393484
YPL264C	133,46	0,51	-0,96	-3,12217411	0,00179521	0,0039364
YGR245C	3282,89	1,64	0,71	3,12191792	0,00179677	0,00393844
YBL045C	12361	1,4	0,49	3,12100697	0,00180234	0,00394925
YOR114W	294,63	0,61	-0,71	-3,11984521	0,00180946	0,00396346
YBL035C	764,24	0,68	-0,56	-3,11917389	0,00181359	0,0039711
YLR187W	653,66	0,61	-0,72	-3,11636158	0,00183098	0,00400776
YMR244W	42,03	2,85	1,51	3,11596284	0,00183345	0,00401177
YDR316W	1491,34	1,41	0,49	3,11499724	0,00183947	0,00402351
YGR263C	595,46	0,66	-0,6	-3,11409369	0,00184511	0,00403443
YNL021W	1386,94	0,71	-0,5	-3,11379577	0,00184697	0,00403709
YBR302C	125,76	0,41	-1,3	-3,11368446	0,00184767	0,00403719

YGL120C	4674,37	1,57	0,65	3,11314361	0,00185106	0,00404317
YKR055W	524,38	0,65	-0,63	-3,11297552	0,00185211	0,00404405
YBL066C	1098,74	0,71	-0,5	-3,10843076	0,00188084	0,00410533
YIR029W	337	0,58	-0,78	-3,10770455	0,00188546	0,00411398
YLR103C	550,51	0,65	-0,62	-3,10688537	0,0018907	0,00412395
YOL088C	1337,23	0,71	-0,5	-3,10640936	0,00189374	0,00412915
YBR298C	1899,83	0,58	-0,78	-3,10607787	0,00189587	0,00413233
YGR170W	2843,56	0,74	-0,43	-3,1046581	0,00190499	0,00415076
YNL214W	693,36	1,54	0,63	3,10205871	0,0019218	0,00418472
YNL267W	2790,56	0,74	-0,44	-3,10203885	0,00192193	0,00418472
YMR202W	3396,67	0,7	-0,51	-3,10103856	0,00192843	0,00419741
YBL010C	550,89	0,67	-0,58	-3,10032649	0,00193307	0,00420604
YGL247W	422,04	1,54	0,62	3,09984163	0,00193624	0,00421146
YOR350C	777,16	0,68	-0,55	-3,09940682	0,00193909	0,00421617
YJL096W	1003,05	0,69	-0,53	-3,09905426	0,00194139	0,00421971
YPL180W	1480,66	0,72	-0,47	-3,09023844	0,00199996	0,00434549
YPR139C	1500,38	0,72	-0,48	-3,08741606	0,00201905	0,00438543
YKL145W	9753,91	1,33	0,41	3,08718557	0,00202061	0,0043873
YBR039W	5858,6	0,66	-0,59	-3,08698523	0,00202198	0,00438872
YPR010C-A	1073,72	0,54	-0,88	-3,08262241	0,00205185	0,00445201
YCR034W	2134,55	0,68	-0,56	-3,08066822	0,00206537	0,00447977
YPR195C	81	0,42	-1,25	-3,07993025	0,00207049	0,00448932
YHR200W	3193,87	0,73	-0,46	-3,07588236	0,00209881	0,00454912
YBR193C	867,93	0,7	-0,52	-3,07248983	0,00212281	0,00459794
YOR252W	695,81	1,53	0,61	3,07252124	0,00212259	0,00459794
YER041W	849,08	0,7	-0,52	-3,07217399	0,00212506	0,00460002
YML012W	4366,36	0,73	-0,45	-3,07214679	0,00212525	0,00460002
YBL018C	246,35	1,66	0,73	3,07157897	0,0021293	0,00460557
YOR272W	1618,08	1,51	0,6	3,0715929	0,0021292	0,00460557
YNL193W	969,93	0,71	-0,49	-3,06932605	0,00214542	0,00463883
YGR003W	989,4	1,44	0,53	3,06583235	0,00217065	0,00469175
YMR125W	4318,7	1,39	0,48	3,06501831	0,00217657	0,0047029
YJL170C	508,2	0,56	-0,85	-3,062015	0,00219852	0,00474869
YNCF0013W	23,41	4	2	3,06102555	0,0022058	0,00476276
YFL014W	27063,26	5,55	2,47	3,05870065	0,00222299	0,0047982
YER136W	8889,06	1,33	0,41	3,05832496	0,00222578	0,00480255
YER092W	648,7	1,45	0,53	3,05758277	0,0022313	0,00481279
YER118C	1314,72	0,7	-0,51	-3,05666818	0,00223812	0,00482582
YLR071C	1641,87	0,72	-0,47	-3,05557098	0,00224632	0,00484183
YOL124C	843,59	1,57	0,65	3,05544288	0,00224728	0,00484222
YMR064W	1490,14	0,73	-0,45	-3,05490211	0,00225134	0,00484928
YMR109W	4701,39	0,71	-0,49	-3,05402698	0,00225792	0,00486176
YIL095W	1364,05	0,72	-0,48	-3,0513747	0,00227796	0,00490322
YOR246C	1814,07	0,72	-0,47	-3,05115052	0,00227966	0,00490518
YLR060W	9956,04	0,7	-0,5	-3,05007398	0,00228785	0,0049211
YPL213W	619,24	1,45	0,54	3,04899504	0,00229608	0,00493709
YNCK0006C	9,3	10,53	3,4	3,04762955	0,00230654	0,00495787
YJR074W	604,79	0,63	-0,66	-3,04683936	0,00231261	0,0049692
YDL042C	696,28	0,67	-0,57	-3,04585419	0,0023202	0,00498378
YLR127C	1000,95	0,69	-0,53	-3,04566749	0,00232164	0,00498516

YBR097W	955,55	0,69	-0,54	-3,04207107	0,00234956	0,00504336
YGR155W	19348,65	1,5	0,58	3,04190917	0,00235083	0,00504433
YDL131W	3778,52	1,33	0,41	3,03956823	0,00236918	0,00508195
YER017C	2568,75	0,75	-0,42	-3,03935939	0,00237082	0,00508268
YHR022C	110,47	0,51	-0,98	-3,03931688	0,00237115	0,00508268
YDR083W	1410,75	1,49	0,58	3,03865592	0,00237636	0,00509209
YBR204C	1303,38	0,71	-0,49	-3,03736605	0,00238655	0,00511216
YGR262C	1418,8	0,73	-0,45	-3,03676772	0,0023913	0,00512056
YEL040W	3787,46	0,57	-0,82	-3,03480092	0,00240695	0,00515229
YJR147W	979,79	0,67	-0,59	-3,03426475	0,00241123	0,00515968
YMR239C	601,69	1,62	0,7	3,03344739	0,00241777	0,00517033
YMR242C	13381,8	0,75	-0,42	-3,033435	0,00241787	0,00517033
YML006C	957,01	0,66	-0,6	-3,03064319	0,00244033	0,00521657
YGR167W	5428,5	0,76	-0,4	-3,02711297	0,00246902	0,00527607
YNL167C	1954,42	0,72	-0,48	-3,02339899	0,00249952	0,00533942
YKL133C	706,5	0,68	-0,55	-3,02190958	0,00251186	0,00536392
YLL036C	1348,31	0,72	-0,47	-3,02009392	0,00252696	0,00539433
YBR040W	170,27	1,97	0,98	3,00927308	0,00261874	0,00558832
YNCO0015C	44,04	3,09	1,63	3,00866267	0,002624	0,00559763
YKL163W	1044,26	0,72	-0,48	-3,00723828	0,00263633	0,005622
YHR078W	1298,3	0,71	-0,49	-3,00561098	0,00265048	0,00565023
YDR125C	382,25	0,63	-0,67	-3,00378524	0,00266643	0,0056823
YPL196W	1515,01	0,7	-0,52	-3,00342959	0,00266955	0,00568699
YDL081C	21754,09	1,36	0,45	3,00129264	0,00268836	0,00572509
YKL050C	365	1,53	0,62	3,00117669	0,00268938	0,00572531
YNL100W	1873,16	0,64	-0,65	-3,00079131	0,00269279	0,0057306
YMR171C	1851,51	0,74	-0,43	-2,99923689	0,00270657	0,00575794
YNCL0048W	105,22	2,22	1,15	2,99860404	0,0027122	0,00576794
YGL081W	317,94	1,59	0,67	2,99722256	0,00272452	0,00579216
YLR058C	22215,92	0,23	-2,13	-2,99696587	0,00272681	0,00579506
YPR180W	1693,93	1,35	0,44	2,99531729	0,00274159	0,00582448
YDR189W	3675,16	0,72	-0,46	-2,99492436	0,00274513	0,00583
YDL222C	820,16	0,16	-2,63	-2,99391897	0,00275419	0,00584724
YML013W	1944,82	0,74	-0,43	-2,99334851	0,00275934	0,00585618
YKL012W	1891,38	1,35	0,44	2,99312124	0,0027614	0,00585854
YGR092W	1362,32	0,73	-0,44	-2,99031576	0,00278689	0,00591061
YOL039W	20049,61	1,38	0,47	2,98855381	0,00280301	0,00594277
YNCE0017W	764,97	0,68	-0,56	-2,98698352	0,00281745	0,00597134
YDR168W	6132,07	1,39	0,47	2,98652843	0,00282165	0,00597819
YIL163C	86,68	2,06	1,04	2,98639519	0,00282288	0,00597876
YDL160C	2261,53	1,42	0,5	2,98591902	0,00282728	0,00598604
YJL112W	1275,78	0,71	-0,49	-2,9847867	0,00283776	0,00600619
YNL245C	345,5	1,53	0,61	2,98451991	0,00284024	0,00600938
YPL083C	660,31	0,7	-0,52	-2,98322343	0,0028523	0,00603114
YPL179W	2444,63	1,37	0,45	2,98320569	0,00285246	0,00603114
YJL156C	1292,77	0,71	-0,5	-2,98186472	0,00286499	0,00605556
YNL160W	21317,94	0,64	-0,65	-2,98127115	0,00287055	0,00606525
YML125C	2148,15	1,35	0,44	2,98096441	0,00287342	0,00606926
YMR078C	1070,19	0,68	-0,55	-2,97684235	0,00291234	0,00614937
YDR248C	1010,49	0,6	-0,73	-2,97601603	0,0029202	0,00616387

YDR106W	48,68	0,37	-1,44	-2,97536586	0,00292639	0,00617485
YMR274C	309,02	0,64	-0,63	-2,97323063	0,00294683	0,00621586
YGL220W	1349,01	1,4	0,49	2,97079548	0,0029703	0,00626323
YDR408C	3272,96	0,62	-0,68	-2,96885096	0,00298916	0,00630086
YIL098C	905,84	1,46	0,55	2,96817632	0,00299572	0,00631256
YDR339C	1240,73	1,36	0,45	2,96759574	0,00300139	0,00632235
YMR308C	4262,97	0,7	-0,52	-2,9659478	0,00301752	0,00635417
YDR182W	2249,41	0,72	-0,47	-2,96570184	0,00301993	0,0063571
YER145C	2060,76	1,44	0,53	2,96503025	0,00302653	0,00636884
YCL056C	344,83	0,64	-0,64	-2,96430669	0,00303366	0,00638167
YDR139C	1202,79	1,36	0,45	2,96263617	0,00305017	0,00641423
YBR147W	413,82	0,49	-1,02	-2,96034726	0,00307292	0,0064599
YDR437W	151,9	0,55	-0,87	-2,95989588	0,00307743	0,00646718
YER123W	1246,47	0,72	-0,47	-2,95958747	0,00308051	0,00647147
YNR040W	316,14	0,59	-0,75	-2,95804815	0,00309594	0,00650168
YPL152W-A	23,28	4	2	2,95518024	0,00312486	0,00656021
YLR319C	1851,01	1,39	0,48	2,95377119	0,00313917	0,00658801
YLR210W	608,84	1,5	0,59	2,95332541	0,0031437	0,0065953
YJR031C	1131,95	0,7	-0,51	-2,94808063	0,00319754	0,00670597
YNR009W	251,36	0,62	-0,7	-2,94781972	0,00320024	0,00670937
YMR095C	244,65	1,63	0,7	2,94694795	0,00320927	0,00672605
YDL231C	1760,83	1,37	0,46	2,94639017	0,00321507	0,00673592
YLR002C	1713,41	1,51	0,6	2,94490174	0,00323057	0,00676612
YHR172W	578,38	0,66	-0,59	-2,9443334	0,00323651	0,00677628
YML114C	1307,57	0,73	-0,44	-2,94419986	0,00323791	0,00677692
YGR024C	1490,28	1,43	0,52	2,94117186	0,00326973	0,00684121
YMR122W-A	6366,3	0,76	-0,4	-2,9377533	0,003306	0,00691477
YMR016C	2568,06	1,43	0,52	2,93741078	0,00330965	0,00691776
YMR223W	653	0,66	-0,6	-2,93741932	0,00330956	0,00691776
YDR358W	3396,02	0,73	-0,45	-2,935699	0,00332797	0,0069537
YER137C	129,83	1,86	0,9	2,93504913	0,00333495	0,00696594
YOR164C	4229,49	1,33	0,41	2,93392895	0,00334701	0,00698752
YPR202W	82,93	2,13	1,09	2,93388062	0,00334753	0,00698752
YOR080W	1246,04	1,38	0,47	2,93369783	0,0033495	0,00698929
YOR278W	1003,91	1,41	0,49	2,93293742	0,00335772	0,00700407
YOL008W	542,92	0,69	-0,53	-2,93234365	0,00336414	0,00701512
YJR151C	360,46	0,66	-0,61	-2,93124579	0,00337606	0,0070376
YGL128C	858,76	1,41	0,5	2,93105174	0,00337817	0,00703964
YDR035W	8047,73	1,3	0,38	2,93091326	0,00337967	0,00704042
YKL117W	9233,13	0,77	-0,39	-2,93022102	0,00338721	0,00705375
YDR090C	664,62	1,43	0,52	2,92970965	0,00339279	0,007063
YLR162W-A	19,37	4,69	2,23	2,92888378	0,00340182	0,00707942
YPL019C	7988,99	1,49	0,57	2,92628187	0,0034304	0,00713652
YJR135W-A	609,51	1,6	0,68	2,92483474	0,00344639	0,00716553
YML080W	317,17	1,59	0,67	2,92481127	0,00344665	0,00716553
YEL032W	1564,12	0,71	-0,49	-2,92460368	0,00344895	0,00716791
YML004C	5400,1	0,72	-0,47	-2,92431486	0,00345215	0,00717217
YMR271C	660,89	0,61	-0,72	-2,92363762	0,00345967	0,00718538
YBR105C	1800,93	0,69	-0,54	-2,9231822	0,00346474	0,00718883
YDR144C	1241,46	0,6	-0,74	-2,92331362	0,00346327	0,00718883

YDR392W	1333,39	1,36	0,44	2,92317606	0,00346481	0,00718883
YGL233W	2261,55	0,74	-0,44	-2,92241437	0,00347329	0,00720403
YPL234C	3395,6	1,4	0,48	2,92096026	0,00348954	0,00723533
YLR018C	718,1	0,7	-0,51	-2,91772194	0,00352599	0,00730845
YBR182C	255,1	1,64	0,71	2,91700532	0,0035341	0,00732232
YKR036C	994,64	0,73	-0,46	-2,91692256	0,00353504	0,00732232
YER127W	620,2	1,5	0,58	2,91641057	0,00354084	0,0073319
YOR039W	2899,18	0,74	-0,43	-2,91461995	0,00356122	0,00737164
YDR514C	574,06	1,46	0,54	2,91397583	0,00356858	0,00738441
YJL198W	1072,16	1,45	0,53	2,91316336	0,00357787	0,00740118
YOR156C	641,17	0,67	-0,57	-2,91281907	0,00358182	0,00740688
YJL122W	748,97	1,59	0,67	2,91049386	0,00360858	0,00745973
YDR319C	640,78	0,67	-0,57	-2,9076983	0,00364099	0,00752424
YCR007C	308,04	0,6	-0,73	-2,90615198	0,00365904	0,00755901
YDR255C	1226,84	0,74	-0,44	-2,90600306	0,00366078	0,00756009
YCR100C	433,18	1,48	0,57	2,90556609	0,00366589	0,00756814
YBR146W	2243,65	0,72	-0,48	-2,90521674	0,00366999	0,00757408
YJL078C	3789,46	0,73	-0,46	-2,90435795	0,00368007	0,00758984
YLR051C	707,13	1,43	0,52	2,90444732	0,00367902	0,00758984
YOR099W	4070,74	0,75	-0,41	-2,90411444	0,00368294	0,00759323
YFL023W	1010,6	1,4	0,49	2,90298546	0,00369624	0,00761813
YMR283C	763,89	1,44	0,53	2,90226607	0,00370474	0,00763311
YIR023W	1759,1	0,74	-0,43	-2,90180304	0,00371022	0,00764187
YHR211W	484,18	0,67	-0,58	-2,89512402	0,00379009	0,00780379
YPL131W	49282,01	0,76	-0,39	-2,89380371	0,00380606	0,00783408
YIL085C	2008,47	0,73	-0,46	-2,8915009	0,00383406	0,00788911
YOR212W	3389,48	0,76	-0,39	-2,89110552	0,00383889	0,00789642
YMR216C	1748,1	0,71	-0,49	-2,89071039	0,00384372	0,00790112
YMR285C	1682,6	1,36	0,44	2,89076	0,00384311	0,00790112
YJL066C	1935,78	0,7	-0,52	-2,88810595	0,00387569	0,00796421
YDR105C	1220,33	0,67	-0,57	-2,88746529	0,00388359	0,00797517
YHL025W	2352,7	1,45	0,53	2,88750247	0,00388314	0,00797517
YJL047C	933,85	0,72	-0,47	-2,88650241	0,0038955	0,00799697
YNL219C	1745,9	0,73	-0,45	-2,88530397	0,00391036	0,00802483
YDR529C	4848,01	0,63	-0,67	-2,8846335	0,0039187	0,00803928
YKL122C	2290,95	1,34	0,42	2,88220581	0,00394902	0,00809881
YPL265W	2306,89	1,51	0,6	2,88146645	0,00395829	0,00811516
YDL075W	13443,18	1,39	0,47	2,87877626	0,00399221	0,00817516
YDL161W	4610,51	1,32	0,4	2,87893071	0,00399026	0,00817516
YIL097W	1613,71	0,72	-0,46	-2,87872808	0,00399282	0,00817516
YOR360C	1732,51	1,37	0,45	2,87889151	0,00399076	0,00817516
YFL005W	3108,86	0,75	-0,41	-2,87807269	0,00400113	0,00818946
YEL059C-A	132,19	0,45	-1,16	-2,87796601	0,00400248	0,00818953
YBL107C	723,22	0,71	-0,48	-2,87665582	0,00401914	0,0082209
YFL012W	30,03	3,35	1,74	2,87640869	0,00402229	0,00822463
YAL007C	2718,74	0,75	-0,42	-2,87627694	0,00402396	0,00822536
YBR077C	998,68	0,69	-0,54	-2,87507903	0,00403926	0,00825244
YLR135W	737,08	0,7	-0,5	-2,87503158	0,00403987	0,00825244
YKR025W	908,52	1,42	0,5	2,87460834	0,00404529	0,0082608
YMR149W	2992,85	0,76	-0,39	-2,87442435	0,00404765	0,00826289

YDL025C	2656,68	0,75	-0,41	-2,87267997	0,00407006	0,00830592
YEL036C	1934,43	0,72	-0,48	-2,87160861	0,00408388	0,00833139
YNL211C	304,33	1,72	0,78	2,87029621	0,00410087	0,00836331
YHR201C	2590,21	0,76	-0,4	-2,87007832	0,0041037	0,00836633
YDR143C	1703,76	0,75	-0,42	-2,86928147	0,00411406	0,00838468
YBR220C	849,41	0,72	-0,48	-2,86470392	0,00417399	0,00850405
YOR372C	1894,21	0,74	-0,44	-2,86434095	0,00417878	0,00851101
YBR263W	4442,36	0,72	-0,47	-2,86209644	0,00420849	0,0085641
YGL204C	244,35	1,57	0,65	2,86205908	0,00420898	0,0085641
YHL037C	27,75	3,2	1,68	2,86225623	0,00420637	0,0085641
YIR041W	28,25	3,18	1,67	2,86175796	0,00421299	0,00856944
YBR240C	115,53	2	1	2,85990485	0,00423768	0,00861685
YDL120W	1109,32	0,71	-0,5	-2,85809557	0,00426192	0,0086633
YLR181C	1491,55	1,35	0,44	2,85661119	0,0042819	0,00870107
YLL016W	1182,66	0,67	-0,58	-2,85515527	0,00430158	0,0087382
YPL232W	3346,93	0,76	-0,4	-2,85472381	0,00430743	0,00874722
YGR132C	4338,86	0,7	-0,51	-2,85450741	0,00431036	0,00874746
YLR090W	1077,92	0,72	-0,47	-2,85450839	0,00431035	0,00874746
YHR014W	106,23	1,99	0,99	2,85208663	0,00434333	0,00881148
YDR410C	653,03	0,71	-0,5	-2,85106866	0,00435726	0,00883685
YKL171W	1751,23	0,73	-0,46	-2,85021025	0,00436903	0,00885785
YPL194W	409,36	0,65	-0,61	-2,84979952	0,00437468	0,0088664
YGR140W	718,99	0,71	-0,5	-2,84908575	0,00438451	0,00888263
YHR001W-A	2196,48	1,58	0,66	2,84901028	0,00438555	0,00888263
YNCE0020C	10,91	7,47	2,9	2,84861887	0,00439094	0,00889066
YFR036W	206,39	1,64	0,72	2,84849312	0,00439268	0,00889128
YML092C	4074,63	1,31	0,39	2,84794709	0,00440022	0,00890365
YLL009C	615,35	0,55	-0,86	-2,84693006	0,00441431	0,00892923
YAL015C	955,33	1,41	0,49	2,84607381	0,00442619	0,0089464
YGL168W	23,18	0,24	-2,03	-2,84600753	0,00442711	0,0089464
YIL051C	11129,26	0,72	-0,48	-2,84605777	0,00442642	0,0089464
YKL124W	1477,23	1,41	0,5	2,84577653	0,00443033	0,00894707
YMR301C	1378,5	0,74	-0,44	-2,84580376	0,00442995	0,00894707
YDR419W	723,35	0,71	-0,49	-2,84506451	0,00444024	0,00896418
YGR278W	547,62	0,7	-0,52	-2,84215823	0,00448092	0,00904337
YPR028W	7349,09	1,47	0,55	2,84119702	0,00449445	0,00906773
YDR303C	2079,53	1,52	0,61	2,84039569	0,00450576	0,00908759
YIR012W	1820,93	1,47	0,56	2,83703586	0,00455345	0,0091808
YBR056W-A	395,13	1,7	0,76	2,83403248	0,00459647	0,00926452
YOR061W	3331,76	0,76	-0,39	-2,83363295	0,00460222	0,0092731
YLR340W	80000,23	1,33	0,41	2,83186217	0,00462778	0,00932158
YNL304W	749,4	1,38	0,47	2,83123673	0,00463684	0,0093368
YGL033W	68,56	2,53	1,34	2,83010626	0,00465325	0,00936682
YHR178W	801,77	1,39	0,47	2,82872951	0,00467332	0,00940416
YGR004W	515,95	0,69	-0,53	-2,82376258	0,00474635	0,00954804
YML002W	873,77	0,72	-0,47	-2,82358158	0,00474903	0,00955034
YDR520C	604,38	0,69	-0,53	-2,82265238	0,00476282	0,00957496
YKR051W	1029,85	0,72	-0,47	-2,81822737	0,00482896	0,00970479
YKR074W	1422,39	0,69	-0,53	-2,81759658	0,00483846	0,00972073
YKR085C	800,06	0,69	-0,53	-2,81697098	0,00484789	0,00973653

YDR359C	1394,46	0,72	-0,46	-2,81680339	0,00485042	0,00973847
YNCE0016C	6,37	38,87	5,28	2,81632577	0,00485764	0,00974981
YGR294W	39,68	2,67	1,42	2,81115344	0,00493642	0,00990474
YLR030W	171,73	0,58	-0,79	-2,80760206	0,00499119	0,01001138
YJL207C	2680,22	1,41	0,49	2,80527375	0,00502739	0,01008074
YLR004C	193,97	0,6	-0,74	-2,80497803	0,005032	0,01008673
YEL069C	111,74	1,82	0,86	2,8022997	0,00507397	0,01016431
YLR364W	269,84	1,55	0,63	2,8023897	0,00507256	0,01016431
YHR135C	4891,55	0,77	-0,38	-2,80123002	0,00509082	0,01019193
YNL291C	887,01	0,7	-0,52	-2,80121597	0,00509104	0,01019193
YMR286W	855,51	1,48	0,57	2,80090348	0,00509598	0,01019852
YFL010C	2353,45	0,76	-0,39	-2,79995422	0,00511099	0,01022526
YDR089W	2386,93	0,73	-0,46	-2,79734658	0,00515242	0,01030485
YFL029C	1313,4	1,41	0,5	2,7966223	0,00516399	0,01032465
YMR040W	268,07	1,68	0,75	2,79624443	0,00517003	0,0103334
YNL186W	1867,26	1,36	0,44	2,79589788	0,00517558	0,01034116
YHR077C	3898,69	1,33	0,41	2,79446287	0,0051986	0,01038383
YDL091C	2137,56	1,37	0,45	2,79360771	0,00521237	0,01040798
YOR168W	7891,59	1,4	0,49	2,79068383	0,00525968	0,01049908
YIL130W	1368,63	0,75	-0,41	-2,7885369	0,00529467	0,01056553
YHR127W	703,69	0,7	-0,51	-2,7880506	0,00530263	0,01057801
YLR414C	2892,47	1,35	0,43	2,78669954	0,00532478	0,0106188
YGR062C	732,81	0,64	-0,65	-2,78559387	0,00534298	0,01064824
YPR004C	2195,39	0,67	-0,59	-2,78567793	0,00534159	0,01064824
YMR264W	1379,71	0,72	-0,47	-2,78415353	0,00536676	0,01069222
YGR124W	21520,18	1,39	0,48	2,78150215	0,0054108	0,01077649
YIL009W	689,92	1,49	0,57	2,78052529	0,0054271	0,0108055
YBR151W	5727,73	0,74	-0,43	-2,78009867	0,00543424	0,01081624
YGR188C	347,05	1,51	0,6	2,77985238	0,00543836	0,01082098
YMR169C	2236,06	0,21	-2,26	-2,77853904	0,0054604	0,01086134
YMR155W	716,16	0,7	-0,51	-2,77626229	0,00549878	0,0109342
YBR165W	937,35	1,4	0,48	2,77572611	0,00550786	0,01094874
YPR119W	597,9	0,66	-0,6	-2,77425861	0,00553277	0,01099474
YOL086W-A	410,32	0,66	-0,6	-2,77190356	0,00557295	0,01107106
YBL080C	627,11	0,68	-0,56	-2,7705106	0,00559685	0,01111142
YJL072C	381,11	0,68	-0,55	-2,77052305	0,00559663	0,01111142
YCR046C	1114,93	0,64	-0,64	-2,77022555	0,00560175	0,01111759
YBR010W	6266,07	1,29	0,37	2,76957312	0,00561298	0,01113633
YPL094C	2500,85	0,76	-0,4	-2,76675517	0,00566173	0,01122945
YKL072W	916,44	1,48	0,56	2,76502744	0,0056918	0,0112855
YBL031W	462,97	0,69	-0,53	-2,76476344	0,00569641	0,01129103
YPL233W	497,66	0,69	-0,53	-2,76411153	0,0057078	0,01131001
YCL032W	952,67	0,72	-0,47	-2,76240445	0,00573774	0,01136569
YDL052C	2424,99	0,76	-0,39	-2,76119699	0,00575899	0,01140416
YDL245C	78,45	0,48	-1,06	-2,75908251	0,00579639	0,01147224
YOR100C	204,35	2,08	1,06	2,75904463	0,00579706	0,01147224
YCR026C	1474,65	0,75	-0,42	-2,75770317	0,0058209	0,01151575
YKL089W	522,95	1,43	0,52	2,75755935	0,00582346	0,01151715
YEL018W	862,49	1,38	0,46	2,75726201	0,00582876	0,01152396
YHR121W	1937,94	0,76	-0,4	-2,75566963	0,00585721	0,01157652

YLR367W	5526,58	0,75	-0,41	-2,75488018	0,00587136	0,0116008
YDR268W	713,52	0,7	-0,51	-2,75346321	0,00589684	0,01164743
YPL254W	641,73	0,72	-0,47	-2,75330874	0,00589962	0,01164922
YAL044C	5246,47	0,19	-2,41	-2,75150796	0,00593216	0,01170974
YCR045C	442,48	0,66	-0,59	-2,75129029	0,0059361	0,01171381
YDR059C	713,1	0,64	-0,64	-2,75077566	0,00594543	0,0117285
YPL188W	1262,3	0,74	-0,43	-2,75043001	0,00595171	0,01173715
YNCJ0030W	61,01	2,23	1,16	2,74669085	0,00601999	0,01186803
YIR026C	1534,7	1,47	0,55	2,74556095	0,00604075	0,01190519
YKL134C	618,77	0,71	-0,5	-2,74437347	0,00606265	0,01194456
YPR198W	1167,97	0,74	-0,44	-2,74397593	0,00607	0,01195524
YNCO0027C	208,49	1,78	0,84	2,7426134	0,00609524	0,01200115
YMR017W	87,29	1,97	0,98	2,74165332	0,00611308	0,01203247
YGR174W-A	124,75	0,44	-1,19	-2,74021333	0,00613993	0,01208149
YBR068C	8527,38	0,77	-0,38	-2,74009422	0,00614216	0,01208204
YHR187W	624,29	0,72	-0,48	-2,73950146	0,00615324	0,01210002
YJL157C	1127,2	0,71	-0,5	-2,73917996	0,00615926	0,01210802
YDL035C	2199,12	0,75	-0,41	-2,73859358	0,00617026	0,0121258
YJL080C	7888,28	0,66	-0,6	-2,73734202	0,00619379	0,01216818
YHR143W-A	813,37	1,37	0,45	2,73485142	0,00624084	0,01225288
YNL078W	2374,66	1,35	0,43	2,73493795	0,0062392	0,01225288
YHR112C	1884,77	0,72	-0,47	-2,73440787	0,00624926	0,01226553
YNR059W	784,01	0,73	-0,45	-2,73337742	0,00626885	0,01230008
YGR246C	1709,39	1,31	0,39	2,73321311	0,00627197	0,01230234
YBL017C	6126,62	1,29	0,37	2,73298758	0,00627627	0,01230688
YMR175W	322,89	5,79	2,53	2,73208056	0,00629357	0,01233356
YNL072W	480,49	0,68	-0,55	-2,73206621	0,00629385	0,01233356
YBR278W	647,35	1,52	0,6	2,73139444	0,00630669	0,01235484
YER129W	2723,14	0,75	-0,42	-2,72936503	0,00634564	0,01242722
YDR238C	7244,02	0,72	-0,48	-2,72626747	0,00640551	0,0125405
YGL087C	2120,28	0,69	-0,53	-2,72457705	0,00643839	0,01260091
YDR019C	4271,42	0,22	-2,19	-2,72414176	0,00644688	0,01261355
YOL061W	2550,23	0,76	-0,39	-2,72388537	0,00645189	0,01261938
YGR231C	4412,7	0,75	-0,42	-2,72342683	0,00646085	0,01263293
YDR317W	288,81	0,63	-0,66	-2,72238166	0,00648132	0,01266807
YJL136W-A	19,42	4,05	2,02	2,72230121	0,0064829	0,01266807
YGL167C	2756,5	0,7	-0,52	-2,72187376	0,00649129	0,01268048
YPL199C	2727,04	1,31	0,39	2,72038081	0,00652068	0,01273387
YER058W	421,43	1,52	0,6	2,71983566	0,00653144	0,01275088
YLR055C	867,4	0,73	-0,46	-2,71813077	0,00656519	0,01281274
YJL087C	712,86	1,48	0,57	2,71798733	0,00656804	0,01281427
YGR250C	7586,03	0,77	-0,39	-2,71510029	0,00662557	0,01292246
YIL008W	990,57	0,71	-0,5	-2,71250225	0,00667773	0,01302011
YPL105C	4550,04	1,31	0,39	2,71013403	0,0067256	0,01310933
YPR030W	2262,21	0,31	-1,69	-2,70759286	0,00677731	0,01320598
YDR072C	2154,7	1,31	0,39	2,70728549	0,00678359	0,01321407
YHL036W	1443,34	3,32	1,73	2,70626983	0,00680437	0,0132504
YBL099W	14328,9	0,74	-0,43	-2,70508099	0,00682877	0,01329375
YHR196W	1104,85	0,7	-0,51	-2,70410542	0,00684886	0,0133264
YKL009W	2617,59	1,46	0,54	2,70405778	0,00684984	0,0133264

YDR318W	816,77	1,37	0,46	2,70353613	0,0068606	0,01334316
YGL190C	2523,43	0,77	-0,37	-2,70321048	0,00686732	0,01335206
YCL064C	6524,98	6,48	2,7	2,70251335	0,00688174	0,0133759
YDR279W	842,74	1,38	0,46	2,69785139	0,00697886	0,01356042
YJR065C	8046,01	0,78	-0,35	-2,69729965	0,00699043	0,01357867
YLR291C	2705,55	1,35	0,44	2,69640969	0,00700914	0,01361075
YDR424C	1140,98	1,37	0,45	2,69584545	0,00702102	0,01362956
YDR129C	12323,61	0,78	-0,35	-2,69308209	0,00707948	0,01373446
YMR091C	2063,12	0,73	-0,44	-2,6931374	0,00707831	0,01373446
YNL097C-B	105,16	1,84	0,88	2,69269946	0,00708761	0,01374594
YKL051W	1893,53	0,77	-0,38	-2,69214708	0,00709936	0,01376443
YDL033C	361,85	0,69	-0,54	-2,69166583	0,00710961	0,01378001
YLR246W	586,91	0,67	-0,58	-2,68972064	0,00715119	0,01385626
YHR005C-A	2695,57	0,76	-0,4	-2,6890717	0,0071651	0,0138789
YGR295C	2838,32	0,77	-0,38	-2,68736653	0,00720179	0,01394561
YDR159W	1676,74	0,74	-0,43	-2,68655566	0,00721929	0,01397515
YFR031C-A	20078,4	0,76	-0,39	-2,68528142	0,00724688	0,01402417
YER188C-A	39,48	0,36	-1,47	-2,68340263	0,00728772	0,01409882
YCR023C	1802,12	0,74	-0,43	-2,682529	0,00730678	0,01410647
YDL145C	10277,18	0,73	-0,46	-2,68263994	0,00730436	0,01410647
YGL243W	833,34	1,35	0,43	2,6828052	0,00730075	0,01410647
YHL048W	1085,73	0,74	-0,43	-2,68281447	0,00730055	0,01410647
YMR267W	1233,7	0,7	-0,52	-2,68249298	0,00730757	0,01410647
YPL152W	1251,33	1,32	0,4	2,68271794	0,00730266	0,01410647
YPR118W	2609,75	0,71	-0,49	-2,68279048	0,00730107	0,01410647
YOR229W	572,87	0,69	-0,54	-2,67966411	0,00736961	0,01422181
YPL060W	639,72	1,43	0,52	2,67827974	0,00740014	0,01427629
YOL139C	7348,35	0,76	-0,4	-2,67750829	0,0074172	0,01430296
YOR217W	1988,08	0,73	-0,45	-2,67744674	0,00741856	0,01430296
YDR022C	377,94	0,66	-0,61	-2,67676967	0,00743357	0,01432744
YNCG0024W	102,5	1,84	0,88	2,67612367	0,00744792	0,01435064
YLL021W	3363,65	1,41	0,5	2,67569644	0,00745742	0,01436449
YNL224C	1439,06	1,32	0,4	2,67557621	0,00746009	0,01436518
YNL210W	133,53	1,77	0,82	2,67418794	0,00749105	0,01442032
YJL011C	262,02	1,56	0,64	2,67220211	0,00753552	0,01450145
YGL018C	407,93	0,63	-0,67	-2,66905564	0,00760648	0,01462624
YKR059W	10997,43	0,75	-0,41	-2,66910538	0,00760536	0,01462624
YPR100W	42,09	0,39	-1,37	-2,66901372	0,00760743	0,01462624
YDR525W	12,9	5,19	2,38	2,66796102	0,00763131	0,01466307
YNCJ0004C	67,16	2,65	1,41	2,668039	0,00762954	0,01466307
YGL185C	669,48	0,72	-0,47	-2,66607097	0,00767435	0,0147412
YGR012W	1222,29	1,35	0,44	2,66285948	0,00774798	0,01487803
YGR119C	3150,42	0,76	-0,39	-2,66227094	0,00776154	0,01489947
YKL173W	1660,66	0,74	-0,44	-2,66118582	0,0077866	0,01494295
YMR080C	2887,93	0,72	-0,47	-2,6604083	0,0078046	0,01497287
YLR113W	6062,99	0,79	-0,35	-2,66007795	0,00781226	0,01498294
YHR155W	1005,52	0,73	-0,45	-2,65918705	0,00783295	0,01501798
YGL262W	20,17	0,24	-2,07	-2,65882346	0,0078414	0,01502956
YAL016W	5782,27	1,28	0,35	2,65805672	0,00785927	0,01505646
YAL044W-A	699,06	0,7	-0,52	-2,65801294	0,00786029	0,01505646

YNL298W	991,57	0,73	-0,44	-2,65712807	0,00788095	0,01509139
YPR063C	2574,65	0,75	-0,42	-2,65698759	0,00788423	0,01509302
YPL221W	2129,25	1,34	0,42	2,65640166	0,00789795	0,01511462
YDR422C	1085,62	0,73	-0,46	-2,65532865	0,00792312	0,01515812
YDR481C	4835,52	1,29	0,37	2,65381079	0,00795884	0,01522178
YNL163C	2725,01	1,38	0,47	2,65353759	0,00796529	0,01522942
YOL022C	1716,9	1,33	0,41	2,6513435	0,00801723	0,015324
YNL202W	279,89	0,57	-0,82	-2,65059516	0,00803501	0,01535327
YOL093W	1150,75	1,33	0,41	2,64980995	0,00805371	0,01538426
YJL099W	841,01	1,36	0,45	2,64669067	0,00812837	0,01552211
YPL097W	1060,5	1,34	0,42	2,6425902	0,00822745	0,0157065
YML008C	10401,38	1,31	0,39	2,64105601	0,00826481	0,01577296
YPL022W	1948,59	0,75	-0,41	-2,64023219	0,00828492	0,0158065
YNL339C	27,19	0,31	-1,68	-2,64012014	0,00828766	0,01580687
YDL002C	1039,57	1,35	0,43	2,63969188	0,00829814	0,015822
YOL123W	7321,95	1,32	0,4	2,63941122	0,00830502	0,01583024
YIL120W	399,15	0,69	-0,54	-2,63878088	0,00832047	0,01585484
YPR005C	813,05	1,38	0,46	2,6316699	0,00849664	0,01618556
YOR279C	569,77	1,38	0,46	2,62814679	0,00858515	0,01634915
YKL197C	1173,13	0,73	-0,45	-2,62483301	0,00866915	0,01650406
YOR308C	855,62	1,36	0,45	2,62463955	0,00867407	0,01650838
YNCN0018W	440,78	1,55	0,64	2,6242624	0,00868369	0,01652161
YKL001C	805,19	1,66	0,73	2,62405015	0,0086891	0,01652685
YDR283C	1834,33	0,75	-0,41	-2,62022167	0,00878726	0,01670844
YGR154C	464,12	1,41	0,5	2,61770302	0,00885238	0,01682711
YDR414C	607,57	0,71	-0,49	-2,61661072	0,00888076	0,01686793
YNCL0043C	69,51	0,49	-1,04	-2,61656271	0,008882	0,01686793
YPL090C	14453,59	1,34	0,43	2,61668327	0,00887887	0,01686793
YLL015W	2487,72	0,76	-0,39	-2,61588075	0,00889976	0,01689288
YNCD0030W	88,55	0,48	-1,04	-2,61584947	0,00890058	0,01689288
YDR288W	927,09	0,69	-0,53	-2,61540225	0,00891224	0,01690985
YJL214W	251,69	0,65	-0,61	-2,61366821	0,0089576	0,01699072
YBR233W-A	409,16	0,7	-0,51	-2,61187172	0,0090048	0,01707504
YPR053C	35,66	0,37	-1,42	-2,61156834	0,0090128	0,01708499
YJR055W	164,61	1,61	0,69	2,61066195	0,00903672	0,0171241
YML045W	223,4	0,65	-0,62	-2,61057764	0,00903895	0,0171241
YER057C	3889,77	0,78	-0,36	-2,61035931	0,00904472	0,01712981
YIL039W	3742,38	0,77	-0,39	-2,61024458	0,00904775	0,01713033
YDR246W-A	96,83	1,83	0,87	2,61013125	0,00905075	0,01713079
YDR029W	443,62	0,65	-0,62	-2,6091945	0,00907556	0,01717253
YLR330W	9174,44	1,27	0,34	2,60901747	0,00908026	0,01717618
YPL190C	3763,06	0,76	-0,4	-2,60865017	0,00909001	0,01718939
YCL019W	48,46	2,59	1,38	2,60752712	0,00911988	0,01724063
YDR046C	3509,51	0,72	-0,47	-2,60684806	0,00913799	0,01726961
YNL053W	573,23	0,71	-0,49	-2,60446738	0,00920172	0,01738475
YMR197C	1609,32	0,77	-0,38	-2,60378259	0,00922012	0,01741423
YJL054W	2264,53	1,29	0,37	2,60297692	0,00924182	0,0174499
YNL044W	1618,51	0,76	-0,39	-2,60099112	0,00929549	0,0175459
YBR011C	37350,37	1,27	0,35	2,60023287	0,00931605	0,01757404
YNL142W	387,84	1,54	0,63	2,6002654	0,00931517	0,01757404

YOR288C	1594,32	0,74	-0,43	-2,60012319	0,00931903	0,01757432
YMR111C	643,99	1,36	0,44	2,60001548	0,00932196	0,0175745
YDL126C	27617,36	1,28	0,36	2,59951412	0,00933558	0,01759486
YDR284C	1830,19	0,71	-0,5	-2,59895778	0,00935073	0,01760677
YGR187C	1235,6	1,43	0,51	2,59905195	0,00934816	0,01760677
YML009C	774,18	0,69	-0,53	-2,59905788	0,009348	0,01760677
YPL191C	522,52	0,72	-0,47	-2,59886551	0,00935324	0,01760677
YDR301W	1944,81	1,36	0,45	2,59739059	0,0093935	0,0176772
YGL226C-A	1145,32	1,31	0,39	2,59615705	0,0094273	0,01773542
YKL137W	833,19	0,69	-0,53	-2,59555282	0,00944389	0,01776126
YML081C-A	1514,1	0,66	-0,59	-2,59439453	0,00947577	0,01781582
YJR058C	794,65	0,74	-0,43	-2,59380971	0,0094919	0,01783907
YNCN0019C	39,93	2,6	1,38	2,59373821	0,00949387	0,01783907
YGL023C	1567,04	0,74	-0,44	-2,58754185	0,00966635	0,01815765
YHR066W	880,38	1,51	0,59	2,58626402	0,00970226	0,0182196
YNL122C	712,94	0,69	-0,54	-2,58592323	0,00971186	0,01823211
YFL001W	653,69	0,72	-0,48	-2,58571565	0,00971771	0,01823758
YNL195C	701,63	0,27	-1,87	-2,58318629	0,00978924	0,01836629
YBR112C	5716,67	0,77	-0,37	-2,58228174	0,00981494	0,01840894
YHR120W	618,11	1,46	0,55	2,58200317	0,00982287	0,01841825
YHR030C	5791,69	1,45	0,54	2,581584	0,00983481	0,01843507
YJL008C	5815,11	1,4	0,49	2,57838021	0,00992647	0,01860128
YDR110W	917,87	1,38	0,46	2,57705037	0,00996474	0,01866737
YNL216W	1578,98	0,75	-0,41	-2,5742934	0,01004451	0,01881111
YDL208W	3101,03	1,28	0,36	2,57384865	0,01005743	0,01882964
YMR013C	589,69	0,72	-0,47	-2,57322707	0,01007551	0,01885781
YHR204W	1123,05	0,72	-0,46	-2,5716824	0,01012057	0,01893644
YLR072W	1180,32	0,76	-0,4	-2,57139333	0,01012902	0,01894655
YBR158W	4829,87	0,79	-0,35	-2,5702161	0,01016351	0,01900533
YMR029C	2028,24	0,77	-0,37	-2,56985533	0,0101741	0,01901941
YCR065W	507,83	0,7	-0,52	-2,56740654	0,01024624	0,01914275
YNR029C	1252,65	1,33	0,41	2,56750432	0,01024335	0,01914275
YJL065C	1007,87	0,68	-0,57	-2,56564987	0,01029827	0,01923418
YOR160W	1504,22	0,72	-0,48	-2,5634432	0,01036397	0,01935105
YGR061C	8260,48	0,79	-0,34	-2,5629384	0,01037905	0,01936757
YGR285C	21381,35	1,35	0,43	2,5630108	0,01037688	0,01936757
YOL038C-A	110,76	0,57	-0,82	-2,56205948	0,01040535	0,01941082
YEL053C	1912,04	0,76	-0,39	-2,56175668	0,01041443	0,01942192
YLR011W	527,29	1,41	0,49	2,55955789	0,01048054	0,01953935
YNL030W	7669,92	0,79	-0,35	-2,55595388	0,01058972	0,01973697
YHR061C	302,84	1,49	0,57	2,55565094	0,01059894	0,01974823
YGL251C	767,16	0,74	-0,43	-2,55403441	0,01064827	0,01983421
YOR193W	648,08	1,36	0,44	2,55140562	0,01072894	0,01997847
YLL017W	122,94	0,56	-0,84	-2,5510158	0,01074095	0,01999483
YLL063C	391,54	1,48	0,57	2,55058547	0,01075422	0,02001354
YNL326C	413,47	0,7	-0,52	-2,55005744	0,01077052	0,02003787
YJL138C	9987,25	0,75	-0,41	-2,54922389	0,0107963	0,02007982
YER175C	1482,98	1,45	0,53	2,54911494	0,01079967	0,02008008
YKL183C-A	24,4	3,44	1,78	2,5477373	0,01084241	0,02015351
YGR157W	7305,78	1,49	0,58	2,54524289	0,01092017	0,02029199

YGR217W	681,26	1,49	0,57	2,5446156	0,01093981	0,02031632
YIL103W	877,52	1,34	0,42	2,54467406	0,01093798	0,02031632
YDR352W	952,73	1,32	0,4	2,54083863	0,01105869	0,02053097
YLR097C	1124,48	0,73	-0,45	-2,54043602	0,01107143	0,02054848
YDL169C	818,7	1,55	0,63	2,54005042	0,01108365	0,020565
YIL021W	3176,22	1,27	0,34	2,53948111	0,0111017	0,02059235
YBR140C	3407,45	0,77	-0,37	-2,53787729	0,01115271	0,02068078
YAL043C	1811,11	0,77	-0,37	-2,53772453	0,01115758	0,02068353
YNCN0010C	3,31	55,28	5,79	2,53762185	0,01116085	0,02068353
YER135C	35,46	0,35	-1,53	-2,53577927	0,01121974	0,02078647
YIL096C	491,04	1,48	0,57	2,5317697	0,01134885	0,0210194
YNL091W	6409,99	1,26	0,33	2,52918602	0,01143274	0,02116846
YNCL0018W	69,83	2,05	1,04	2,52841488	0,01145789	0,0212087
YMR211W	526,39	1,41	0,49	2,52710222	0,0115008	0,02128179
YHR151C	381,5	0,7	-0,52	-2,52629188	0,01152737	0,02132141
YPR031W	463,31	0,71	-0,48	-2,52623984	0,01152907	0,02132141
YNL081C	1635,32	1,34	0,42	2,52472126	0,01157901	0,02140738
YLR185W	8673,93	1,33	0,42	2,5237212	0,011612	0,02146198
YHR193C	15279,93	1,26	0,34	2,5232556	0,01162739	0,02148403
YOR111W	874,94	1,35	0,44	2,52043946	0,01172084	0,02165027
YMR044W	2071,03	1,28	0,36	2,51882978	0,01177456	0,02174303
YGL203C	2779,64	0,77	-0,38	-2,51743869	0,01182115	0,02181738
YMR259C	1474,15	1,35	0,44	2,51741817	0,01182184	0,02181738
YML054C	835,07	0,27	-1,87	-2,51627033	0,01186042	0,02188206
YPR185W	2455,5	0,78	-0,35	-2,51495933	0,01190461	0,02195708
YBR186W	148,66	0,59	-0,75	-2,51131805	0,01202813	0,02217831
YLR398C	2546,61	0,73	-0,45	-2,50997789	0,01207387	0,02225605
YPL016W	1432,51	1,31	0,39	2,50962088	0,01208608	0,02227195
YCR072C	416,51	1,56	0,64	2,50905219	0,01210556	0,02230123
YOL164W	772,77	1,36	0,44	2,50856198	0,01212237	0,02232557
YPL039W	442,51	0,67	-0,57	-2,50815823	0,01213623	0,02234448
YIL161W	876,97	1,36	0,44	2,50793736	0,01214382	0,02235182
YOR363C	1233,23	0,75	-0,42	-2,50772874	0,01215099	0,0223584
YLR287C	1225	1,39	0,47	2,5074529	0,01216048	0,02236923
YCL057C-A	845,62	0,65	-0,61	-2,50694198	0,01217807	0,02239496
YDR310C	2298,51	0,77	-0,38	-2,50587567	0,01221486	0,02245596
YLR425W	1347,69	0,75	-0,42	-2,50576532	0,01221867	0,02245632
YGR243W	2384,68	4,04	2,01	2,50293812	0,01231671	0,02262981
YDR228C	1656,48	1,28	0,36	2,50273302	0,01232385	0,02263623
YIR001C	1295,48	0,76	-0,4	-2,49877258	0,01246243	0,02288401
YGR059W	76,32	0,5	-1,01	-2,49866204	0,01246631	0,02288438
YNCN0008C	17,74	0,25	-2,02	-2,4975387	0,01250588	0,02295023
YDR460W	928,01	1,36	0,44	2,4963576	0,0125476	0,02302
YOR290C	4655,61	1,27	0,34	2,49459866	0,01260997	0,02312758
YDR085C	2291	0,77	-0,37	-2,49336939	0,01265371	0,02320096
YHR033W	295,08	1,57	0,65	2,49190077	0,01270615	0,02329024
YCR018C	755,2	1,38	0,47	2,49161394	0,01271642	0,02330081
YGR288W	546,11	0,7	-0,51	-2,49152998	0,01271942	0,02330081
YIL075C	14018,07	1,32	0,4	2,49119878	0,01273129	0,02331567
YCL008C	1539,4	0,76	-0,39	-2,49108302	0,01273544	0,02331574

YNR052C	1730,77	0,76	-0,4	-2,49098833	0,01273883	0,02331574
YPL045W	1634,98	1,29	0,37	2,48942941	0,01279483	0,02341134
YLR336C	1161,45	1,42	0,51	2,48788629	0,01285048	0,02350624
YJL178C	1898,68	0,78	-0,37	-2,48300514	0,01302792	0,0238238
YDR349C	2719,64	1,28	0,36	2,48284386	0,01303382	0,02382757
YGR109W-B	1289,64	1,29	0,37	2,48238772	0,01305052	0,02385108
YPL050C	2941,64	0,76	-0,4	-2,4806582	0,01311401	0,02396006
YDR244W	1339,01	0,77	-0,38	-2,48010941	0,01313421	0,02398991
YBR111W-A	765,67	1,41	0,49	2,47954667	0,01315495	0,02402074
YNCP0014C	88,3	0,51	-0,96	-2,47877349	0,0131835	0,0240658
YDR225W	7843,44	0,8	-0,32	-2,47692719	0,01325189	0,02418354
YOR224C	2942,66	1,35	0,43	2,47648317	0,01326839	0,02420653
YNL250W	1155,13	1,39	0,47	2,47405922	0,01335875	0,02436424
YGR223C	1898,45	1,29	0,37	2,47328344	0,01338779	0,02441003
YBR176W	603,32	1,37	0,45	2,47208178	0,01343288	0,02447069
YGL014W	2532,49	1,34	0,42	2,47217896	0,01342923	0,02447069
YKR028W	3190,99	1,33	0,42	2,47218059	0,01342917	0,02447069
YIR021W	452,67	0,69	-0,54	-2,47102833	0,01347251	0,0245357
YML112W	734,45	0,75	-0,42	-2,46985072	0,01351694	0,0246094
YDR328C	4530,26	1,36	0,45	2,46551888	0,0136815	0,02490169
YIL150C	385,17	1,49	0,57	2,46537407	0,01368703	0,02490446
YPL195W	4445,09	1,28	0,36	2,46484678	0,01370719	0,02493383
YKL013C	3677,51	0,8	-0,33	-2,46128017	0,01384422	0,02517573
YDL114W	17,94	4,06	2,02	2,46035125	0,01388011	0,02523361
YNL164C	368,37	1,42	0,51	2,45954889	0,01391117	0,02528269
YKL217W	490,67	0,65	-0,62	-2,45873296	0,01394283	0,0253328
YDR031W	424,91	0,57	-0,82	-2,45799921	0,01397135	0,0253772
YGR204W	23548,33	1,26	0,33	2,45732481	0,01399761	0,02541746
YOR312C	18245,39	1,27	0,34	2,45594709	0,01405138	0,02550766
YLR146C	582,92	0,74	-0,44	-2,45579321	0,0140574	0,02551113
YNL191W	792,27	0,67	-0,57	-2,4554352	0,01407141	0,02552909
YBL102W	1226,23	0,76	-0,39	-2,45489665	0,01409251	0,0255599
YIL001W	604,84	0,74	-0,44	-2,45433273	0,01411463	0,02559255
YOL015W	389,22	0,69	-0,54	-2,4537619	0,01413706	0,02562573
YKL104C	5356,27	0,76	-0,39	-2,45361966	0,01414265	0,02562839
YDL137W	9120,26	1,34	0,42	2,45337634	0,01415222	0,02563826
YFR018C	2064,7	1,28	0,35	2,45236265	0,01419215	0,02570311
YIL102C-A	200,93	1,53	0,62	2,45121173	0,01423762	0,02577793
YLR015W	855,84	1,36	0,44	2,45056557	0,0142632	0,02581672
YGL103W	23538,77	1,27	0,34	2,44970655	0,01429727	0,02587085
YLR032W	834,13	0,7	-0,51	-2,44669591	0,01441724	0,02608035
YMR101C	44,71	2,35	1,23	2,44637928	0,01442991	0,02609566
YKL093W	1120,66	0,58	-0,78	-2,44370978	0,01453712	0,02628189
YKR027W	1074,14	0,76	-0,4	-2,44348423	0,01454621	0,02629067
YOL087C	3570,45	0,79	-0,34	-2,44246095	0,01458751	0,02635765
YBL103C	1637,8	1,28	0,36	2,44042438	0,01467002	0,02649336
YEL004W	471,77	0,73	-0,46	-2,44039662	0,01467114	0,02649336
YDR101C	1702,24	1,45	0,54	2,43848594	0,01474893	0,02662609
YBR202W	1162,21	0,76	-0,39	-2,43580715	0,0148586	0,02681628
YLR020C	1872,47	1,33	0,42	2,43514348	0,01488589	0,02685772

YPL137C	1470,62	1,42	0,5	2,43318957	0,01496646	0,02699526
YHR119W	1690,64	0,77	-0,37	-2,43146169	0,01503804	0,02711649
YNL161W	2567,74	1,27	0,35	2,43050483	0,0150778	0,0271803
YMR004W	2457,52	1,26	0,34	2,42839413	0,01516585	0,02733109
YIL018W	27858,27	0,8	-0,32	-2,42792392	0,01518553	0,02735861
YPL047W	451,78	1,37	0,45	2,4267571	0,01523445	0,0274388
YDL090C	937,21	0,74	-0,43	-2,42626475	0,01525514	0,02746809
YKL054C	9323,31	0,78	-0,37	-2,42491403	0,01531201	0,02756251
YBR058C-A	1355,47	1,42	0,51	2,42450177	0,01532941	0,02758583
YOR103C	1412,36	0,78	-0,37	-2,42391215	0,01535432	0,02762266
YBR250W	90,17	1,8	0,85	2,41892782	0,01556633	0,02799596
YNL290W	1599,78	0,77	-0,37	-2,41840307	0,0155888	0,02802825
YNCK0021W	7,3	8,08	3,02	2,41816312	0,01559908	0,02803052
YPL164C	1115,62	0,75	-0,41	-2,41822869	0,01559627	0,02803052
YHR074W	3820,99	0,8	-0,33	-2,41749745	0,01562764	0,02807372
YDR440W	592,32	0,7	-0,51	-2,41597091	0,01569332	0,02818354
YPL117C	4195,42	0,79	-0,33	-2,41518503	0,01572722	0,02823627
YJR126C	2794,59	0,77	-0,37	-2,41273272	0,01583343	0,02841874
YHL043W	188,81	0,65	-0,63	-2,41198285	0,01586603	0,02846903
YHR141C	13467,71	1,26	0,34	2,4103918	0,0159354	0,02858524
YGR173W	1089,45	1,42	0,51	2,40883291	0,01600362	0,02869934
YDR174W	5022,62	0,8	-0,32	-2,40764705	0,01605569	0,02878441
YGL207W	8124,75	1,34	0,43	2,4072945	0,0160712	0,0288039
YMR312W	1004,98	0,77	-0,38	-2,40704178	0,01608233	0,02881553
YDR305C	1225,31	1,33	0,41	2,40531078	0,01615872	0,0289357
YER183C	621,85	0,73	-0,46	-2,40539876	0,01615483	0,0289357
YML047C	38,64	2,41	1,27	2,40463409	0,01618867	0,02898098
YFL010W-A	68,19	0,39	-1,38	-2,40421509	0,01620723	0,02899955
YNL302C	13592,25	1,29	0,36	2,40418932	0,01620838	0,02899955
YMR210W	1717,44	0,71	-0,48	-2,4033422	0,01624598	0,02905845
YLR155C	4,5	26,83	4,75	2,40140107	0,01633242	0,02920467
YLR086W	1722,77	1,33	0,42	2,40033077	0,01638026	0,02928178
YPL007C	962,83	0,76	-0,4	-2,40008033	0,01639147	0,02929339
YJR091C	3954,28	0,77	-0,38	-2,39582155	0,01658316	0,02962744
YBR036C	3647,52	0,79	-0,34	-2,39535156	0,01660444	0,02965692
YKL194C	842,52	0,74	-0,44	-2,39334071	0,01669573	0,0298114
YMR230W-A	13	4,76	2,25	2,39258644	0,01673009	0,02986417
YKL135C	3185,26	1,26	0,33	2,39238507	0,01673927	0,02987197
YGL111W	1950,84	1,37	0,45	2,38802151	0,01693935	0,03022035
YEL056W	2081,78	0,78	-0,35	-2,38750582	0,01696313	0,03025409
YLR099W-A	275,84	0,62	-0,69	-2,38634856	0,01701661	0,03034076
YBR198C	2992,53	0,78	-0,35	-2,38624037	0,01702162	0,03034098
YMR189W	4121,55	0,25	-2,02	-2,38355462	0,01714634	0,03055452
YLR249W	46071,68	1,45	0,53	2,38154599	0,01724014	0,03071286
YLR314C	7278,65	1,25	0,32	2,38045267	0,01729138	0,03079532
YDR176W	2610,76	1,25	0,33	2,37602699	0,01750018	0,03115825
YHR198C	1267,52	0,71	-0,49	-2,37349408	0,01762068	0,03136379
YPL167C	786,27	0,74	-0,44	-2,37265742	0,01766064	0,03142591
YBR155W	1112,07	1,41	0,5	2,3699379	0,01779107	0,03164895
YDR348C	3148,8	1,25	0,32	2,3685745	0,01785678	0,03175675

YLR229C	3247,12	0,79	-0,34	-2,36831572	0,01786928	0,03176988
YPL034W	389,1	0,71	-0,49	-2,36692289	0,01793667	0,03188057
YPR102C	11593,58	1,25	0,32	2,36557837	0,01800193	0,03198742
YMR297W	18911,32	0,74	-0,43	-2,36535054	0,01801301	0,03199795
YGR152C	1168,65	1,41	0,49	2,36313401	0,01812112	0,03218078
YBL051C	5421,81	1,33	0,41	2,36269046	0,01814282	0,03221011
YLL049W	643,15	1,35	0,43	2,36234076	0,01815994	0,0322313
YJL139C	881	0,76	-0,4	-2,36186924	0,01818305	0,03226311
YOR235W	4,82	14,03	3,81	2,3616165	0,01819545	0,03227589
YLR304C	12258,53	0,81	-0,3	-2,36088568	0,01823135	0,03233033
YDR329C	1903,11	0,77	-0,38	-2,35587189	0,01847929	0,03276066
YLR399C	5163,59	1,26	0,34	2,35420638	0,0185623	0,03289844
YGL106W	4949,52	1,24	0,31	2,35222719	0,01866137	0,03306459
YNL183C	1268,74	0,78	-0,36	-2,35204915	0,01867031	0,03307099
YPL141C	891,82	0,73	-0,45	-2,35128484	0,01870871	0,03312956
YBR262C	760,55	0,73	-0,45	-2,35101242	0,01872241	0,03314437
YMR005W	1690,92	1,27	0,34	2,34944768	0,01880129	0,03327453
YCL028W	3610,85	0,8	-0,32	-2,34820464	0,01886415	0,03337628
YJL095W	1211,32	1,31	0,39	2,34610382	0,01897082	0,0335459
YKL023C-A	383,88	0,7	-0,51	-2,34614222	0,01896887	0,0335459
YGL044C	1282,66	0,77	-0,38	-2,34585498	0,01898349	0,03355875
YMR056C	280,27	0,6	-0,74	-2,34452567	0,0190513	0,03366904
YKL080W	9902,46	1,23	0,3	2,34353629	0,0191019	0,03374887
YJL081C	2485,33	0,79	-0,34	-2,343298	0,01911411	0,03376083
YML031W	1558,14	0,78	-0,36	-2,34159983	0,01920129	0,03390518
YNR022C	1213,48	0,76	-0,39	-2,33814954	0,01937949	0,03421012
YMR107W	835,85	4,19	2,07	2,33666636	0,01945654	0,03433637
YGR251W	453,17	1,36	0,45	2,33577836	0,0195028	0,03440543
YJL090C	969,25	0,77	-0,37	-2,33570261	0,01950675	0,03440543
YDL029W	7449,9	1,27	0,34	2,33304087	0,019646	0,0346412
YPL112C	1390,74	1,27	0,34	2,33275479	0,01966102	0,03465785
YOR196C	1186,33	0,76	-0,39	-2,33259734	0,01966929	0,03466259
YHR165C	3609,1	1,26	0,34	2,33132794	0,01973607	0,03477042
YFL020C	70,33	1,97	0,98	2,33074561	0,01976678	0,03481464
YKL098W	876,98	0,73	-0,45	-2,3300377	0,01980416	0,03486071
YNL002C	3039,64	1,3	0,38	2,3300769	0,01980209	0,03486071
YGR247W	796,34	0,73	-0,45	-2,32972328	0,01982078	0,03487046
YKL006W	11620,26	0,76	-0,4	-2,32972056	0,01982093	0,03487046
YPR072W	5079,61	1,26	0,33	2,32949096	0,01983307	0,03488195
YFR047C	2845,3	0,66	-0,59	-2,32930564	0,01984288	0,03488932
YOR349W	510,52	1,35	0,44	2,32864852	0,01987769	0,03494064
YOL078W	746,6	0,76	-0,4	-2,32852017	0,0198845	0,03494272
YDR416W	1134,71	1,32	0,4	2,32811699	0,01990589	0,03497042
YJR063W	467,9	0,7	-0,5	-2,32698975	0,01996581	0,03506577
YMR134W	738,96	1,33	0,41	2,32632462	0,02000124	0,03511806
YFL062W	26,55	2,73	1,45	2,32592336	0,02002264	0,0351457
YPL208W	1095,38	0,75	-0,41	-2,32491631	0,02007644	0,03523018
YOR232W	5701,21	1,25	0,32	2,32285273	0,02018707	0,0354143
YIL006W	489,38	1,37	0,46	2,32112615	0,02028004	0,03556736
YGL108C	877	1,31	0,39	2,31808883	0,02044449	0,03584567

YJL146W	877,4	0,76	-0,4	-2,31753328	0,0204747	0,0358885
YNL178W	28374,19	1,24	0,31	2,31689882	0,02050924	0,03593891
YPL227C	1009,92	0,71	-0,49	-2,31675327	0,02051718	0,03594267
YGR196C	1988,18	1,26	0,34	2,31543348	0,02058922	0,03605871
YOR033C	1260,21	1,3	0,38	2,31505695	0,02060982	0,0360846
YPL161C	1267,67	1,32	0,4	2,31448407	0,02064118	0,03612934
YJR101W	1113,45	0,75	-0,42	-2,31386592	0,02067508	0,03617847
YGR041W	1175,42	0,77	-0,38	-2,3126616	0,02074125	0,03628405
YDL084W	12934,68	0,82	-0,29	-2,30669811	0,02107166	0,0368413
YER023W	4160,48	0,79	-0,34	-2,30679341	0,02106634	0,0368413
YJL014W	5276,41	0,73	-0,46	-2,30592669	0,02111473	0,03690622
YJL060W	2702,31	0,73	-0,46	-2,3046964	0,02118358	0,03701615
YGR048W	1025,18	0,76	-0,4	-2,3013506	0,02137182	0,03733458
YML042W	617,32	0,59	-0,77	-2,3005315	0,02141813	0,03740495
YPR097W	1592,37	0,74	-0,43	-2,30011451	0,02144173	0,03743565
YGR261C	3014,61	0,78	-0,36	-2,29803421	0,02155984	0,03763128
YDL240W	818,95	1,34	0,42	2,29381892	0,0218009	0,03804135
YBR110W	1954,97	1,26	0,33	2,29239196	0,02188304	0,03817394
YIR019C	438,43	0,71	-0,5	-2,29095714	0,02196589	0,03830773
YDR039C	6,11	8,46	3,08	2,29013795	0,02201332	0,03837967
YAR064W	56,91	0,47	-1,1	-2,29001307	0,02202056	0,03838151
YOR159C	656,78	0,73	-0,45	-2,28863735	0,02210043	0,03850992
YMR079W	2051,29	1,28	0,35	2,28577469	0,02226744	0,03879006
YNL190W	2804,75	0,72	-0,48	-2,28517588	0,02230252	0,03884027
YDR369C	715,18	0,75	-0,41	-2,28452812	0,02234051	0,03889553
YIL084C	615,84	0,76	-0,4	-2,28385734	0,02237992	0,03895322
YKL216W	5733,22	0,7	-0,51	-2,28252105	0,0224586	0,03907922
YDR137W	1963,59	0,79	-0,35	-2,28128482	0,0225316	0,03919526
YDR038C	154,55	1,55	0,63	2,2808897	0,02255497	0,03922495
YMR263W	973,48	1,29	0,37	2,27998787	0,02260841	0,03930687
YNL117W	183,42	1,58	0,66	2,27986058	0,02261596	0,03930899
YIL105C	1973,03	0,78	-0,36	-2,27815327	0,02271745	0,03947435
YNL221C	2258,33	1,29	0,37	2,27741883	0,02276123	0,03953936
YDR412W	1226,4	0,75	-0,41	-2,27449636	0,02293616	0,0398321
YPR070W	1244,09	0,79	-0,34	-2,27366196	0,02298632	0,03990805
YBR070C	478,08	0,71	-0,49	-2,27345209	0,02299895	0,03991883
YAL061W	6237,12	0,67	-0,58	-2,27173547	0,02310249	0,04008734
YEL033W	259,23	1,42	0,51	2,27124426	0,0231322	0,04012767
YHR154W	1574,34	0,74	-0,44	-2,27019479	0,02319577	0,04020657
YLR411W	240,28	1,54	0,63	2,2702696	0,02319123	0,04020657
YPR135W	1240,17	0,75	-0,42	-2,27017291	0,02319709	0,04020657
YOR155C	1967,74	0,78	-0,35	-2,26828412	0,0233119	0,04039428
YML085C	8158,42	1,23	0,3	2,2669362	0,02339412	0,04052546
YDR375C	1524,99	1,3	0,38	2,26545769	0,02348461	0,04067086
YDR427W	5563,8	1,25	0,32	2,26357579	0,02360022	0,04085969
YDR531W	1724,53	0,77	-0,37	-2,2622001	0,02368504	0,04099512
YPR144C	1324,86	1,32	0,4	2,26054899	0,0237872	0,04116047
YGL150C	3990,12	1,29	0,37	2,25887098	0,02389141	0,04132928
YDL149W	1091,44	0,78	-0,35	-2,25865384	0,02390493	0,04134115
YBL014C	759,83	1,31	0,39	2,257933	0,02394984	0,04139701

YLR248W	5794,2	0,8	-0,33	-2,25792145	0,02395056	0,04139701
YLL022C	1097,85	1,29	0,37	2,25779592	0,02395839	0,04139902
YMR212C	1371,68	0,76	-0,4	-2,25762378	0,02396912	0,04140606
YGR230W	284,95	0,67	-0,58	-2,2559711	0,02407244	0,04157298
YHR123W	1223,8	0,73	-0,46	-2,25586146	0,02407931	0,04157328
YLR034C	2181,71	0,79	-0,33	-2,25534603	0,02411162	0,0416175
YNL073W	1851,15	0,76	-0,39	-2,25447531	0,02416628	0,04170026
YIL148W	19699,17	0,82	-0,29	-2,25288401	0,02426646	0,0418615
YGL166W	1139,64	1,31	0,39	2,2524481	0,02429397	0,04189732
YHR203C	21968,38	0,8	-0,32	-2,25210328	0,02431575	0,04192323
YJL149W	1300,89	1,28	0,36	2,24945915	0,0244833	0,0422004
YGR088W	8234,94	0,65	-0,61	-2,24790051	0,02458253	0,0423597
YLR006C	1450,75	0,73	-0,45	-2,24776802	0,02459099	0,04236251
YMR104C	4069,03	1,24	0,31	2,24676784	0,02465487	0,04246078
YBL055C	1248,41	1,28	0,36	2,24643203	0,02467635	0,04248109
YML007C-A	28,58	2,76	1,47	2,24636974	0,02468034	0,04248109
YNL084C	4886,41	1,23	0,3	2,24585019	0,02471361	0,04252657
YFL017C	1021,07	0,78	-0,36	-2,24483995	0,02477841	0,04262627
YKR079C	835,46	1,34	0,43	2,24281913	0,02490848	0,04283817
YMR243C	4059,02	0,8	-0,31	-2,24098645	0,02502695	0,04303001
YNCM0002C	61,13	0,48	-1,07	-2,2399698	0,02509288	0,04313143
YGL227W	3399,34	0,8	-0,32	-2,23973503	0,02510813	0,0431457
YJL071W	559,56	0,76	-0,4	-2,23769137	0,02524119	0,04336235
YHR005C	3076,9	0,81	-0,31	-2,23533396	0,02539543	0,04361527
YNL158W	678,95	0,76	-0,39	-2,23474803	0,0254339	0,04366925
YML046W	921,51	0,77	-0,37	-2,23408853	0,02547725	0,0437316
YIL043C	6479,03	0,8	-0,31	-2,23368263	0,02550396	0,04376536
YDR214W	4898,78	1,23	0,3	2,23338245	0,02552373	0,0437872
YDR362C	1046,3	0,77	-0,37	-2,23156203	0,02564392	0,04397127
YGR089W	1495,07	0,76	-0,4	-2,2315429	0,02564519	0,04397127
YOR316C-A	34,78	2,23	1,16	2,23005759	0,02574362	0,04412786
YOR005C	1196	1,33	0,41	2,22976491	0,02576305	0,04414899
YDR320C-A	355,38	0,62	-0,69	-2,22939021	0,02578795	0,04417947
YLL032C	1077,24	0,77	-0,38	-2,22927182	0,02579582	0,04418077
YDL154W	454,73	0,74	-0,43	-2,22829467	0,02586087	0,04427997
YJL006C	476,78	1,35	0,44	2,22812954	0,02587188	0,04428661
YBR254C	414,48	0,74	-0,43	-2,22746504	0,02591621	0,04435027
YDL233W	968,48	0,77	-0,38	-2,22457339	0,02610989	0,04466941
YNCN0001W	37,72	2,34	1,23	2,22415624	0,02613793	0,04470508
YPL183W-A	488,13	0,72	-0,47	-2,22329724	0,02619576	0,04479165
YDR371W	1075,07	0,79	-0,34	-2,22084277	0,02636161	0,04506283
YGL051W	278,89	1,48	0,57	2,22011198	0,02641117	0,04513512
YDR525W-A	182,96	1,58	0,66	2,21726082	0,02660527	0,04545433
YEL006W	1182,03	0,78	-0,35	-2,21706317	0,02661878	0,0454649
YER090W	5321,53	1,31	0,39	2,21616521	0,02668019	0,04555727
YLR289W	557,99	0,76	-0,41	-2,21520436	0,02674604	0,04565716
YML094W	1269,6	0,79	-0,34	-2,21420965	0,02681436	0,04576121
YGR271W	2071,09	1,29	0,37	2,21373976	0,02684669	0,04580379
YNL018C	79,7	0,56	-0,83	-2,21256906	0,02692737	0,04592884
YKR015C	259,35	0,7	-0,51	-2,20897362	0,02717648	0,046341

YER084W	41,31	0,39	-1,34	-2,20854725	0,02720615	0,04637887
YBR153W	617,77	0,77	-0,38	-2,20677542	0,02732975	0,0465768
YLR147C	453,98	0,75	-0,42	-2,20579341	0,02739847	0,0466683
YNL177C	2138,26	0,8	-0,32	-2,20589944	0,02739104	0,0466683
YML010W	7141,6	1,32	0,4	2,20542172	0,02742452	0,04669986
YDR488C	489,88	0,74	-0,43	-2,20480915	0,02746749	0,04676022
YDL128W	2506,5	1,24	0,31	2,20457026	0,02748426	0,04677596
YJR089W	1229,13	1,29	0,36	2,2036674	0,02754774	0,04687115
YAL046C	325,73	0,68	-0,55	-2,20309881	0,02758779	0,04692643
YDR393W	1099,63	1,26	0,34	2,20254983	0,02762649	0,04697941
YDR500C	9113,6	1,27	0,35	2,2017099	0,02768581	0,04706739
YMR317W	1054,17	1,27	0,35	2,2015694	0,02769574	0,04707139
YGL068W	1898,1	0,76	-0,4	-2,19888697	0,02788596	0,04737746
YPR055W	1358,62	0,76	-0,4	-2,19881501	0,02789108	0,04737746
YDR489W	280,07	0,71	-0,5	-2,19860475	0,02790604	0,04738992
YGR229C	3655,04	0,81	-0,31	-2,19646548	0,02805864	0,04763605
YJR090C	1826,83	0,78	-0,36	-2,19591414	0,02809809	0,04768998
YJR041C	1035,47	1,37	0,45	2,19151713	0,02841439	0,04821366
YGR286C	434,49	0,74	-0,44	-2,18988504	0,02853258	0,04840097
YBL001C	1723,55	0,78	-0,36	-2,18939596	0,02856807	0,04843587
YER138C	101,31	1,71	0,77	2,18929831	0,02857516	0,04843587
YJR117W	4266,65	1,28	0,35	2,18927935	0,02857654	0,04843587
YGL249W	20,39	0,32	-1,64	-2,18844063	0,02863752	0,04852599
YLR029C	43613,42	1,22	0,29	2,18830768	0,0286472	0,04852915
YGL236C	375,43	1,39	0,48	2,18810099	0,02866225	0,04854141
YEL005C	672,82	0,72	-0,48	-2,18769919	0,02869152	0,04857774
YLR306W	679,92	0,77	-0,38	-2,18738178	0,02871467	0,04860368
YOL054W	824,49	1,3	0,37	2,18695118	0,02874609	0,04864361
YLL013C	998,23	1,27	0,35	2,18572877	0,02883545	0,04878155
YLR191W	741,64	1,32	0,4	2,18329707	0,02901394	0,04907013
YHL034C	17805,83	0,81	-0,31	-2,1823547	0,02908336	0,04917416
YMR310C	710,49	0,73	-0,45	-2,1818074	0,02912375	0,04922904
YKL037W	96,67	1,64	0,71	2,18078381	0,02919941	0,04934351
YDL121C	1458,04	0,73	-0,46	-2,18034611	0,02923182	0,04938484
YGR220C	1251,88	0,77	-0,39	-2,17904642	0,02932822	0,04953423
YDL109C	610,16	1,31	0,39	2,17825483	0,02938707	0,04959318
YGL011C	5334,63	0,8	-0,32	-2,17831638	0,02938249	0,04959318
YOR048C	2229,33	1,32	0,4	2,17837154	0,02937839	0,04959318
YMR293C	549,42	0,75	-0,41	-2,17708201	0,02947445	0,04972713
YBL009W	972,86	0,74	-0,44	-2,17623421	0,02953775	0,0498204
YEL066W	231,99	1,52	0,6	2,17530172	0,02960751	0,0499245
YKL142W	10148,88	1,25	0,32	2,17518802	0,02961603	0,04992531
YOL011W	1995,32	1,29	0,37	2,17494284	0,0296344	0,04994273
YGL156W	2183,47	0,71	-0,5	-2,17428529	0,02968372	0,05001227
YLR410W	4440,59	1,27	0,34	2,17378503	0,02972128	0,05006199
YML127W	1855,18	0,81	-0,31	-2,173479	0,02974428	0,05008715
YER116C	706,33	0,76	-0,39	-2,17290478	0,02978748	0,0501463
YDL040C	5960,51	0,8	-0,33	-2,17187621	0,029865	0,05026318
YIL177C	53,9	0,48	-1,06	-2,17071817	0,02995248	0,05039676
YDR076W	262,88	0,68	-0,56	-2,17019371	0,02999217	0,05044988

YPR067W	1351,21	1,31	0,39	2,16883729	0,03009504	0,0506092
YDL001W	958,57	1,27	0,35	2,16864368	0,03010975	0,05062023
YBL023C	810,57	0,74	-0,43	-2,16785711	0,03016956	0,05070706
YMR118C	161,22	0,65	-0,62	-2,16671629	0,03025649	0,05082567
YNL328C	132,09	0,64	-0,64	-2,16672915	0,03025551	0,05082567
YER114C	2168,06	0,79	-0,34	-2,1661571	0,03029918	0,05088363
YGL222C	926,05	0,74	-0,43	-2,16559238	0,03034235	0,05094234
YKL021C	2102,41	1,31	0,39	2,16491963	0,03039384	0,05101501
YGR110W	359,03	0,65	-0,63	-2,16361707	0,03049375	0,05116725
YNCO0012C	2,5	42,6	5,41	2,16352244	0,03050102	0,05116725
YKR063C	794,34	1,28	0,35	2,16319715	0,03052602	0,05119537
YDR034W-B	148,74	0,62	-0,68	-2,16132089	0,03067056	0,05142389
YGR006W	311,21	0,72	-0,47	-2,16013934	0,03076188	0,05156309
YHR164C	1267,81	1,27	0,34	2,15969467	0,03079631	0,05160688
YDL150W	1382,24	1,29	0,37	2,15894668	0,0308543	0,05168628
YNL313C	989,93	1,4	0,48	2,15886894	0,03086034	0,05168628
YML058W	3324,81	0,81	-0,31	-2,15873186	0,03087098	0,05169016
YNCG0020C	3,82	22,45	4,49	2,15813239	0,03091754	0,05175418
YBR049C	1817,86	0,8	-0,32	-2,1578116	0,03094248	0,05178198
YMR315W	8610,25	0,82	-0,29	-2,15740472	0,03097414	0,051821
YDR006C	2392,82	0,8	-0,33	-2,15391427	0,0312469	0,05226325
YIL003W	657,7	1,38	0,47	2,15346838	0,03128189	0,05230769
YIL109C	6611,25	0,79	-0,35	-2,15235816	0,03136916	0,05243951
YPL187W	15,35	3,32	1,73	2,15027698	0,03153331	0,05269974
YBL026W	1771,96	1,24	0,31	2,14920905	0,03161783	0,05282678
YJR152W	341,29	1,35	0,44	2,14870763	0,03165758	0,05287898
YBR103W	1334,93	1,26	0,33	2,14721505	0,03177617	0,05306278
YBL090W	745,44	1,33	0,41	2,14422223	0,03201508	0,05344738
YLR310C	3880,3	1,25	0,32	2,14334071	0,03208575	0,05355096
YDR113C	1048,7	1,26	0,33	2,14298151	0,03211458	0,05358469
YMR007W	71,52	0,57	-0,82	-2,14254153	0,03214993	0,05362926
YOL082W	3712,28	1,23	0,3	2,14075402	0,03229388	0,05385492
YNR053C	1335,08	1,31	0,39	2,14061979	0,03230471	0,05385853
YMR183C	3151,71	0,81	-0,3	-2,14034915	0,03232656	0,05388049
YLR105C	751,16	0,77	-0,38	-2,14018122	0,03234012	0,05388864
YBR048W	15928,07	0,8	-0,33	-2,13910874	0,03242686	0,05401868
YBR042C	756,3	0,75	-0,41	-2,13781167	0,03253203	0,05417935
YOR369C	26744,3	1,21	0,28	2,13759364	0,03254974	0,05419431
YCR024C-B	2623,52	1,35	0,43	2,1371524	0,0325856	0,05423948
YBR281C	1649,63	0,72	-0,47	-2,13656605	0,0326333	0,05430433
YGL105W	16588,12	0,8	-0,33	-2,13452515	0,03279982	0,05456681
YLR081W	225,35	0,7	-0,52	-2,13381917	0,03285759	0,05464828
YDR200C	1545,24	0,81	-0,31	-2,13287981	0,03293459	0,05476168
YIL012W	19,84	2,83	1,5	2,13231058	0,03298132	0,05482472
YHR002W	492,6	0,73	-0,46	-2,13113936	0,03307766	0,05497016
YHR080C	2619,78	0,78	-0,35	-2,13068945	0,03311473	0,05501705
YJR097W	108,65	1,81	0,85	2,13002037	0,03316993	0,05509401
YBL041W	4120,96	1,24	0,31	2,12855743	0,03329089	0,0552739
YCL017C	2733,85	1,24	0,31	2,12849542	0,03329603	0,0552739
YDR503C	480,8	0,75	-0,42	-2,12598917	0,03350416	0,05560456

YOL146W	442,63	0,75	-0,42	-2,12522619	0,03356774	0,0556952
YLR142W	500,33	1,44	0,53	2,12441763	0,03363524	0,05579228
YOR214C	17,15	2,96	1,56	2,12153855	0,03387651	0,05617749
YFR038W	1429,31	1,27	0,34	2,12023295	0,03398641	0,05634469
YHR026W	4336,5	0,82	-0,29	-2,11960746	0,03403916	0,0564171
YCR032W	1335,11	0,79	-0,33	-2,11703972	0,03425648	0,056747
YPL257W	252,96	0,71	-0,48	-2,11712631	0,03424913	0,056747
YIL061C	340,91	0,74	-0,44	-2,11665495	0,03428914	0,05678596
YLR462W	29,97	2,33	1,22	2,11535415	0,03439977	0,056954
YMR209C	834,75	0,79	-0,35	-2,11439707	0,03448137	0,05707388
YNL165W	421,62	0,75	-0,42	-2,11408476	0,03450803	0,05710279
YCR093W	3991,64	0,77	-0,37	-2,11167837	0,03471405	0,05741311
YOR101W	1045,65	1,26	0,33	2,11174428	0,03470839	0,05741311
YGR296W	25,07	0,39	-1,37	-2,11144035	0,03473448	0,05743162
YNL083W	751,27	1,27	0,35	2,11125698	0,03475023	0,05744237
YHR032W	1125,55	1,26	0,33	2,11018879	0,0348421	0,05757891
YER025W	6040,63	0,77	-0,37	-2,10863876	0,03497577	0,05778444
YCL039W	2556,05	1,24	0,31	2,10838965	0,0349973	0,05780463
YER093C	1326,64	0,78	-0,37	-2,1061155	0,03519432	0,0581146
YER093C-A	379,04	0,72	-0,47	-2,10586659	0,03521594	0,05813485
YDL060W	1962,01	1,36	0,44	2,10435257	0,0353477	0,05830588
YML102W	768,06	0,78	-0,35	-2,10441826	0,03534198	0,05830588
YNCF0010C	2,25	38,09	5,25	2,10435439	0,03534754	0,05830588
YLR305C	2001,9	0,78	-0,35	-2,10422807	0,03535856	0,0583083
YER021W	9026,17	1,23	0,3	2,10223619	0,03553259	0,05856957
YNCL0041C	25,35	2,9	1,54	2,10219895	0,03553585	0,05856957
YAL062W	802,51	1,41	0,49	2,09845271	0,03586517	0,05909667
YMR241W	2406,75	0,8	-0,33	-2,09735548	0,03596212	0,0592407
YJL187C	870,03	0,78	-0,37	-2,09692572	0,03600015	0,05928763
YPL028W	5620,72	0,83	-0,27	-2,09505587	0,03616603	0,05954502
YNL180C	2891,06	1,22	0,29	2,09254318	0,03638995	0,05989782
YLR056W	6244,07	0,79	-0,33	-2,09216236	0,03642399	0,05993797
YMR289W	1474,58	0,79	-0,34	-2,09072006	0,03655317	0,0601346
YNR036C	1874,14	0,76	-0,4	-2,08965516	0,03664879	0,06027595
YML065W	1641,68	1,3	0,38	2,08642936	0,03693975	0,06073841
YKL063C	1983,24	1,26	0,34	2,08620014	0,0369605	0,06075645
YGL135W	18310,87	1,23	0,3	2,08334693	0,03721961	0,0611662
YDR444W	1019,66	0,8	-0,33	-2,08190171	0,03735145	0,06136662
YJR082C	880,93	1,31	0,39	2,08010436	0,03751596	0,06162061
YGL089C	122,71	1,58	0,66	2,07948523	0,03757277	0,06169761
YOL101C	190,97	1,44	0,53	2,07905291	0,03761249	0,0617465
YMR305C	7579,99	0,78	-0,35	-2,07731239	0,03777274	0,06199319
YDR111C	1273,15	0,79	-0,34	-2,0753174	0,03795713	0,06227937
YAR029W	169,53	0,66	-0,61	-2,07340693	0,03813442	0,06253723
YDR457W	4970,09	0,81	-0,3	-2,07345047	0,03813038	0,06253723
YER130C	2298,27	0,74	-0,43	-2,07320865	0,03815287	0,06255096
YER147C	588,75	1,29	0,37	2,07265309	0,03820458	0,06261922
YLR107W	706,39	0,78	-0,36	-2,07062708	0,03839366	0,06291253
YGL140C	1900,5	0,8	-0,33	-2,06800389	0,03863966	0,06329893
YMR228W	480,57	0,76	-0,4	-2,06788653	0,03865069	0,06330032

YOL002C	747,17	0,78	-0,36	-2,06713267	0,03872165	0,06339982
YLR033W	1946,47	0,81	-0,3	-2,06669041	0,03876334	0,06345134
YNL294C	2239,49	1,22	0,29	2,06637735	0,03879286	0,06348295
YNR041C	431,61	0,75	-0,41	-2,06554799	0,03887118	0,06359436
YBR227C	1495,04	1,29	0,36	2,06455386	0,03896523	0,06373144
YKL191W	1112,12	0,78	-0,37	-2,06432497	0,03898692	0,06375012
YOL143C	3685,21	0,82	-0,28	-2,06348094	0,03906696	0,06386419
YNL156C	1899,46	0,81	-0,31	-2,06291355	0,03912084	0,06393546
YPL158C	1669,16	0,81	-0,3	-2,06245982	0,03916398	0,06398912
YGR183C	2950,09	1,4	0,48	2,06146238	0,03925895	0,06411848
YGR198W	2138,71	0,78	-0,37	-2,06141155	0,03926379	0,06411848
YLR430W	2869,93	1,28	0,36	2,06049503	0,03935124	0,06424439
YKL005C	1637,03	0,78	-0,36	-2,06012158	0,03938692	0,06428574
YGL092W	2631,14	0,75	-0,41	-2,05846799	0,03954523	0,06452718
YKL146W	2327,16	0,8	-0,33	-2,05773521	0,03961556	0,06462496
YDL236W	3174,12	1,22	0,29	2,05756127	0,03963227	0,06463525
YER026C	2886,55	0,81	-0,3	-2,05674715	0,03971055	0,06474592
YOL094C	595,98	0,75	-0,41	-2,05523603	0,03985621	0,06496636
YER110C	4419,88	1,4	0,49	2,05473267	0,03990483	0,06502855
YMR099C	5636,06	0,79	-0,34	-2,05452083	0,03992531	0,06504485
YDR472W	1102,94	0,79	-0,33	-2,05424242	0,03995224	0,06507165
YNL273W	549,23	0,74	-0,43	-2,05293943	0,04007845	0,06526012
YFR039C	1164,62	1,25	0,32	2,05219779	0,04015044	0,06536021
YEL026W	2380,23	0,72	-0,46	-2,04995403	0,04036892	0,06569864
YKL155C	1000,13	0,8	-0,33	-2,04970805	0,04039293	0,0657205
YDR438W	447,77	0,74	-0,44	-2,04877689	0,04048394	0,06585133
YNCE0026W	6,53	5,71	2,51	2,04775814	0,0405837	0,06599633
YGR116W	7229,27	1,28	0,35	2,04646085	0,04071105	0,06618609
YLR389C	3379,01	0,78	-0,35	-2,04551528	0,04080408	0,06631998
YBR231C	392,38	0,75	-0,41	-2,0450091	0,04085396	0,06638368
YLR153C	6294,2	0,79	-0,34	-2,04428397	0,0409255	0,06648254
YER150W	10449,26	0,69	-0,53	-2,04243341	0,04110856	0,06676245
YNCM0016W	13,6	0,23	-2,1	-2,04151501	0,04119966	0,06689292
YER091C	9184,93	2,44	1,29	2,04023089	0,04132733	0,06708268
YBL074C	321,36	1,4	0,49	2,03777747	0,04157219	0,0674625
YOR106W	811,93	1,27	0,35	2,03714325	0,04163569	0,0675479
YDR454C	5235,42	0,83	-0,27	-2,03691866	0,04165819	0,06756676
YNL070W	1077,51	0,8	-0,32	-2,036503	0,04169987	0,06761671
YER179W	295,03	1,41	0,5	2,03605206	0,04174513	0,06767243
YMR281W	706,03	0,78	-0,36	-2,03451824	0,04189937	0,06790475
YDR497C	5593,32	0,77	-0,37	-2,0329264	0,04205996	0,06814723
YNR065C	893,65	1,28	0,36	2,0314207	0,04221234	0,06837628
YKR098C	1740,98	0,8	-0,33	-2,03038628	0,04231729	0,06852842
YBR106W	4226,56	1,22	0,29	2,02839172	0,04252028	0,0688392
YGL241W	1318,82	0,8	-0,33	-2,02796996	0,04256331	0,06889091
YDR175C	1859,03	1,26	0,34	2,02576127	0,04278925	0,06923856
YJR004C	796,71	0,79	-0,33	-2,02506519	0,04286067	0,06933606
YEL030W	422,15	0,76	-0,39	-2,02331188	0,043041	0,06960966
YJR042W	2541,92	1,25	0,33	2,02151497	0,04322648	0,06987326
YLR242C	375,28	0,73	-0,46	-2,02153444	0,04322447	0,06987326

YFR052W	2702,12	1,23	0,29	2,01632106	0,04376641	0,07072762
YBR215W	1338,04	0,81	-0,31	-2,01219951	0,04419891	0,0713894
YDR028C	5203,47	0,83	-0,27	-2,01227648	0,0441908	0,0713894
YDL005C	2036,94	1,24	0,31	2,01207942	0,04421157	0,07139128
YDL217C	685,45	1,28	0,35	2,01118731	0,04430568	0,07152465
YCR090C	991,08	0,8	-0,32	-2,01006239	0,04442459	0,07169798
YLR312C	438,58	0,65	-0,62	-2,00914908	0,04452133	0,07183545
YMR073C	642,08	0,77	-0,37	-2,00802923	0,04464019	0,07200853
YML024W	14579,39	0,82	-0,29	-2,00755551	0,04469055	0,07207105
YGR028W	1624,9	1,3	0,37	2,00585129	0,04487212	0,07234508
YJL197W	2008,14	1,31	0,39	2,00459415	0,04500645	0,07254283
YNR031C	2007,16	1,26	0,33	2,00430771	0,04503711	0,07257342
YDR291W	1083,18	0,77	-0,37	-2,00370249	0,04510194	0,07264432
YER060W	714,5	0,77	-0,37	-2,00367862	0,0451045	0,07264432
YLR114C	2417,82	0,81	-0,3	-2,00254221	0,04522645	0,07282185
YGL027C	3914,72	1,28	0,36	1,99954803	0,04554909	0,07332235
YBL101C	1581,04	0,76	-0,4	-1,99862319	0,04564914	0,07343192
YDR252W	165,27	0,68	-0,55	-1,99859365	0,04565234	0,07343192
YER085C	15	0,31	-1,7	-1,99849619	0,04566289	0,07343192
YNCD0017W	2,15	36,72	5,2	1,99837277	0,04567626	0,07343192
YOR040W	712,68	1,31	0,39	1,99847745	0,04566492	0,07343192
YEL038W	1127,88	0,79	-0,34	-1,99664331	0,04586394	0,07371457
YLR385C	66,39	1,72	0,78	1,99639	0,04589149	0,07373976
YIL041W	5789,12	0,83	-0,28	-1,99628086	0,04590336	0,07373977
YHR124W	565,24	1,28	0,36	1,99550402	0,04598794	0,07385653
YNL118C	5100,39	1,21	0,28	1,99497581	0,04604552	0,07392218
YNL132W	2250,64	1,43	0,51	1,99491077	0,04605261	0,07392218
YDL006W	1222,61	1,24	0,31	1,9932068	0,04623881	0,07420188
YKR043C	4284,48	0,79	-0,34	-1,99270864	0,04629336	0,07427024
YOR206W	3548,77	1,32	0,4	1,99100773	0,04648004	0,07455048
YPL021W	8,29	4,74	2,24	1,99075488	0,04650784	0,07457582
YPR109W	826,9	0,76	-0,39	-1,9855486	0,04708347	0,07547936
YAL039C	1792,41	1,32	0,4	1,98494812	0,04715024	0,0755669
YGR163W	2324,64	0,82	-0,28	-1,98108126	0,04758216	0,07623946
YEL064C	331,89	0,74	-0,44	-1,97969235	0,04773811	0,0764696
YCR082W	1447,58	0,8	-0,32	-1,97822467	0,04790337	0,07671454
YNCB0009C	19,29	0,36	-1,48	-1,97778726	0,04795271	0,07677377
YAL051W	1462,64	0,81	-0,3	-1,97487556	0,04828227	0,07728149
YBR046C	2209,36	0,79	-0,34	-1,97441248	0,04833486	0,07734574
YDR120C	2167,68	1,28	0,36	1,97284047	0,04851374	0,07760166
YPL134C	577,87	0,69	-0,53	-1,9727875	0,04851978	0,07760166
YIL048W	1325,23	0,79	-0,33	-1,97188346	0,04862291	0,0777466
YNL042W	975,83	0,79	-0,34	-1,96842219	0,04901948	0,07836053
YBL024W	2475,36	0,77	-0,38	-1,9680413	0,04906329	0,07841038
YOR295W	413,59	0,76	-0,4	-1,9670088	0,0491822	0,0785802
YLR361C	645,13	0,79	-0,34	-1,96515915	0,04939582	0,07890122
YNR054C	1081,87	1,25	0,32	1,96435002	0,04948951	0,07903056
YMR082C	13,06	3,24	1,69	1,96055135	0,04993138	0,07971569
YAR008W	369,36	0,75	-0,42	-1,9588042	0,05013572	0,08002135
YOL048C	2314,76	0,76	-0,4	-1,95846478	0,0501755	0,08006427

YNL016W	8033,9	0,79	-0,33	-1,9567672	0,05037485	0,08036172
YJR112W-A	584,01	0,71	-0,5	-1,95567144	0,05050387	0,08054687
YJL127C-B	600,54	0,73	-0,45	-1,95517127	0,05056286	0,08062024
YCL061C	1349,08	0,79	-0,35	-1,95409339	0,05069017	0,0808025
YGL076C	24330,28	0,83	-0,28	-1,9529818	0,05082175	0,08097069
YKL081W	30008,14	0,78	-0,37	-1,95306235	0,05081221	0,08097069
YGR015C	532,95	0,76	-0,39	-1,95184285	0,05095686	0,08114433
YNCC0004C	20,28	0,28	-1,83	-1,95186829	0,05095384	0,08114433
YLR447C	9185,31	0,84	-0,25	-1,9504361	0,05112416	0,08138988
YPR015C	218,3	0,72	-0,47	-1,94921496	0,05126976	0,08160075
YGL004C	1562,69	1,23	0,3	1,94781054	0,05143764	0,08184698
YLR429W	8205,07	1,2	0,26	1,94665004	0,05157671	0,08204725
YJR145C	23961,38	0,8	-0,31	-1,94614806	0,05163696	0,08212206
YGR270W	5157,26	1,22	0,28	1,94580551	0,05167811	0,08216647
YNCJ0003C	46,57	2,09	1,06	1,94407324	0,05188662	0,08247689
YMR018W	105,89	1,59	0,67	1,94259542	0,05206506	0,08273936
YDR191W	196,2	1,44	0,53	1,94151314	0,05219607	0,08292634
YDR067C	1372,08	1,23	0,3	1,94088078	0,05227274	0,08302691
YBL068W	1049,06	1,27	0,34	1,93942448	0,05244967	0,08328665
YCL029C	1010,44	1,24	0,32	1,93885308	0,05251923	0,08337579
YBL022C	4211,81	0,8	-0,33	-1,93859503	0,05255067	0,08340438
YMR146C	10073,07	1,21	0,27	1,93611009	0,05285422	0,08386473
YAL024C	608,61	0,75	-0,41	-1,93183584	0,05337977	0,084677
YJL225C	139,87	0,68	-0,56	-1,92745999	0,05392233	0,08551583
YDR242W	655,99	0,79	-0,33	-1,92712421	0,05396415	0,08555859
YFR033C	7720,87	1,26	0,34	1,92691191	0,05399061	0,08555859
YPR117W	2658,08	1,2	0,27	1,92691908	0,05398972	0,08555859
YDR275W	756,53	1,27	0,34	1,92582433	0,05412631	0,08575176
YBR144C	29,78	2,35	1,23	1,9221646	0,05458505	0,08645648
YKL203C	2471,79	0,81	-0,3	-1,9211139	0,05471735	0,08664394
YKR024C	484,4	1,37	0,45	1,91929691	0,05494677	0,08698505
YHR167W	265,14	1,34	0,42	1,91776975	0,05514021	0,08726904
YCL048W-A	63,83	0,44	-1,18	-1,91747123	0,05517809	0,08730675
YJL151C	4086,69	0,76	-0,39	-1,91687927	0,05525327	0,08740344
YFR002W	2696,16	1,22	0,29	1,91347657	0,05568707	0,08806723
YMR150C	735,67	0,76	-0,39	-1,91311405	0,05573345	0,08811815
YBR019C	52,55	1,76	0,82	1,91224342	0,05584498	0,08827201
YMR069W	73,45	1,62	0,7	1,9097601	0,05616411	0,08875387
YNL015W	3811,5	0,74	-0,43	-1,90950942	0,05619641	0,08878232
YGL263W	187,8	0,71	-0,5	-1,90585458	0,05666908	0,08950631
YCR031C	23615,65	0,84	-0,26	-1,90541127	0,05672663	0,08955167
YML133C	80,65	1,59	0,67	1,90548854	0,0567166	0,08955167
YIL128W	1161,04	0,77	-0,37	-1,90452644	0,05684166	0,08971046
YLR331C	1,9	32,18	5,01	1,90432066	0,05686843	0,08972993
YLR315W	112,04	0,66	-0,6	-1,90378657	0,05693799	0,08981685
YMR226C	5843,35	1,2	0,26	1,90122222	0,05727291	0,09032225
YPL189W	97,42	1,6	0,68	1,90041874	0,05737819	0,09046531
YNL249C	458,98	0,77	-0,37	-1,89966804	0,0574767	0,09059763
YGL061C	650,89	0,8	-0,33	-1,89942143	0,05750909	0,09060521
YOL142W	811,45	0,8	-0,32	-1,89940933	0,05751068	0,09060521

YDR418W	15902,29	0,81	-0,3	-1,89780188	0,05772219	0,09091537
YDR172W	6792,51	1,24	0,31	1,89540286	0,05803905	0,09139127
YIL002C	2089,91	0,8	-0,32	-1,8935098	0,05829011	0,09176334
YDR246W	1646,15	0,81	-0,3	-1,89332151	0,05831513	0,09177947
YDR277C	3625,32	0,83	-0,26	-1,89215946	0,05846974	0,0919995
YDR188W	5575,57	1,2	0,27	1,89103925	0,05861911	0,09221116
YJL052C-A	7,1	4,5	2,17	1,88990275	0,05877097	0,09242665
YLR380W	2377,19	0,82	-0,29	-1,8894224	0,05883525	0,09250432
YPR121W	341	0,76	-0,4	-1,88918652	0,05886684	0,09253057
YJR067C	259,25	0,74	-0,44	-1,88218755	0,05981056	0,09399018
YDR116C	1169,81	0,75	-0,41	-1,88123434	0,05994005	0,09416985
Q0250	4,07	11,51	3,53	1,88067686	0,06001589	0,09426516
YOL083W	952,74	0,72	-0,47	-1,87953385	0,06017164	0,0944859
YLR420W	4834,89	1,21	0,27	1,87935036	0,06019667	0,09450132
YLR016C	556,05	0,76	-0,39	-1,87892976	0,06025408	0,09456755
YHR210C	353,66	1,41	0,5	1,87677398	0,06054908	0,0949846
YIR035C	1790,89	0,81	-0,3	-1,87665293	0,06056568	0,0949846
YJR052W	1343,17	1,23	0,3	1,87673228	0,0605548	0,0949846
YNCK0013W	51,91	1,81	0,86	1,87625428	0,06062037	0,09504638
YNL037C	6317,68	1,2	0,26	1,87581891	0,06068015	0,0951161
YGR236C	359,46	0,31	-1,69	-1,87548885	0,0607255	0,09516317
YBL092W	23715,28	1,23	0,29	1,87480315	0,06081981	0,09528692
YGL056C	1385,08	1,23	0,3	1,87280087	0,06109589	0,09569532
YGL041W-A	691,02	1,28	0,35	1,87013058	0,06146569	0,09625028
YIL004C	704,57	0,8	-0,32	-1,86970447	0,06152487	0,09631867
YMR144W	444,13	0,78	-0,36	-1,8688916	0,0616379	0,09647131
YHR027C	9836,39	1,28	0,36	1,8671227	0,06188446	0,09678405
YIR036C	1418,34	0,78	-0,37	-1,86717973	0,06187649	0,09678405
YJR118C	2070,04	1,2	0,26	1,86713765	0,06188237	0,09678405
YOR145C	1515,37	1,25	0,32	1,86619025	0,06201475	0,09696341
YLR445W	36,39	2,11	1,08	1,86296306	0,06246746	0,09764667
YLR022C	1152,33	0,8	-0,32	-1,8628442	0,06248419	0,09764825
YOL075C	617,85	0,76	-0,39	-1,86184474	0,06262498	0,09784365
YDR262W	2170,28	0,82	-0,28	-1,86131584	0,06269959	0,09791908
YNCH0007W	8,8	3,96	1,98	1,86127906	0,06270478	0,09791908
YOR008C	3575,13	1,19	0,25	1,86068205	0,06278909	0,09802609
YDL098C	449,74	1,31	0,39	1,86042343	0,06282564	0,09805852
YNL332W	22,16	2,42	1,28	1,85842579	0,06310857	0,09847537
YBR168W	631,87	0,78	-0,36	-1,8579348	0,06317827	0,09853463
YBR248C	2453,7	0,83	-0,26	-1,85804166	0,0631631	0,09853463
YDL140C	6796,44	1,27	0,34	1,85769733	0,06321201	0,0985625
YKL178C	107,66	1,53	0,62	1,85718815	0,06328439	0,0986506
YDR376W	1433,18	0,82	-0,29	-1,85406578	0,06372975	0,09931992
YCR063W	101,07	0,64	-0,64	-1,85335773	0,0638311	0,09942798
YML106W	4269,04	0,83	-0,27	-1,85342482	0,06382149	0,09942798
YDL061C	7373,69	1,19	0,25	1,85320148	0,06385349	0,09943792
YOR015W	252,64	0,74	-0,43	-1,85281931	0,06390826	0,09949828
YGL123W	38649,64	0,82	-0,29	-1,8507696	0,06420271	0,09993165
YKR022C	1044,25	1,23	0,3	1,84882378	0,06448326	0,10034319
YNL024C	174,83	1,39	0,48	1,84807425	0,0645916	0,10048661

YNCK0019W	4,8	6,48	2,7	1,8473737	0,064693	0,10061916
YOR251C	1560,44	0,83	-0,27	-1,84678528	0,06477827	0,10072656
YER170W	490,02	0,75	-0,42	-1,84540334	0,06497889	0,10101322
YKL130C	856,56	0,8	-0,33	-1,84463738	0,06509031	0,10116112
YLR409C	1837,95	1,3	0,38	1,84281979	0,06535533	0,1015476
YER073W	3239,61	0,75	-0,41	-1,84193511	0,06548464	0,10172069
YOR238W	1000,25	1,22	0,29	1,84183372	0,06549948	0,10172069
YBR211C	852,4	1,23	0,3	1,84018635	0,06574088	0,10207007
YFL019C	66,05	1,64	0,72	1,83912569	0,0658967	0,10228643
YLR288C	627,3	0,8	-0,33	-1,8380943	0,06604851	0,10249646
YLR035C-A	96,32	1,51	0,6	1,83763291	0,06611652	0,10257637
YGL125W	1418,57	1,21	0,27	1,83514869	0,06648366	0,10312022
YGL107C	1416,9	1,22	0,28	1,83480545	0,06653452	0,10317335
YDR108W	704,43	0,77	-0,37	-1,83311368	0,06678567	0,1034937
YLL038C	516,78	0,76	-0,4	-1,83307721	0,06679109	0,1034937
YNCP0007C	3	17,89	4,16	1,83309876	0,06678788	0,1034937
YJL148W	1745,74	1,33	0,41	1,83245386	0,06688383	0,10361156
YJR064W	4271,42	0,81	-0,3	-1,83122863	0,06706643	0,10386852
YOL077C	2599,29	0,77	-0,37	-1,83101287	0,06709862	0,10389248
YGL238W	3136,24	0,79	-0,35	-1,83011464	0,0672328	0,10407429
YLR026C	1317,07	0,82	-0,29	-1,82792422	0,06756093	0,10455617
YDR357C	1046,51	1,3	0,37	1,8275848	0,06761189	0,10460898
YFR019W	2160,22	0,81	-0,3	-1,82718487	0,06767198	0,10467588
YOR237W	76,49	1,6	0,68	1,82668128	0,06774771	0,10476693
YNL222W	402,11	0,76	-0,39	-1,8260277	0,06784609	0,10489296
YJL069C	874,06	1,3	0,38	1,82571945	0,06789253	0,10493864
YBR221C	8975,82	0,85	-0,23	-1,82476903	0,06803589	0,10513407
YBR009C	7974,64	0,82	-0,29	-1,8237573	0,06818877	0,10534411
YPR171W	1495,62	1,21	0,27	1,82037635	0,06870171	0,10611015
YAL048C	780,93	0,8	-0,33	-1,81935254	0,06885766	0,10632458
YAL029C	2482,31	0,8	-0,32	-1,81778061	0,06909767	0,10666867
YLR244C	5059,58	0,84	-0,25	-1,81718415	0,06918892	0,106783
YGR189C	5900,59	0,82	-0,29	-1,81601549	0,069368	0,10703278
YJL209W	876,91	1,23	0,3	1,81560894	0,06943038	0,10710244
YDR155C	33225,94	1,19	0,25	1,81260296	0,06989308	0,10777882
YNL138W	8233,64	0,85	-0,24	-1,8125351	0,06990356	0,10777882
YOR242C	98,59	0,64	-0,63	-1,8118446	0,07001021	0,10791648
YFL027C	1238,16	1,22	0,29	1,81138857	0,07008072	0,10799838
YOL062C	2098,28	1,2	0,26	1,80910663	0,07043444	0,10851655
YCL004W	698,84	0,8	-0,32	-1,80893108	0,07046171	0,10853165
YNL126W	783,33	0,81	-0,3	-1,80689405	0,0707788	0,10899304
YOR378W	263,99	1,33	0,41	1,80675882	0,07079989	0,1089985
YEL068C	432,51	0,78	-0,36	-1,80630773	0,07087028	0,10907983
YKR082W	1970,11	0,8	-0,32	-1,80505811	0,07106559	0,10935334
YLR045C	1884,81	0,84	-0,26	-1,80423465	0,07119453	0,10952462
YOR026W	803,65	1,23	0,3	1,8003406	0,07180687	0,11043929
YPL229W	463,78	0,78	-0,36	-1,79995942	0,07186705	0,11050448
YLL004W	543,39	1,26	0,34	1,79849462	0,07209866	0,11083318
YLR218C	352,06	1,29	0,37	1,79792441	0,07218899	0,11094458
YBR268W	741,62	0,77	-0,38	-1,79756486	0,07224599	0,11099088

YJL213W	728,01	1,26	0,33	1,79750902	0,07225485	0,11099088
YER188W	498,69	0,73	-0,46	-1,79699659	0,07233616	0,11108831
YNR049C	950,27	1,25	0,32	1,79606493	0,07248419	0,11128814
YJL127C	1094,25	1,22	0,28	1,79571442	0,07253995	0,11134622
YOL051W	3048,46	1,18	0,24	1,79246843	0,07305796	0,11211365
YBL095W	421,54	0,76	-0,41	-1,79028475	0,07340815	0,11262322
YFR035C	343,45	1,47	0,56	1,78872444	0,07365921	0,11298049
YAL065C	25,63	0,42	-1,24	-1,78838678	0,07371363	0,11303605
YMR129W	1712,39	0,78	-0,35	-1,78791401	0,07378988	0,11312506
YBR188C	523,77	0,79	-0,34	-1,78736843	0,07387796	0,11323215
YDL156W	367,94	0,73	-0,45	-1,78693624	0,07394779	0,11331122
YJL110C	1289,42	1,2	0,27	1,78657057	0,07400692	0,11337386
YBR233W	1021,48	1,21	0,28	1,78490146	0,0742773	0,11376001
YIL104C	405,88	1,35	0,43	1,78322103	0,07455033	0,11415003
YDR017C	2169,15	0,83	-0,27	-1,78286507	0,07460827	0,11421059
YDL174C	3278,78	0,75	-0,41	-1,78238723	0,07468611	0,11430158
YGL210W	1081,54	1,21	0,28	1,78106311	0,07490214	0,11460397
YDL214C	462,93	1,55	0,64	1,78084683	0,07493748	0,1146298
YER128W	180,99	0,72	-0,46	-1,78060558	0,07497691	0,11466188
YGR181W	1171,5	0,81	-0,3	-1,77817759	0,07537469	0,11519743
YNCH0009C	8,1	4,63	2,21	1,77813063	0,0753824	0,11519743
YNCP0002W	9,99	0,25	-1,98	-1,77812862	0,07538273	0,11519743
YHR206W	1411,55	0,79	-0,34	-1,77771489	0,07545069	0,11527292
YNCL0005C	31,09	0,5	-1,01	-1,77453208	0,07597518	0,11604569
YHR182W	739,85	0,81	-0,3	-1,7725969	0,07629553	0,11650634
YHR008C	8616,28	0,81	-0,31	-1,77115405	0,07653509	0,11684344
YMR288W	756,38	0,81	-0,3	-1,7686704	0,07694889	0,11744632
YOR071C	727,68	0,82	-0,29	-1,76842721	0,07698951	0,11747945
YML015C	875,84	1,22	0,29	1,76811477	0,07704172	0,11753025
YJR034W	503,51	0,79	-0,34	-1,76776685	0,07709989	0,11759011
YER056C-A	15567,66	0,84	-0,26	-1,76718666	0,07719697	0,11770928
YPR060C	1155,89	0,8	-0,33	-1,76647693	0,07731587	0,11786164
YGR027C	21947,92	0,85	-0,24	-1,76533341	0,07750775	0,11812515
YLL003W	1365,52	1,2	0,26	1,76456735	0,07763651	0,11829237
YDL111C	1112,53	0,8	-0,32	-1,76422522	0,07769407	0,11835104
YCL034W	2504,15	0,81	-0,31	-1,76328997	0,0778516	0,11856193
YMR196W	6951,15	0,79	-0,34	-1,76179825	0,07810339	0,11891624
YDR221W	1379,95	0,83	-0,27	-1,75928685	0,0785288	0,11953465
YLR220W	1370,92	0,83	-0,27	-1,75824274	0,07870622	0,11977536
YDR370C	704,87	0,79	-0,34	-1,7578009	0,07878139	0,1198604
YJL085W	843,66	0,81	-0,31	-1,75550151	0,07917356	0,12042756
YML111W	1571,55	0,79	-0,33	-1,75469174	0,07931205	0,12060868
YLR065C	1867,63	1,21	0,28	1,74990079	0,08013543	0,12183096
YBL037W	1819,44	1,2	0,26	1,74893065	0,08030301	0,12205586
YGL201C	1289,78	0,8	-0,32	-1,74794863	0,08047292	0,1222842
YKR046C	2690,56	0,78	-0,36	-1,74724563	0,08059474	0,12243936
YKL144C	619,72	1,26	0,33	1,7454471	0,08090707	0,1228838
YLR028C	10317,19	0,84	-0,25	-1,74489543	0,08100307	0,12299953
YIL160C	189,59	0,67	-0,57	-1,74282251	0,08136462	0,12351834
YDR453C	2177,9	1,41	0,49	1,74259381	0,08140459	0,12354882

YIL049W	480,47	0,79	-0,34	-1,7420167	0,08150552	0,12367179
YDR302W	678,31	0,81	-0,3	-1,74051319	0,08176895	0,1240412
YOR126C	543,7	0,79	-0,33	-1,73946922	0,08195226	0,12428893
YPL037C	8213,42	0,83	-0,28	-1,73799704	0,08221134	0,12465141
YIL002W-A	783,99	1,28	0,35	1,73758124	0,08228463	0,12473209
YGR014W	1720,66	1,25	0,33	1,73669281	0,08244141	0,12490879
YHR056C	1634,95	1,21	0,28	1,7367356	0,08243385	0,12490879
YMR203W	3739,34	0,85	-0,24	-1,73162711	0,08333998	0,12623944
YNR075W	485,28	1,31	0,39	1,73105257	0,08344239	0,12636375
YPL122C	873,64	0,81	-0,3	-1,72964168	0,08369431	0,12671438
YJR099W	1107,37	0,82	-0,28	-1,7285069	0,08389739	0,12699088
YJL062W-A	1046,87	0,75	-0,42	-1,72828611	0,08393694	0,1270198
YML083C	107,11	1,58	0,66	1,72592077	0,08436167	0,12763144
YOR195W	1609,46	1,29	0,37	1,72454945	0,0846087	0,12797401
YKL148C	5254,02	0,78	-0,37	-1,72161018	0,08514016	0,12874651
YCR086W	254,46	0,75	-0,42	-1,71985271	0,08545922	0,12919753
YCR044C	882,68	0,81	-0,31	-1,71968498	0,08548972	0,1292122
YNL099C	1363,15	0,84	-0,26	-1,71952888	0,08551811	0,12922367
YLR070C	351,04	0,73	-0,45	-1,71922982	0,08557253	0,12924303
YLR157C-B	305,31	1,29	0,37	1,71933582	0,08555324	0,12924303
YOL092W	1366,68	0,84	-0,26	-1,71907296	0,08560109	0,12925473
YDL159W-A	53,06	0,54	-0,9	-1,71837565	0,08572812	0,12941509
YPR111W	642,77	0,81	-0,3	-1,71700917	0,0859775	0,12976001
YCR001W	67,73	1,58	0,66	1,71513216	0,086321	0,13021517
YNCM0034C	1980,03	1,23	0,3	1,71520944	0,08630684	0,13021517
YDR465C	2363,62	1,32	0,4	1,71397715	0,08653292	0,13050316
YNL162W-A	57,35	1,73	0,79	1,71281006	0,08674749	0,130795
YCL014W	920,26	1,24	0,31	1,71258907	0,08678817	0,13082458
YKL222C	606,04	0,79	-0,33	-1,71241802	0,08681966	0,13083551
YNL182C	1042,32	1,29	0,37	1,71232092	0,08683754	0,13083551
YDR536W	160,9	0,72	-0,47	-1,71098931	0,08708309	0,13117365
YAL033W	726,41	0,82	-0,29	-1,70957654	0,08734421	0,13153508
YIL135C	2357,19	1,19	0,25	1,70941189	0,08737468	0,13154908
YNL041C	1963	1,2	0,27	1,70917726	0,08741812	0,13158259
YHR010W	16598,51	1,24	0,31	1,70798879	0,08763842	0,1318503
YMR244C-A	656,24	0,78	-0,36	-1,70801487	0,08763358	0,1318503
YDR326C	1427,91	1,2	0,26	1,7076191	0,08770704	0,13192158
YHR184W	89,18	1,49	0,58	1,70596796	0,08801405	0,13235131
YER124C	5650,68	0,86	-0,23	-1,70490227	0,08821265	0,13261786
YPL154C	18439,23	0,83	-0,26	-1,70369986	0,08843717	0,13292323
YKL004W	1781,14	0,82	-0,29	-1,70140791	0,08886642	0,13353609
YOR314W	18,57	0,38	-1,38	-1,70024056	0,08908569	0,1338332
YDR428C	551,59	1,23	0,3	1,69913613	0,08929354	0,13411302
YLR392C	916,77	0,81	-0,3	-1,69884131	0,08934909	0,13416402
YDR212W	4299,68	1,22	0,28	1,69852873	0,08940802	0,13422006
YPR159C-A	4,3	0,1	-3,29	-1,69833573	0,08944441	0,13424226
YGR240C-A	147,41	1,38	0,47	1,69640746	0,08980874	0,1347565
YLR361C-A	429,35	0,8	-0,33	-1,69627168	0,08983444	0,13476251
YJL092W	1406,4	0,82	-0,29	-1,69365496	0,09033087	0,13547449
YBR104W	540,21	1,39	0,47	1,69277073	0,09049912	0,13566132

YNL090W	1034,4	0,83	-0,27	-1,6928459	0,0904848	0,13566132
YFL025C	718,23	0,8	-0,32	-1,69253088	0,0905448	0,13566432
YPL144W	304,72	0,76	-0,4	-1,69254031	0,090543	0,13566432
YGR128C	1738,13	0,78	-0,37	-1,69198777	0,0906483	0,13578666
YDL007W	10299,18	1,2	0,27	1,69179246	0,09068555	0,1358097
YJL166W	4548,11	0,73	-0,45	-1,69015671	0,09099798	0,13624474
YER035W	3230,78	1,25	0,33	1,68656456	0,09168711	0,13721893
YGR046W	1763,16	1,2	0,26	1,68650177	0,09169919	0,13721893
YGR129W	361,24	1,28	0,36	1,68642013	0,09171491	0,13721893
YLR224W	1524,65	1,19	0,25	1,68608402	0,09177962	0,13728268
YIR024C	658	1,25	0,32	1,68572396	0,09184898	0,13735336
YLR196W	2531,56	1,28	0,35	1,68426005	0,09213142	0,13774257
YKL157W	8206,4	1,16	0,22	1,68326746	0,09232333	0,13799627
YGR150C	1176,79	0,84	-0,26	-1,68131255	0,09270222	0,13852928
YFL059W	129,56	0,68	-0,56	-1,68105844	0,09275156	0,13853636
YOR317W	9835,78	0,86	-0,21	-1,68107872	0,09274762	0,13853636
YDL112W	1547,52	1,28	0,36	1,67972204	0,09301141	0,13889108
YNCO0019W	11,31	0,32	-1,64	-1,6794681	0,09306085	0,13893151
YDL127W	671,04	1,29	0,37	1,67686829	0,09356824	0,13962188
YNCE0002W	60,93	1,74	0,8	1,67693169	0,09355584	0,13962188
YJL012C	7385,23	1,21	0,27	1,67621181	0,09369671	0,13978001
YMR025W	253,89	0,75	-0,42	-1,67563269	0,09381016	0,13991566
YPR204W	314,76	0,78	-0,36	-1,67431462	0,09406878	0,14026771
YOL111C	3405,43	0,84	-0,25	-1,67365732	0,09419797	0,14042663
YLR363W-A	288,95	1,33	0,41	1,67271219	0,09438397	0,14063641
YPR008W	3530,66	1,17	0,23	1,67273208	0,09438005	0,14063641
YLR406C	6537,77	1,2	0,26	1,67059246	0,0948022	0,14122573
YDR065W	430,83	0,79	-0,33	-1,67014966	0,09488976	0,14132227
YOL138C	1243,92	1,19	0,25	1,66706168	0,09550214	0,14220022
YPL087W	3184,39	0,83	-0,28	-1,66673758	0,0955666	0,14226209
YCR009C	6966,2	0,86	-0,22	-1,66614642	0,09568425	0,14240311
YJL068C	3043,37	0,8	-0,33	-1,66534045	0,09584485	0,14260796
YJR075W	2635,89	0,85	-0,23	-1,66512115	0,09588859	0,14263887
YJR149W	380,79	0,8	-0,33	-1,66465436	0,09598173	0,14274324
YOL128C	233,61	1,33	0,42	1,6639488	0,09612266	0,14291862
YJL045W	193,81	0,64	-0,65	-1,66221406	0,09646986	0,14340052
YIL068C	1720,13	0,84	-0,25	-1,66183898	0,09654506	0,14347797
YJR068W	2122,26	1,18	0,23	1,66025432	0,0968633	0,14391649
YNCG0028W	2,41	0,06	-4,08	-1,66002894	0,09690863	0,14394941
YOR140W	585,37	0,8	-0,32	-1,65943397	0,09702838	0,14409282
YMR153W	1589,17	0,84	-0,25	-1,65921008	0,09707347	0,14412533
YCL063W	474,48	0,74	-0,44	-1,65707132	0,09750506	0,14473152
YOL110W	395,78	0,76	-0,39	-1,65694735	0,09753012	0,14473414
YKL128C	2116,93	1,19	0,25	1,65608526	0,09770455	0,14495837
YJR113C	1322,03	0,77	-0,38	-1,65511924	0,09790031	0,14521412
YLR115W	1297,95	0,83	-0,27	-1,65114458	0,09870906	0,14637877
YBR286W	33654,08	1,16	0,21	1,65062896	0,09881436	0,14649996
YJR069C	903,27	0,79	-0,33	-1,64997828	0,09894738	0,14666216
YBR021W	357,95	1,27	0,34	1,64861971	0,09922557	0,14703942
YPL216W	678,06	0,82	-0,28	-1,64735124	0,09948587	0,14738999

YNCD0032C	162,01	1,36	0,44	1,64713614	0,09953007	0,14742031
YDL212W	2299,65	0,85	-0,23	-1,64663489	0,09963312	0,14753776
YMR135C	2540,29	0,85	-0,23	-1,64504524	0,09996048	0,14798725
YOL155C	11100,4	1,21	0,27	1,64131027	0,10073303	0,14909544
YDL064W	1898,17	1,18	0,23	1,64045376	0,10091086	0,14925196
YGL071W	1737,86	1,21	0,28	1,64056395	0,10088796	0,14925196
YLR381W	651,88	0,82	-0,29	-1,64057107	0,10088648	0,14925196
YHR042W	6772,08	0,83	-0,27	-1,640021	0,1010008	0,14934943
YLR335W	5612,67	1,19	0,25	1,63982649	0,10104125	0,14937369
YMR041C	728,46	1,26	0,34	1,63881749	0,10125128	0,14964857
YCR002C	3098,79	0,85	-0,24	-1,63798651	0,10142451	0,14986895
YDR477W	3602,66	0,85	-0,23	-1,63746239	0,10153389	0,14997317
YHR076W	1978,62	0,83	-0,27	-1,63741718	0,10154333	0,14997317
YER151C	3708,48	1,19	0,25	1,63599928	0,10183974	0,1503752
YPL062W	15,26	2,52	1,33	1,63383538	0,10229342	0,1510092
YMR238W	1671,03	0,82	-0,28	-1,63287015	0,10249631	0,15127277
YPL002C	838,68	0,82	-0,29	-1,63213774	0,10265047	0,15146431
YEL055C	3020,11	1,27	0,34	1,63149366	0,1027862	0,15162856
YLL014W	1012,65	0,81	-0,3	-1,63012996	0,10307403	0,15201707
YPR165W	9247,11	0,87	-0,21	-1,62958119	0,10319004	0,15215204
YMR084W	385,75	1,25	0,32	1,62934202	0,10324063	0,15219051
YGR206W	369,14	1,25	0,32	1,62884197	0,10334648	0,15231039
YKL024C	1839,74	0,81	-0,31	-1,62809497	0,10350475	0,15250747
YHR086W-A	11,91	2,68	1,42	1,62663511	0,10381462	0,15292777
YDL130W	10614,36	0,85	-0,23	-1,62377438	0,10442397	0,15375248
YLR228C	1737,3	0,84	-0,24	-1,62379058	0,10442051	0,15375248
YNCE0024W	909,85	2,26	1,17	1,62144076	0,10492315	0,15445085
YPL057C	2820,01	1,17	0,22	1,62093453	0,10503168	0,15457399
YLR358C	13,15	0,3	-1,73	-1,61767985	0,10573161	0,15556721
YDR086C	3444,47	0,86	-0,23	-1,6174239	0,10578681	0,15561157
YLR256W	6112,78	0,86	-0,22	-1,6172276	0,10582916	0,15563701
YPL257W-B	51,33	1,65	0,72	1,61624443	0,10604148	0,15591234
YGL151W	2738,79	0,84	-0,25	-1,61521482	0,10626418	0,15620282
YAL058W	758,68	0,83	-0,27	-1,6140838	0,10650925	0,15652602
YGL095C	3319,17	1,17	0,22	1,6137356	0,10658479	0,15659998
YJR008W	2232,7	0,8	-0,33	-1,60995705	0,10740723	0,15777104
YAR066W	38,11	0,57	-0,82	-1,60971165	0,10746082	0,15781244
YOR304W	2274,81	0,84	-0,25	-1,60331868	0,10886432	0,15983577
YNR011C	1057,68	0,83	-0,26	-1,60301206	0,10893199	0,15989734
YHR218W	3,98	0,11	-3,16	-1,60213174	0,10912648	0,16010716
YMR309C	2801,63	1,3	0,38	1,60222797	0,10910521	0,16010716
YHR192W	958,78	1,2	0,27	1,60080691	0,10941969	0,16049944
YER186C	1271,89	1,19	0,25	1,59953011	0,10970286	0,16087681
YGL170C	30,91	1,98	0,99	1,59864591	0,1098993	0,16112685
YMR299C	1049,44	1,19	0,25	1,59843457	0,1099463	0,16115771
YHR118C	623,84	1,23	0,3	1,59732762	0,1101927	0,16148078
YDR456W	2135,99	0,86	-0,22	-1,59693175	0,11028092	0,16157195
YLR401C	1405,97	1,23	0,3	1,59677956	0,11031485	0,16158356
YHR059W	505,72	1,26	0,33	1,59605711	0,11047604	0,16176016
YPL226W	3599,8	1,31	0,39	1,59600578	0,1104875	0,16176016

YLR129W	1657,86	1,25	0,32	1,59509941	0,11069001	0,16198029
YPR178W	881,82	0,83	-0,26	-1,59516817	0,11067463	0,16198029
YML067C	1915,75	0,85	-0,23	-1,59419872	0,11089153	0,16223698
YJR136C	561,78	1,23	0,3	1,59407153	0,11092002	0,16224044
YCL001W-A	47,66	0,57	-0,82	-1,59332446	0,11108742	0,16244705
YKL095W	555,75	1,25	0,32	1,59013856	0,11180358	0,16345583
YNCP0024C	1,47	24,94	4,64	1,58973449	0,11189467	0,16355051
YGR275W	1239,43	0,83	-0,26	-1,58873301	0,11212068	0,16384231
YGR239C	499,53	1,29	0,36	1,58856219	0,11215927	0,16386016
YOR021C	2145,99	1,19	0,25	1,58813094	0,11225673	0,16396399
YKL193C	2823,45	1,21	0,27	1,58769055	0,11235633	0,16407088
YDR432W	11351,98	1,19	0,25	1,58750935	0,11239733	0,16409218
YBR171W	1836,07	0,84	-0,25	-1,58641333	0,11264558	0,16441596
YER165W	16598,42	0,81	-0,3	-1,58506487	0,1129516	0,16482389
YHR132W-A	1211,98	0,83	-0,28	-1,58335884	0,1133397	0,16535139
YER101C	911	0,82	-0,29	-1,58311117	0,11339613	0,16539487
YNL137C	2211,55	0,83	-0,26	-1,58026864	0,11404536	0,16630276
YMR090W	2006,46	1,36	0,45	1,57578674	0,11507496	0,16776476
YBL105C	3802,68	0,86	-0,21	-1,57547452	0,11514696	0,16781969
YKL106C-A	4,55	6,06	2,6	1,57527196	0,11519368	0,16781969
YNL146W	186,88	1,36	0,44	1,57528204	0,11519136	0,16781969
YGR168C	677,33	1,21	0,27	1,57411981	0,11545975	0,16816787
YHL024W	792,67	0,81	-0,3	-1,57277423	0,11577111	0,16858183
YDL165W	1151,94	1,2	0,26	1,5719216	0,11596873	0,16879048
YKR086W	986,58	0,83	-0,26	-1,57201289	0,11594756	0,16879048
YPR176C	995,15	1,2	0,27	1,56981437	0,1164583	0,16946334
YLR197W	9809,06	1,23	0,3	1,5694208	0,11654992	0,16955693
YNL130C	1976,09	0,85	-0,24	-1,56775407	0,11693854	0,17008246
YDR140W	605,01	0,81	-0,3	-1,56652913	0,1172248	0,1704589
YGR039W	75,99	1,49	0,58	1,56616521	0,11730995	0,1705428
YNL119W	654,73	1,23	0,3	1,56471495	0,11764976	0,1709968
YLR423C	653,25	0,8	-0,32	-1,56412928	0,11778721	0,17115653
YLR104W	550,4	1,24	0,31	1,56291878	0,1180717	0,17152979
YLR464W	2,09	0,07	-3,88	-1,56250202	0,11816977	0,17163213
YGR066C	224,49	0,73	-0,46	-1,56051722	0,1186377	0,17227149
YJL203W	666,3	1,22	0,28	1,55931637	0,11892152	0,17264325
YBR175W	422,84	0,8	-0,33	-1,55891085	0,11901748	0,1727422
YNCO0003C	2,41	0,06	-4,08	-1,55839882	0,11913874	0,17287779
YBR156C	681,33	0,83	-0,27	-1,55739952	0,11937566	0,17318113
YHR142W	1454,1	0,84	-0,25	-1,55672001	0,11953698	0,17337467
YLR274W	1607,45	0,84	-0,25	-1,5542753	0,12011876	0,17417781
YOL053W	1793,09	1,2	0,27	1,55325423	0,12036241	0,17449038
YOR019W	1311,02	1,34	0,42	1,5528548	0,12045783	0,17458797
YOR094W	465,61	0,82	-0,29	-1,55272576	0,12048866	0,17459193
YCL042W	79,52	1,48	0,57	1,55120598	0,12085233	0,17507805
YBL079W	2670,81	0,85	-0,23	-1,55093873	0,12091637	0,17512998
YDL083C	10647,23	1,15	0,2	1,5500824	0,12112174	0,17538654
YNL071W	11232,23	0,87	-0,2	-1,54922701	0,12132716	0,17563203
YOR249C	1031,12	0,83	-0,26	-1,54914104	0,12134782	0,17563203
YIL054W	13,43	2,45	1,29	1,54749403	0,12174416	0,17616463

YMR054W	2781,16	0,84	-0,25	-1,54626201	0,1220413	0,17655346
YDL097C	7746,38	1,17	0,22	1,54584518	0,12214196	0,17665794
YOR115C	1183,73	1,19	0,25	1,54484094	0,12238474	0,17696788
YER015W	610,23	0,79	-0,34	-1,54376532	0,12264519	0,17730322
YDL135C	2404,85	0,86	-0,23	-1,54309848	0,12280688	0,17749566
YOR192C	198,21	0,77	-0,38	-1,54279013	0,12288171	0,1775212
YPL065W	982,02	1,19	0,25	1,54284883	0,12286746	0,1775212
YDR130C	754,33	1,21	0,28	1,54174024	0,12313673	0,17784826
YBL049W	254,62	0,76	-0,4	-1,5394191	0,12370201	0,17862318
YGR105W	1051,76	1,18	0,24	1,53796891	0,12405622	0,17906117
YMR242W-A	12,29	2,47	1,31	1,53794123	0,12406299	0,17906117
YDR267C	1158,07	1,18	0,23	1,53535383	0,12469694	0,17993435
YBR272C	1329,68	1,17	0,23	1,53513468	0,12475075	0,17997019
YLR325C	12644,29	1,19	0,24	1,5334529	0,1251643	0,18052488
YMR097C	1295,42	1,19	0,25	1,53326535	0,12521049	0,18054957
YKL125W	659,36	1,2	0,26	1,53267371	0,12535627	0,18071783
YPR010C	5304,63	1,28	0,36	1,53219943	0,12547323	0,18084448
YHL003C	2613,79	0,86	-0,21	-1,53058097	0,12587299	0,18137857
YHR158C	2808,1	1,18	0,24	1,53017595	0,12597318	0,18148085
YPL038W-A	5,21	4,4	2,14	1,52919044	0,12621724	0,18179029
YPL118W	3727,99	0,85	-0,23	-1,528868	0,12629717	0,18186324
YER142C	637,45	1,2	0,27	1,52672297	0,12682991	0,18258805
YGL075C	357,61	1,25	0,32	1,52598597	0,12701336	0,18280977
YGR158C	741,57	1,23	0,29	1,52556209	0,12711896	0,18291938
YJR076C	3095,45	0,86	-0,22	-1,52540724	0,12715755	0,18293254
YGL186C	1819,47	0,83	-0,27	-1,52514747	0,12722232	0,18297849
YML037C	200,25	1,3	0,38	1,52504289	0,1272484	0,18297849
YLR412W	1202,56	1,18	0,23	1,52454896	0,12737164	0,18311331
YIL146C	735,12	1,21	0,28	1,52422955	0,12745138	0,18316921
YLR342W-A	88,89	0,69	-0,54	-1,524157	0,1274695	0,18316921
YDL010W	756,93	0,79	-0,33	-1,52250662	0,12788219	0,1837028
YOR059C	1600,42	0,84	-0,24	-1,52243559	0,12789997	0,1837028
YOR105W	18,55	2,1	1,07	1,52179576	0,12806026	0,18389051
YKL106W	503,89	1,35	0,44	1,52155144	0,12812151	0,18393595
YLR453C	268,82	0,78	-0,35	-1,52124546	0,12819825	0,18400359
YPL243W	2993,1	0,85	-0,23	-1,51883823	0,12880322	0,1848292
YNL301C	3802,44	0,86	-0,23	-1,51844669	0,12890183	0,18492798
YKL053C-A	763,35	1,25	0,32	1,51716583	0,12922482	0,18530577
YLR439W	1184,93	0,81	-0,3	-1,51721584	0,1292122	0,18530577
YHR150W	647,24	0,83	-0,26	-1,51613268	0,12948581	0,18563717
YOL112W	1281,3	0,85	-0,23	-1,515432	0,12966304	0,18584836
YKL165C	916,81	0,77	-0,38	-1,51479565	0,12982416	0,18603638
YER051W	907,43	1,19	0,25	1,5137917	0,13007868	0,18635811
YMR124W	2582,87	0,85	-0,23	-1,51333845	0,13019371	0,1864799
YDR057W	1082,19	0,84	-0,25	-1,51273357	0,13034735	0,1865845
YOR244W	1055,84	1,19	0,25	1,51269618	0,13035685	0,1865845
YPR157W	599,17	1,32	0,4	1,512919	0,13030023	0,1865845
YNL333W	138,4	0,7	-0,51	-1,51168902	0,13061299	0,18690807
YHR137W	2038,25	0,72	-0,48	-1,51097567	0,13079465	0,18712491
YIL056W	1893,47	1,21	0,27	1,50909217	0,13127523	0,18776922

YNCD0008W	2,25	12,81	3,68	1,50893755	0,13131474	0,1877825
YER056C	4133,05	0,84	-0,24	-1,5080177	0,13155	0,18805786
YOR122C	9064,75	0,82	-0,28	-1,50794797	0,13156784	0,18805786
YGR084C	1351,64	0,84	-0,26	-1,50602474	0,13206082	0,18871908
YPL082C	1403,56	1,19	0,25	1,50500128	0,13232375	0,18905132
YLR162W	21,97	2,24	1,16	1,5041868	0,13253328	0,18930713
YIL166C	494,28	0,8	-0,33	-1,50285219	0,13287716	0,18971109
YLR426W	663,15	1,2	0,27	1,50296579	0,13284786	0,18971109
YCR079W	1137,68	0,82	-0,29	-1,50257199	0,13294945	0,18977068
YML023C	964,83	0,84	-0,24	-1,50223801	0,13303565	0,18984343
YOR275C	2045,14	0,86	-0,21	-1,50213775	0,13306154	0,18984343
YLR406C-A	9,25	2,84	1,51	1,50117715	0,13330975	0,19015388
YJR073C	2612,15	0,82	-0,28	-1,4995055	0,13374254	0,19067761
YOR095C	1279,72	1,26	0,34	1,49940345	0,133769	0,19067761
YPL071C	840,51	1,18	0,24	1,49958396	0,13372221	0,19067761
YLR061W	12014,78	0,86	-0,21	-1,49839487	0,13403069	0,19096299
YLR287C-A	18133,25	1,15	0,2	1,49850227	0,1340028	0,19096299
YGR091W	562,73	0,83	-0,28	-1,49717608	0,13434745	0,19137041
YDR390C	2901,46	0,87	-0,21	-1,49508039	0,13489346	0,19210413
YKR064W	1151,98	1,18	0,23	1,49471557	0,13498869	0,19215163
YNCM0003W	2,56	15,16	3,92	1,49482692	0,13495962	0,19215163
YMR055C	273,66	0,79	-0,34	-1,49370059	0,13525389	0,19248504
YMR088C	1004,77	0,84	-0,25	-1,49339607	0,13533354	0,19255427
YIR027C	129,5	0,69	-0,54	-1,49203735	0,13568935	0,19301632
YDR341C	7132,64	0,84	-0,25	-1,49180144	0,13575121	0,19306009
YJL010C	1423,25	1,28	0,35	1,49082829	0,13600658	0,19329053
YLR355C	8587,87	1,3	0,38	1,49086225	0,13599766	0,19329053
YPR149W	21772,51	0,85	-0,24	-1,49104661	0,13594926	0,19329053
YKR020W	684,74	0,82	-0,28	-1,49055987	0,13607709	0,19334649
YML050W	401,9	0,79	-0,34	-1,48734943	0,13692255	0,19450328
YPL048W	13823,23	0,86	-0,21	-1,48651994	0,13714165	0,19476998
YGR093W	817,32	1,19	0,25	1,48574084	0,13734768	0,19499134
YKR083C	479,12	1,22	0,29	1,48569322	0,13736028	0,19499134
YNR076W	42,95	1,61	0,69	1,48456459	0,1376592	0,19537102
YDL138W	1049,93	1,19	0,25	1,48388594	0,13783918	0,19556761
YOR188W	1127,39	0,84	-0,26	-1,48380493	0,13786068	0,19556761
YJR066W	2075,62	0,86	-0,21	-1,4823944	0,13823538	0,19605438
YGL028C	2695,43	0,86	-0,23	-1,48202783	0,13833289	0,1961446
YML082W	618,7	1,24	0,31	1,48191789	0,13836214	0,1961446
YBL042C	847,22	0,84	-0,25	-1,48088357	0,1386376	0,19649025
YOL070C	2652,36	1,15	0,2	1,47727326	0,1396024	0,19781253
YER044C	1819,08	0,83	-0,27	-1,4763233	0,13985712	0,19812826
YML005W	545,28	1,2	0,26	1,47616793	0,13989882	0,19814215
YNCO0020C	3,92	5,15	2,37	1,47515538	0,14017077	0,19848207
YKL019W	1723,44	0,85	-0,23	-1,47247664	0,14089221	0,19945816
YDL247W	24,98	1,88	0,91	1,47070581	0,1413707	0,20008994
YNCG0037W	14,05	2,37	1,24	1,47054409	0,14141445	0,20010628
YNL049C	1848,19	0,82	-0,29	-1,47041152	0,14145033	0,20011147
YDL166C	655,19	1,19	0,26	1,46911725	0,14180099	0,20056056
YDR434W	1919,29	1,16	0,21	1,46900164	0,14183235	0,20056056

YAL034W-A	381,63	0,81	-0,3	-1,46767869	0,14219152	0,2010227
YHR213W-B	27,24	0,54	-0,89	-1,4663803	0,14254471	0,20147616
YHR101C	724,7	1,19	0,25	1,46542685	0,1428045	0,20179744
YLR098C	863,73	0,84	-0,24	-1,46487741	0,14295437	0,20196328
YAR068W	32,18	0,57	-0,81	-1,46334654	0,14337258	0,20250806
YJR153W	312,74	0,8	-0,31	-1,4620108	0,14373825	0,20297841
YGR076C	806,81	1,21	0,27	1,46101391	0,14401162	0,20331823
YOR152C	782,03	1,21	0,28	1,46000018	0,14429002	0,20366499
YBR301W	46,43	1,59	0,67	1,4589242	0,14458597	0,20403636
YNL218W	361,73	0,82	-0,29	-1,45845106	0,14471626	0,20417383
YOR271C	2152,66	0,82	-0,29	-1,45764781	0,14493765	0,20443975
YNL035C	1010,12	0,85	-0,23	-1,45700484	0,14511504	0,20464351
YJL028W	6,48	3,47	1,79	1,45596807	0,14540145	0,20500086
YEL015W	2803,14	1,15	0,2	1,45381912	0,14599646	0,20574638
YJR119C	388,91	1,26	0,33	1,45385659	0,14598607	0,20574638
YBR089C-A	317,4	1,39	0,48	1,45218996	0,14644879	0,20633702
YJR054W	500,83	0,78	-0,35	-1,45039994	0,14694702	0,20699204
YAL064W-B	36,98	0,59	-0,75	-1,45020875	0,14700032	0,20702017
YBR127C	15150,71	0,88	-0,18	-1,44981114	0,14711119	0,20712486
YLR092W	717,57	0,83	-0,26	-1,44970308	0,14714134	0,20712486
YCR054C	450,8	0,81	-0,3	-1,44762824	0,14772105	0,20789378
YNL005C	2565,79	0,86	-0,22	-1,44747443	0,14776409	0,20790726
YPR093C	476,28	0,83	-0,27	-1,44700972	0,1478942	0,2080432
YCR051W	1731,6	1,17	0,23	1,4465758	0,14801577	0,20811995
YEL041W	501,56	0,8	-0,33	-1,44658348	0,14801361	0,20811995
YPR058W	1048,69	0,86	-0,22	-1,44595346	0,14819025	0,20831814
YKL102C	20,65	1,97	0,98	1,44582805	0,14822544	0,20832046
YGL013C	1656,14	0,83	-0,27	-1,44483652	0,14850381	0,20866448
YDR213W	1218,21	1,2	0,26	1,44469782	0,14854278	0,20867204
YMR184W	623,72	0,83	-0,27	-1,44410876	0,14870839	0,20885745
YPL261C	3,32	5,98	2,58	1,44380135	0,14879486	0,20893167
YOL081W	6858,79	1,15	0,2	1,4432061	0,14896242	0,20911968
YDR374W-A	251,77	1,29	0,37	1,44249997	0,14916138	0,20933374
YLR459W	741,9	0,84	-0,25	-1,44242575	0,14918231	0,20933374
YOR189W	659,99	0,84	-0,25	-1,44203629	0,14929214	0,20944055
YAL055W	678,81	1,22	0,29	1,44044959	0,14974024	0,21002176
YMR175W-A	12,71	2,29	1,19	1,44021491	0,14980661	0,21006741
YDR210C-D	386,11	0,8	-0,32	-1,44007903	0,14984504	0,21007389
YML104C	965,02	0,84	-0,25	-1,43976803	0,14993304	0,21014983
YPR071W	369,67	1,22	0,28	1,43954698	0,14999561	0,2101901
YBR199W	6564,73	1,14	0,19	1,43937723	0,15004367	0,21021003
YLR281C	137,37	0,69	-0,54	-1,43672725	0,15079551	0,21121571
YDR524W-C	5261,6	0,85	-0,23	-1,43609456	0,15097544	0,21142007
YNCP0008C	5,89	0,25	-1,98	-1,43476355	0,15135449	0,21190311
YAR035W	225,74	1,28	0,35	1,4346221	0,15139481	0,2119118
YKR035W-A	1413,63	0,85	-0,24	-1,43417922	0,15152113	0,21204083
YOR324C	793,91	0,84	-0,24	-1,43390551	0,15159923	0,21210234
YOR030W	1252,81	0,85	-0,24	-1,43124281	0,15236064	0,21311963
YGR177C	373,05	0,81	-0,31	-1,43099266	0,15243232	0,21317189
YJR077C	2818,34	0,84	-0,26	-1,43033625	0,15262053	0,21338707

YFR025C	1214,33	0,85	-0,23	-1,42907652	0,15298224	0,21384466
YMR275C	1704,63	0,85	-0,24	-1,4285499	0,15313364	0,21400814
YOR281C	1910,78	1,15	0,2	1,42722675	0,15351455	0,21449221
YML014W	790,45	1,22	0,29	1,42605818	0,15385155	0,21491473
YDR373W	1362,7	0,85	-0,24	-1,42583207	0,15391682	0,21495758
YDR016C	407,8	0,77	-0,38	-1,42567355	0,1539626	0,21497317
YDL133W	905,66	0,85	-0,23	-1,42489046	0,15418887	0,21524073
YDR316W-B	66,97	1,51	0,59	1,42472483	0,15423676	0,21525921
YLR442C	1088,42	0,84	-0,25	-1,42277985	0,15479998	0,21599674
YHR219W	44,44	0,63	-0,68	-1,42109318	0,15528967	0,21663136
YAL014C	1078,74	0,84	-0,24	-1,42076907	0,1553839	0,21671415
YLR266C	1329,45	1,16	0,22	1,42030991	0,15551748	0,21685176
YIL072W	247,27	0,77	-0,37	-1,41948648	0,15575724	0,21713733
YMR093W	713,7	0,81	-0,3	-1,4190356	0,15588864	0,21727175
YDR519W	1152,53	0,85	-0,23	-1,41670864	0,15656813	0,21816986
YGL257C	1842,44	1,15	0,21	1,41318782	0,15760051	0,21955917
YGL118C	4,96	4,11	2,04	1,41244244	0,15781973	0,21981528
YOR254C	4948,2	0,83	-0,27	-1,41090919	0,15827139	0,22039495
YJR012C	546,24	1,22	0,28	1,41049042	0,15839492	0,22051754
YKR072C	3509,67	1,16	0,21	1,40974381	0,15861535	0,22077493
YDR157W	17,48	2,02	1,02	1,40910842	0,15880311	0,22098677
YNCO0023W	1,97	11,36	3,51	1,40794373	0,15914773	0,22141674
YDL086W	2947,69	0,84	-0,26	-1,40653268	0,15956601	0,22194896
YBR209W	60,09	1,52	0,6	1,40312807	0,16057865	0,22325752
YNCN0003W	9,67	2,53	1,34	1,40314572	0,16057339	0,22325752
YGL073W	3384,72	0,88	-0,19	-1,40119078	0,16115703	0,2238802
YLR085C	1104,8	0,86	-0,22	-1,40114512	0,16117068	0,2238802
YLR222C	1254,9	1,22	0,29	1,40140014	0,16109445	0,2238802
YOR316C	2469,2	1,15	0,2	1,40134148	0,16111198	0,2238802
YDR010C	30,95	0,55	-0,85	-1,40047031	0,16137253	0,22411047
YBR208C	3163,13	1,14	0,19	1,39913917	0,16177125	0,224614
YDL129W	497,76	0,83	-0,27	-1,39781689	0,16216806	0,22511464
YDR181C	752,3	0,84	-0,24	-1,39494501	0,16303242	0,22626394
YMR137C	802,29	1,17	0,23	1,39469581	0,16310759	0,2263177
YPR183W	6431,41	1,16	0,21	1,39430117	0,16322668	0,22643236
YAL019W	1573,06	1,26	0,33	1,39376716	0,16338793	0,22660545
YOR319W	482,32	1,2	0,26	1,39335999	0,16351096	0,22672546
YDR041W	1713,69	0,82	-0,28	-1,39304163	0,1636072	0,22680567
YOR222W	1916,74	1,15	0,2	1,39292713	0,16364183	0,22680567
YPL155C	841,41	1,17	0,23	1,3910618	0,16420669	0,2275378
Q0275	1,22	20,38	4,35	1,39040761	0,16440514	0,2276097
YMR075C-A	1,22	20,38	4,35	1,39040761	0,16440514	0,2276097
YNCO0013C	1,22	20,38	4,35	1,39040761	0,16440514	0,2276097
YPL147W	746,64	0,84	-0,25	-1,39066166	0,16432805	0,2276097
YER038C	437,22	1,2	0,26	1,39001389	0,16452466	0,22772442
YML113W	401,19	0,82	-0,29	-1,38937168	0,16471976	0,22794367
YNL329C	1122,14	0,85	-0,23	-1,38865838	0,16493666	0,22819299
YNR019W	2924,22	1,19	0,25	1,38820223	0,16507547	0,22833419
YDR124W	352,04	0,82	-0,29	-1,38665581	0,16554674	0,22893507
YDR461W	7347,88	1,3	0,38	1,38643355	0,16561456	0,22897789

YLR010C	210,45	1,29	0,36	1,38475526	0,16612731	0,22963571
YOL159C	472,45	0,83	-0,27	-1,38453961	0,16619329	0,2296758
YLR227C	467,75	1,22	0,29	1,38405759	0,16634082	0,22982856
YGR289C	1021	0,79	-0,35	-1,38389965	0,16638918	0,22984426
YML132W	174,98	0,68	-0,55	-1,38267073	0,16676585	0,23031335
YLR007W	629,06	0,83	-0,27	-1,38241056	0,16684568	0,23037238
YOR343C	110,32	0,68	-0,56	-1,38215165	0,16692514	0,23037969
YPL085W	2969,8	0,85	-0,24	-1,38224272	0,16689719	0,23037969
YBR228W	509,29	1,19	0,25	1,38200529	0,16697008	0,23039052
YNL311C	665,16	0,85	-0,24	-1,38106291	0,16725962	0,23073879
YDR245W	2463,04	1,15	0,21	1,38077868	0,16734702	0,2308081
YBR173C	3460,85	1,14	0,19	1,38014619	0,16754164	0,23102523
YER048C	4901,96	1,13	0,18	1,37912173	0,16785723	0,23140903
YER181C	5,79	0,24	-2,03	-1,3781565	0,16815498	0,23171666
YHR051W	1880,22	0,86	-0,22	-1,37815926	0,16815412	0,23171666
YPL263C	2347,37	1,21	0,27	1,37798202	0,16820884	0,23173948
YBR162W-A	944,33	0,83	-0,26	-1,37785388	0,16824841	0,23174259
YIL140W	949,9	0,8	-0,32	-1,37682771	0,16856552	0,23212792
YGR203W	421,25	0,8	-0,32	-1,37663728	0,16862442	0,23215756
YOL097C	5827,33	1,17	0,23	1,37616315	0,16877113	0,23230806
YLR323C	349,15	0,82	-0,29	-1,37585129	0,16886768	0,23238946
YHL009C	982,82	0,86	-0,22	-1,37477151	0,16920229	0,23279837
YAL013W	2133,34	1,18	0,24	1,37423766	0,16936791	0,23297464
YER167W	1905,05	1,2	0,27	1,3738814	0,1694785	0,23307515
YHL041W	8,31	2,7	1,43	1,37032294	0,17058612	0,23454647
YLL048C	4242,02	0,85	-0,24	-1,36879856	0,17106225	0,23514416
YPR164W	1072,09	0,85	-0,24	-1,36868895	0,17109652	0,23514416
YPL207W	2723,7	1,18	0,24	1,36537909	0,17213393	0,23651758
YML096W	686,04	0,84	-0,26	-1,36428706	0,17247724	0,23693689
YFL060C	70,05	0,7	-0,52	-1,36252069	0,17303362	0,23764865
YIR034C	1124,71	1,16	0,22	1,36142322	0,17337998	0,23807172
YCR059C	1062,74	0,86	-0,22	-1,35961284	0,17395247	0,23880503
YGR169C	913,41	0,85	-0,23	-1,35780593	0,17452527	0,23953844
YPR141C	738,34	0,85	-0,23	-1,35667861	0,17488335	0,23997689
YCL005W	696,8	0,83	-0,27	-1,35274427	0,17613734	0,24164425
YNCN0006W	4,08	4,12	2,04	1,34859596	0,17746678	0,24341436
YKL017C	986,55	0,85	-0,23	-1,34817386	0,17760247	0,2435467
YLR125W	129,48	1,31	0,39	1,34576031	0,17837983	0,24455871
YPL076W	973,55	1,18	0,24	1,34518576	0,17856525	0,2447589
YJR132W	1616,31	1,18	0,24	1,3449198	0,17865114	0,2448226
YBL039W-B	294,81	0,82	-0,29	-1,34470841	0,17871942	0,24486216
YFR008W	585,11	1,18	0,23	1,34391884	0,17897463	0,24515776
YBR125C	1047,09	0,86	-0,21	-1,34367939	0,17905209	0,2452004
YJL075C	17,43	2	1	1,34357856	0,17908471	0,2452004
YGL064C	392,82	0,81	-0,3	-1,34328037	0,1791812	0,24527846
YDR179W-A	326,98	0,81	-0,3	-1,3403133	0,18014351	0,24654107
YEL019C	181,84	1,27	0,34	1,34019195	0,18018295	0,24654107
YMR132C	297,77	0,82	-0,29	-1,33807793	0,18087103	0,24742807
YPL283C	14,42	0,47	-1,09	-1,33547259	0,18172172	0,24853706
YDR421W	1045,42	1,16	0,22	1,33419202	0,18214093	0,24898677

YKL221W	98,46	0,73	-0,45	-1,33410095	0,18217077	0,24898677
YML103C	3552,75	0,85	-0,23	-1,334125	0,18216289	0,24898677
YNL268W	3690,59	0,88	-0,18	-1,33389461	0,1822384	0,2490244
YLR157W-D	3,27	5,7	2,51	1,33364303	0,18232087	0,24908231
YPL111W	2096,29	0,86	-0,21	-1,33255915	0,18267652	0,24951331
YFL034W	1779,93	1,14	0,19	1,33221887	0,18278828	0,24961107
Q0115	1,12	19,04	4,25	1,32913517	0,18380338	0,25094211
YMR126C	584,34	1,18	0,23	1,32831017	0,18407566	0,25125862
YOR388C	26,38	1,83	0,87	1,32742522	0,18436806	0,25160246
YMR222C	1010,71	0,84	-0,25	-1,32636541	0,18471869	0,25202559
YBR069C	16,79	2,25	1,17	1,32503964	0,185158	0,2525695
YMR314W	5822,24	1,13	0,17	1,3248755	0,18521245	0,2525883
YDR060W	3678,65	1,21	0,28	1,32271532	0,18593007	0,25351132
YNR021W	4053,32	0,87	-0,21	-1,32229143	0,18607113	0,25364798
YFL007W	3670,53	1,18	0,24	1,32162365	0,1862935	0,2538954
YKL183W	829,51	1,19	0,25	1,32115499	0,18644969	0,25405253
YBR162C	11192,35	1,17	0,23	1,32101815	0,18649531	0,25405897
YNCP0001C	26,47	1,84	0,88	1,32055447	0,18664996	0,25421389
YDR012W	13976,98	0,89	-0,18	-1,31945169	0,18701815	0,25462991
YJL191W	3024,96	0,81	-0,31	-1,31939411	0,18703739	0,25462991
YML070W	6477,34	1,16	0,22	1,31902683	0,18716014	0,25474119
YMR065W	508,15	1,2	0,26	1,31863777	0,18729024	0,25486242
YBL027W	21840,17	0,86	-0,22	-1,31808095	0,18747655	0,25506008
YHL026C	321,39	0,83	-0,28	-1,31692069	0,18786521	0,25553288
YHR070W	992,99	1,16	0,22	1,31450035	0,18867788	0,25658209
YNCO0007W	19,15	0,52	-0,94	-1,31390733	0,18887739	0,25677932
YOL056W	1357,46	1,17	0,23	1,31382356	0,18890559	0,25677932
YGR025W	12,29	2,31	1,21	1,31334808	0,18906568	0,25694072
YOR351C	96,97	0,73	-0,45	-1,3123864	0,18938979	0,25732489
YGL219C	3031,88	1,14	0,19	1,31206197	0,18949923	0,25741727
YMR127C	430,15	0,79	-0,34	-1,3118092	0,18958452	0,25747683
YKL105C	2392,29	0,88	-0,18	-1,31061883	0,18998657	0,25796647
YNL323W	966,45	0,86	-0,22	-1,30919208	0,19046929	0,2585654
YOR258W	344,34	1,21	0,27	1,30833072	0,19076116	0,25890503
YJR085C	2793,54	0,79	-0,35	-1,30812078	0,19083235	0,25894507
YEL028W	160,16	0,77	-0,38	-1,30704214	0,1911984	0,25938512
YHR100C	545,8	1,18	0,24	1,30506023	0,19187234	0,26024257
YJL147C	438,92	1,19	0,25	1,30443693	0,19208465	0,26047366
YGR166W	1071,37	0,84	-0,25	-1,30230665	0,19281158	0,2613967
YNCM0022C	3,55	6,22	2,64	1,3021957	0,1928495	0,2613967
YHR045W	1114,23	0,87	-0,21	-1,30148404	0,19309283	0,26166942
YNCI0001W	56,2	1,52	0,6	1,30062853	0,19338564	0,26200907
YLL035W	561,17	1,26	0,33	1,29962723	0,19372876	0,26241672
YIL158W	760,02	1,19	0,25	1,29934375	0,19382599	0,26249118
YOR141C	2470,2	1,15	0,2	1,29893202	0,19396726	0,26262524
YMR163C	431,07	1,19	0,25	1,29664702	0,19475267	0,2636312
YDL160C-A	385,44	0,83	-0,27	-1,29623777	0,19489358	0,26376447
YHR023W	2417,72	1,16	0,21	1,29573165	0,19506795	0,26394296
YGL216W	892,33	0,86	-0,22	-1,29357157	0,19581345	0,26489398
YIL129C	2513	0,88	-0,19	-1,29212793	0,19631285	0,26545394

YJR030C	724,28	0,85	-0,24	-1,29218194	0,19629415	0,26545394
YDR507C	1640,1	0,84	-0,25	-1,29088621	0,19674314	0,26597788
YDR314C	502,31	0,84	-0,25	-1,29012011	0,19700896	0,2662793
YJL113W	311,6	1,21	0,27	1,28942245	0,19725126	0,2665488
YGR047C	1086,32	1,15	0,21	1,28888456	0,19743822	0,26665771
YMR182W-A	70,55	0,62	-0,69	-1,28889792	0,19743358	0,26665771
YPL096W	1313,37	0,87	-0,2	-1,28882009	0,19746064	0,26665771
YDR079W	679,66	0,82	-0,29	-1,28684914	0,19814687	0,26752627
YNCB0006W	1,72	9,67	3,27	1,28553615	0,19860499	0,26808653
YFR057W	1,72	9,65	3,27	1,28452686	0,19895767	0,26844593
YGL088W	1,72	9,65	3,27	1,28452686	0,19895767	0,26844593
YBL057C	977,52	0,86	-0,22	-1,28436579	0,199014	0,26846362
YOR098C	2032,23	0,87	-0,2	-1,28424155	0,19905745	0,26846394
YDR218C	55,94	1,6	0,68	1,2836315	0,19927092	0,2686935
YBL113C	359,23	0,83	-0,27	-1,28290223	0,19952633	0,26897006
YLR277C	1414,56	1,17	0,23	1,28279866	0,19956262	0,26897006
YOR182C	4339,19	1,13	0,18	1,28173436	0,19993585	0,26941464
YKL154W	848,46	0,84	-0,25	-1,28049112	0,20037247	0,26994443
YMR019W	965,79	1,16	0,21	1,28007844	0,20051755	0,27008131
YHR105W	338	0,83	-0,27	-1,27950466	0,2007194	0,27029458
YBR221W-A	5,79	2,97	1,57	1,27838996	0,20111196	0,27076452
YDR282C	639,5	0,86	-0,22	-1,27798923	0,20125322	0,270896
YLR466W	32,9	0,6	-0,73	-1,27603793	0,20194211	0,27176438
YCL016C	496,97	1,19	0,25	1,27501871	0,20230262	0,2721811
YOR340C	1637,81	0,83	-0,27	-1,27491482	0,20233939	0,2721811
YGL235W	2,83	4,83	2,27	1,27444231	0,2025067	0,27234719
YDR027C	2768,78	1,13	0,18	1,27205854	0,20335232	0,27342524
YNCF0004C	3,01	5,14	2,36	1,27057291	0,20388063	0,27407628
YDR263C	401,36	1,2	0,26	1,26965593	0,20420722	0,27445591
YBL011W	2224,43	0,88	-0,18	-1,26850946	0,20461607	0,27494593
YHR134W	614,85	1,16	0,22	1,26434521	0,20610615	0,27688828
YGR235C	1702,36	0,82	-0,28	-1,26408236	0,20620047	0,27695509
YDR364C	797,4	0,86	-0,21	-1,26388837	0,2062701	0,27698872
YKL170W	1361,33	0,83	-0,26	-1,25741194	0,20860451	0,28006294
YDR292C	3180,18	0,87	-0,2	-1,25592246	0,20914408	0,28072668
YNR048W	431,45	0,85	-0,24	-1,25351017	0,2100201	0,28184162
YOR322C	1524,58	0,88	-0,19	-1,25274753	0,2102976	0,28215307
YBR135W	844,31	1,17	0,23	1,25260515	0,21034944	0,28216168
YBL020W	903,31	1,19	0,25	1,25186063	0,21062065	0,28246448
YOR213C	533,07	0,85	-0,24	-1,24881502	0,21173274	0,28389462
YDR037W	17378,4	1,18	0,24	1,24857546	0,21182039	0,28395086
YMR188C	1834,53	1,17	0,23	1,24711036	0,21235703	0,28460882
YDR346C	4125,5	0,88	-0,19	-1,24561672	0,21290514	0,28522034
YNCL0038W	1,31	21,84	4,45	1,24571927	0,21286748	0,28522034
YGR178C	7483,07	0,89	-0,16	-1,24406282	0,21347644	0,28592403
YOR089C	4763,84	1,13	0,17	1,24390639	0,21353402	0,2859395
YOL147C	363,55	0,81	-0,31	-1,24321947	0,21378697	0,28621652
YER166W	2703,95	0,88	-0,18	-1,24015508	0,21491804	0,28766879
YFL065C	21,73	1,77	0,82	1,23935259	0,21521495	0,28788012
YML026C	15941,32	0,87	-0,2	-1,23944359	0,21518127	0,28788012

YNCP0009W	5,19	3,02	1,59	1,23951259	0,21515573	0,28788012
YEL049W	44,54	1,52	0,6	1,23607149	0,21643199	0,28944576
YNCL0047W	7,85	2,7	1,43	1,23462291	0,21697088	0,29010398
YBR050C	663,55	1,34	0,43	1,23260076	0,21772476	0,29101283
YOL021C	2196,25	0,87	-0,2	-1,23254838	0,21774431	0,29101283
YGR201C	832,32	0,79	-0,33	-1,23224513	0,21785753	0,29110152
YHR099W	4008,73	1,13	0,17	1,23205361	0,21792906	0,29113448
YCR075W-A	169,82	0,77	-0,38	-1,23185908	0,21800173	0,29116894
YNCN0007W	2,73	4,69	2,23	1,2305225	0,21850151	0,29171102
YNR012W	837,79	0,85	-0,24	-1,230564	0,21848598	0,29171102
YNL236W	2131,43	0,87	-0,2	-1,2301533	0,2186397	0,2918328
YPL279C	78,1	0,68	-0,55	-1,22982008	0,21876449	0,29193663
YBL111C	10,03	0,42	-1,24	-1,22967673	0,21881818	0,29194557
YER125W	2457,44	0,86	-0,21	-1,22735536	0,21968906	0,29302258
YGR079W	168,31	0,76	-0,4	-1,2271481	0,21976693	0,29302258
YOR143C	447,19	0,84	-0,25	-1,22726638	0,21972249	0,29302258
YJR079W	2,85	0,16	-2,6	-1,22362711	0,22109293	0,29472731
YPR108W-A	1,63	9,21	3,2	1,22303692	0,22131575	0,29496105
YCR008W	2422,44	0,88	-0,19	-1,22247342	0,22152865	0,29514255
YGL077C	2030,53	1,13	0,18	1,2221736	0,22164198	0,29514255
YNL334C	73,45	0,73	-0,46	-1,22233866	0,22157959	0,29514255
YPR054W	325,05	0,83	-0,26	-1,22223612	0,22161835	0,29514255
YHR024C	1957,59	1,13	0,18	1,22116344	0,22202414	0,29558808
YDR524C-B	20627,82	1,11	0,16	1,22081204	0,22215719	0,29570184
YMR256C	994,01	0,82	-0,28	-1,21778076	0,2233073	0,29716901
YOR311C	1625,34	0,88	-0,18	-1,21733124	0,22347821	0,29733276
YOR293W	14482,31	1,15	0,2	1,21698976	0,22360811	0,29744188
YLR271W	1074,85	1,18	0,23	1,21641036	0,22382864	0,29767149
YAR031W	421,6	0,84	-0,26	-1,21416266	0,22468561	0,29874723
YKL018W	725,49	0,86	-0,22	-1,21390723	0,22478315	0,29881296
YDR334W	1698,42	0,86	-0,22	-1,21300542	0,22512775	0,29914301
YNL051W	1396,91	1,14	0,18	1,213008	0,22512676	0,29914301
YNCH0015W	1,88	10,85	3,44	1,21079196	0,22597515	0,30020479
YFR012W	34,37	0,64	-0,64	-1,20933631	0,22653367	0,30088243
YGR017W	2191,7	0,89	-0,17	-1,20892262	0,22669257	0,30096854
YPL244C	960,41	0,86	-0,21	-1,20891528	0,2266954	0,30096854
YEL075C	9,81	2,22	1,15	1,20868167	0,22678517	0,30099865
YKL212W	5081,07	0,86	-0,21	-1,20844215	0,22687723	0,30099865
YLR456W	327,15	1,21	0,28	1,20842336	0,22688446	0,30099865
YNR004W	225,8	1,22	0,28	1,208352	0,2269119	0,30099865
YOR368W	631,48	0,86	-0,22	-1,20795407	0,22706493	0,30113735
YGL258W	53,97	0,69	-0,53	-1,2074559	0,22725662	0,30132724
YNL040W	1303,25	0,87	-0,2	-1,20669962	0,22754784	0,301649
YDR385W	18616,97	1,15	0,2	1,20592696	0,22784565	0,30197935
YER003C	6148	1,12	0,16	1,20542637	0,22803875	0,3021708
YJL044C	2403,93	0,88	-0,18	-1,20511426	0,2281592	0,30226593
YGL237C	930,46	0,85	-0,24	-1,20394296	0,22861162	0,30280073
YPR200C	91,01	1,4	0,49	1,2036634	0,2287197	0,3028793
YIL171W	36,3	1,55	0,64	1,20238661	0,22921378	0,30346888
YGL133W	2102,61	1,14	0,19	1,20093056	0,22977814	0,30409649

YIL007C	964,03	0,86	-0,22	-1,20091101	0,22978572	0,30409649
YNCD0002C	381,95	1,19	0,25	1,20003641	0,2301252	0,30448088
YNL275W	361,14	1,18	0,24	1,19819533	0,23084098	0,3053629
YLR175W	8448,42	1,15	0,2	1,19744488	0,2311332	0,30568436
YDR127W	5488,05	1,14	0,18	1,19712011	0,23125974	0,30578662
YLR226W	506,09	1,17	0,23	1,19688637	0,23135085	0,30584198
YOR177C	118,27	0,78	-0,36	-1,19642731	0,23152985	0,3060135
YBR251W	1707,41	1,15	0,2	1,19601925	0,23168905	0,30615877
YPL235W	3198,2	0,89	-0,17	-1,19543437	0,23191737	0,30639529
YDR452W	5744,61	1,12	0,16	1,19529574	0,2319715	0,30640165
YIL156W-B	723,93	0,8	-0,31	-1,19445956	0,23229825	0,30676801
YGL240W	166,77	0,79	-0,34	-1,19373758	0,23258064	0,30707565
YPL060C-A	50,3	0,67	-0,58	-1,1928252	0,23293784	0,30748191
YGR266W	1675,65	1,14	0,19	1,19182373	0,23333037	0,30793462
YDR030C	432,57	1,22	0,29	1,19083596	0,23371799	0,30838066
YNCF0003C	127,15	1,29	0,37	1,19018097	0,23397527	0,30865457
YNL168C	1788,94	1,15	0,2	1,1896128	0,23419861	0,3088836
YMR247W-A	125,07	0,78	-0,36	-1,1893128	0,2343166	0,30897362
YBR252W	798,36	0,86	-0,22	-1,18892309	0,23446993	0,30911019
YBR184W	219,89	0,82	-0,29	-1,18817646	0,23476389	0,30943206
YNL166C	1819,74	1,13	0,18	1,18670769	0,23534294	0,31012948
YLR166C	2232,69	1,14	0,18	1,18574234	0,23572407	0,31056584
YNL278W	655,71	0,87	-0,21	-1,18513901	0,23596249	0,31081403
YKR062W	1522,38	1,13	0,18	1,18394532	0,23643471	0,31137003
YNCH0005W	1,45	0,1	-3,34	-1,17904637	0,23837971	0,31386493
YKL057C	1965,08	0,86	-0,22	-1,17887029	0,23844983	0,31389072
YHR126C	5,51	2,83	1,5	1,17821618	0,23871043	0,31410066
YNCE0018W	5,51	2,83	1,5	1,17821618	0,23871043	0,31410066
YDL139C	378,53	1,21	0,27	1,17743222	0,23902304	0,31444538
YDR177W	3464	1,12	0,16	1,17689734	0,23923649	0,31459294
YKL206C	1089,13	0,88	-0,19	-1,1769267	0,23922476	0,31459294
YML098W	682,56	1,15	0,2	1,17676899	0,23928772	0,31459372
YER016W	1974,91	0,87	-0,2	-1,17514955	0,23993489	0,31537781
YER049W	2523,62	1,17	0,23	1,1708488	0,24165956	0,31757757
YLR133W	2260,81	1,14	0,18	1,16797771	0,24281576	0,3190295
YGL063W	563,88	1,18	0,24	1,16720061	0,24312937	0,31930647
YPL008W	1070,25	0,88	-0,19	-1,16721825	0,24312224	0,31930647
YDR498C	1341,32	0,87	-0,2	-1,16634483	0,24347506	0,3195578
YGR104C	764,72	1,15	0,2	1,16659102	0,24337557	0,3195578
YMR021C	752,07	0,85	-0,24	-1,1663658	0,24346658	0,3195578
YJR083C	981,8	1,15	0,2	1,16501673	0,24401222	0,32019517
YKL119C	599,09	1,16	0,21	1,16462676	0,24417011	0,32033468
YHR007C	5322,94	0,84	-0,25	-1,16386723	0,24447782	0,32067066
YDL235C	1716,85	1,13	0,18	1,16241017	0,24506889	0,32124245
YKL068W	2017,59	0,86	-0,21	-1,16254005	0,24501616	0,32124245
YMR106C	488,21	0,86	-0,21	-1,16248968	0,24503661	0,32124245
YGL006W-A	71,78	1,34	0,43	1,1603891	0,24589042	0,32225133
YLR131C	1246,44	0,89	-0,18	-1,15964108	0,24619497	0,32244637
YNCK0003W	1,45	0,1	-3,34	-1,15983127	0,24611751	0,32244637
YOR047C	1199,98	0,87	-0,21	-1,15972197	0,24616202	0,32244637

YER047C	1222,63	0,88	-0,18	-1,15854245	0,24664274	0,32290961
YPR190C	1151,87	0,83	-0,28	-1,15851821	0,24665263	0,32290961
YPR036W	11972,16	1,11	0,15	1,15717764	0,24719979	0,32355775
YNCM0025C	2,69	0,18	-2,5	-1,15668661	0,24740042	0,32375214
YBR163W	887,95	0,87	-0,2	-1,15504739	0,24807102	0,32456132
YDR047W	3320,28	1,12	0,16	1,15469883	0,24821378	0,32462825
YIL016W	752,5	0,87	-0,21	-1,15466732	0,24822669	0,32462825
YLR318W	418,09	1,18	0,24	1,15132796	0,24959734	0,32635206
YHL022C	137,6	1,26	0,34	1,1502424	0,25004405	0,32686734
YBR079C	7234,7	1,14	0,19	1,15005613	0,25012075	0,32689882
YDL225W	3430,78	1,12	0,16	1,14891074	0,25059279	0,32744687
YFR054C	26,87	0,61	-0,71	-1,14556479	0,25197527	0,3291841
YDR331W	730,06	1,16	0,21	1,14287845	0,25308906	0,33056965
YFL054C	1222,66	0,81	-0,31	-1,14270026	0,25316306	0,33059679
YML129C	1252,11	0,83	-0,27	-1,14107499	0,25383872	0,33140944
YIL090W	1203,08	0,88	-0,18	-1,14000804	0,25428295	0,33191967
YCR085W	18,24	0,53	-0,92	-1,13681177	0,25561698	0,3335909
YGR184C	3650,61	1,15	0,2	1,13639764	0,25579018	0,33374682
YBL063W	858,2	0,86	-0,22	-1,13600162	0,25595589	0,3338929
YMR063W	182,29	1,22	0,29	1,13510814	0,25633001	0,33431074
YLR094C	1240,36	1,13	0,18	1,13365581	0,25693895	0,33503459
YDL043C	685,46	0,88	-0,19	-1,13261355	0,25737658	0,33553479
YDR058C	650,7	1,2	0,27	1,13172339	0,25775074	0,33595208
YDR198C	750,54	1,15	0,21	1,13058804	0,25822853	0,33650014
YMR067C	1559,02	0,89	-0,18	-1,13046683	0,25827957	0,33650014
YBL036C	2341,19	1,13	0,17	1,12951889	0,258679	0,33694988
YCR077C	3726,52	0,88	-0,19	-1,12837542	0,2591614	0,33750747
YJR150C	71,15	0,72	-0,48	-1,12749986	0,2595312	0,33791822
YNCE0004C	4,75	3,26	1,7	1,12693627	0,25976943	0,33815752
YJR025C	2136,99	0,83	-0,27	-1,12610899	0,26011939	0,33854214
YBR063C	658,02	1,15	0,21	1,12566836	0,26030592	0,33871395
YOR042W	5251,89	0,9	-0,16	-1,12537074	0,26043196	0,33880699
YKL129C	4443,42	1,11	0,15	1,12458716	0,26076402	0,33916794
YOR355W	2580,36	1,17	0,23	1,12429513	0,26088784	0,33925796
YAL042W	6551,93	1,11	0,15	1,12169478	0,26199224	0,34062281
YMR030W-A	4,09	0,26	-1,97	-1,12025721	0,26260417	0,34120416
YMR138W	318,33	1,19	0,25	1,12045544	0,26251973	0,34120416
YMR303C	416,75	1,16	0,22	1,12037342	0,26255467	0,34120416
YDR475C	3347,65	0,9	-0,15	-1,1188103	0,26322108	0,34193419
YER096W	165,69	1,23	0,29	1,11852445	0,26334307	0,34202115
YPL030W	758,27	1,2	0,26	1,1175595	0,26375517	0,34248477
YOR034C	723,59	1,14	0,19	1,11652581	0,26419713	0,34295178
YOR299W	2067,02	0,89	-0,17	-1,11646009	0,26422524	0,34295178
YGL261C	22,16	1,79	0,84	1,11628565	0,26429988	0,34297699
YDR389W	1187,34	1,16	0,21	1,11601881	0,26441408	0,34298189
YKR001C	8414,39	0,89	-0,16	-1,11610908	0,26437545	0,34298189
YNCD0023C	3,48	4,48	2,16	1,11539384	0,26468169	0,34325734
YIR010W	1290,18	0,89	-0,17	-1,11515457	0,26478419	0,34331859
YHR050W-A	34,66	0,67	-0,58	-1,11427121	0,26516286	0,34373783
YDR335W	5330,3	1,14	0,18	1,11321137	0,26561766	0,34423506

YHR028C	5223,9	0,9	-0,15	-1,11299012	0,26571267	0,34423506
YJL194W	129,02	1,26	0,33	1,11305448	0,26568503	0,34423506
YGL194C	828,02	0,88	-0,18	-1,11282476	0,2657837	0,34425529
YOR049C	1473,61	1,15	0,2	1,11044332	0,26680804	0,34551001
YDL143W	4790,6	1,15	0,2	1,10860425	0,26760094	0,34646458
YJR158W	2,44	0,18	-2,46	-1,10731093	0,26815952	0,34711543
YLR100W	4164,49	1,11	0,15	1,10591931	0,26876144	0,3478221
YBR018C	192,33	0,8	-0,32	-1,1057019	0,26885557	0,34787144
YDR211W	3470,49	1,15	0,21	1,10524988	0,26905133	0,34805224
YAL067C	75,25	1,33	0,41	1,10274275	0,27013889	0,34938639
YIR025W	509,23	1,16	0,21	1,1025693	0,27021425	0,3494111
YOR185C	2313,18	1,13	0,17	1,10190395	0,27050343	0,34971224
YDL210W	106,96	0,78	-0,36	-1,10117734	0,27081948	0,35004798
YDR446W	25,06	1,62	0,7	1,10074361	0,27100826	0,35021912
YBR129C	1100,82	0,88	-0,18	-1,09730108	0,2725098	0,35208629
YKL094W	3549,34	0,87	-0,19	-1,09568591	0,27321625	0,35292563
YNCN0005C	219,33	1,34	0,42	1,09255567	0,27458894	0,35462504
YJL160C	89,57	1,3	0,38	1,09211588	0,27478217	0,35480084
YMR225C	833,51	1,16	0,21	1,09185627	0,27489628	0,35487442
YGL146C	256,47	1,26	0,33	1,09162311	0,27499879	0,354933
YBR276C	2195,79	1,12	0,17	1,09132805	0,27512856	0,35502671
YGR141W	1611,16	0,9	-0,16	-1,09118342	0,27519218	0,35503507
YNCO0014C	111,91	0,73	-0,45	-1,09061248	0,27544343	0,35528543
YBR035C	3128,37	0,87	-0,2	-1,08878408	0,27624911	0,35625067
YMR220W	2261,12	0,9	-0,16	-1,08854509	0,27635454	0,35631266
YLL012W	401,57	1,21	0,27	1,08798649	0,27660107	0,35655652
YPL276W	33,02	0,63	-0,67	-1,08766634	0,27674243	0,35666473
YLR234W	182,77	0,82	-0,28	-1,08692384	0,27707047	0,35701343
YML099C	1320,09	1,12	0,17	1,08453281	0,27812863	0,35830258
YER113C	880,02	0,87	-0,2	-1,0831011	0,27876355	0,35904606
YNCG0019W	1,98	5,05	2,34	1,08257497	0,27899712	0,35927241
YHR115C	2121,5	1,11	0,15	1,08208277	0,27921575	0,35933138
YLR082C	630,6	0,85	-0,23	-1,08232538	0,27910797	0,35933138
YNL102W	1442,12	0,86	-0,21	-1,08208121	0,27921645	0,35933138
YDR180W	607,14	1,19	0,26	1,08180066	0,27934111	0,35941735
YJR053W	254,11	0,84	-0,26	-1,08161191	0,27942501	0,35945085
YDR169C-A	59,41	1,37	0,45	1,07874821	0,28069999	0,36101622
YPL212C	1357,62	0,84	-0,25	-1,07799161	0,2810375	0,36137548
YDL211C	143,66	0,8	-0,32	-1,07766097	0,28118508	0,36149043
YOR012W	228,9	1,19	0,25	1,07729119	0,2813502	0,36162786
YJR124C	680,69	0,84	-0,25	-1,07676655	0,28158457	0,36185423
YHR015W	83,54	0,75	-0,42	-1,07547451	0,28216234	0,3625217
YIL092W	468,85	0,86	-0,21	-1,07427861	0,28269783	0,3631346
YFL009W	1808,25	0,89	-0,16	-1,07413518	0,2827621	0,36314206
YER011W	3383,52	1,56	0,65	1,07148095	0,28395323	0,36459642
YLR158C	1,03	17,61	4,14	1,07038559	0,28444578	0,36492714
YNCI0003C	1,03	17,61	4,14	1,07038559	0,28444578	0,36492714
YNCL0004C	1,03	17,61	4,14	1,07038559	0,28444578	0,36492714
YPL278C	68,28	0,75	-0,41	-1,07048842	0,28439952	0,36492714
YNL042W-B	10,15	2,16	1,11	1,06951831	0,28483618	0,36535255

YGL230C	30,96	1,49	0,58	1,0692326	0,28496487	0,36544215
YDR462W	829,94	0,84	-0,25	-1,06867789	0,28521484	0,3656867
YLR219W	2972,3	0,9	-0,15	-1,06854819	0,2852733	0,3656867
YKL069W	523,52	0,85	-0,23	-1,0673494	0,28581409	0,36630434
YPL148C	182,76	0,79	-0,33	-1,06715345	0,28590255	0,36634213
YKL103C	7040,14	0,88	-0,19	-1,0661289	0,28636538	0,36671625
YNCG0026W	25,59	0,64	-0,65	-1,06635799	0,28626185	0,36671625
YNCP0019W	29,05	1,66	0,73	1,06611508	0,28637162	0,36671625
YAR035C-A	3,23	4,06	2,02	1,06552433	0,28663872	0,36698263
YNCE0012W	4,75	2,69	1,43	1,06464843	0,28703506	0,36741433
YNL154C	4821,67	1,13	0,18	1,06407438	0,28729501	0,36767132
YNR075C-A	89,46	1,29	0,37	1,06341399	0,28759425	0,36797848
YER007W	425,82	0,87	-0,21	-1,0626443	0,28794329	0,3683492
YBR219C	9,31	2,03	1,02	1,06220813	0,28814121	0,36852649
YBR255W	1157,57	0,89	-0,17	-1,06155716	0,28843677	0,36856947
YLR005W	1272,51	0,89	-0,17	-1,06192875	0,28826803	0,36856947
YNCL0036C	1,29	0,11	-3,17	-1,06156093	0,28843506	0,36856947
YOL095C	360,69	0,86	-0,22	-1,06143051	0,2884943	0,36856947
YOR072W	43,57	0,7	-0,52	-1,06178101	0,28833511	0,36856947
YPR201W	302,54	0,84	-0,25	-1,06135015	0,2885308	0,36856947
YKL187C	522,24	0,86	-0,21	-1,06104915	0,28866757	0,36866836
YGR208W	2007,79	0,9	-0,15	-1,05947161	0,28938505	0,36950872
YOR354C	2496,29	1,11	0,15	1,05714974	0,29044325	0,3707837
YFL036W	2684,05	1,11	0,15	1,05562899	0,29113775	0,37159394
YJL201W	1393,65	1,13	0,18	1,05511011	0,29137497	0,37182032
YPR014C	226,87	1,2	0,27	1,05273522	0,29246236	0,37313128
YNL192W	6190,66	1,18	0,23	1,05188555	0,29285206	0,37355175
YNCL0039W	4,85	2,74	1,45	1,05150991	0,29302446	0,37369492
YKL046C	3009,1	1,11	0,15	1,05052756	0,29347562	0,37419347
YHR139C	1,38	7,48	2,9	1,04984474	0,2937895	0,37451681
YGL211W	840,88	0,88	-0,18	-1,049707	0,29385284	0,37452069
YBR084W	3586,46	0,86	-0,22	-1,04878859	0,29427543	0,37498235
YGL007W	22,92	1,58	0,66	1,04849147	0,29441223	0,37506533
YKL220C	276,41	0,82	-0,29	-1,04838491	0,2944613	0,37506533
YDR239C	933,88	1,12	0,17	1,04626552	0,29543846	0,37623282
YIL064W	744,55	0,87	-0,21	-1,046128	0,29550194	0,37623653
YHR159W	895,05	1,13	0,18	1,04598927	0,29556598	0,37624096
YMR319C	3116,87	1,13	0,18	1,04521586	0,29592322	0,37661852
YDL009C	68,07	1,46	0,55	1,04388642	0,29653794	0,3773008
YLR243W	746,77	0,87	-0,19	-1,04379384	0,29658078	0,3773008
YKR045C	348	0,85	-0,23	-1,0435847	0,29667757	0,37734666
YHR012W	1978,04	1,12	0,16	1,04343751	0,29674571	0,37735605
YGR106C	6391,18	0,91	-0,14	-1,04275354	0,29706246	0,37766191
YKR019C	632,88	1,14	0,19	1,04265562	0,29710782	0,37766191
YDL085C-A	650,33	1,19	0,25	1,04179836	0,29750517	0,3780123
YEL058W	2592,73	0,9	-0,14	-1,04185045	0,29748102	0,3780123
YGL137W	8347,4	0,9	-0,15	-1,04047366	0,2981199	0,37871591
YDR261C	2958,83	1,1	0,14	1,03953225	0,29855727	0,37919398
YJL206C	490,41	0,88	-0,19	-1,03588186	0,30025726	0,38127516
YCR006C	159,87	0,8	-0,32	-1,03564179	0,30036929	0,38133947

YER043C	17583,31	1,13	0,18	1,03248073	0,30184697	0,38313719
YGR111W	2909,57	0,9	-0,15	-1,03161047	0,30225463	0,38357626
YOR046C	5060,49	1,12	0,16	1,03026779	0,30288432	0,38429685
YGR276C	1589,44	1,12	0,16	1,03002598	0,30299781	0,38436234
YMR117C	237,55	1,19	0,25	1,02719587	0,30432824	0,38597122
YGR060W	3744,86	0,86	-0,22	-1,02625769	0,30477014	0,38645276
YDR337W	1419,16	0,88	-0,18	-1,02320044	0,30621308	0,3882032
YAL047C	773,68	0,89	-0,17	-1,02253114	0,30652958	0,38852515
YIL086C	19,78	1,62	0,69	1,02209317	0,30673681	0,38870849
YBR182C-A	18,42	1,67	0,74	1,02195219	0,30680353	0,38871375
YHR117W	2655,68	0,9	-0,15	-1,02166413	0,30693989	0,38880722
YJR138W	1220,67	0,89	-0,16	-1,01916173	0,30812619	0,39023036
YFL061W	52,74	0,73	-0,46	-1,01843529	0,30847114	0,39052282
YOR243C	2278,01	1,16	0,21	1,01841063	0,30848285	0,39052282
YPR037C	352,47	1,16	0,22	1,01805303	0,30865275	0,39065829
YLR003C	1104,87	1,13	0,18	1,01758267	0,30887633	0,39086163
YNL271C	4155,21	1,12	0,17	1,01528173	0,30997155	0,39216767
YBR062C	1077,28	1,14	0,18	1,01374807	0,31070298	0,39267506
YDR480W	761,1	0,87	-0,21	-1,01413505	0,31051832	0,39267506
YJR084W	1092,46	0,89	-0,16	-1,01362099	0,31076364	0,39267506
YKL058W	955,65	0,87	-0,2	-1,01351324	0,31081508	0,39267506
YKR018C	6220,42	1,1	0,13	1,01360753	0,31077007	0,39267506
YMR179W	621,58	0,86	-0,22	-1,01404414	0,31056169	0,39267506
YMR190C	1242,21	1,12	0,16	1,01358572	0,31078047	0,39267506
YCR052W	2133,9	0,9	-0,15	-1,01299648	0,31106184	0,39290691
YNL233W	1371,61	1,12	0,16	1,01251663	0,3112911	0,39311655
YHL038C	968	1,13	0,17	1,0110797	0,31197829	0,39390429
YEL063C	2353,59	0,91	-0,14	-1,01079347	0,31211529	0,39399719
YAL067W-A	0,78	13,14	3,72	1,00987752	0,31255397	0,39407049
YDR194W-A	0,78	13,14	3,72	1,00987752	0,31255397	0,39407049
YJL117W	3059,79	1,11	0,15	1,01037817	0,31231414	0,39407049
YNCE0006W	0,78	13,14	3,72	1,00987752	0,31255397	0,39407049
YNCG0030C	0,78	13,14	3,72	1,00987752	0,31255397	0,39407049
YNCN0012C	0,78	13,14	3,72	1,00987752	0,31255397	0,39407049
YBR264C	714,54	1,13	0,18	1,00926813	0,31284606	0,39430839
YNCO0006W	13,31	1,97	0,98	1,009219	0,31286962	0,39430839
YJR144W	1363,53	0,9	-0,15	-1,00693972	0,31396374	0,39560705
YDR170C	6008,27	1,12	0,16	1,0052652	0,31476917	0,39654148
YNCM0012W	19,1	0,61	-0,72	-1,0050803	0,31485819	0,39657321
YER039C-A	43,73	0,72	-0,47	-1,00236192	0,31616883	0,39814327
YKL181W	5562,33	0,9	-0,15	-1,00215524	0,31626862	0,39818822
YNCM0021C	1,28	7,02	2,81	1,00162981	0,31652242	0,398427
YBR045C	266,98	1,18	0,24	1,00067362	0,31698462	0,39892797
YJL208C	725,14	0,88	-0,19	-0,99939085	0,31760539	0,39954732
YNR050C	3330,2	0,89	-0,17	-0,99947033	0,3175669	0,39954732
YCR088W	10175,76	0,9	-0,15	-0,99907738	0,31775721	0,39965738
YNCF0009C	2,28	0,2	-2,36	-0,9984817	0,31804584	0,39993942
YLR449W	6235,31	1,2	0,27	0,99788013	0,31833749	0,40022516
YLR405W	591,9	0,87	-0,19	-0,99736635	0,31858672	0,40045744
YGL254W	522,77	1,16	0,21	0,99663491	0,31894175	0,40074154

YOR205C	729,62	0,89	-0,18	-0,99664789	0,31893545	0,40074154
YDR293C	8883,82	0,91	-0,13	-0,99644605	0,31903347	0,40077571
YNCO0029C	2,27	0,2	-2,35	-0,99485949	0,31980461	0,40166321
YDL104C	375,43	0,87	-0,2	-0,99429085	0,32008129	0,40184821
YDR075W	448,83	0,88	-0,19	-0,99436583	0,3200448	0,40184821
YHR034C	581,15	1,13	0,18	0,99280531	0,32080484	0,40267522
YDR280W	1238,83	0,9	-0,15	-0,9900317	0,32215862	0,4042928
YOR117W	4650,44	1,1	0,13	0,98967048	0,32233521	0,4044327
YHR129C	535,07	1,14	0,18	0,98806136	0,3231226	0,40533877
YOR293C-A	13,99	1,71	0,78	0,98763297	0,32333243	0,4055201
YFL042C	2526	0,9	-0,15	-0,98618206	0,32404378	0,40633023
YKL020C	2483,5	1,1	0,14	0,98569091	0,32428481	0,4065504
YNR066C	253,8	1,17	0,23	0,98552811	0,32436473	0,40656854
YNL024C-A	614,1	1,13	0,18	0,98519629	0,32452766	0,40669071
YIL069C	7803,51	1,11	0,15	0,98397886	0,32512591	0,40735824
YJL076W	3151,76	0,89	-0,17	-0,98271523	0,32574761	0,40799801
YJL136C	17544,09	1,09	0,13	0,98254065	0,32583357	0,40799801
YJR003C	870,1	0,89	-0,17	-0,98266948	0,32577014	0,40799801
YCR027C	588,47	0,87	-0,2	-0,98239436	0,3259056	0,40800596
YGR227C-A	11,4	1,82	0,87	0,98196637	0,32611641	0,40818762
YER180C	900,94	1,12	0,16	0,98002965	0,32707148	0,40930057
YBR210W	912,57	0,89	-0,16	-0,97938614	0,32738922	0,40961568
YLR031W	90,07	0,78	-0,35	-0,97828209	0,32793482	0,41021569
YNCJ0002C	1,88	4,84	2,28	0,978087	0,3280313	0,41025376
YOR285W	4454,97	0,85	-0,24	-0,97640935	0,32886165	0,41120946
YJL030W	555,59	0,89	-0,17	-0,97323147	0,33043828	0,41309772
YPR145C-A	306,6	0,84	-0,25	-0,97255614	0,33077395	0,41343416
YFL050C	622,44	1,14	0,19	0,97213364	0,33098407	0,41361356
YER121W	277,76	0,78	-0,36	-0,97046454	0,33181499	0,41448516
YGR010W	1224,46	0,89	-0,16	-0,9704733	0,33181063	0,41448516
YOL052C-A	8270,49	1,48	0,56	0,9699012	0,33209574	0,41475247
YLR375W	2617,7	0,9	-0,16	-0,96942468	0,33233334	0,41496578
YLR067C	642,58	0,89	-0,17	-0,96911176	0,33248943	0,41507724
YGL130W	1747,33	1,1	0,14	0,96887725	0,33260644	0,41512631
YLR384C	2951,17	1,14	0,19	0,96876513	0,33266239	0,41512631
YML105C	1685,01	0,91	-0,14	-0,96817012	0,33295941	0,41541351
YIL147C	1696	1,13	0,17	0,96741135	0,33333844	0,41580289
YGR043C	1191,89	0,64	-0,64	-0,9666689	0,33370958	0,41618227
YDL072C	4609,92	1,09	0,13	0,96403701	0,33502737	0,41774188
YER115C	755,61	1,14	0,18	0,96181491	0,33614259	0,41904831
YGR179C	754,16	0,89	-0,17	-0,96145561	0,33632314	0,41918927
YNL204C	163,4	0,82	-0,29	-0,96104115	0,33653148	0,41930857
YOL023W	888,47	1,11	0,15	0,96099666	0,33655385	0,41930857
YJL100W	1499	1,14	0,19	0,96073588	0,33668498	0,41938783
YBR032W	32,54	0,69	-0,53	-0,96043996	0,33683384	0,41938911
YOL164W-A	37,61	0,68	-0,55	-0,96033122	0,33688854	0,41938911
YOR062C	548,44	1,15	0,21	0,9604327	0,33683749	0,41938911
YBR170C	1865,48	0,9	-0,15	-0,95879998	0,33765952	0,42026466
YNL157W	2740,09	0,91	-0,14	-0,95828798	0,33791756	0,42050158
YDL201W	1074,29	0,88	-0,19	-0,95677697	0,33867984	0,42136574

YBR166C	1871,66	1,1	0,14	0,95615678	0,33899303	0,42159971
YML075C	1853,19	0,9	-0,15	-0,9561358	0,33900363	0,42159971
YFL068W	1,13	0,13	-2,98	-0,95480287	0,33967739	0,42235308
YDR237W	1646,81	1,11	0,16	0,95339367	0,34039065	0,42315524
YLR419W	1038,72	0,88	-0,19	-0,95141459	0,34139396	0,42431758
YDR165W	1778,21	1,14	0,19	0,95097784	0,34161563	0,42450815
YLR099C	1271,05	1,12	0,16	0,94936567	0,34243466	0,42535281
YLR441C	31246,17	0,89	-0,16	-0,94934373	0,34244582	0,42535281
YOR023C	2526,06	1,11	0,15	0,94923567	0,34250077	0,42535281
YBL007C	6971,79	0,91	-0,14	-0,94872992	0,34275799	0,42558718
YNR003C	1237,88	1,12	0,16	0,94840143	0,34292513	0,42570961
YKR094C	15498,99	0,89	-0,16	-0,94748819	0,34339007	0,42620162
YKL151C	5003,34	1,15	0,21	0,94528724	0,34451225	0,42750901
YPR155C	1676,26	0,86	-0,22	-0,94504011	0,3446384	0,42758013
YPR120C	409,28	0,88	-0,18	-0,94481701	0,34475231	0,42763605
YJR070C	3615,49	0,9	-0,15	-0,94412263	0,34510699	0,42799054
YNCG0021W	3,15	2,89	1,53	0,94243864	0,34596811	0,42897284
YGL145W	1600,7	1,11	0,15	0,94223138	0,34607419	0,42901873
YPR186C	339,13	1,23	0,3	0,94199215	0,34619666	0,42908493
YKL190W	2365,73	1,1	0,14	0,94141266	0,34649343	0,42928145
YMR246W	3975,28	0,87	-0,19	-0,94143065	0,34648421	0,42928145
YDL078C	4903,92	0,89	-0,17	-0,940567	0,3469268	0,42973266
YNL151C	1071,36	0,9	-0,16	-0,94019697	0,34711653	0,42988196
YDR240C	406,61	0,88	-0,19	-0,93991389	0,34726173	0,42992738
YLL064C	4,51	2,47	1,3	0,93985562	0,34729162	0,42992738
YNCM0020W	8,87	1,89	0,92	0,93945425	0,34749757	0,43009661
YGR034W	16130,99	1,12	0,16	0,9382339	0,34812422	0,43078639
YDL191W	4736,81	0,9	-0,15	-0,9378596	0,34831657	0,43093856
YOR274W	784,98	0,9	-0,16	-0,93763376	0,34843265	0,43099634
YDR062W	5307,76	0,91	-0,14	-0,93713216	0,34869058	0,43121122
YKL047W	1053,72	0,9	-0,15	-0,93697862	0,34876956	0,43121122
YPL160W	9706,35	1,15	0,2	0,93689103	0,34881461	0,43121122
YGL001C	2680,18	0,91	-0,14	-0,93650233	0,34901461	0,43137262
YLR179C	5306,74	0,92	-0,13	-0,9357647	0,34939435	0,43175606
YBR243C	1283,45	0,91	-0,14	-0,93530446	0,34963142	0,43196308
YMR020W	2232,26	1,1	0,14	0,9326952	0,35097736	0,43353975
YNCE0014W	0,87	14,55	3,86	0,93214551	0,35126133	0,43371803
tP(UGG)Q	0,87	14,55	3,86	0,93214551	0,35126133	0,43371803
YNCK0011C	1,13	0,13	-2,99	-0,93192852	0,35137347	0,43377027
YPR001W	227,42	1,23	0,3	0,93146327	0,35161397	0,43398093
YCR024C-A	2576,54	0,88	-0,19	-0,9292176	0,35277633	0,43532907
YNL113W	788,96	1,13	0,18	0,92829269	0,35325576	0,43583412
YGL138C	6,5	2,13	1,09	0,92588066	0,354508	0,43725662
YOL137W	770,06	1,12	0,16	0,9258008	0,35454951	0,43725662
YPR017C	457,29	1,13	0,17	0,92463001	0,3551584	0,4379206
YPL273W	4458,45	0,9	-0,15	-0,92448798	0,35523231	0,4379248
YLR043C	5767,66	1,09	0,13	0,92380919	0,35558567	0,43827345
YLR467W	4,19	0,37	-1,43	-0,92327591	0,35586344	0,4385288
YDR504C	231,58	0,86	-0,21	-0,92309289	0,3559588	0,43855931
YOR361C	7841,32	1,1	0,14	0,92272431	0,35615089	0,43870897

YLR268W	1742,94	0,91	-0,14	-0,92156747	0,35675423	0,43936504
YNL082W	800,03	0,88	-0,19	-0,92107214	0,35701276	0,43959629
YBR131W	468,61	0,87	-0,2	-0,92007091	0,3575357	0,44014943
YPL124W	272,94	1,15	0,2	0,9199408	0,3576037	0,44014943
YOR336W	1215,31	0,89	-0,17	-0,91956446	0,35780041	0,44030431
YMR320W	274,33	0,85	-0,24	-0,91732231	0,35897377	0,44166074
YBL038W	1274,21	0,88	-0,18	-0,91714643	0,35906592	0,44168663
YLR424W	470,04	0,88	-0,19	-0,9148032	0,36029495	0,44311072
YDR405W	1862,83	1,12	0,16	0,91427552	0,36057209	0,44336378
YNCM0017W	27,28	1,5	0,59	0,91155017	0,36200556	0,44503831
YMR174C	816	1,22	0,29	0,91044546	0,36258763	0,44566569
YDR142C	662,29	0,89	-0,18	-0,90901054	0,36334456	0,4465077
YGL078C	2921,15	1,13	0,17	0,90860821	0,36355697	0,44668036
YNCD0018W	2,07	5,35	2,42	0,90812141	0,36381408	0,44690786
YHR057C	1494,61	1,11	0,15	0,90786327	0,36395047	0,44698701
YDR260C	537,98	0,89	-0,16	-0,90671192	0,36455916	0,44764607
YBR181C	11545,78	1,12	0,16	0,90524376	0,36533626	0,44851163
YJR133W	958,64	1,1	0,14	0,90467148	0,36563945	0,44879516
YGL129C	1947,5	0,91	-0,13	-0,90301052	0,36652031	0,44978747
YLL057C	270,11	1,17	0,22	0,90260591	0,36673509	0,44996215
YNL297C	2659,36	1,11	0,15	0,90187051	0,36712566	0,45035241
YDL047W	3082,08	1,09	0,12	0,90085364	0,36766614	0,45092637
YPL070W	1838,57	0,91	-0,14	-0,89963703	0,36831344	0,45163109
YBR288C	1641,12	1,11	0,15	0,89866825	0,36882939	0,4521745
YLR057W	978,18	1,11	0,15	0,89844396	0,36894891	0,45223177
YLR438W	1094,53	1,11	0,15	0,89638653	0,37004636	0,45348747
YNL188W	814,27	0,9	-0,16	-0,8959187	0,37029618	0,45370412
YHR020W	6106,39	1,15	0,2	0,89573049	0,37039672	0,45373781
YDR321W	3094,23	0,91	-0,14	-0,89555894	0,37048837	0,4537606
YPR134W	710,32	1,14	0,19	0,89518066	0,37069052	0,45391869
YKR050W	1038,4	1,1	0,14	0,89386193	0,37139577	0,45468831
YNL331C	429,69	1,13	0,18	0,89373178	0,37146542	0,45468831
YGL231C	2025,2	1,1	0,13	0,89329241	0,3717006	0,45488655
YNCD0022C	2,74	3,18	1,67	0,89233762	0,37221199	0,45542267
YNR062C	228,63	1,16	0,21	0,89158435	0,37261576	0,45582692
YEL077C	588,46	1,12	0,17	0,8914193	0,37270427	0,45584543
YER187W	229,13	0,85	-0,23	-0,88727664	0,37492998	0,45847737
YAL064C-A	70,32	0,79	-0,34	-0,88673058	0,37522397	0,45874656
YJL105W	206,97	1,16	0,21	0,88564336	0,37580974	0,45937232
YGL019W	2606,47	1,1	0,14	0,88308394	0,37719092	0,4609699
YNL148C	604,73	0,9	-0,15	-0,88023403	0,37873254	0,46276289
YBL006C	1526,49	1,13	0,18	0,87884745	0,37948399	0,46358988
YHR213W	5,17	2,19	1,13	0,87856199	0,37963881	0,46368783
YHL012W	148,66	1,18	0,24	0,87726907	0,38034051	0,46445356
YDL102W	1074,11	0,89	-0,17	-0,87656641	0,38072219	0,46482827
YMR218C	1522,82	1,12	0,16	0,87634818	0,38084078	0,46488169
YGL017W	904,79	1,1	0,14	0,87503682	0,38155387	0,46566064
YOR223W	1011,65	1,11	0,15	0,8745018	0,38184504	0,46592446
YIL152W	726,29	1,11	0,15	0,87404041	0,38209625	0,46613942
YKR038C	2152,54	1,09	0,12	0,8737823	0,38223682	0,46621935

YNCL0033C	1,95	0,24	-2,09	-0,87312047	0,38259742	0,46647599
YOL120C	25442,86	0,92	-0,13	-0,87319661	0,38255592	0,46647599
YLL066C	75,21	0,79	-0,34	-0,87199079	0,3832134	0,46704369
YPL001W	606,51	1,14	0,19	0,87206544	0,38317268	0,46704369
YGR258C	1872,58	0,91	-0,13	-0,87137703	0,38354832	0,46736017
YLR328W	1583	1,09	0,13	0,87104451	0,38372985	0,46748967
YNR064C	147,14	1,18	0,24	0,86526947	0,3868909	0,47124829
YGL260W	11,28	1,71	0,77	0,86509721	0,38698544	0,47127103
YDR147W	839,17	0,9	-0,15	-0,86435463	0,38739311	0,47167502
YDR226W	8238,46	0,9	-0,15	-0,86307806	0,38809455	0,47243648
YDR184C	423,99	1,15	0,2	0,86118218	0,38913771	0,47361353
YNCO0031W	2,05	0,25	-2,01	-0,86020059	0,38967848	0,47417879
YHR131C	1369,03	0,91	-0,13	-0,8596429	0,38998592	0,47445996
YPL275W	30,24	0,7	-0,52	-0,85923864	0,39020887	0,47463824
YNR027W	938,27	1,11	0,15	0,85791893	0,39093723	0,47543111
YOR009W	413,19	0,89	-0,17	-0,85708094	0,39140016	0,47590091
YKL186C	1793,27	0,92	-0,13	-0,85635272	0,39180271	0,47629715
YKL138C	1579,23	1,1	0,13	0,85562287	0,39220642	0,47660138
YMR053C	890,3	0,91	-0,14	-0,85566775	0,39218158	0,47660138
YNCE0007C	149,34	0,75	-0,41	-0,85420649	0,39299059	0,47746089
YLR255C	33,28	0,72	-0,47	-0,85281224	0,39376343	0,47830631
YLR397C	1241,38	1,12	0,16	0,85164825	0,39440935	0,47899724
YHR213W-A	1,7	4,36	2,13	0,85109859	0,39471459	0,47927425
YFR026C	128,08	0,83	-0,27	-0,85091836	0,3948147	0,47930212
YLR357W	1522,14	0,91	-0,14	-0,84951727	0,39559353	0,48015377
YOR359W	368,36	0,86	-0,22	-0,84854956	0,39613198	0,4807134
YKR080W	4707,24	0,89	-0,17	-0,8479933	0,3964417	0,48099528
YNCE0009C	2,05	0,25	-2	-0,84703409	0,39697612	0,48154963
YGR232W	988,32	0,9	-0,15	-0,84479153	0,39822724	0,48297299
YJR142W	2062,65	0,92	-0,13	-0,84439491	0,39844877	0,48314732
YBL056W	5441,73	0,92	-0,12	-0,84420685	0,39855383	0,4831804
YOR396W	6,76	0,5	-0,99	-0,84250835	0,39950347	0,48423718
YPR193C	76,33	0,8	-0,33	-0,84201637	0,39977879	0,48447637
YHL019C	1539,27	0,92	-0,13	-0,84170455	0,39995335	0,48459337
YKL065W-A	85,25	1,27	0,35	0,84116406	0,40025603	0,48486554
YDL134C	3505,06	0,92	-0,12	-0,84019774	0,40079753	0,48542684
YDR524C	1225,26	0,91	-0,14	-0,84004068	0,40088558	0,48543883
YBR122C	2162,23	0,91	-0,13	-0,83978838	0,40102705	0,4855155
YNCL0032C	17,57	1,54	0,63	0,83803107	0,40201325	0,48661464
YBL025W	370,61	1,12	0,17	0,83732161	0,40241181	0,48700218
YDL200C	314,85	1,14	0,19	0,83706428	0,40255644	0,48708231
YLR407W	358,5	1,13	0,18	0,83647646	0,40288692	0,48738725
YOR381W-A	22,94	1,46	0,55	0,83499821	0,40371872	0,48829841
YDR492W	438,38	0,9	-0,16	-0,83130505	0,40580132	0,49072178
YKL097C	11,14	1,76	0,82	0,82903932	0,40708216	0,49184646
YNCB0008W	57,66	0,78	-0,35	-0,82938834	0,4068847	0,49184646
YNCE0025C	2,04	0,25	-2	-0,82928907	0,40694085	0,49184646
YNL196C	111,73	1,22	0,28	0,82895963	0,40712725	0,49184646
YPL267W	350,18	1,13	0,17	0,82901973	0,40709324	0,49184646
YDR034C-A	13,39	0,6	-0,74	-0,82855571	0,40735586	0,49202695

YBR213W	265,44	0,87	-0,19	-0,82749533	0,40795637	0,49256073
YLR272C	895,72	0,9	-0,15	-0,82759772	0,40789836	0,49256073
YDR201W	315,82	1,15	0,2	0,82734021	0,40804426	0,49257111
YJL058C	361,54	1,12	0,17	0,82632261	0,40862111	0,49317162
YHL032C	2229,41	0,88	-0,18	-0,82544168	0,40912088	0,4935367
YLR320W	661,86	1,13	0,17	0,82536932	0,40916195	0,4935367
YPL120W	902,46	0,91	-0,14	-0,82540794	0,40914003	0,4935367
YOL009C	728,64	0,9	-0,15	-0,8219409	0,41111053	0,49579084
YDR013W	378,87	0,85	-0,24	-0,82123754	0,41151098	0,49617745
YLR378C	7316,91	0,92	-0,12	-0,8210818	0,41159968	0,49618808
YFL002C	1249,31	1,12	0,16	0,82071288	0,41180983	0,49624882
YMR316C-A	7,92	1,91	0,94	0,82077761	0,41177295	0,49624882
YOL034W	680,08	1,13	0,18	0,81972296	0,41237406	0,49683236
YKR073C	16,92	0,64	-0,65	-0,81942963	0,41254134	0,49693752
YJR140C	1188,02	0,9	-0,16	-0,81873415	0,41293811	0,49731903
YNCJ0015W	0,96	0,15	-2,76	-0,81721455	0,41380583	0,49826746
YPL173W	1165,7	1,1	0,14	0,8167645	0,41406302	0,49848052
YDR447C	8299,49	1,08	0,11	0,8156368	0,41470789	0,49916013
YMR115W	1495,6	0,92	-0,12	-0,81543379	0,41482405	0,49920321
YMR048W	548,92	0,9	-0,15	-0,81526483	0,41492074	0,49922286
YJR092W	2300,97	0,92	-0,12	-0,81199402	0,41679506	0,50138089
YHL005C	8,08	1,81	0,85	0,81115905	0,41727434	0,50186025
YDR381C-A	1108,07	1,16	0,21	0,81088898	0,41742943	0,50189967
YJL024C	1247,7	0,91	-0,13	-0,8108206	0,4174687	0,50189967
YPR089W	2474,86	0,92	-0,12	-0,81019117	0,41783031	0,50223721
YAR003W	776,2	0,91	-0,13	-0,80914888	0,41842951	0,50286015
YMR260C	7684,22	0,92	-0,12	-0,80890241	0,41857129	0,50287714
YPR051W	892,02	1,1	0,14	0,80884284	0,41860555	0,50287714
YKR054C	2197,51	0,92	-0,12	-0,80832815	0,4189017	0,50313561
YLR443W	824,03	1,1	0,14	0,80751422	0,41937028	0,50350371
YOL097W-A	16,01	0,65	-0,62	-0,80761071	0,41931472	0,50350371
YER164W	3513,32	1,08	0,12	0,80712121	0,41959665	0,50367815
YDR145W	3241,73	1,09	0,12	0,80676353	0,41980273	0,50373538
YDR254W	840,63	0,91	-0,14	-0,80662194	0,41988433	0,50373538
YKR100C	1877,08	0,92	-0,12	-0,80661626	0,4198876	0,50373538
YMR213W	742,96	0,91	-0,14	-0,80626926	0,42008761	0,50387802
YBR236C	1639,17	1,09	0,12	0,80504711	0,4207925	0,50462606
YNCA0006C	2,23	3,54	1,82	0,8033685	0,42176178	0,50569082
YDR287W	1123,02	0,91	-0,13	-0,80175053	0,42269728	0,50671469
YAL060W	7284,78	0,71	-0,49	-0,80156046	0,42280726	0,50674873
YDR079C-A	340,6	1,14	0,19	0,80063189	0,42334478	0,5072951
YPL157W	710,69	1,11	0,15	0,79728204	0,42528725	0,50952447
YMR195W	1945,82	1,14	0,19	0,79665012	0,42565426	0,50986584
YLR264W	2508,02	1,15	0,2	0,7956305	0,42624684	0,51047722
YBR004C	950,98	1,1	0,13	0,79466246	0,42680988	0,51093542
YNCK0007W	1,8	0,26	-1,94	-0,79472516	0,4267734	0,51093542
YOR323C	4774,03	0,93	-0,11	-0,79454851	0,42687618	0,51093542
YBL048W	77,3	1,23	0,3	0,79373394	0,42735034	0,51140441
YBR164C	2107,82	1,1	0,14	0,79351906	0,42747547	0,51145563
YIL035C	3930,45	1,08	0,11	0,79329538	0,42760575	0,51151298

YBR275C	1844,45	1,11	0,16	0,79217109	0,42826092	0,51219808
YBR029C	1484,81	1,09	0,13	0,79142746	0,42869459	0,51242078
YLR176C	1259,14	0,92	-0,13	-0,79147793	0,42866515	0,51242078
YOL057W	3829,61	0,92	-0,13	-0,79152182	0,42863955	0,51242078
YOR391C	6,15	1,96	0,97	0,79020352	0,42940892	0,51317588
YDR123C	361,54	1,12	0,17	0,78970771	0,42969849	0,51342316
YLR421C	2510,94	1,08	0,12	0,7865737	0,43153146	0,515415
YPR042C	1218,34	0,92	-0,12	-0,78665773	0,43148225	0,515415
YOR180C	207,3	0,87	-0,2	-0,785915	0,43191728	0,51577666
YNCD0025W	0,69	11,75	3,55	0,78538255	0,43222931	0,5159509
YNCN0004W	0,69	11,75	3,55	0,78538255	0,43222931	0,5159509
YNL152W	1220,43	1,09	0,12	0,78513975	0,43237164	0,51602163
YKL008C	2264,63	1,08	0,11	0,78428971	0,43287013	0,51651734
YBR200W-A	13,09	1,6	0,68	0,78208331	0,43416561	0,51796366
YNCO0016W	9,1	1,69	0,76	0,77981146	0,43550186	0,51945806
YBR294W	598,63	0,91	-0,14	-0,77854861	0,43624566	0,52024535
YNCD0016C	1,63	3,97	1,99	0,77795528	0,43659537	0,52046257
YNCK0010W	1,63	3,97	1,99	0,77795528	0,43659537	0,52046257
YGR094W	11379,76	1,11	0,15	0,7772881	0,43698881	0,52064526
YJR010C-A	507,34	0,9	-0,15	-0,77726903	0,43700006	0,52064526
YPL183C	1131,14	1,12	0,16	0,77728431	0,43699105	0,52064526
YBR200W	2124,28	1,08	0,11	0,77319671	0,43940597	0,5234113
YOR003W	548,47	1,11	0,16	0,77274513	0,43967323	0,52362924
YDL146W	1156,24	0,92	-0,12	-0,77240351	0,43987547	0,52366931
YNCG0031W	1,45	3,51	1,81	0,77242326	0,43986378	0,52366931
YNCB0017C	2,14	3,37	1,75	0,7718561	0,44019966	0,52395483
YOL122C	1901,51	1,08	0,11	0,77060741	0,44093967	0,5247351
YOL020W	1367,9	0,92	-0,11	-0,76875757	0,44203724	0,5259405
YNCG0008W	4,16	2,19	1,13	0,76851832	0,44217931	0,52600879
YPL041C	232,72	0,88	-0,19	-0,76782488	0,44259123	0,526398
YGL020C	1634,63	1,08	0,11	0,76725958	0,4429272	0,52669674
YLL054C	766,54	0,91	-0,14	-0,76708608	0,44303033	0,52671855
YJL037W	19,59	0,68	-0,56	-0,76669354	0,44326374	0,52689521
YDL022C-A	3,95	0,43	-1,2	-0,76637677	0,44345215	0,52694822
YDR104C	702,51	1,1	0,13	0,76633333	0,44347799	0,52694822
YBL081W	359,79	1,13	0,18	0,76593805	0,44371317	0,52712683
YGR200C	2498,6	1,09	0,13	0,76501741	0,44426118	0,52767695
YOR304C-A	236,05	0,88	-0,18	-0,76418046	0,44475971	0,5281681
YPR033C	4670,96	0,92	-0,12	-0,76353885	0,4451421	0,52852116
YBR073W	2562,79	0,91	-0,14	-0,76293519	0,44550205	0,52873241
YHR149C	982,23	1,11	0,15	0,76281227	0,44557537	0,52873241
YIL074C	3422,38	1,08	0,11	0,76284523	0,4455557	0,52873241
YJL216C	159,7	0,85	-0,23	-0,76085186	0,44674555	0,53001974
YJL180C	834,72	0,92	-0,12	-0,75903078	0,44783414	0,5312098
YOR034C-A	45,88	0,78	-0,36	-0,75530311	0,45006713	0,53375659
YEL023C	584,65	1,1	0,14	0,75391117	0,45090256	0,5346453
YNCD0020W	30,84	0,73	-0,45	-0,75295626	0,4514762	0,53522331
YOR008C-A	51,93	1,28	0,36	0,75228528	0,45187952	0,53549706
YOR022C	859,47	0,91	-0,14	-0,752415	0,45180153	0,53549706
YGR252W	1633,67	1,08	0,11	0,75130117	0,45247142	0,53609622

YGR056W	1420,62	0,92	-0,13	-0,75089686	0,45271473	0,53628221
YGR016W	221,34	1,13	0,18	0,75022841	0,45311715	0,53665658
YOR137C	773,42	0,91	-0,13	-0,74995339	0,45328278	0,5367504
YLR350W	4808,74	1,08	0,11	0,74904621	0,45382935	0,53729521
YMR159C	205,18	1,14	0,19	0,74874492	0,45401097	0,5374078
YER081W	2453,72	0,66	-0,59	-0,74812837	0,45438273	0,53774538
YNR061C	832,54	1,1	0,14	0,74710735	0,45499876	0,53837187
YBL003C	4641,88	0,93	-0,1	-0,74578922	0,45579475	0,53921101
YDR207C	1590,48	1,08	0,11	0,7448604	0,45635612	0,53970704
YKR081C	1626,39	1,11	0,15	0,74480798	0,45638781	0,53970704
YCL054W-A	161,38	1,18	0,24	0,74088133	0,45876539	0,54241542
YNCE0013W	5,65	1,94	0,96	0,74022909	0,459161	0,54277985
YPL107W	631,95	1,09	0,13	0,7391483	0,45981695	0,54345184
YPR137W	1051,95	1,11	0,15	0,73618733	0,4616167	0,54547516
YBR282W	967,15	1,09	0,13	0,73422221	0,46281332	0,54678516
YJL059W	576,43	0,91	-0,13	-0,7332864	0,46338377	0,547355
YEL057C	147,27	1,17	0,23	0,73252517	0,46384809	0,5477993
YKL182W	10440,8	0,91	-0,14	-0,72862311	0,46623224	0,55040926
YNL312W	1476,01	0,93	-0,11	-0,72861815	0,46623527	0,55040926
YDR138W	1317,27	0,91	-0,13	-0,72729615	0,46704456	0,55125989
YDL053C	1775,35	0,93	-0,11	-0,72571154	0,46801562	0,55230111
YDR527W	1642,56	1,09	0,13	0,72532823	0,46825069	0,55247356
YJR021C	1004,37	1,09	0,12	0,72382685	0,46917204	0,55345551
YER074W-A	1688,49	0,89	-0,16	-0,72352104	0,46935983	0,55356084
YHR089C	4421,62	0,92	-0,13	-0,72339124	0,46943955	0,55356084
YHL015W-A	33,84	0,74	-0,43	-0,72286287	0,46976414	0,55383845
YLR342W	11126,55	0,9	-0,15	-0,72152672	0,47058551	0,55470155
YGR075C	302,03	0,9	-0,16	-0,72127586	0,47073981	0,55477816
YHR163W	2589,3	1,07	0,1	0,72089322	0,47097521	0,55495031
YPR105C	1992,93	1,1	0,13	0,71908821	0,47208657	0,55615433
YDR166C	2044,86	0,92	-0,11	-0,71891219	0,47219502	0,55617662
YPR104C	1193,53	1,09	0,12	0,71624775	0,47383838	0,55800645
YML061C	1113,79	0,92	-0,12	-0,71604077	0,47396617	0,55805115
YPL017C	800,46	1,1	0,14	0,71522438	0,47447041	0,55853898
YIL013C	849,2	0,92	-0,12	-0,71489869	0,47467165	0,55856417
YKR030W	1155,59	0,93	-0,11	-0,71503812	0,47458549	0,55856417
YKL065C	6268,09	1,08	0,11	0,71391426	0,4752802	0,55911612
YPR188C	609,1	1,09	0,12	0,71384876	0,47532071	0,55911612
YDR522C	96,56	1,23	0,3	0,71262921	0,47607523	0,55979166
YLR021W	638,75	0,92	-0,13	-0,71265688	0,47605811	0,55979166
YOL163W	63,52	1,23	0,3	0,7121906	0,47634676	0,56000493
YDL242W	132,57	0,87	-0,21	-0,71162914	0,47669446	0,56030766
YOR210W	1333,26	0,92	-0,11	-0,71120684	0,47695607	0,5605091
YNCE0023W	0,81	0,18	-2,5	-0,71094053	0,47712109	0,56059697
YDR306C	1296,21	0,91	-0,13	-0,71024303	0,47755344	0,56099886
YNCI0002W	0,81	0,18	-2,5	-0,7099342	0,47774494	0,56111771
YGR018C	2,96	2,72	1,44	0,70901999	0,47831207	0,56167762
YJL039C	2316,75	1,09	0,13	0,70705705	0,47953103	0,5630026
YLR290C	1210,8	0,92	-0,13	-0,70690236	0,47962716	0,56300905
YHL018W	93,29	1,19	0,25	0,70647878	0,47989044	0,56321168

YLR343W	1167,08	0,92	-0,11	-0,70515979	0,4807108	0,56406791
YCR024C	933,74	0,91	-0,13	-0,70288748	0,48212588	0,56562152
YNCD0027C	1,04	5,32	2,41	0,70234197	0,48246592	0,56580674
YNCK0004W	1,04	5,32	2,41	0,70234197	0,48246592	0,56580674
YOR255W	5,49	1,83	0,87	0,70149528	0,48299398	0,5663191
YER146W	3,66	2,2	1,13	0,7010592	0,48326607	0,5665312
YJL176C	1914,13	0,93	-0,1	-0,7008172	0,4834171	0,56660133
YDL192W	7170,73	1,07	0,1	0,70039069	0,48368335	0,56680644
YBL008W-A	1,45	3,5	1,81	0,69988839	0,48399701	0,56706704
YLR195C	2488,62	0,93	-0,1	-0,69907817	0,48450318	0,56753643
YOR147W	1571,25	1,08	0,11	0,69895467	0,48458036	0,56753643
YMR170C	969,18	1,09	0,13	0,69852178	0,48485094	0,56774629
YNCD0024C	1,63	4,01	2	0,69817782	0,485066	0,56789106
YOR029W	29,95	1,33	0,41	0,69734493	0,48558696	0,56839385
YJL027C	5,5	1,83	0,87	0,69716374	0,48570033	0,56841945
YOR221C	2037,63	1,07	0,1	0,69699015	0,48580896	0,56843949
YPL086C	1586,44	1,08	0,12	0,69660213	0,48605182	0,56861655
YKR026C	1810,71	0,92	-0,11	-0,69637042	0,48619688	0,56867916
YML025C	2079,26	1,1	0,13	0,69579381	0,48655796	0,56899437
YER153C	322,33	0,9	-0,15	-0,69545248	0,48677178	0,56913727
YDR386W	468,84	1,09	0,13	0,69477485	0,48719641	0,56952655
YER145C-A	10,69	1,55	0,63	0,69440305	0,48742948	0,5696918
YDR308C	890,62	0,93	-0,11	-0,6936723	0,48788774	0,57012014
YIR009W	289,38	0,9	-0,15	-0,69282042	0,48842225	0,5706374
YAL030W	1227,87	0,93	-0,1	-0,69261895	0,48854872	0,57067782
YNCP0018C	5,7	0,53	-0,91	-0,69191491	0,48899076	0,57108679
YHR091C	929,36	1,08	0,11	0,68994658	0,49022778	0,57242387
YDR278C	2,62	0,37	-1,42	-0,68876227	0,49097288	0,57318615
YMR033W	2475,25	1,07	0,1	0,68783545	0,49155641	0,57375957
YDL176W	986,3	1,08	0,11	0,68225119	0,49508013	0,577764
YJL162C	474,6	1,1	0,14	0,68052375	0,49617289	0,5789305
YDR073W	885	1,08	0,11	0,6749792	0,49968898	0,58292356
YER062C	3517,59	1,07	0,09	0,67425607	0,50014853	0,58335011
YNCM0019W	8	1,62	0,69	0,67348269	0,50064026	0,58381402
YDL108W	921,84	1,08	0,11	0,67321392	0,50081121	0,58385041
YNCH0011W	7,46	0,59	-0,77	-0,6731381	0,50085944	0,58385041
YJL133W	1294,06	1,08	0,1	0,67188643	0,501656	0,58466925
YKR092C	2726,66	1,1	0,14	0,67095021	0,50225225	0,58521251
YNL027W	2731,73	1,08	0,11	0,6708587	0,50231055	0,58521251
YLL030C	4,32	1,95	0,96	0,66969042	0,50305516	0,58590625
YPL280W	7,33	1,68	0,75	0,66962849	0,50309465	0,58590625
YJR086W	553,47	0,91	-0,13	-0,66805072	0,50410122	0,58696846
YDL017W	710,9	1,09	0,12	0,66605868	0,50537359	0,58823387
YMR076C	1820,57	0,91	-0,13	-0,66605279	0,50537736	0,58823387
YKL195W	4622,74	0,94	-0,09	-0,66584007	0,50551333	0,5882819
YNCN0002C	2,97	0,4	-1,33	-0,66479483	0,50618173	0,58894941
YEL034W	9340,89	1,09	0,12	0,66403391	0,50666861	0,58907456
YHR153C	80,11	0,84	-0,25	-0,66411033	0,5066197	0,58907456
YNCJ0021C	2,36	2,72	1,44	0,66426567	0,50652029	0,58907456
YPR099C	2,62	2,29	1,19	0,664088	0,50663399	0,58907456

YGL007C-A	12,69	0,67	-0,58	-0,66354323	0,5069827	0,58932944
YCR037C	1256,8	1,07	0,1	0,66153967	0,50826628	0,59071097
YIL044C	1811,15	1,07	0,1	0,66089444	0,50868001	0,59108122
YDR150W	3683,57	1,07	0,09	0,66067746	0,50881918	0,59113236
YPR038W	7,21	1,76	0,81	0,6583709	0,51029983	0,59274169
YIL046W-A	13,23	1,47	0,56	0,65782873	0,51064819	0,59303544
YNL248C	2317,01	0,91	-0,14	-0,65621319	0,51168697	0,59413074
YLR078C	1092,36	1,08	0,11	0,65487513	0,51254816	0,59501946
YGL065C	1545,54	0,94	-0,1	-0,65437261	0,51287177	0,59528391
YNL096C	13653,67	0,93	-0,11	-0,653992	0,51311696	0,59545723
YBR138C	761,69	1,09	0,12	0,65340864	0,51349287	0,59578216
YML017W	1410,28	1,08	0,11	0,65264792	0,51398329	0,59621927
YNR057C	108,82	0,87	-0,21	-0,65252654	0,51406156	0,59621927
YGR215W	614,24	1,09	0,13	0,65051769	0,51535788	0,59761119
YGR120C	592,34	0,92	-0,12	-0,65029067	0,51550448	0,59766962
YCR036W	2352,83	0,93	-0,1	-0,64879046	0,51647382	0,59862127
YNCG0002C	6,07	1,69	0,76	0,64872207	0,51651804	0,59862127
YLR251W	1853,93	1,09	0,13	0,64821812	0,51684389	0,59888719
YCR053W	10849,43	0,93	-0,1	-0,64671693	0,51781516	0,59969818
YLL018C-A	544,08	0,91	-0,14	-0,6469281	0,51767848	0,59969818
YNCK0001W	16,14	0,67	-0,57	-0,64668877	0,51783339	0,59969818
YGR029W	1381,6	1,08	0,11	0,64580707	0,51840431	0,60024745
YNCL0042C	24,03	0,75	-0,42	-0,64481453	0,51904738	0,60088005
YER077C	1119,61	1,08	0,11	0,64385462	0,51966971	0,6014884
YNL086W	225,73	1,12	0,16	0,64362289	0,51982	0,60155027
YNCC0013W	1,72	0,31	-1,67	-0,64314716	0,52012862	0,6017953
YNCB0015W	1,35	3,28	1,71	0,64140938	0,52125674	0,60298825
YMR089C	2927,71	0,94	-0,09	-0,63976359	0,5223263	0,60411301
YHR139C-A	38,72	1,25	0,32	0,63919502	0,52269607	0,60442814
YDR517W	3922,06	0,94	-0,09	-0,63882295	0,52293812	0,60459549
YPR131C	838,52	1,07	0,1	0,63790053	0,52353843	0,60517692
YIR038C	2610,66	1,1	0,14	0,6371456	0,52403001	0,60563245
YMR122C	72,4	0,84	-0,24	-0,63673478	0,52429762	0,60582902
YLR408C	266,5	0,9	-0,15	-0,6354154	0,52515753	0,60652327
YMR072W	6409,24	1,07	0,09	0,63536668	0,5251893	0,60652327
YPL262W	6668,39	0,93	-0,1	-0,63536353	0,52519135	0,60652327
YCL048W	43,47	1,23	0,3	0,63423797	0,52592553	0,60725825
YAL026C	1942,12	1,08	0,11	0,63315531	0,52663222	0,60796123
YDR324C	1631,4	0,92	-0,12	-0,632352	0,52715689	0,60845385
YGL134W	1191,03	1,07	0,1	0,63146225	0,52773832	0,60901179
YBR102C	1475,71	0,93	-0,11	-0,62955968	0,52898271	0,61022109
YJL004C	941,44	1,07	0,1	0,62970346	0,52888862	0,61022109
YOR077W	317	1,1	0,13	0,62846215	0,52970124	0,61093652
YNCD0007C	7,49	1,59	0,66	0,62769497	0,53020378	0,61140261
YDL197C	1836,39	1,07	0,1	0,62683929	0,53076458	0,61193571
YJR105W	5588,92	0,94	-0,09	-0,62668858	0,53086339	0,61193605
YLR292C	2259,8	0,94	-0,09	-0,62589021	0,53138696	0,61242593
YNCO0026W	90,9	0,77	-0,38	-0,62518813	0,53184759	0,61284311
YOL160W	18,71	1,38	0,46	0,62360832	0,53288485	0,61392446
YPR161C	2001,08	1,06	0,09	0,62343387	0,53299945	0,61394263

YHL046C	37,97	1,27	0,34	0,62295047	0,53331707	0,6141946
YOL102C	702,28	0,93	-0,1	-0,62177292	0,53409119	0,6149721
YNR039C	866,16	0,93	-0,11	-0,62147285	0,53428855	0,61508534
YNCC0012C	51,55	1,31	0,39	0,62106373	0,53455769	0,61528115
YBL015W	4828,05	0,9	-0,15	-0,61897496	0,53593285	0,6167497
YLR438C-A	1492,82	1,07	0,09	0,61872103	0,53610015	0,61682796
YLR284C	533,72	0,91	-0,13	-0,61827366	0,53639496	0,61705287
YMR247C	1445	0,93	-0,11	-0,61687148	0,5373195	0,61800199
YMR085W	785,78	0,92	-0,12	-0,61665896	0,5374597	0,61804881
YMR094W	192,88	0,88	-0,18	-0,61618957	0,53776941	0,6182905
YJR155W	368,3	1,1	0,14	0,61428014	0,53903022	0,61962542
YBR027C	2,97	2,04	1,03	0,61374246	0,53938553	0,61991913
YOR266W	604,59	1,08	0,11	0,61342981	0,53959218	0,62004192
YDL088C	2219,8	1,06	0,09	0,61295862	0,5399037	0,62028515
YDR378C	1394,99	1,07	0,09	0,61188642	0,5406129	0,62047743
YDR476C	1538,96	0,94	-0,09	-0,61179958	0,54067037	0,62047743
YKL011C	766,41	1,08	0,11	0,61182522	0,5406534	0,62047743
YLR199C	1374,65	0,94	-0,09	-0,6119105	0,54059697	0,62047743
YNL286W	1199,84	1,08	0,11	0,61246882	0,54022762	0,62047743
YOL161C	1,47	0,34	-1,58	-0,6120418	0,5405101	0,62047743
YER163C	1068,08	1,07	0,1	0,61087372	0,54128319	0,62106597
YLR427W	1854,57	1,07	0,09	0,61071031	0,54139138	0,6210754
YIL134C-A	9,52	1,56	0,64	0,61040013	0,54159678	0,62119631
YPL133C	797,34	0,92	-0,12	-0,60890165	0,54258963	0,62222019
YDR484W	1231,16	0,93	-0,1	-0,60851986	0,54284274	0,62239555
YAL020C	735,55	0,92	-0,12	-0,60722458	0,54370188	0,62326556
YML100W-A	37,95	0,81	-0,3	-0,60544141	0,54488575	0,62450743
YIL029C	438,99	0,92	-0,13	-0,60505129	0,54514492	0,62468922
YNCG0032W	2,45	0,41	-1,3	-0,60421762	0,54569897	0,62520878
YOL127W	19586,7	0,93	-0,1	-0,60367434	0,54606018	0,62550725
YDR190C	4063,41	1,06	0,09	0,60208506	0,54711752	0,6265923
YGR281W	3129,9	1,06	0,09	0,60194741	0,54720915	0,6265923
YDL202W	903,07	0,93	-0,1	-0,60108197	0,54778539	0,62713654
YDR109C	705,65	1,08	0,11	0,60022549	0,54835597	0,62767409
YHL039W	1208,03	0,91	-0,13	-0,59989492	0,54857626	0,62781057
YJL114W	24,98	0,77	-0,38	-0,59610397	0,55110577	0,63058925
YFL064C	18,04	1,34	0,42	0,59412504	0,55242848	0,63198633
YGR191W	2635,53	1,06	0,09	0,59356171	0,5528053	0,63230096
YKL156W	8504,79	1,08	0,11	0,59329054	0,55298673	0,63239204
YAL064W	4,23	1,9	0,92	0,59210141	0,55378268	0,63313725
YDR014W-A	32,3	1,25	0,32	0,59201251	0,55384221	0,63313725
YKL205W	1272,13	0,92	-0,13	-0,58987617	0,55527367	0,63465686
YIL079C	382,12	1,09	0,13	0,58941708	0,55558152	0,63489191
YDL246C	7,13	0,63	-0,66	-0,58856638	0,55615219	0,63542715
YCL002C	694,22	0,93	-0,1	-0,58766944	0,55675419	0,63591702
YEL002C	5553,26	0,95	-0,08	-0,58752857	0,55684877	0,63591702
YPL113C	442,27	1,1	0,14	0,58747006	0,55688805	0,63591702
YJL077W-B	110,78	1,16	0,22	0,58691259	0,55726242	0,63622756
YMR321C	31	0,78	-0,36	-0,58659296	0,55747711	0,63635572
YMR059W	173,2	0,9	-0,16	-0,58529723	0,55834788	0,63716539

YOR138C	1725,53	0,94	-0,09	-0,58523225	0,55839156	0,63716539
YHR125W	1,57	0,36	-1,48	-0,58411974	0,55913975	0,63790196
YER078W-A	19,14	1,33	0,41	0,5834286	0,55960481	0,63808096
YNCI0004W	0,85	4,36	2,13	0,58342973	0,55960405	0,63808096
YPL281C	1,56	0,36	-1,48	-0,58343953	0,55959746	0,63808096
YOL027C	3280,86	1,06	0,08	0,58269526	0,56009847	0,63852664
YNCG0022W	0,85	4,35	2,12	0,58234413	0,56033491	0,63867898
YPL068C	286,55	0,92	-0,13	-0,58073343	0,56142013	0,63979854
YGR274C	1464,81	1,07	0,1	0,58011185	0,5618392	0,64015867
YCR060W	423,49	0,92	-0,12	-0,57784837	0,5633665	0,64178116
YML022W	1700,26	0,92	-0,12	-0,57744296	0,56364026	0,6419753
YIR037W	4522,21	0,94	-0,09	-0,57667639	0,56415808	0,6424473
YOL080C	413,48	1,1	0,14	0,575936	0,56465844	0,64289924
YNCF0012C	1,56	0,36	-1,48	-0,57512192	0,56520884	0,64340798
YLR157W-E	3,28	1,94	0,96	0,57448425	0,56564016	0,64366306
YOL103W	2497,26	1,06	0,08	0,57456534	0,5655853	0,64366306
YHR170W	3500,67	1,08	0,12	0,57432299	0,56574925	0,6436693
YGL184C	1088,66	1,56	0,64	0,57358531	0,56624845	0,64411928
YLR017W	1825,86	1,06	0,08	0,5721109	0,56724684	0,64513684
YAL018C	4,54	1,85	0,88	0,570304	0,56847153	0,6464048
YMR100W	1103,32	1,09	0,12	0,5700056	0,5686739	0,6464048
YMR217W	9353,62	0,94	-0,09	-0,57006157	0,56863594	0,6464048
YOR091W	2026,8	1,06	0,09	0,56859095	0,56963376	0,64737741
YDR196C	1283,24	1,07	0,1	0,56830469	0,56982809	0,64747981
YNCG0009C	0,64	0,22	-2,18	-0,56782919	0,57015096	0,64772819
YBR289W	1509,72	1,06	0,09	0,56708273	0,57065798	0,64806716
YJL104W	1585,67	1,06	0,08	0,56719328	0,57058287	0,64806716
YNL184C	1,35	3,27	1,71	0,56654264	0,57102495	0,64836538
YMR009W	1116,21	0,94	-0,1	-0,56421958	0,5726047	0,65004028
YGL008C	38489,08	1,09	0,12	0,56378074	0,57290336	0,65026049
YDL116W	2020,93	1,06	0,09	0,56261199	0,57369913	0,65097158
YNL247W	6983,09	0,94	-0,09	-0,5625528	0,57373944	0,65097158
YER048W-A	1227,61	1,06	0,09	0,55953206	0,57579866	0,65318869
YIL102C	27,82	0,78	-0,35	-0,55916869	0,5760466	0,65335065
YOR283W	1216,42	0,93	-0,1	-0,55597428	0,57822844	0,65570556
YMR141C	282,65	1,1	0,13	0,55545679	0,57858226	0,65598704
YPL077C	424,73	0,93	-0,11	-0,5541762	0,57945826	0,65686035
YPR116W	240,27	1,09	0,13	0,55354047	0,57989338	0,65723366
YGL026C	7067,42	0,95	-0,07	-0,55332717	0,5800394	0,65727923
YJL077C	114,73	1,13	0,18	0,55115334	0,58152856	0,65882003
YMR287C	868,53	1,07	0,09	0,55103272	0,58161124	0,65882003
YER105C	3074,49	1,06	0,09	0,55002979	0,58229894	0,65947877
YFL046W	1093,74	0,94	-0,09	-0,54809767	0,58362484	0,66067655
YGR097W	3462,8	1,08	0,11	0,54802373	0,58367561	0,66067655
YKL198C	1270,4	0,94	-0,09	-0,54815676	0,58358427	0,66067655
YOR186W	130,79	1,2	0,26	0,54732267	0,58415707	0,66110106
YNR023W	1296,57	1,06	0,08	0,54647762	0,58473767	0,66163759
YBL021C	395,37	0,92	-0,13	-0,54599731	0,58506779	0,66189057
YKL107W	113,21	1,16	0,22	0,54577692	0,58521929	0,66194141
YGL112C	2403,17	0,95	-0,08	-0,54522624	0,58559793	0,6622491

YGL189C	34862,33	0,95	-0,07	-0,5433319	0,5869013	0,6634837
YHR209W	972,02	0,94	-0,09	-0,54332908	0,58690324	0,6634837
YNL125C	1465,74	0,92	-0,12	-0,54311068	0,5870536	0,66353293
YIL153W	1365,23	0,94	-0,08	-0,54200464	0,58781531	0,66416449
YOL063C	1330,05	0,94	-0,08	-0,54198883	0,5878262	0,66416449
YLR138W	3246,75	1,07	0,1	0,54129718	0,58830276	0,66458206
YPL198W	4136,23	0,95	-0,07	-0,54108779	0,58844708	0,66462422
YMR105W-A	36,05	0,81	-0,3	-0,53986279	0,58929166	0,66545715
YGR172C	1476,34	1,06	0,08	0,53927902	0,58969435	0,66579085
YEL067C	151,85	1,12	0,17	0,53859757	0,59016456	0,66620066
YLR341W	95,76	1,13	0,18	0,53750252	0,59092055	0,66693285
YLR150W	40257,53	0,94	-0,09	-0,53691922	0,59132342	0,66716306
YPL129W	3663,88	0,95	-0,07	-0,5368962	0,59133932	0,66716306
YCR076C	755,48	0,91	-0,13	-0,53636425	0,59170683	0,66745648
YDL030W	732,24	0,94	-0,09	-0,53596952	0,59197962	0,66764295
YJR148W	5700,29	1,06	0,08	0,53457393	0,59294451	0,66860979
YDL181W	1776,81	1,08	0,12	0,53271396	0,5942316	0,66993951
YCR081W	901,95	1,07	0,09	0,53224188	0,59455848	0,67018641
YOL130W	2129,13	0,94	-0,08	-0,5317104	0,59492658	0,67047967
YBR291C	438,79	0,92	-0,12	-0,53145197	0,59510562	0,67055979
YDL203C	1138,53	1,06	0,08	0,52858982	0,59709003	0,6726314
YHL011C	2873,49	0,94	-0,09	-0,52848799	0,59716068	0,6726314
YBL097W	450,47	1,08	0,12	0,52488025	0,59966644	0,67521898
YLL067C	71,6	1,15	0,2	0,52486747	0,59967533	0,67521898
YHR004C	1773,14	1,06	0,08	0,52429107	0,60007611	0,6755478
YOR093C	1041,8	0,94	-0,08	-0,5228083	0,60110767	0,67658649
YMR112C	456,34	0,93	-0,11	-0,52245668	0,6013524	0,67673933
YDL101C	820,77	0,94	-0,09	-0,52127115	0,6021779	0,67754557
YDR243C	529,27	1,07	0,1	0,52038941	0,60279219	0,67811393
YNCF0001C	47,79	0,78	-0,36	-0,51954573	0,60338023	0,67865254
YOR161C	1620,08	0,92	-0,12	-0,51829402	0,60425315	0,67951132
YGR218W	6520,17	1,06	0,08	0,51796469	0,60448291	0,67958876
YLR415C	30,21	0,82	-0,29	-0,51788171	0,6045408	0,67958876
YJR160C	45,76	0,85	-0,24	-0,51640911	0,60556871	0,68058667
YLR312W-A	1107,93	0,93	-0,1	-0,51629607	0,60564764	0,68058667
YFR023W	34,53	1,23	0,3	0,51593944	0,60589671	0,68074342
YPL013C	1118,34	0,93	-0,11	-0,51505264	0,60651624	0,68131625
YDR420W	1512,28	0,94	-0,09	-0,51480235	0,60669114	0,68138951
YPR085C	830,87	1,06	0,09	0,51200128	0,60865011	0,68346611
YAR010C	64,18	1,18	0,24	0,51122745	0,6091918	0,68382715
YCL001W-B	51,88	0,85	-0,24	-0,51136655	0,60909441	0,68382715
YML107C	446,5	0,93	-0,1	-0,50947128	0,61042192	0,68496044
YNR038W	904,51	0,93	-0,11	-0,50960558	0,61032781	0,68496044
YER004W	2016,21	1,06	0,08	0,50912736	0,61066296	0,68510591
YLR189C	2169,14	0,95	-0,08	-0,50897159	0,61077214	0,68510591
YBR160W	1318,85	0,95	-0,08	-0,50837574	0,61118986	0,6854507
YLR347C	2216,22	0,94	-0,1	-0,50612513	0,61276881	0,68709745
YMR142C	22270,86	1,05	0,07	0,5055061	0,61320341	0,68746067
YOR183W	6,72	0,67	-0,58	-0,50529376	0,61335252	0,68750377
YBL104C	819,85	1,07	0,09	0,50281713	0,61509285	0,68920577

YIL077C	1757,42	1,07	0,1	0,50293916	0,61500705	0,68920577
YLR174W	287,14	1,1	0,14	0,50107453	0,61631867	0,69045475
YCL040W	43987,06	0,94	-0,09	-0,50052039	0,6167087	0,69076712
YAL027W	829	1,06	0,09	0,49959715	0,61735877	0,69123487
YFL024C	1761,42	1,06	0,08	0,49945324	0,61746012	0,69123487
YPL231W	14627,84	0,94	-0,09	-0,4997407	0,61725767	0,69123487
YNL330C	2124,51	0,95	-0,08	-0,4980555	0,61844493	0,69221259
YAR002W	2648,1	0,95	-0,07	-0,49762841	0,61874597	0,69242479
YLR200W	1274,28	0,94	-0,08	-0,49708935	0,61912605	0,69272532
YMR253C	1252,37	0,94	-0,09	-0,49667972	0,61941493	0,69279898
YMR272W-B	5,72	1,54	0,62	0,49682895	0,61930968	0,69279898
YNCD0003W	3,98	1,71	0,77	0,49608128	0,61983707	0,69305207
YPL149W	710,19	1,07	0,1	0,49604262	0,61986434	0,69305207
YPL079W	13166,73	0,95	-0,07	-0,49492611	0,62065228	0,69380816
YFL004W	6939,1	1,05	0,08	0,49180366	0,62285815	0,69613289
YNL197C	2243,19	1,06	0,08	0,49166524	0,62295601	0,69613289
YCR073W-A	1788,72	0,95	-0,08	-0,4906424	0,62367939	0,69671417
YDR205W	682,32	0,94	-0,09	-0,49061256	0,6237005	0,69671417
YLR307W	28,22	0,82	-0,28	-0,48946629	0,62451161	0,69744695
YOR076C	979,2	1,06	0,08	0,48936822	0,62458103	0,69744695
YOR339C	53,15	0,86	-0,22	-0,48816565	0,62543251	0,69827224
YNCN0013W	123,76	1,16	0,21	0,48715298	0,62614993	0,69882202
YOR310C	6978,54	1,07	0,1	0,48725386	0,62607844	0,69882202
YDR450W	17429,58	0,96	-0,07	-0,48610374	0,62689362	0,69952634
YPL055C	1273,46	0,95	-0,08	-0,48465489	0,62792117	0,70054711
YNL054W	1565,06	0,95	-0,08	-0,48362618	0,62865119	0,70123563
YGL144C	389,37	0,93	-0,1	-0,48301928	0,62908205	0,70159025
YDR194C	3315,11	1,05	0,07	0,48274304	0,6292782	0,70168304
YIL020C	719,94	1,08	0,11	0,48249681	0,62945307	0,70175206
YHR064C	13188,74	1,07	0,1	0,48213347	0,62971113	0,70191379
YDL105W	449,77	0,94	-0,09	-0,48189794	0,62987845	0,70195955
YHL042W	142,06	0,9	-0,15	-0,48175756	0,62997818	0,70195955
YJR050W	791,21	1,06	0,08	0,48022299	0,63106884	0,70304872
YHR113W	2791,96	0,95	-0,07	-0,4792935	0,63172984	0,70365892
YBL098W	1159,93	0,8	-0,33	-0,47878037	0,63209488	0,7039393
YEL076C	4,65	1,59	0,67	0,47794412	0,63268998	0,70434949
YGR164W	1,4	0,42	-1,26	-0,47797761	0,63266614	0,70434949
YHR114W	2944,14	1,05	0,07	0,47564704	0,63432586	0,70604412
YIL101C	1524,07	0,91	-0,13	-0,47448605	0,63515335	0,70667737
YJR078W	159,47	0,88	-0,19	-0,47437002	0,63523607	0,70667737
YOR265W	843,54	1,06	0,08	0,47464383	0,63504086	0,70667737
YKL208W	345,51	0,92	-0,12	-0,47417869	0,63537249	0,70670257
YDR510W	5835,87	1,05	0,07	0,47201506	0,63691604	0,70816582
YHR107C	2515,38	1,05	0,07	0,47205476	0,6368877	0,70816582
YDR049W	1294,85	1,05	0,08	0,47130573	0,63742243	0,70852978
YOR179C	554,83	0,94	-0,1	-0,471237	0,6374715	0,70852978
YNL269W	10,96	0,74	-0,43	-0,47044849	0,63803463	0,70899265
YOL044W	447,15	0,93	-0,1	-0,47033427	0,63811622	0,70899265
YPL162C	506,78	0,94	-0,09	-0,46986219	0,63845348	0,70924053
YJR035W	1358,9	1,05	0,07	0,46940363	0,63878115	0,70947765

YDR236C	874,68	0,94	-0,08	-0,46874071	0,63925499	0,70987701
YJR151W-A	9,14	1,37	0,46	0,46791986	0,6398419	0,71040177
YDL057W	556,77	1,06	0,08	0,46741113	0,64020576	0,71067873
YIL114C	733,23	1,07	0,1	0,46689989	0,6405715	0,71095769
YFL038C	8872,86	1,06	0,08	0,46538986	0,64165229	0,71203002
YJL007C	11,55	0,75	-0,41	-0,46504155	0,6419017	0,71217957
YDL207W	1885,05	1,05	0,07	0,46462668	0,64219882	0,71225479
YMR015C	1026,13	1,06	0,08	0,46473955	0,64211798	0,71225479
YNCG0004W	0,64	0,22	-2,17	-0,46413402	0,64255173	0,71239181
YNCG0006C	0,64	0,22	-2,17	-0,46413402	0,64255173	0,71239181
YOR253W	1030,9	1,05	0,08	0,46382536	0,64277287	0,71250983
YBL085W	2889,27	1,06	0,08	0,46144424	0,64447992	0,71427462
YHR079C	1355,61	0,95	-0,08	-0,46043919	0,64520101	0,71494625
YNCG0015C	46,2	0,86	-0,21	-0,45988742	0,64559703	0,71525749
YBR107C	607,4	0,95	-0,08	-0,45939633	0,64594959	0,71552047
YOR128C	9903,9	1,05	0,07	0,45789153	0,64703037	0,71658988
YDR007W	1132,94	0,94	-0,08	-0,45522263	0,64894908	0,71858676
YML056C	5251,1	0,95	-0,07	-0,45455908	0,64942648	0,71898722
YBL108C-A	10,16	1,33	0,41	0,45147479	0,65164739	0,72131746
YOR044W	700,24	0,94	-0,08	-0,45103552	0,65196395	0,72153929
YNCG0042C	1,51	2,19	1,13	0,44951652	0,65305909	0,72249387
YNCL0035C	1,51	2,19	1,13	0,44951652	0,65305909	0,72249387
YGL060W	1022,66	0,95	-0,07	-0,44921969	0,65327319	0,72260204
YOR166C	998,84	0,95	-0,08	-0,44799877	0,65415409	0,7234476
YFR027W	272,4	0,93	-0,1	-0,44757828	0,65445758	0,72365441
YOR236W	508,15	1,06	0,08	0,44498718	0,65632902	0,72559456
YGR121C	1237,61	0,95	-0,08	-0,44388763	0,65712383	0,7261764
YNL116W	821,89	0,95	-0,07	-0,44405503	0,6570028	0,7261764
YOR163W	1833,5	0,94	-0,08	-0,44377397	0,65720601	0,7261764
YBR132C	1571,22	0,94	-0,08	-0,44309599	0,65769631	0,72658891
YDR235W	1363,97	1,05	0,07	0,44216682	0,65836849	0,72708007
YJR007W	5499,02	1,05	0,07	0,44215785	0,65837499	0,72708007
YHL016C	339,13	1,08	0,11	0,4413987	0,65892438	0,72755746
YDR347W	1951,5	1,05	0,07	0,43941399	0,66036159	0,72901479
YIR032C	98,27	0,88	-0,18	-0,43729842	0,66189494	0,73057772
YEL031W	5172,56	1,08	0,1	0,43565727	0,66308541	0,73163172
YER036C	6604,83	1,07	0,09	0,43569207	0,66306016	0,73163172
YNL279W	370,76	0,94	-0,09	-0,43489385	0,66363948	0,73211302
YNL140C	3,89	0,62	-0,7	-0,4346145	0,66384227	0,73220671
YKL207W	3267,19	0,96	-0,06	-0,43409362	0,66422046	0,73249379
YDR223W	315,6	0,93	-0,1	-0,43336484	0,66474974	0,73281728
YJL183W	994,57	1,06	0,09	0,43350057	0,66465115	0,73281728
YLR351C	2983,05	1,04	0,06	0,43294774	0,66505274	0,73302122
YPR075C	2641,1	0,95	-0,07	-0,4324273	0,66543088	0,7333079
YKL090W	524,92	1,07	0,1	0,43094164	0,66651081	0,73436771
YNCP0023W	1,96	1,98	0,98	0,43002925	0,66717436	0,73496846
YDR304C	6926,1	0,96	-0,06	-0,42830096	0,66843202	0,73622335
YAL041W	2207,41	1,06	0,08	0,4251307	0,6707414	0,73863599
YIL030C	2176,49	0,94	-0,09	-0,42234232	0,67277517	0,7407443
YOR305W	729,71	1,05	0,07	0,42096849	0,67377809	0,74171708

YMR032W	1039,75	0,95	-0,07	-0,42060241	0,67404544	0,74187992
YIL014C-A	271,97	1,09	0,12	0,42019426	0,67434355	0,74207655
YDR542W	5,13	1,45	0,54	0,41908528	0,67515381	0,7428366
YHR191C	280,98	0,94	-0,09	-0,4183934	0,67565951	0,74326136
YPR061C	372,49	0,93	-0,1	-0,41732151	0,67644326	0,74399177
YHR130C	52,55	0,87	-0,21	-0,41676289	0,67685185	0,74430938
YNCE0003W	3,7	1,56	0,65	0,41546299	0,677803	0,74522341
YGL147C	17656,96	0,96	-0,05	-0,41527284	0,67794218	0,74524453
YNCD0005C	2,68	1,82	0,87	0,41489132	0,67822146	0,74541962
YLR446W	742,32	1,05	0,07	0,41439668	0,67858362	0,74555385
YNCE0021C	11,85	0,77	-0,38	-0,41440798	0,67857534	0,74555385
YHL009W-B	29,25	1,18	0,24	0,41347313	0,67926001	0,74616501
YFL048C	2181,84	0,96	-0,06	-0,41268974	0,67983395	0,74666345
YDL107W	872,29	0,95	-0,07	-0,41170323	0,68055696	0,74732541
YLR047C	789,61	0,95	-0,07	-0,41135371	0,68081319	0,74747464
YNL262W	2846,16	1,05	0,08	0,41113188	0,68097583	0,7475211
YOR037W	1247,93	0,96	-0,06	-0,40912925	0,68244481	0,74900127
YJL221C	4,77	1,5	0,59	0,40781186	0,6834118	0,74987386
YNCD0015C	0,48	0,29	-1,76	-0,40771723	0,68348129	0,74987386
YGR055W	3091,49	0,85	-0,23	-0,40689414	0,68408574	0,75040449
YNCO0009W	0,48	0,3	-1,76	-0,40645716	0,68440673	0,75062404
YAL063C-A	16,97	1,26	0,33	0,40577198	0,68491015	0,75104358
YDR439W	688,68	0,95	-0,08	-0,40528037	0,68527143	0,75130712
YEL014C	68,53	0,9	-0,16	-0,405061	0,68543267	0,75135129
YNR067C	3550,92	0,94	-0,09	-0,40282594	0,68707628	0,75302008
YIR003W	3011,49	1,04	0,06	0,40240664	0,68738478	0,75322531
YPL121C	7,66	1,36	0,44	0,40204049	0,68765423	0,75338767
YDR530C	1636,12	1,06	0,09	0,40092232	0,68847732	0,75415643
YOR069W	1595,85	1,04	0,06	0,40041172	0,68885329	0,75443523
YMR315W-A	32,37	1,17	0,22	0,39911642	0,68980742	0,75534703
YOL001W	972,79	1,05	0,07	0,3988002	0,69004043	0,755469
YHR152W	1187,85	0,95	-0,07	-0,39749048	0,69100581	0,75625935
YMR047C	3430,55	1,05	0,08	0,39761914	0,69091095	0,75625935
YPL255W	641,05	0,95	-0,07	-0,39597104	0,69212639	0,75735232
YNCP0017C	2,28	1,83	0,88	0,39521038	0,69268763	0,75783297
YOL084W	352,39	0,93	-0,1	-0,39487157	0,69293767	0,75797302
YJL124C	2990,98	1,04	0,05	0,39186059	0,69516122	0,76027139
YDR338C	788,9	0,96	-0,07	-0,39093754	0,69584341	0,76063033
YEL022W	2388,3	0,95	-0,08	-0,39075384	0,6959792	0,76063033
YGR195W	1219,48	0,96	-0,06	-0,39078451	0,69595652	0,76063033
YOR393W	1,15	0,47	-1,09	-0,39080998	0,6959377	0,76063033
YGR090W	2055,45	1,07	0,09	0,38972999	0,69673622	0,76132373
YAL063C	415,08	1,06	0,09	0,38696495	0,69878216	0,7632908
YBR197C	532,16	1,05	0,07	0,3870166	0,69874392	0,7632908
YLR256W-A	647,59	0,95	-0,07	-0,38636863	0,69922368	0,7636388
YNCJ0001C	1,01	2,2	1,14	0,3856911	0,69972545	0,76405247
YBR043C	1237,81	0,96	-0,06	-0,38458644	0,70054385	0,76481166
YMR103C	797,32	0,96	-0,07	-0,38212697	0,70236719	0,76666754
YNCH0006C	1,77	1,76	0,81	0,38190457	0,70253215	0,76671288
YDR102C	32,34	1,17	0,23	0,38166547	0,70270952	0,76677174

YFR012W-A	13,65	1,24	0,31	0,37993108	0,70399658	0,76804122
YLR396C	982,22	1,05	0,07	0,37944636	0,70435643	0,76829888
YGL221C	2685,43	1,04	0,06	0,37736476	0,70590255	0,76985019
YNCN0016C	3,35	1,64	0,71	0,3766074	0,70646538	0,77032876
YLR371W	2049,95	1,04	0,06	0,37494527	0,70770117	0,77154084
YBL067C	1198,91	0,96	-0,06	-0,37396148	0,70843297	0,77198237
YNL299W	299,49	1,07	0,1	0,37396288	0,70843194	0,77198237
YOL089C	875,86	0,96	-0,06	-0,37389963	0,70847899	0,77198237
YPL119C	506,27	1,06	0,08	0,37278364	0,70930948	0,77275175
YNR037C	832,43	0,95	-0,08	-0,37218048	0,70975848	0,77310533
YLR435W	702,86	0,95	-0,08	-0,37113929	0,71053379	0,77378968
YNCN0011W	2,89	0,64	-0,64	-0,37100222	0,71063588	0,77378968
YLR451W	1258,15	0,96	-0,06	-0,37027789	0,71117545	0,77424148
YKL022C	1339,14	1,04	0,06	0,3699425	0,71142533	0,77437781
YDR493W	525,05	0,94	-0,08	-0,36893646	0,71217509	0,7750581
YBR273C	2322,59	1,04	0,05	0,36771597	0,71308503	0,77591245
YPR128C	909,81	1,04	0,06	0,36673875	0,7138139	0,77656952
YHR062C	624,86	1,05	0,07	0,36632582	0,71412197	0,77676863
YDR093W	3033,41	1,05	0,06	0,3640209	0,7158424	0,77806165
YFL003C	163,91	1,07	0,1	0,36395454	0,71589196	0,77806165
YNCB0001W	0,49	0,29	-1,77	-0,36389428	0,71593696	0,77806165
YNCE0029C	0,49	0,29	-1,77	-0,36389428	0,71593696	0,77806165
YPR016C	2882,48	1,04	0,06	0,36433159	0,71561041	0,77806165
YKL010C	2665,34	1,04	0,05	0,36313743	0,71650223	0,77853976
YAL068C	0,48	0,3	-1,76	-0,36150351	0,71772308	0,77895362
YGR273C	5,69	1,38	0,46	0,36240193	0,7170517	0,77895362
YLR151C	774,07	0,95	-0,07	-0,36187025	0,71744899	0,77895362
YNCN0009W	0,48	0,3	-1,76	-0,36150351	0,71772308	0,77895362
YNCO0018W	0,48	0,3	-1,76	-0,36150351	0,71772308	0,77895362
YOR092W	1285,04	1,04	0,06	0,36145295	0,71776087	0,77895362
YPL125W	2029,76	0,96	-0,06	-0,36184381	0,71746875	0,77895362
YER039C	73,75	0,91	-0,14	-0,36008008	0,71878725	0,77985544
YJR102C	697,17	1,05	0,07	0,36000562	0,71884293	0,77985544
YLR261C	7,46	1,32	0,4	0,35953156	0,71919747	0,78010384
YNCI0008C	1,24	0,5	-0,99	-0,35932387	0,71935282	0,78013613
YDR461C-A	503	1,07	0,1	0,35617817	0,72170714	0,78255275
YPR110C	3869,12	0,96	-0,06	-0,35424826	0,72315284	0,7839835
YDR344C	87,96	1,1	0,14	0,35406594	0,72328946	0,78399479
YOR104W	402,62	1,05	0,08	0,35298514	0,72409958	0,78473598
YBR093C	4196,93	1,04	0,05	0,3521836	0,72470058	0,7852503
YIL173W	44,7	0,88	-0,18	-0,35084085	0,72570775	0,78620449
YLR130C	2176,52	0,97	-0,05	-0,34919454	0,72694326	0,78740568
YHR038W	692,11	1,05	0,06	0,34838309	0,7275525	0,78792819
YAL009W	598,06	1,04	0,06	0,34751015	0,7282081	0,78840746
YOL017W	532,19	0,95	-0,07	-0,34745585	0,72824888	0,78840746
YGL097W	2230,2	1,03	0,05	0,34672352	0,72879904	0,78861372
YPR029C	4055,29	1,04	0,05	0,3466953	0,72882025	0,78861372
YPR158W-B	249,95	0,95	-0,08	-0,34694075	0,72863583	0,78861372
YOL090W	1452,74	1,06	0,08	0,34579107	0,72949974	0,78921149
YNCK0014W	2,07	0,6	-0,74	-0,34483067	0,73022167	0,78985496

YGR030C	275,22	1,06	0,08	0,34434414	0,73058749	0,79011308
YCL033C	616,24	1,06	0,08	0,3438222	0,73098001	0,79039997
YIL154C	3197,47	0,96	-0,06	-0,34228804	0,73213413	0,79151013
YMR172W	1046,02	1,04	0,05	0,34203585	0,73232392	0,79157755
YDL113C	2052,32	0,97	-0,05	-0,34161874	0,73263783	0,79177909
YNCG0013W	2,07	0,6	-0,73	-0,34105685	0,73306079	0,79209838
YOL121C	6001,65	0,96	-0,06	-0,3406391	0,73337529	0,7923004
YHL014C	582,97	0,96	-0,06	-0,3389109	0,73467686	0,79356853
YJR128W	21,03	0,85	-0,24	-0,33811448	0,73527692	0,79394059
YNL314W	431,38	0,96	-0,06	-0,33825594	0,73517032	0,79394059
YKR021W	1678,26	0,97	-0,05	-0,3370343	0,73609104	0,79468154
YDR286C	224,08	0,94	-0,09	-0,33592827	0,73692496	0,79537288
YOL068C	2207,91	1,04	0,05	0,33584535	0,73698749	0,79537288
YDR222W	921,85	0,96	-0,05	-0,33503908	0,7375956	0,7958909
YDL027C	2442,04	1,04	0,05	0,33380118	0,73852959	0,79609398
YDR332W	780,24	0,96	-0,06	-0,33425371	0,73818812	0,79609398
YHL030W	2047,18	1,05	0,07	0,3337705	0,73855274	0,79609398
YLR073C	315,5	1,06	0,08	0,33384566	0,73849602	0,79609398
YNCD0026W	1,01	2,18	1,12	0,33440121	0,73807682	0,79609398
YOR394C-A	10,95	0,8	-0,32	-0,33402917	0,73835755	0,79609398
YJL184W	543,09	0,96	-0,06	-0,33180029	0,74004006	0,79755879
YDR333C	1839,53	1,04	0,05	0,33042046	0,74108228	0,79792503
YNCD0028W	0,6	2,6	1,38	0,33017106	0,74127072	0,79792503
YNCF0011C	0,6	2,6	1,38	0,33017106	0,74127072	0,79792503
YNCG0029C	1,15	0,47	-1,09	-0,33016005	0,74127903	0,79792503
YNCJ0005C	0,6	2,6	1,38	0,33017106	0,74127072	0,79792503
YNCK0012C	0,6	2,6	1,38	0,33017106	0,74127072	0,79792503
YNCM0024W	0,6	2,6	1,38	0,33017106	0,74127072	0,79792503
YDL093W	1262,28	1,04	0,05	0,32908082	0,7420946	0,79811435
YLR160C	29,71	1,14	0,19	0,32952463	0,74175918	0,79811435
YNCC0005W	0,6	2,59	1,37	0,32907724	0,74209731	0,79811435
YNCD0012W	0,6	2,59	1,37	0,32907724	0,74209731	0,79811435
YNCL0044W	0,6	2,59	1,37	0,32907724	0,74209731	0,79811435
YIL094C	4953,08	1,03	0,05	0,32850443	0,7425303	0,79844179
YBR271W	461,01	0,95	-0,07	-0,32828619	0,74269529	0,79848098
YOR017W	1194,53	1,04	0,05	0,32798421	0,7429236	0,79858824
YGL175C	200,17	0,95	-0,08	-0,32665789	0,74392665	0,79938979
YPL181W	2440,76	1,03	0,05	0,32678185	0,74383289	0,79938979
YNCC0006C	339,59	0,94	-0,09	-0,32645732	0,74407838	0,79941454
YNL230C	682,02	1,04	0,05	0,32576295	0,74460371	0,79984061
YIL057C	38,57	1,18	0,24	0,32516301	0,7450577	0,80018991
YDR322C-A	1187,61	1,04	0,06	0,3242433	0,74575384	0,80039204
YMR036C	545,5	1,04	0,06	0,32413296	0,74583737	0,80039204
YMR131C	4034,6	1,04	0,06	0,32435891	0,74566632	0,80039204
YMR323W	4,39	0,72	-0,48	-0,32406326	0,74589014	0,80039204
YNCN0014W	53,92	1,11	0,15	0,32421477	0,74577544	0,80039204
YLR383W	1087,94	0,96	-0,06	-0,32192516	0,74750939	0,80199108
YCR057C	1610,75	1,05	0,07	0,32082366	0,74834403	0,80268757
YMR192W	1093,86	0,96	-0,05	-0,32072738	0,748417	0,80268757
YPR125W	1193,64	1,04	0,05	0,31978108	0,74913429	0,80331818

YOR358W	658,01	0,95	-0,07	-0,31960856	0,74926509	0,80331977
YER030W	2427,07	0,96	-0,05	-0,31789954	0,75056114	0,80457046
YPR006C	717,56	1,05	0,07	0,31507325	0,75270603	0,80673048
YPL119C-A	74,44	1,09	0,12	0,31401339	0,75351087	0,80745377
YOR270C	11045,85	0,97	-0,05	-0,31322932	0,75410644	0,80795261
YFR046C	587,65	0,96	-0,06	-0,31299817	0,75428206	0,8080014
YMR234W	1090,27	1,04	0,05	0,31242014	0,75472125	0,80833248
YML068W	842,68	0,97	-0,05	-0,31177669	0,75521024	0,80871677
YIL110W	851,93	0,96	-0,06	-0,31129371	0,75557735	0,80897044
YDR320C	2213,33	0,97	-0,04	-0,31054214	0,75614872	0,80944267
YNL251C	3358,81	1,03	0,04	0,30968669	0,75679923	0,80999945
YDR066C	294,26	0,96	-0,07	-0,30927345	0,75711353	0,81019625
YOL159C-A	185,44	0,95	-0,08	-0,30726076	0,7586449	0,81169517
YML011C	440,85	1,04	0,06	0,30657566	0,75916638	0,81197341
YNL199C	1964,88	1,03	0,05	0,30664686	0,75911218	0,81197341
YOL125W	717,46	0,96	-0,05	-0,30501026	0,76035834	0,81310828
YPL092W	1633,19	0,96	-0,05	-0,30400248	0,761126	0,81378911
YOR172W	1060,02	0,97	-0,05	-0,30342103	0,76156902	0,81412266
YER149C	715,42	1,04	0,05	0,30306743	0,76183847	0,81427057
YKL113C	1059,07	1,03	0,05	0,30284375	0,76200894	0,81431267
YLR454W	4002,86	1,03	0,04	0,30222758	0,76247858	0,81467439
YDR515W	611,49	0,96	-0,06	-0,3018228	0,76278715	0,81486394
YDR208W	2387,18	0,97	-0,04	-0,30131631	0,7631733	0,81513627
YPL228W	1745,31	0,97	-0,05	-0,30085821	0,76352262	0,81536918
YKL059C	1206,25	0,97	-0,05	-0,30050869	0,76378917	0,81551363
YKR084C	2943,85	0,97	-0,05	-0,30023678	0,76399655	0,81559487
YGR284C	3667,14	0,97	-0,04	-0,29868424	0,76518098	0,81671895
YNL048W	1407,23	1,03	0,04	0,29745789	0,76611695	0,81757748
YJL173C	678,54	0,97	-0,05	-0,29698757	0,766476	0,81782015
YNCG0040C	1,68	1,64	0,72	0,29618714	0,76708717	0,8183317
YDL157C	794,23	1,04	0,05	0,29519713	0,7678433	0,81897217
YPL100W	1670,07	0,97	-0,04	-0,29505587	0,7679512	0,81897217
YML109W	648,01	1,04	0,06	0,2947322	0,76819846	0,81909524
YDR518W	2068,34	1,03	0,04	0,29407867	0,76869779	0,81948698
YHR041C	656,41	0,97	-0,05	-0,29382284	0,76889328	0,81955474
YKR010C	828,29	0,97	-0,05	-0,29345994	0,76917062	0,81970971
YMR214W	1580,62	0,97	-0,05	-0,29174167	0,77048415	0,82082791
YOR127W	2211,76	1,03	0,04	0,29190776	0,77035715	0,82082791
YNL189W	5676,86	0,97	-0,04	-0,29034746	0,77155044	0,82169056
YOL131W	27,65	1,14	0,18	0,29033699	0,77155844	0,82169056
YEL009C-A	27,43	1,13	0,18	0,28996834	0,77184046	0,82185
YDL223C	1465,97	1,05	0,07	0,28881415	0,7727236	0,82256035
YNCM0008C	8,83	0,82	-0,29	-0,28875038	0,77277241	0,82256035
YNL295W	1098,07	1,03	0,04	0,28827652	0,77313508	0,8228054
YMR176W	1203,51	0,97	-0,05	-0,28764512	0,7736184	0,82317874
YDR323C	462,4	1,04	0,06	0,28699565	0,77411565	0,82356678
YDR395W	3275,85	1,04	0,06	0,28633773	0,77461946	0,82367958
YML081W	1942,2	0,97	-0,05	-0,28644099	0,77454039	0,82367958
YOR032W-A	21,09	1,14	0,19	0,28640011	0,77457169	0,82367958
YGR081C	787,32	1,04	0,05	0,28535434	0,77537268	0,82431264

YNCG0003C	1,81	0,64	-0,65	-0,28521398	0,77548021	0,82431264
YDR482C	475,69	1,04	0,06	0,28354693	0,77675761	0,82552922
YGL153W	956,34	0,97	-0,05	-0,28223802	0,77776101	0,82645422
YPR049C	790,94	0,97	-0,05	-0,28178663	0,77810712	0,82668059
YDL123W	847,35	0,96	-0,05	-0,28117308	0,77857765	0,82703905
YPL277C	223,23	1,05	0,07	0,27921091	0,78008298	0,8284964
YGR264C	5894,24	0,96	-0,07	-0,27621427	0,7823835	0,83037183
YKR032W	0,5	2,33	1,22	0,27622691	0,78237379	0,83037183
YNCE0008C	0,5	2,33	1,22	0,27622691	0,78237379	0,83037183
YNCJ0029W	0,5	2,33	1,22	0,27622691	0,78237379	0,83037183
YCL011C	6459,85	0,97	-0,04	-0,27525094	0,78312345	0,83064593
YNCA0001W	0,51	2,32	1,21	0,27518172	0,78317663	0,83064593
YNCD0014C	0,51	2,32	1,21	0,27518172	0,78317663	0,83064593
YOR096W	13055,36	1,03	0,04	0,27557995	0,78287071	0,83064593
YIL106W	1524,75	1,03	0,05	0,27482258	0,78345255	0,83079673
YHR021W-A	10,73	1,22	0,28	0,27422791	0,78390948	0,83113939
YMR254C	64,99	1,08	0,11	0,27368286	0,78432835	0,83144159
YLL033W	181,69	0,95	-0,07	-0,27322661	0,78467902	0,8316714
YBR284W	311,93	1,05	0,06	0,27235756	0,7853471	0,8322375
YJL013C	1155,17	1,03	0,04	0,26948007	0,78756028	0,83444046
YML051W	2262,08	0,97	-0,04	-0,26893053	0,78798314	0,83461604
YOR133W	12794,96	1,03	0,04	0,26880552	0,78807934	0,83461604
YOR211C	1818,66	0,97	-0,04	-0,26874092	0,78812906	0,83461604
YML048W	5833,26	1,03	0,04	0,26601028	0,79023129	0,83669964
YLR046C	337,19	1,04	0,06	0,26548002	0,7906397	0,83698941
YBR234C	6353,06	1,03	0,04	0,2651845	0,79086733	0,83708773
YLR024C	1070,88	0,96	-0,05	-0,26475863	0,79119541	0,83729232
YDR512C	915,72	1,03	0,05	0,2643074	0,79154306	0,83737492
YER172C	2086,51	1,03	0,05	0,26448223	0,79140836	0,83737492
YLR377C	145,46	0,94	-0,09	-0,26349977	0,7921654	0,83774795
YOL155W-A	72,36	1,07	0,1	0,26360577	0,79208371	0,83774795
YCR071C	789,44	0,97	-0,04	-0,26292852	0,79260568	0,83792826
YNCB0014W	6,65	0,8	-0,31	-0,26303527	0,7925234	0,83792826
YGL176C	532,83	0,97	-0,05	-0,26259963	0,79285918	0,83805364
YER117W	13252,94	1,02	0,03	0,26200101	0,79332066	0,83839876
YGR057C	566,61	0,97	-0,05	-0,26095771	0,79412512	0,83910618
YHL006C	149,91	0,95	-0,08	-0,26027648	0,79465051	0,83951853
YCR095W-A	74,34	1,08	0,11	0,25958497	0,79518393	0,83993922
YPR154W	6125,34	1,02	0,03	0,25898369	0,79564783	0,84028635
YEL051W	6745,17	1,02	0,03	0,25852731	0,79599997	0,84051536
YCR017C	4586,57	0,97	-0,04	-0,2566916	0,79741685	0,84186837
YBL043W	151,76	1,05	0,07	0,25535144	0,79845167	0,84281764
YPL214C	1338,31	1,03	0,04	0,25517342	0,79858916	0,84281955
YHR166C	630,84	0,97	-0,04	-0,2533299	0,80001329	0,84417913
YNL335W	63,08	0,93	-0,1	-0,24738874	0,80460739	0,84888266
YHR185C	3,31	0,74	-0,44	-0,24509966	0,80637926	0,85060757
YAL053W	2675,3	0,97	-0,04	-0,24450188	0,80684215	0,85080689
YHR058C	448,24	1,03	0,05	0,24450793	0,80683746	0,85080689
YMR001C-A	40,65	1,08	0,12	0,24344362	0,80766176	0,85152662
YBL044W	9,59	0,85	-0,24	-0,24072263	0,8097701	0,85224441

YBL093C	1422,78	1,03	0,04	0,2384039	0,81156784	0,85224441
YCR050C	0,32	0,44	-1,17	-0,23934476	0,81083826	0,85224441
YEL076C-A	0,32	0,44	-1,18	-0,24049699	0,80994499	0,85224441
YGL195W	5870,74	0,97	-0,05	-0,23831704	0,81163521	0,85224441
YGR162W	6199,39	0,96	-0,05	-0,238543	0,81145997	0,85224441
YIL169C	6745,24	0,97	-0,04	-0,23838537	0,81158222	0,85224441
YLL066W-B	0,32	0,44	-1,17	-0,23934476	0,81083826	0,85224441
YNCB0011C	0,32	0,44	-1,17	-0,23934476	0,81083826	0,85224441
YNCE0010W	0,32	0,44	-1,18	-0,24049699	0,80994499	0,85224441
YNCG0035W	0,32	0,44	-1,17	-0,23934476	0,81083826	0,85224441
YNCG0044C	0,32	0,44	-1,17	-0,23934476	0,81083826	0,85224441
YNCG0046W	0,32	0,44	-1,18	-0,24164574	0,80905468	0,85224441
YNCI0005W	0,32	0,44	-1,17	-0,23934476	0,81083826	0,85224441
YNCI0011W	0,32	0,44	-1,17	-0,23934476	0,81083826	0,85224441
YNCK0002C	0,32	0,44	-1,17	-0,23934476	0,81083826	0,85224441
YNCL0007W	0,32	0,44	-1,18	-0,24049699	0,80994499	0,85224441
YNCL0027W	0,32	0,44	-1,18	-0,24049699	0,80994499	0,85224441
YNCL0034W	5,81	0,82	-0,29	-0,23846922	0,81151719	0,85224441
YNCM0028C	1,81	0,63	-0,67	-0,23841986	0,81155546	0,85224441
YNCN0015W	0,32	0,44	-1,18	-0,24164574	0,80905468	0,85224441
YNCO0004C	0,32	0,44	-1,17	-0,23934476	0,81083826	0,85224441
YNCP0012W	0,32	0,44	-1,18	-0,24049699	0,80994499	0,85224441
YOL013W-A	0,32	0,44	-1,17	-0,23934476	0,81083826	0,85224441
YCR048W	1272,51	0,97	-0,04	-0,23748465	0,81228082	0,85277818
YNCO0021C	8,67	0,84	-0,24	-0,23701373	0,81264614	0,85301755
YER155C	4792,97	1,03	0,04	0,23404278	0,81495177	0,85529319
YLR413W	1666,11	0,96	-0,06	-0,23346159	0,81540299	0,8556222
YPL220W	10675,92	0,98	-0,03	-0,23300326	0,81575888	0,85585106
YMR182C	716,68	1,03	0,04	0,23209188	0,81646665	0,85644897
YER189W	42,2	0,92	-0,12	-0,23165883	0,81680301	0,85665715
YBL087C	7696,2	1,02	0,04	0,23028129	0,81787319	0,85763475
YFL055W	262,26	1,04	0,06	0,22939381	0,81856285	0,85821306
YGR240C	22411,2	0,97	-0,04	-0,22835619	0,81936935	0,85876875
YOL045W	1476,56	0,97	-0,05	-0,2284915	0,81926417	0,85876875
YLR237W	533,91	1,03	0,04	0,2277065	0,81987442	0,85915315
YGR171C	932,75	0,97	-0,04	-0,2247505	0,82217338	0,86127167
YPL266W	1424,55	0,97	-0,05	-0,22486222	0,82208646	0,86127167
YNCM0033C	1,08	0,63	-0,66	-0,22418845	0,82261067	0,86158446
YJR112W	293,83	1,04	0,05	0,2230566	0,82349146	0,86227457
YLR236C	19,95	0,9	-0,16	-0,22281976	0,82367579	0,86227457
YPR086W	2421,24	0,98	-0,03	-0,22280667	0,82368597	0,86227457
YOL024W	15,17	0,88	-0,19	-0,22157907	0,82464158	0,86312948
YHR084W	1971	0,98	-0,03	-0,21927923	0,82643254	0,86485831
YAL025C	1368,4	1,03	0,05	0,21757365	0,82776132	0,86581168
YER190W	32,4	1,09	0,12	0,21774843	0,82762513	0,86581168
YGR121W-A	33,82	1,09	0,12	0,2175376	0,82778941	0,86581168
YNCP0010W	2,48	0,74	-0,44	-0,21739431	0,82790106	0,86581168
YBR292C	55,48	1,07	0,09	0,21691805	0,82827221	0,86598241
YKL214C	922,07	0,98	-0,03	-0,21682706	0,82834312	0,86598241
YGL179C	762,9	1,03	0,05	0,21471532	0,82998928	0,86755736

YOL148C	1277,77	1,02	0,04	0,21346388	0,83096516	0,86843129
YJR159W	8,71	1,16	0,21	0,2091698	0,83431569	0,87178622
YPL209C	211,33	1,04	0,05	0,20794794	0,83526962	0,8726362
YBR055C	1402,37	0,98	-0,03	-0,20766894	0,83548747	0,87271703
YML039W	115,56	1,04	0,06	0,20558185	0,83711756	0,87427274
YEL009C	16416,96	0,98	-0,03	-0,20458354	0,83789752	0,87494022
YCR087C-A	808,78	1,03	0,04	0,20350929	0,83873699	0,87566961
YER157W	1185,18	0,98	-0,03	-0,20099286	0,84070416	0,87756686
YNL270C	98,41	1,06	0,08	0,20082327	0,84083676	0,87756686
YLR431C	651,58	0,98	-0,04	-0,19993696	0,84152988	0,87814273
YKL114C	1455,23	0,98	-0,03	-0,19954526	0,84183624	0,87816738
YNR063W	220,24	1,04	0,05	0,19969744	0,84171722	0,87816738
YFL030W	644,29	1,04	0,06	0,19922679	0,84208535	0,87827975
YHR096C	809,64	1,03	0,04	0,19891656	0,84232801	0,87838537
YOR256C	2470,73	1,02	0,03	0,19825516	0,84284543	0,87877741
YNCG0017W	2,58	0,78	-0,35	-0,19779457	0,8432058	0,87900561
YNL255C	10898,39	1,03	0,04	0,19757784	0,84337538	0,87903487
YAR020C	76,45	1,06	0,08	0,19534356	0,845124	0,88070966
YDR231C	1276,52	0,97	-0,04	-0,19328974	0,84673206	0,88208948
YOL055C	606,25	0,98	-0,04	-0,19345184	0,84660512	0,88208948
YMR164C	937,87	1,02	0,03	0,19140702	0,84820672	0,88347755
YCR011C	5699,43	1,02	0,03	0,19117482	0,84838863	0,88351889
YPR203W	3,93	1,24	0,31	0,19038281	0,84900916	0,88401692
YOR374W	10237,87	0,97	-0,05	-0,19005348	0,84926722	0,88413742
YBR023C	1634,19	0,97	-0,04	-0,18985478	0,84942292	0,88415134
YKL033W	808,35	1,02	0,03	0,18791748	0,85094134	0,88558345
YML121W	1418,02	1,02	0,03	0,18435918	0,85373169	0,88833857
YOL114C	184,27	0,97	-0,05	-0,18258484	0,85512378	0,88963807
YIL132C	188,03	0,97	-0,05	-0,18116054	0,85624157	0,89065181
YBR117C	451,87	0,96	-0,06	-0,17849811	0,85833181	0,89252715
YOL165C	6,62	1,15	0,21	0,17861149	0,85824277	0,89252715
YGL206C	4520,95	0,98	-0,03	-0,17708892	0,85943854	0,89352841
YKL188C	918,37	1,02	0,03	0,17676923	0,85968966	0,89363992
YMR257C	658,74	0,98	-0,03	-0,17615577	0,86017157	0,89399126
YNCC0001C	1,65	0,74	-0,43	-0,1758405	0,86041925	0,89400619
YNL246W	1626,34	1,02	0,03	0,17577111	0,86047377	0,89400619
YMR002W	3408,02	1,02	0,02	0,1744734	0,86149344	0,89491593
YKL039W	5952,34	0,98	-0,02	-0,17388581	0,8619552	0,8952459
YMR092C	5358,61	0,98	-0,02	-0,17304935	0,86261264	0,89577895
YGR042W	833,3	0,98	-0,03	-0,17172397	0,86365454	0,89671102
YMR290C	3470,98	1,03	0,04	0,17046478	0,86464463	0,89758898
YHR133C	1829,4	0,98	-0,02	-0,17015395	0,86488907	0,89769271
YHR022C-A	30,09	0,92	-0,11	-0,16912382	0,86569925	0,89838352
YBL052C	1474,88	1,02	0,03	0,16821436	0,86641465	0,89897575
YBR065C	590,44	1,02	0,03	0,16780624	0,86673571	0,89915869
YNCP0015C	104,23	0,96	-0,06	-0,16665365	0,86764257	0,89994919
YJL111W	5982,18	1,02	0,03	0,16552029	0,86853448	0,90072391
YNCM0015W	3,59	0,83	-0,26	-0,16517709	0,8688046	0,90085364
YGR204C-A	39,19	0,93	-0,11	-0,16374139	0,86993473	0,90187492
YNCP0022W	2,5	1,26	0,33	0,16277092	0,87069881	0,90251644

YKR101W	511,54	1,02	0,03	0,16133986	0,87182573	0,90353377
YGL012W	5340,76	1,02	0,02	0,1606742	0,87235001	0,9039022
YJR146W	89,8	1,04	0,06	0,16051902	0,87247224	0,9039022
YBR253W	714,26	0,98	-0,03	-0,15958793	0,87320569	0,90451121
YFL028C	1217,7	0,98	-0,03	-0,15876184	0,87385651	0,90493651
YOR335C	6675,47	1,02	0,03	0,15869698	0,87390762	0,90493651
YLR165C	584,28	1,02	0,03	0,15506767	0,87676797	0,90759584
YNCG0043C	1,52	1,31	0,39	0,15512479	0,87672294	0,90759584
YFR020W	631,13	0,98	-0,03	-0,15462561	0,87711648	0,90780533
YNCE0011C	45,58	0,95	-0,08	-0,15439842	0,8772956	0,90783946
YOR001W	1705,63	0,98	-0,03	-0,15333733	0,87813226	0,9085539
YCL030C	16131,03	1,01	0,02	0,15299695	0,87840068	0,90864209
YNL252C	1237,79	1,02	0,03	0,15285827	0,87851004	0,90864209
YML032C	1417,54	0,98	-0,02	-0,15134359	0,87970469	0,90972624
YNL067W-B	1,75	0,8	-0,32	-0,14921723	0,88138222	0,91130932
YPL099C	556,36	0,98	-0,03	-0,1486152	0,88185728	0,91164876
YGR035W-A	226,33	0,97	-0,04	-0,14697918	0,88314845	0,91244654
YJL179W	825,15	0,98	-0,03	-0,14713803	0,88302307	0,91244654
YNL318C	16,47	1,08	0,12	0,14712363	0,88303444	0,91244654
YPR162C	1161,09	1,02	0,02	0,14689295	0,88321652	0,91244654
YER102W	10069,16	0,98	-0,03	-0,14571226	0,88414855	0,91325753
YMR010W	1258,58	0,98	-0,03	-0,14522558	0,88453277	0,9135025
YNCJ0009C	5,19	1,14	0,19	0,1449361	0,88476132	0,91358666
YOR112W	1570,64	1,02	0,02	0,14373954	0,88570615	0,91440752
YPL029W	600,71	0,98	-0,03	-0,14355651	0,88585069	0,91440752
YBL076C	13787,16	0,98	-0,03	-0,14319586	0,8861355	0,91454955
YGR063C	456,75	0,98	-0,03	-0,14278591	0,88645927	0,91473172
YKL184W	1762,23	1,02	0,02	0,14215159	0,88696027	0,91509669
YFL037W	9553,16	1,01	0,02	0,1405596	0,88821787	0,91624201
YBR217W	595,51	0,98	-0,03	-0,13880352	0,88960541	0,9174309
YDL045W-A	799,44	1,02	0,03	0,13872714	0,88966577	0,9174309
YGR147C	1408,67	1,02	0,02	0,13836137	0,88995483	0,91757666
YNCG0012W	4,26	0,87	-0,19	-0,13724877	0,89083416	0,91833087
YGL139W	1099,11	1,02	0,02	0,13434539	0,89312946	0,92054425
YMR074C	1210,41	0,98	-0,02	-0,13346879	0,89382265	0,92110588
YGR135W	5819,9	0,99	-0,02	-0,13097195	0,89579749	0,9229879
YNR058W	141,33	1,03	0,04	0,1304903	0,89617853	0,92322737
YGR133W	592,04	0,98	-0,03	-0,12788359	0,8982411	0,92505217
YKL092C	1230,06	0,98	-0,02	-0,12787525	0,8982477	0,92505217
YFR044C	9398,59	0,99	-0,02	-0,12687835	0,89903669	0,92571124
YGR036C	452,13	0,98	-0,02	-0,12587578	0,89983026	0,9263748
YBR172C	2268,56	0,99	-0,02	-0,12560342	0,90004585	0,92644322
YJL043W	39,83	0,95	-0,07	-0,12513498	0,90041669	0,92667138
YDR009W	289,74	0,98	-0,03	-0,12485098	0,90064152	0,92669043
YGL148W	8600,5	0,99	-0,02	-0,12473475	0,90073355	0,92669043
YHR093W	30,87	0,95	-0,07	-0,12415899	0,90118939	0,92700588
YER184C	593,41	1,02	0,02	0,12363158	0,90160699	0,92728189
YGL099W	1924,87	0,98	-0,03	-0,12313332	0,90200153	0,9275341
YKL166C	812,76	0,99	-0,02	-0,12284936	0,90222639	0,92761177
YOR370C	3776,46	0,99	-0,02	-0,12244437	0,9025471	0,92778795

YCR089W	1154,07	0,99	-0,02	-0,1216904	0,90314422	0,92824816
YNCB0016W	3,2	1,17	0,23	0,12066503	0,90395636	0,92892918
YOL135C	814,52	1,01	0,02	0,11973962	0,90468941	0,92952872
YBL008W	644,81	0,98	-0,02	-0,11903478	0,9052478	0,92994863
YIL042C	904,54	1,02	0,02	0,1185835	0,90560534	0,93016209
YHR169W	542,2	1,02	0,02	0,11698496	0,90687197	0,93130909
YML078W	2894,96	1,01	0,02	0,11665	0,90713741	0,9314277
Q0285	1,42	1,21	0,27	0,11615311	0,9075312	0,93152409
YNCB0013W	1,42	1,21	0,27	0,11615311	0,9075312	0,93152409
YFL066C	63,41	0,97	-0,05	-0,11590791	0,90772553	0,93156963
YHR199C-A	263,86	1,02	0,03	0,11468313	0,90869628	0,93235854
YIL023C	1086,08	1,02	0,02	0,11455932	0,90879443	0,93235854
YJR060W	1609,88	0,99	-0,02	-0,11371759	0,90946167	0,93242711
YKL003C	1022,79	1,02	0,02	0,11403073	0,90921344	0,93242711
YNL146C-A	59,36	1,04	0,06	0,11371861	0,90946086	0,93242711
YPR189W	1777,52	1,01	0,02	0,11372098	0,90945898	0,93242711
YLR204W	774,51	1,02	0,02	0,11298243	0,91004449	0,93280092
YPL003W	1293,3	0,98	-0,02	-0,11287887	0,91012659	0,93280092
YER175W-A	96,29	1,03	0,04	0,11258574	0,91035899	0,93288519
YIR021W-A	4,6	0,88	-0,19	-0,1117329	0,91103519	0,93342415
YNCG0023C	4,92	0,9	-0,15	-0,11112558	0,91151677	0,93376355
YOL067C	486,96	1,02	0,02	0,11076287	0,91180439	0,93390418
YIR030C	198,25	0,98	-0,03	-0,10730901	0,91454383	0,93652654
YML066C	256,52	1,02	0,03	0,10712106	0,91469294	0,93652654
YNL029C	676,21	0,99	-0,02	-0,10696472	0,91481697	0,93652654
YNCG0033W	4,87	1,14	0,19	0,10653986	0,91515403	0,93671722
YBR298C-A	4,93	0,9	-0,15	-0,10449534	0,91677627	0,93806855
YNL282W	291,45	0,98	-0,02	-0,10452053	0,91675628	0,93806855
YER132C	812,07	1,02	0,02	0,10390129	0,91724769	0,93839635
YNL143C	102,67	0,97	-0,05	-0,10356673	0,91751319	0,93851341
YIL038C	2158,03	1,01	0,02	0,10226323	0,91854774	0,93926231
YNL187W	420,38	0,98	-0,02	-0,10234482	0,91848298	0,93926231
YLR141W	538,23	0,99	-0,02	-0,1012475	0,91935399	0,93993202
YNCL0009C	0,76	1,3	0,38	0,09897393	0,92115897	0,94146751
YNCN0017W	0,76	1,3	0,38	0,09897393	0,92115897	0,94146751
YMR003W	254,7	1,02	0,03	0,09854951	0,92149596	0,94150212
YPR032W	1178,06	0,99	-0,02	-0,09868492	0,92138844	0,94150212
YNCE0028C	0,82	0,78	-0,36	-0,09756997	0,92227377	0,94214186
YHL029C	597,23	1,01	0,02	0,09653334	0,923097	0,94271623
YMR236W	1252,28	1,01	0,02	0,09647976	0,92313954	0,94271623
YGL048C	6692,91	1,01	0,01	0,09612666	0,92341998	0,94284762
YPR192W	242,1	0,98	-0,03	-0,09589891	0,92360086	0,94287733
YOL085C	91,14	0,98	-0,03	-0,09550779	0,92391151	0,94303948
YPL138C	707,27	0,99	-0,02	-0,09418043	0,92496583	0,94396053
YDR470C	999,23	1,01	0,02	0,09369122	0,92535444	0,944202
YOL162W	29,35	0,96	-0,05	-0,09331569	0,92565277	0,94435129
YOR234C	6037,37	0,99	-0,01	-0,09298619	0,92591453	0,94446323
YOL072W	831,84	1,01	0,02	0,09109259	0,92741902	0,94584255
YNCJ0008W	4,57	0,91	-0,13	-0,09001423	0,92827591	0,94656105
YGR282C	15867,92	0,99	-0,01	-0,08951037	0,92867631	0,94681392

YHR148W	438,54	1,01	0,02	0,08838943	0,92956716	0,94756666
YPL056C	369,6	0,99	-0,02	-0,08804121	0,92984392	0,94769326
YMR158C-A	52,98	1,03	0,04	0,08665306	0,93094729	0,94866217
YML049C	2047,14	1,01	0,02	0,08509978	0,93218207	0,94976464
YLR186W	1119,83	0,99	-0,01	-0,08318698	0,93370286	0,95115811
YDL193W	1600,17	0,99	-0,01	-0,08233982	0,93437649	0,95168827
YNCG0018C	3,43	0,91	-0,13	-0,08148222	0,93505846	0,95222675
YOR124C	4699,42	1,01	0,01	0,08126838	0,93522852	0,95224382
YDR499W	887,67	1,01	0,01	0,08088315	0,93553488	0,9522709
YER140W	611,57	0,99	-0,02	-0,08084942	0,9355617	0,9522709
YBR057C	1536,64	0,99	-0,01	-0,0793218	0,93677667	0,95304101
YLR053C	88,5	0,98	-0,03	-0,07955765	0,93658908	0,95304101
YNL229C	2619,08	0,99	-0,01	-0,07931943	0,93677856	0,95304101
YBR154C	2254,48	1,01	0,01	0,07787697	0,93792592	0,9537397
YNCF0014C	0,92	0,85	-0,24	-0,07790649	0,93790244	0,9537397
YNCL0040W	0,92	0,85	-0,24	-0,07790649	0,93790244	0,9537397
YJL057C	2682,88	1,01	0,02	0,0762974	0,9391825	0,95486116
YBR259W	642,81	1,01	0,01	0,07566465	0,9396859	0,95521663
YFR013W	2296,42	1,01	0,01	0,07301601	0,94179338	0,9572023
YBR167C	410,63	0,99	-0,01	-0,07218294	0,94245632	0,95756276
YNCM0032C	20,84	0,97	-0,05	-0,07233677	0,9423339	0,95756276
YKR034W	9,98	0,95	-0,08	-0,0710705	0,94334165	0,95830553
YMR070W	1289,12	1,01	0,01	0,06973152	0,94440736	0,95923128
YMR198W	679,51	1,01	0,01	0,06698482	0,94659379	0,96129485
YKL096C-B	3,08	0,92	-0,12	-0,06660995	0,94689222	0,96144075
YNL106C	1401,12	1,01	0,01	0,06579227	0,94754321	0,96178732
YPL010W	3098,66	1,01	0,01	0,06584286	0,94750293	0,96178732
YNL034W	58,37	1,03	0,04	0,0655805	0,94771181	0,9618013
YNCD0019C	0,67	1,17	0,22	0,06480949	0,94832569	0,96210994
YNCL0001W	0,67	1,17	0,22	0,06480949	0,94832569	0,96210994
YNCL0049C	2	1,1	0,14	0,06419722	0,94881319	0,96244735
YFL067W	125,94	1,01	0,02	0,06250155	0,95016342	0,96365962
YDR114C	1,99	1,1	0,14	0,06132969	0,95109664	0,96444864
YNCH0003C	0,92	0,86	-0,22	-0,06074067	0,95156574	0,96476683
YNL047C	752,07	0,99	-0,01	-0,06053532	0,95172929	0,96477518
YNCM0011W	23,98	0,97	-0,04	-0,0600231	0,95213723	0,96503124
YER034W	465,26	0,99	-0,01	-0,05981556	0,95230253	0,96504133
YAR023C	184,92	0,99	-0,02	-0,05697029	0,95456886	0,96670717
YNCB0012W	0,66	1,17	0,23	0,05698427	0,95455772	0,96670717
YNCK0005C	0,66	1,17	0,23	0,05698427	0,95455772	0,96670717
YNCL0045W	0,66	1,17	0,23	0,05698427	0,95455772	0,96670717
YIL040W	651,99	0,99	-0,01	-0,05613137	0,95523716	0,96722628
YMR068W	492,14	0,99	-0,01	-0,05533507	0,95587152	0,96758531
YNCA0004W	1,33	1,11	0,15	0,05529524	0,95590326	0,96758531
YGR052W	876,26	0,99	-0,01	-0,05499253	0,95614441	0,96767173
YKL045W	1456,11	0,99	-0,01	-0,05448007	0,95655269	0,96782193
YNCJ0024W	0,67	1,16	0,21	0,05441514	0,95660442	0,96782193
YOL012C	1491,7	0,99	-0,01	-0,05404096	0,95690253	0,96796589
YDR052C	2162,24	1,01	0,01	0,05204914	0,95848954	0,96941339
YML054C-A	48,67	1,02	0,02	0,05034689	0,95984596	0,97062723

YJL118W	302,03	1,01	0,01	0,04818367	0,96156987	0,97221224
YFL017W-A	344,99	1,01	0,01	0,04736379	0,96222329	0,97271458
YMR268C	385,96	1,01	0,01	0,04683659	0,96264347	0,972981
YMR180C	651,7	1,01	0,01	0,04593934	0,9633586	0,97354541
YDR115W	414,66	0,99	-0,01	-0,04383729	0,9650341	0,97508
YDL070W	6402,77	1	-0,01	-0,04270926	0,9659333	0,9752836
YDL175C	661,67	0,99	-0,01	-0,04309621	0,96562484	0,9752836
YIL089W	226,72	0,99	-0,01	-0,04306132	0,96565265	0,9752836
YLR278C	3070,21	1	-0,01	-0,04259973	0,96602061	0,9752836
YOR330C	1683,69	1	0,01	0,0429715	0,96572425	0,9752836
YJR024C	799,33	1	0,01	0,04150109	0,96689642	0,97600919
YBR121C	16100	1	0,01	0,04042226	0,96775648	0,97667223
YGL010W	396,79	1,01	0,01	0,04028274	0,96786772	0,97667223
YDL232W	576,91	1,01	0,01	0,03997521	0,96811289	0,97676097
YJL222W	63,1	0,99	-0,02	-0,03875257	0,96908766	0,97758567
YBR053C	3277,76	1	-0,01	-0,03741096	0,97015733	0,97850582
YNCM0014W	2,16	0,95	-0,08	-0,03681941	0,970629	0,97882262
YFR048W	877,13	1	0,01	0,03591863	0,97134724	0,97938793
YNL325C	664,3	1	0,01	0,03569819	0,97152301	0,97940619
YDR406W	4120,61	1	0,01	0,03514299	0,97196572	0,97960738
YPL174C	795,33	0,99	-0,01	-0,03505238	0,97203798	0,97960738
YCL055W	525,71	1	-0,01	-0,03473282	0,97229279	0,97970524
YLR324W	2173,46	1	0	0,03440767	0,97255207	0,97980757
YIL115C	3655,79	1	0	0,03272423	0,9738945	0,98100091
YGL067W	1619,53	1	0,01	0,03241451	0,97414149	0,98109062
YHR072W-A	2266,08	1	-0,01	-0,03220814	0,97430606	0,9810973
YNL033W	54,08	0,99	-0,01	-0,03174272	0,97467722	0,98131198
YNR007C	1153,59	1	-0,01	-0,03154006	0,97483884	0,98131565
YHR173C	29,8	1,01	0,02	0,03022728	0,97588579	0,98221039
YMR035W	558,93	1	-0,01	-0,02952539	0,97644557	0,98261459
YDR296W	1740,7	1	0	-0,02851707	0,97724976	0,98326457
YNCD0013C	1,68	1,05	0,07	0,02739649	0,9781435	0,98400444
YNCE0015C	8,08	1,02	0,03	0,02683936	0,97858786	0,98429207
YDL056W	1802,86	1	0	-0,02383681	0,98098278	0,98622189
YLR161W	25,87	1,01	0,02	0,0242106	0,98068462	0,98622189
YNR010W	403,26	1	0,01	0,02393633	0,9809034	0,98622189
YNCP0021W	45,91	0,99	-0,01	-0,02351661	0,98123819	0,98631903
YHR083W	2814,46	1	0	0,0231195	0,98155495	0,98647781
YGR045C	18,29	0,99	-0,02	-0,02257486	0,98198939	0,98675479
YCL068C	1,58	0,97	-0,05	-0,0219934	0,98245322	0,98690158
YER072W	7177,23	1	0	0,02215864	0,98232141	0,98690158
YJR135C	291,27	1	0	0,02100674	0,98324028	0,98753252
YLR093C	3360,2	1	0	0,02061015	0,98355664	0,98769057
YER040W	1086,58	1	0	0,02029485	0,98380817	0,98778347
YOR198C	9590,72	1	0	-0,01970211	0,98428101	0,98809852
YBR030W	723,01	1	0	0,01790819	0,9857121	0,98922792
YNCG0039W	1,58	0,97	-0,05	-0,0178926	0,98572454	0,98922792
YPL072W	718,19	1	0	0,01710368	0,9863539	0,98969964
YBL071C-B	2,82	0,98	-0,03	-0,015908	0,98730779	0,99033683
YGR292W	189,63	0,99	-0,01	-0,01591033	0,98730593	0,99033683

YDL024C	163,16	1	0	0,01530289	0,98779054	0,9906611
YNR032C-A	402,09	1	0	-0,01509986	0,98795251	0,9906636
YGR123C	1050,5	1	0	0,01416143	0,98870119	0,99125433
YDR081C	1345,58	1	0	0,01298513	0,98963966	0,99203511
YNL292W	977,52	1	0	0,01073003	0,99143884	0,9936783
YHL007C	3055,06	1	0	-0,01005794	0,99197506	0,99405534
YPR163C	9280,47	1	0	0,00907188	0,99276179	0,99468326
YDL132W	4853,77	1	0	-0,00847283	0,99323974	0,99500165
YMR143W	17579,6	1	0	-0,00800844	0,99361026	0,99521234
YPR187W	2303,67	1	0	-0,0077301	0,99383233	0,99527429
YHL023C	1405,6	1	0	0,00727081	0,99419879	0,99532036
YNCM0023C	7,77	1,01	0,01	0,00740872	0,99408875	0,99532036
YDR202C	1742,76	1	0	0,00610911	0,99512567	0,99608776
YNCP0006C	2,25	1,01	0,01	0,00561999	0,99551592	0,99631785
YER012W	5142,58	1	0	0,00528639	0,99578209	0,99642371
YLR037C	245,26	1	0	0,0040071	0,9968028	0,99728443
YAR061W	2,25	1	0,01	0,00335139	0,99732598	0,99764718
YJR156C	96,34	1	0	0,00111053	0,99911392	0,99927478
YEL008W	57,82	1	0	-0,00068604	0,99945262	0,99945262

_p_value
